# Global, regional, and national burden of acute hepatitis (1990–2021): a systematic analysis of the global burden of disease in 2021

**DOI:** 10.3389/fmed.2025.1620371

**Published:** 2025-09-10

**Authors:** Zhixun Bai, Yanjin Chen, Jiaxi Chen, Wenyi Pang, Rubin Zheng, Miao Deng, Rui Sun, Yongping Lan, Houze Li

**Affiliations:** ^1^Department of Nephrology, People’s Hospital of Qianxinan Prefecture, Xingyi, Guizhou, China; ^2^Clinical College, Zunyi Medical University, Zunyi, Guizhou, China; ^3^Clinical College, Guizhou Medical University, Guiyang, Guizhou, China

**Keywords:** acute hepatitis, incidence, mortality, DALYs, global burden of disease

## Abstract

**Background:**

Acute viral hepatitis (AVH) remains a global health concern, with significant variations in incidence, mortality, and DALYs across different regions, age groups, genders, and socioeconomic levels. This study explores AVH trends using data from the Global Burden of Disease (GBD) database (1990–2021).

**Methods:**

We analyzed age-standardized incidence, mortality, and DALY rates across 204 countries and 27 super-regions. Trends were quantified using the estimated annual percentage change (EAPC) and decomposition analysis to assess the contributions of population growth, aging, and epidemiological shifts. Autoregressive integrated moving average (ARIMA) and age-period-cohort (APC) models were also applied, while global health inequalities were evaluated via the Slope Index of Inequality (SII) and Concentration Index (CI).

**Results:**

Incidence and mortality rates were notably higher in individuals under 20 and over 80 years, with males consistently at greater risk than females. Acute Hepatitis A predominates in low-SDI regions, whereas Acute Hepatitis B is more prevalent in high-SDI regions. Projections indicate a continued decline in AVH burden by 2031, driven by population dynamics and epidemiological changes.

**Conclusion:**

While global AVH burden is decreasing, significant disparities persist, warranting tailored interventions to enhance resource equity in high-SDI regions and strengthen healthcare infrastructure in low-SDI areas.

## Introduction

In recent years, acute hepatitis has gradually become one of the leading causes of increased mortality worldwide (1, 2). According to a previous study based on the 2021 Global Burden of Disease data, the age-standardized incidence rate of acute hepatitis was the second highest, with 3411.5 cases per 100,000 people (3). Due to its characteristics, including a large number of patients, prolonged duration, poor treatment outcomes, and high mortality, acute hepatitis poses a significant threat to human health (4), making it an urgent and important public health issue.

Acute hepatitis is a general term for a group of diseases characterized by acute inflammation of the liver parenchyma or hepatocyte injury, leading to elevated liver function indicators. Its etiology is diverse, with viral infections being the most common cause. These include Acute Hepatitis A (AHA), Acute Hepatitis B (AHB), Acute Hepatitis C (AHC), Acute Hepatitis D (AHD), and Acute Hepatitis E (AHE) (5, 6).

In the past few decades, research on acute hepatitis has been primarily focused on hepatitis B and C due to their large share of mortality and the critical nature of their disease progression (7). This focus has somewhat limited the progress in understanding acute viral hepatitis (AVH), particularly in relation to the continuous challenge posed by chronic Hepatitis B Virus (HBV) and Hepatitis C Virus (HCV). A previous study by Zeng et al. (5), based on the GBD 2019, provided a relatively comprehensive description of the global burden of acute viral hepatitis (AVH). However, the burden of AVH is evolving in the context of rapidly changing global economies and populations, and it varies over time, between countries, and even within countries. GBD 2021 updated data from more countries and regions and improved the methodology for estimating disease burden, enhancing the accuracy and representativeness of the study (8). Therefore, it is necessary to update information on AVH in a timely manner, and enhancing our understanding of the burden of AVH is crucial for developing global intervention strategies.

The Global Burden of Disease (GBD) database is a tool that provides quantitative data on health losses caused by 100 of diseases, injuries, and risk factors, including data from surveys, censuses, vital statistics, and other health-related sources such as mortality and disability. GBD 2021 offers new population estimates for 204 countries and regions from 1950 to 2021, as well as for 811 additional subnational regions, from which data on acute hepatitis from 1990 to 2021 were collected. However, data on Hepatitis D virus (HDV), which is the smallest genome among known RNA viruses (approximately 1,682 base pairs) and primarily transmitted through one of two routes (superinfection or coinfection) when co-infected with Hepatitis B virus (9), were not included in GBD 2021. Therefore, this report only discusses AHA, AHB, AHC, and AHE. This study used the GBD results tool to extract estimates of mortality, morbidity, and disability-adjusted life years (DALYs), along with their 95% uncertainty intervals (UIs). It also utilized models such as the autoregressive integrated moving average model and the age-period-cohort model to explore the temporal trends of acute viral hepatitis (AVH), in order to assess the AVH burden across different regions and countries. This analysis aims to clarify the global burden pattern of AVH and provide scientific evidence to guide public health decision-making.

## Materials and methods

### Data source

All the data used in this study were obtained from GBD 2021. This database collects data through international health surveys, hospital records, clinical studies, death records, and registries, using Bayesian models and other methods to estimate and model the original data and disease burden. It analyzes and assesses diseases, injuries, and risk factors across different regions of the world. In addition, the GBD 2021 team conducted a rigorous assessment of data quality and implemented a series of quality control measures to ensure the reliability and accuracy of the data. Further detailed descriptions of the GBD data can be found in previous studies (8), and will not be repeated here. The detailed data used in this study can be accessed at https://ghdx.healthdata.org/gbd-2021.

This study collected annual incidence cases, age-standardized incidence rates (ASIR), mortality numbers, age-standardized mortality rates (ASMR), disability-adjusted life years (DALYs), and age-standardized DALYs rates for acute viral hepatitis (AVH) from 1990 to 2021 through GBD 2021. Data were available for 204 countries and regions, and the data were classified by sex, region, country, and AVH type (AHA, AHB, AHC, and AHE).

The Socio-demographic Index (SDI) is a composite indicator that reflects a country’s level of development, including per capita income, the average educational attainment of the population aged 15 and above, and the total fertility rate for those under 25 years old. It is strongly correlated with health outcomes. The SDI ranges from 0 to 1, where 0 represents the lowest level of development and 1 represents the highest level of development (10). Based on SDI values, the 204 countries and regions are categorized into five groups: high SDI, high-middle SDI, middle SDI, low-middle SDI, and low SDI. Additionally, the world is divided into 21 geographical regions.

### Statistical analysis

This study quantifies the burden of AVH and its different types using age-standardized incidence rates (ASIR), age-standardized mortality rates (ASMR), age-standardized DALYs rates (DALYs rate), and estimated annual percentage changes (EAPC). Standardization is necessary when comparing populations with different age structures or when the age distribution of the same population changes over time. The ASR (per 100,000 population) in accordance with the direct method is calculated by summing up the products of the age-specific rates (a_i_, where I denotes the ith age class) and the number of persons (or weight) (w_i_) in the same age subgroup I of the chosen reference standard population, then dividing the sum of standard population weights, i.e.,


$$\mathrm{ASR}=\frac{\sum_{\left\{i=1\right\}}^{A}a_{i}w_{i}}{\sum_{\left\{i=1\right\}}^{A}w_{i}}\times 100,000$$

This study uses the Estimated Annual Percentage Change (EAPC) and 95% confidence intervals (CI) to quantify the annual average change in age-standardized rates (ASR) of AVH from 1990 to 2021. A log-linear regression model was employed to calculate the EAPC: *y = α + βx+ε,* where EAPC = 100 × (exp(β) –1), *y* = ln(ASR), and *x* = calendar year. The 95% CI can also be derived from the linear regression model. When both the EAPC and the upper limit of the 95% CI are <0, the ASR is considered to be in a declining trend. When both the EAPC and the lower limit of the 95% CI are >0, the ASR is considered to be on the rise. The 95% uncertainty interval (UI) is calculated by obtaining the 2.5th and 97.5th values from the 1,000 estimates for each metric (11). To explore the factors influencing the EAPC, the study assessed the relationship between EAPC and ASR at the national level. Decomposition analysis considers the contributions of population size, age structure, and epidemiologic changes—specifically referring to changes in age- and population-standardized incidence and deaths. These epidemiologic changes reflect the evolving patterns of CKD incidence and outcomes over time, independent of demographic shifts (12).

### Autoregressive integrated moving average model

The ARIMA model, introduced by George EP Box and Gwilym M. Jenkins in their 1970 book *Time Series Analysis*, is highly accurate for both short-term and long-term forecasting. The ARIMA (p, d, q) model can be understood as a combination of non-stationary data patterns, integrating the Autoregressive (AR) model with the Moving Average (MA) model (13). It can be expressed by the following equation: *Y_t_ = φ_1_Y_t-1_ + φ_2_Y_t-2_ + … + φ_p_Y_t–p_ + e_t_ – θ_1_e_t-1_* − *…* − *θ_q_e_t-q_*, where *φ_1_Y_t-1_ + φ_2_Y_t-2_ + … + φ_p_Y_t–p_ + e_t_* represents the AR model part, *e_t_ – θ_1_e_t-1_* − *…* − *θ_q_e_t-q_* represents the MA model part. *Y_t-1_* is the observation at the (t-p) period, while p and q denote the orders of the AR and MA models, respectively, and *e_t_* is the random error at time *t* (14). In the ARIMA model, the time series should be a stationary random sequence with zero mean.

### Age-period-cohort model

The age-period-cohort (APC) model framework is a statistical technique used to analyze and decompose overall trends into three components: age effects, which reflect changes in risk over the course of the human lifespan; period effects, which represent the impact on all age groups during a specific time period; and cohort effects, which capture risk differences between birth cohorts (15). It is expressed using a log-linear regression model: log(Y_i_) = μ + α*age_i_ + β*period_i_ + γ*cohort_i_ + ε, where *Y_i_* represents the incidence rate, mortality rate, or DALYs rate for leukemia, and *α*, *β* and *γ*are the coefficients for age, period, and cohort, respectively. *μ* is the intercept, and *ε* is the model residual. In the APC framework, the intrinsic estimator (IE) method is employed to address the identification challenges posed by the linear dependencies between age, period, and cohort effects. This method effectively resolves the problem of model parameter non-identifiability, ensuring the reliability of the estimates. Detailed methodological explanations can be found in earlier publications (16).

### Cross-country inequalities analysis

The slope index of inequality and the concentration index were used to quantify the level of inequality in the burden of AVH across different countries. These are standardized indicators recommended by the World Health Organization to measure absolute and relative gradients of inequality (17). The slope index of inequality is obtained through regression analysis, using age-standardized incidence rates (ASIR), age-standardized mortality rates (ASMR), or DALYs rates at the national and regional levels as the dependent variable. The midpoint of population cumulative categories, sorted by SDI, is used to determine the relative social position scale (18), which serves as the independent variable. Heteroscedasticity is considered, and a weighted regression model is used for testing. The health inequality concentration index is calculated using the Lorenz concentration curve to represent the cumulative relative distribution of the population. It reflects the relationship between the cumulative proportion of ASIR, ASMR, or DALYs rates and the cumulative population distribution sorted by SDI (19).

All the above statistics were performed using R 4.3.0, and *p* < 0.05 was considered statistically significant.

## Results

### Global burden of acute viral hepatitis

**Figure 1:**
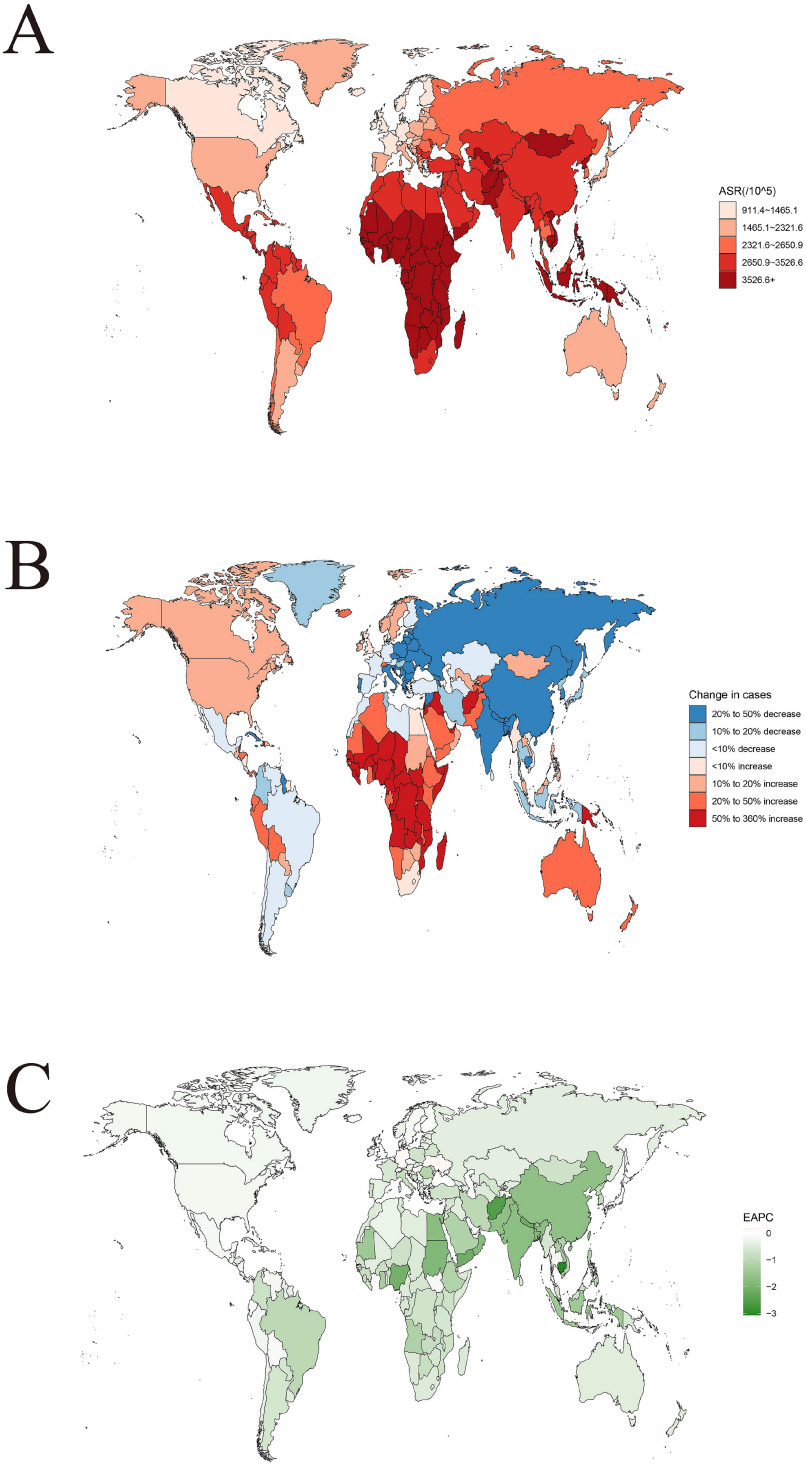
The global disease burden of acute viral hepatitis for both sexes in 204 countries and territories. **(A)** The ASIR of acute viral hepatitis in 2021. **(B)** The relative change in incident cases of acute viral hepatitis between 1990 and 2021. **(C)** The EAPC of acute viral hepatitis ASIR from 1990 to 2021. ASIR, age standardized incidence rate; EAPC, estimated annual percentage change.

Globally, the age-standardized incidence rate (ASIR), age-standardized mortality rate (ASMR), and DALYs rate of acute viral hepatitis (AVH) vary significantly across different regions (Figure 1; Supplementary Figures S1, S2). In 2021, the Federal Republic of Somalia had the highest ASIR (6768.8 per 100,000), followed by the Republic of Zimbabwe (5871.7 per 100,000) and the Republic of Chad (5756.9 per 100,000). In absolute numbers, the Republic of India had the highest number of acute viral hepatitis cases in 2021 (45,503,271.2), followed by the People’s Republic of China (39,808,670.7) and the Federal Republic of Nigeria (14,577,268) (Supplementary Table S1). In contrast, the Federated States of Micronesia had the highest ASMR (17.9 per 100,000), followed by the Islamic Republic of Iran (10.8 per 100,000) and the Federal Democratic Republic of Nepal (4.6 per 100,000). In absolute numbers, the Republic of Estonia had the highest number of acute viral hepatitis deaths in 2021 (27,993.3), followed by the State of Qatar (6,906.1) and the Republic of Finland (5,011.8) (Supplementary Table S2). Unlike the ASMR results, the Federal Republic of Somalia had the highest DALYs rate (664.4 per 100,000), followed by the Islamic Republic of Afghanistan (375.4 per 100,000) and the Republic of Chad (178.9 per 100,000). In absolute terms, the Republic of India had the highest DALYs (1,891,678.2 years), followed by the Islamic Republic of Pakistan (367,462.3 years) and the Federal Republic of Nigeria (212,870.1 years) (Supplementary Table S3).

**Figure 2:**
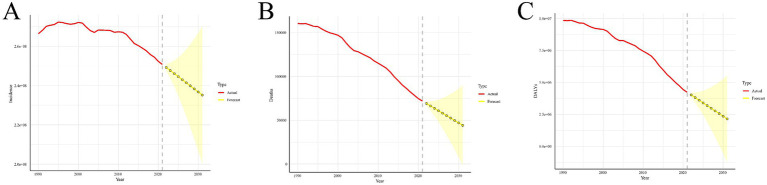
Predicted trends of acute viral hepatitis incident cases **(A)**, deaths **(B)**, and DALYs **(C)** in the next decade (2021–2031). Red lines represent the true trend during 1990–2021; yellow dot lines and shaded regions represent the predicted trend and its 95% CI.

The results from the ARIMA model show that the incidence of acute viral hepatitis increased from 1990 to 1995, stabilized over the next 5 years, and then experienced a significant decline starting in 2011. It is projected that the number of cases will decrease from 250,774,458.4 in 2021 to 235,195,964.4 in 2031. The predicted number of acute viral hepatitis deaths will also continue to decrease in the future, from 71,846.39 in 2021 to 44,157.67 in 2031. In contrast, the DALYs for acute viral hepatitis show a similar long-term downward trend as the mortality, with a predicted decrease from 10,982,836.2 in 2021 to 10,785,356.1 in 2031 (Figure 2; Supplementary Table S4).

Decomposition analysis of age-standardized incidence number, age-standardized death number, and age-standardized DALYs number.

**Figure 3:**
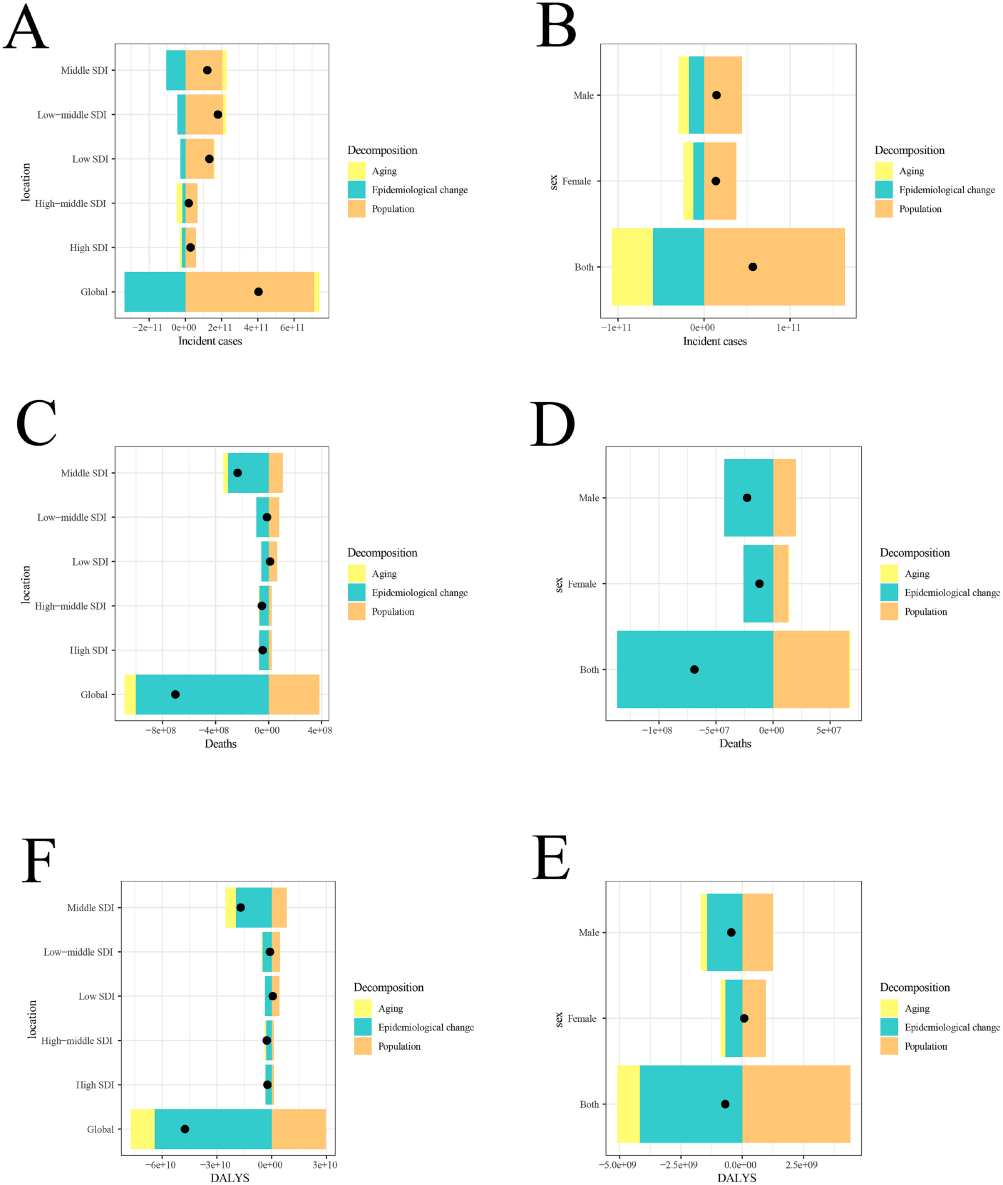
Changes in acute viral hepatitis incidence **(A,B)**, mortality **(C,D)** and DALYs **(E,F)** from 1990 to 2021 according to population-level determinants of population growth, aging, and epidemiological change across different socio-demographic index quintiles and by sex. The black dot represents the overall value of change contributed by all three components. SDI, socio-demographic index; DALYs, disability-adjusted life years.

From 1990 to 2021, the global incidence of acute viral hepatitis was most strongly influenced by population growth (176.07%), followed by aging (7.25%). The increase in the incidence of acute viral hepatitis was greatest in low-middle SDI quintile regions, primarily driven by population growth (115.74%) (Figure 3A; Supplementary Table S5). Similarly, when analyzing by sex, population growth contributed the most (288.33%) (Figure 3B; Supplementary Table S5).

The decline in global acute viral hepatitis mortality was most influenced by epidemiological changes (142.36%), followed by aging (12.19%). The greatest decline in mortality occurred in middle SDI quintile regions, where epidemiological changes contributed the most (130.8%), followed by aging (15.3%) (Figure 3C; Supplementary Table S5). A similar pattern was observed in the sex stratification, where the reduction in acute viral hepatitis mortality was most affected by epidemiological changes (197.96%) (Figure 3D; Supplementary Table S5).

Notably, the global decline in DALYs due to acute viral hepatitis was primarily driven by epidemiological changes (134.88%), followed by aging (27.51%), while the increase in DALYs was primarily driven by population growth (−62.39%). The greatest decline in DALYs occurred in middle SDI quintile regions, where epidemiological changes contributed the most (115.31%) (Figure 3E; Supplementary Table S5). In the sex stratification, the increase in DALYs was solely influenced by population growth (−634.6%) (Figure 3F; Supplementary Table S5).

Additionally, it was observed that only low-SDI regions experienced increases in incidence, mortality, and DALYs due to acute viral hepatitis (Figure 3; Supplementary Table S5).

### The influential factor for EAPC

**Figure 4:**
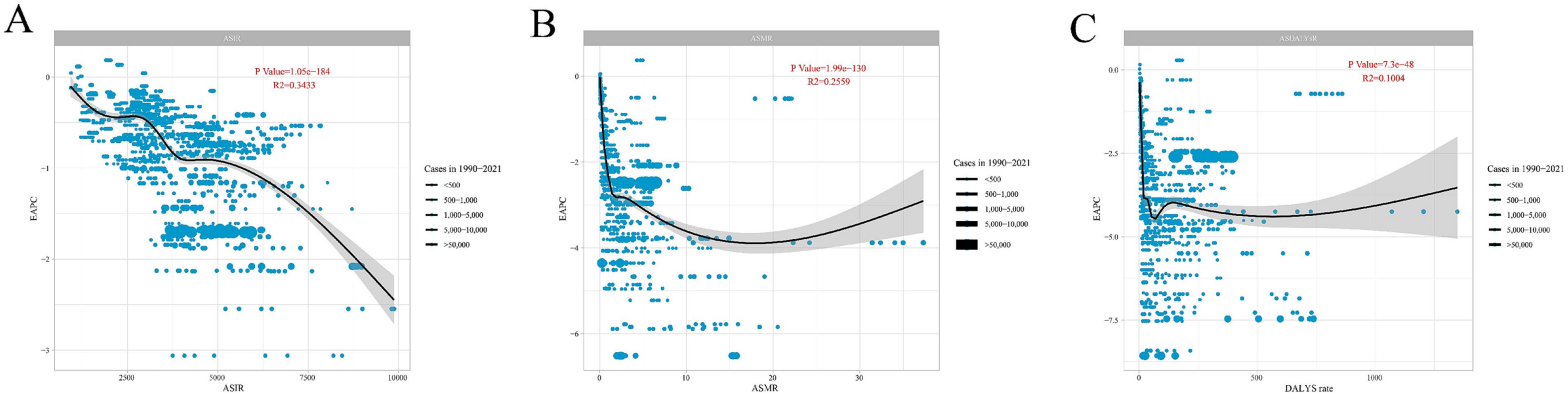
The correlation between EAPC and **(A)** ASIR, **(B)** ASMR, and **(C)** DALYs rate for acute viral hepatitis incidence. Circles represent acute viral hepatitis incidence cases from 1990 to 2021, with larger circles indicating higher cases. The R^2^ and *p* values were derived from Pearson correlation analysis. EAPC, estimated annual percentage change; ASIR, age-standardized incidence rate; ASMR, age-standardized mortality rate; DALYs, disability-adjusted life years.

There is a significant association between EAPC and ASIR, ASMR, and DALYs rate (*p* < 0.05). When ASIR is below 5,000, EAPC fluctuates, alternating between declines and stabilization. As ASIR exceeds 5,000, EAPC shows a steady decline (*p* = 1.05E-184, *R*^2^ = 0.3433). When ASMR is less than 5, EAPC experiences a sharp drop, and when ASMR is above 20, EAPC increases (*p* = 1.99E-130, *R*^2^ = 0.2559). A similar pattern is observed with ASMR results: when DALYs rate is below 100, EAPC shows a sharp decline, followed by small fluctuations and then a stable decline. When DALYs rate exceeds around 750, EAPC slightly increases (*p* = 7.3E-48, *R*^2^ = 0.1004) (Figure 4).

### Age, period and cohort effects on acute viral hepatitis incidence, mortality and DALYs

**Figure 5:**
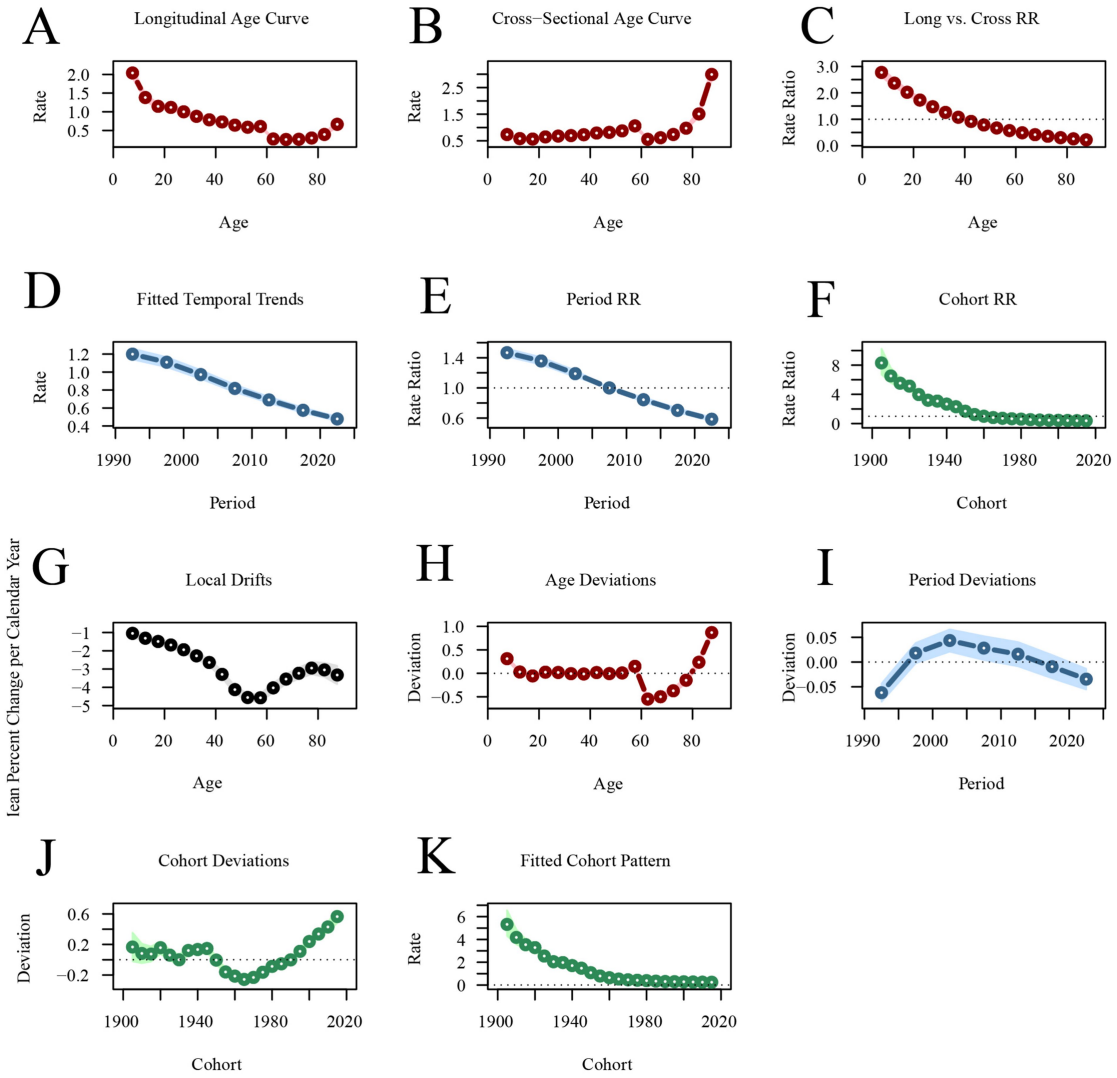
Age-period-cohort analysis of global acute viral hepatitis incidence from 1990 to 2021. **(A,B)** Longitudinal and cross-sectional age curves of acute viral hepatitis incidence. **(C)** Comparison of longitudinal vs. cross-sectional rate ratios. **(D,E)** Fitted temporal trends and period rate ratios. **(F)** Cohort rate ratios. **(G,H)** Local drifts and age deviations. **(I,J)** Period deviations and cohort deviations. **(K)** Fitted cohort pattern.

Figure 5 illustrates the age-period-cohort effects on the incidence of acute viral hepatitis. The longitudinal age curve shows that the incidence of acute viral hepatitis is higher in individuals under 20 years old [RR_age(7.5)_ = 2.0390, 95%CI: 1.9114–2.1751], and as age increases, the incidence gradually decreases [RR_age(67.5)_ = 0.2576, 95%CI: 0.2377–0.2792]. In individuals aged over 70, the incidence begins to increase [RR_age(87.5)_ = 0.6647, 95%CI:0.5963–0.7409]. The incidence of acute viral hepatitis has been decreasing year by year across different periods, with RR_period(1992.5)_ = 1.4669(95%CI: 1.4159–1.5199) decreasing to RR_period(2022.5)_ = 0.5852(95%CI: 0.5618–0.6096). Similarly, the incidence of acute viral hepatitis is higher in earlier birth cohorts [RR_cohort(1905)_ = 8.2982, 95%CI: 6.7167–10.2522], and lower in more recent birth cohorts [RR_cohort(2015)_ = 0.3845, 95%CI: 0.3486–0.4241] (Figure 5; Supplementary File 1).

**Figure 6:**
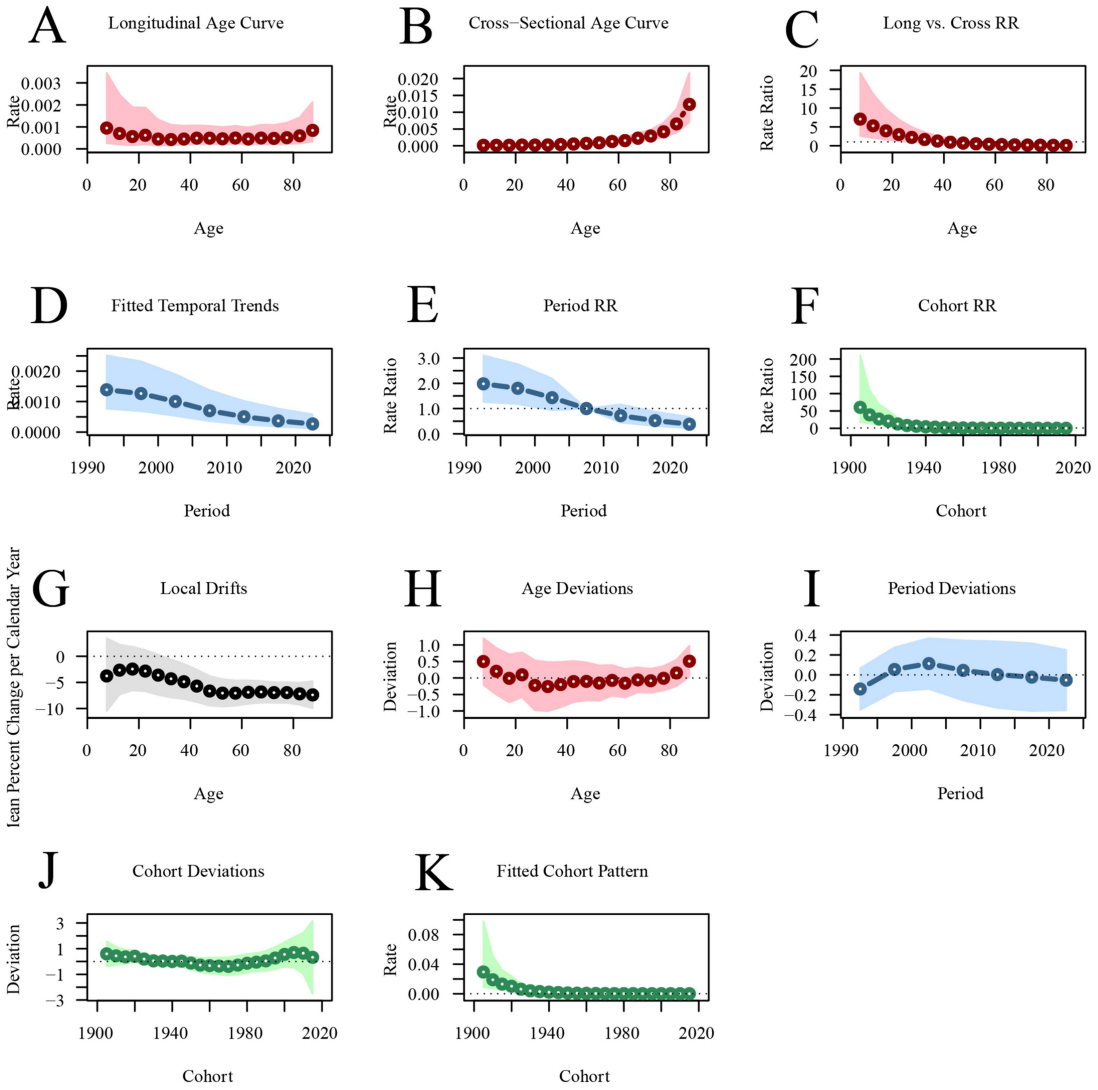
Age-period-cohort analysis of global acute viral hepatitis mortality from 1990 to 2021. **(A,B)** Longitudinal and cross-sectional age curves of acute viral hepatitis incidence. **(C)** Comparison of longitudinal vs. cross-sectional rate ratios. **(D,E)** Fitted temporal trends and period rate ratios. **(F)** Cohort rate ratios. **(G,H)** Local drifts and age deviations. **(I,J)** Period deviations and cohort deviations. **(K)** Fitted cohort pattern.

In contrast, the longitudinal age curve shows that the mortality rate for acute viral hepatitis is low across all age groups. The mortality rate for acute viral hepatitis has been decreasing year by year across different periods, with RR’_period(1992.5)_ = 1.9792 (95%CI: 1.2627–3.1022) decreasing to RR’_period(2022.5)_ = 0.38 (95%CI: 0.2174–0.6641). The mortality rate for acute viral hepatitis is higher in earlier birth cohorts [RR’_cohort(1905)_ = 60.488, 95%CI: 17.4086–210.1713], and lower in more recent birth cohorts [RR’_cohort(2015)_ = 0.0785, 95%CI: 0.0034–1.7966] (Figure 6; Supplementary File 2).

**Figure 7:**
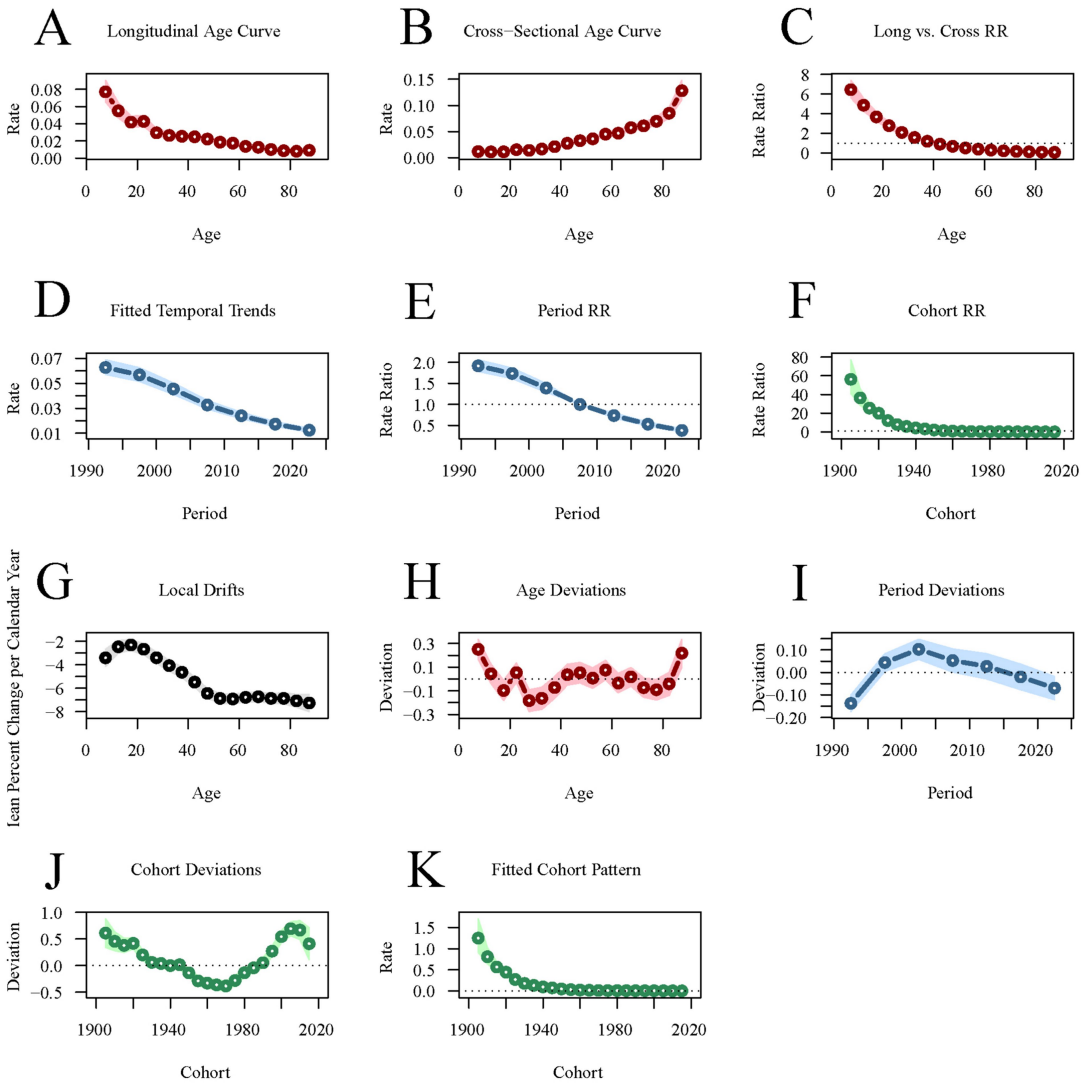
Age-period-cohort analysis of global acute viral hepatitis DALYs from 1990 to 2021. **(A,B)** Longitudinal and cross-sectional age curves of acute viral hepatitis incidence. **(C)** Comparison of longitudinal vs. cross-sectional rate ratios. **(D,E)** Fitted temporal trends and period rate ratios. **(F)** Cohort rate ratios. **(G,H)** Local drifts and age deviations. **(I,J)** Period deviations and cohort deviations. **(K)** Fitted cohort pattern.

Meanwhile, the longitudinal age curve shows that the rate of DALYs for acute viral hepatitis gradually decreases with age, with the RR value decreasing from 0.077 at 7.5 years old to 0.0092 at 87.5 years old. The rate of DALYs for acute viral hepatitis has been decreasing year by year across different periods, with RR``_period(1992.5)_ = 1.9184 (95%CI: 1.7861–2.0604) decreasing to RR``_period(2022.5)_ = 0.3811 (95%CI:0.3468–0.4188). The rate of DALYs for acute viral hepatitis is higher in earlier birth cohorts [RR``_cohort(1905)_ = 56.1426, 95%CI: 40.9994–76.879], and lower in more recent birth cohorts [RR``_cohort(2015)_ = 0.0958, 95%CI: 0.0688–0.1333] (Figure 7; Supplementary File 3).

The distribution changes of acute viral hepatitis subtypes across regions and periods.

**Figure 8:**
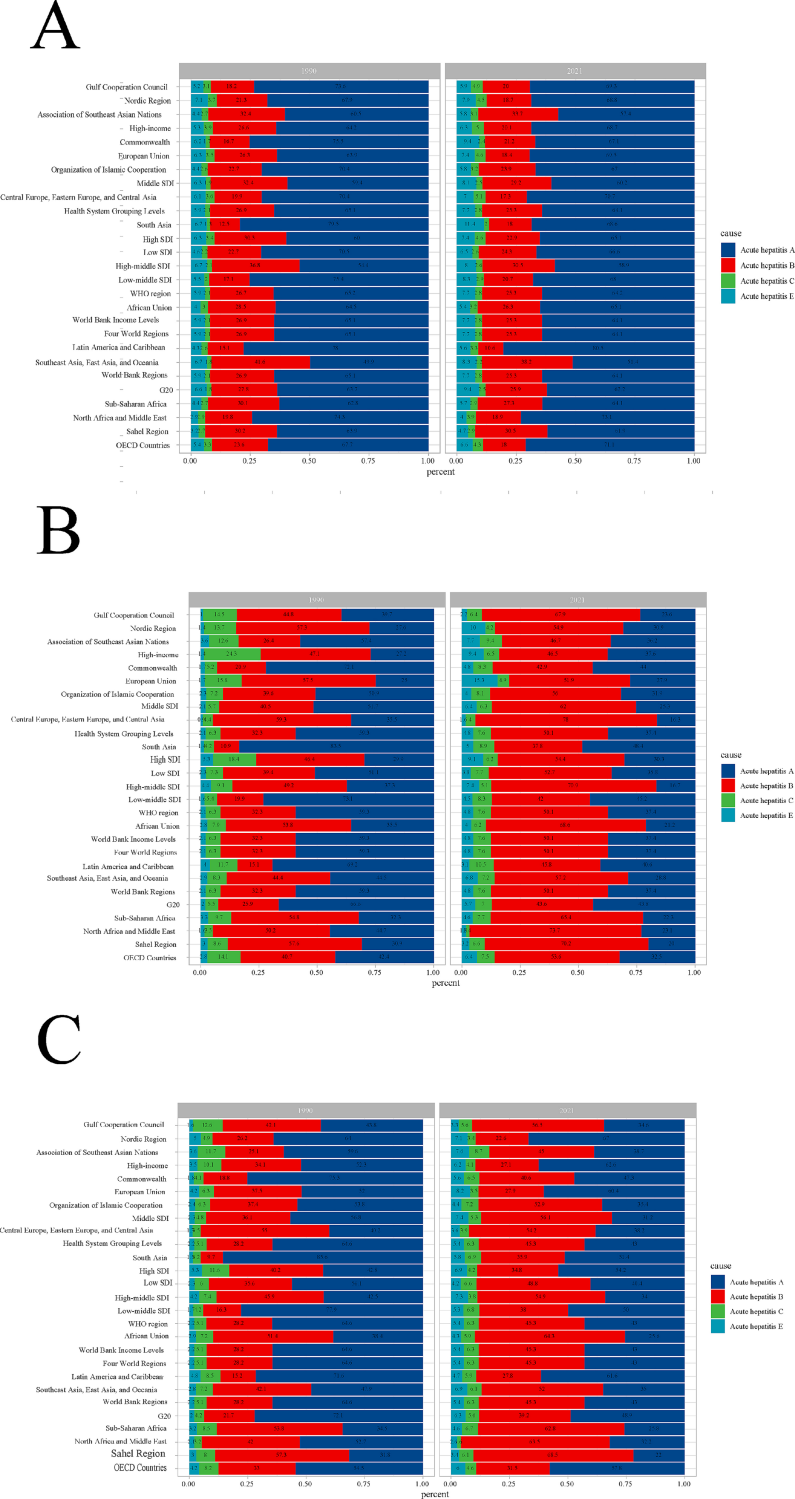
Contribution of acute viral hepatitis subtypes incident cases **(A)**, deaths **(B)**, and DALYs **(C)**, both sexes and by region, in 1990 and 2021.

The burden of different types of acute viral hepatitis worldwide in 1990 and 2021 is shown below. In all regions, compared to 1990, the incidence of AHC and AHE has increased in 2021. Notably, AHA continues to dominate the incidence in all regions (Figure 8A). In contrast, compared to 1990, the mortality share of AHE has increased in all regions. It is worth noting that overall, the mortality share of AHB has increased and gradually surpassed that of AHA, becoming the dominant cause of death (Figure 8B). Similar results were observed for DALYs due to acute viral hepatitis: compared to 1990, the DALY share of AHE has increased in all regions. Overall, the DALY share of AHB has increased, while the DALY share of AHA has decreased (Figure 8C).

**Figure 9:**
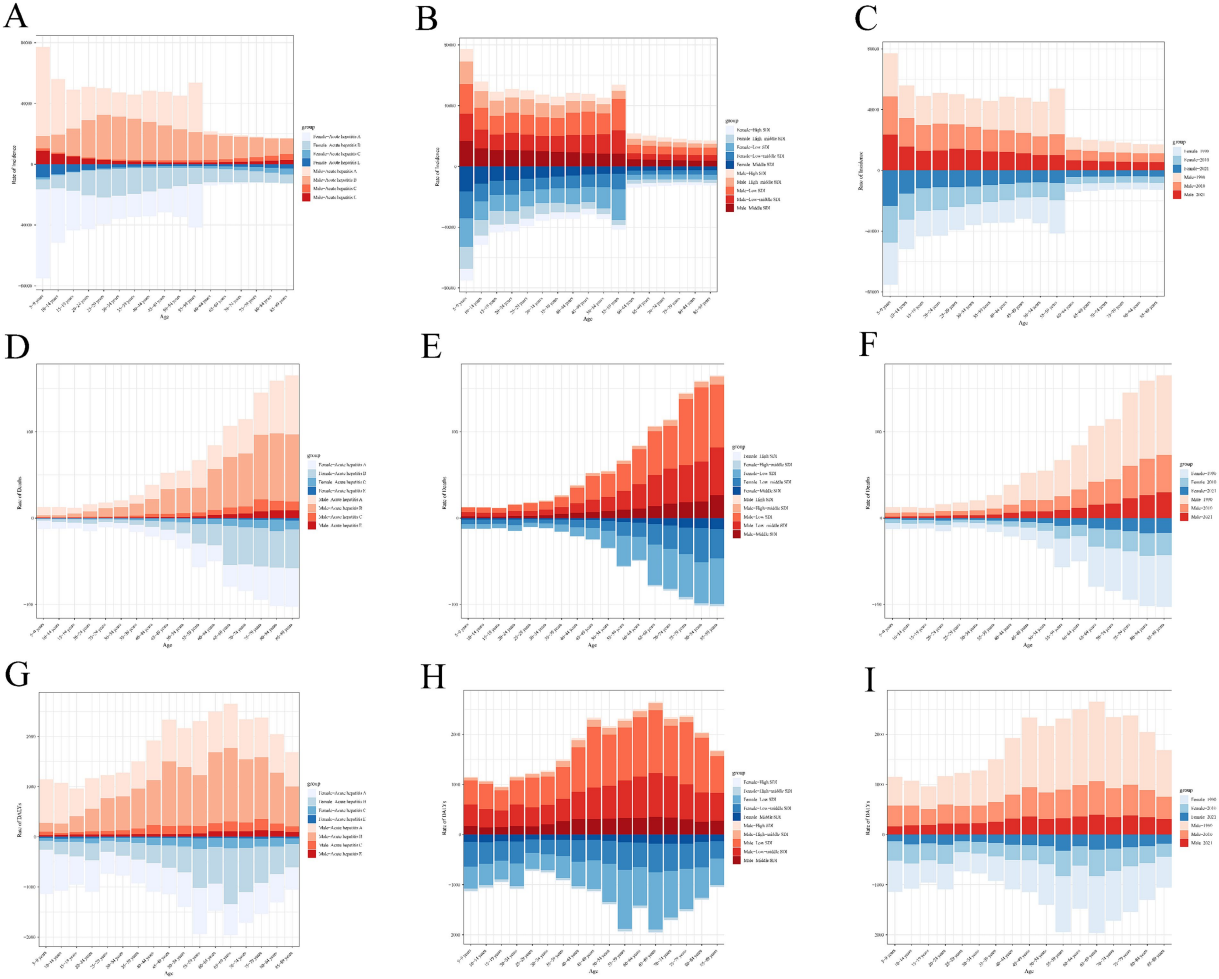
Age-specific incidence **(A–C)**, mortality **(D–F)**, and DALYs **(G–I)** of acute viral hepatitis stratified by cause, sex, SDI levels, and periods.

After stratifying by specific age groups and sex, it was observed that individuals under 25 years of age primarily experienced AHA, while older groups had higher incidences of AHB, and this distribution was not affected by sex. The incidence of acute viral hepatitis gradually decreases with age, and in every age group, the incidence among males is higher than that among females (Figure 9A). Overall, the incidence of acute viral hepatitis across all age groups is predominantly concentrated in lower SDI regions (Figure 9B).

Figure 9C shows that regardless of the year (1990, 2010, or 2021), the incidence of acute viral hepatitis is consistently higher in males than in females across all age groups. In contrast to the incidence results, individuals under 25 years old primarily died from AHA, while older groups experienced mortality from both AHA and AHB. Mortality increased rapidly with age, and in every age group, the mortality rate for males was higher than that for females (Figure 9D). Overall, mortality from acute viral hepatitis across all age groups was concentrated in lower SDI regions, with males having a higher mortality rate than females (Figure 9E).

Regardless of the year (1990, 2010, or 2021), the mortality rate of acute viral hepatitis was consistently higher in males than in females across all age groups (Figure 9F). Figure 9G shows that for individuals under 25 years old, the DALYs burden of acute viral hepatitis was primarily due to AHA, while for older individuals, AHB had a greater impact. Similarly, the DALYs burden of acute viral hepatitis was heavier in lower SDI regions (Figure 9H), and in all periods and age groups, the DALYs burden was greater for males than for females (Figure 9I).

Global health inequality analysis of incidence, mortality and DALYs in acute viral hepatitis from 1990 to 2021.

**Figure 10:**
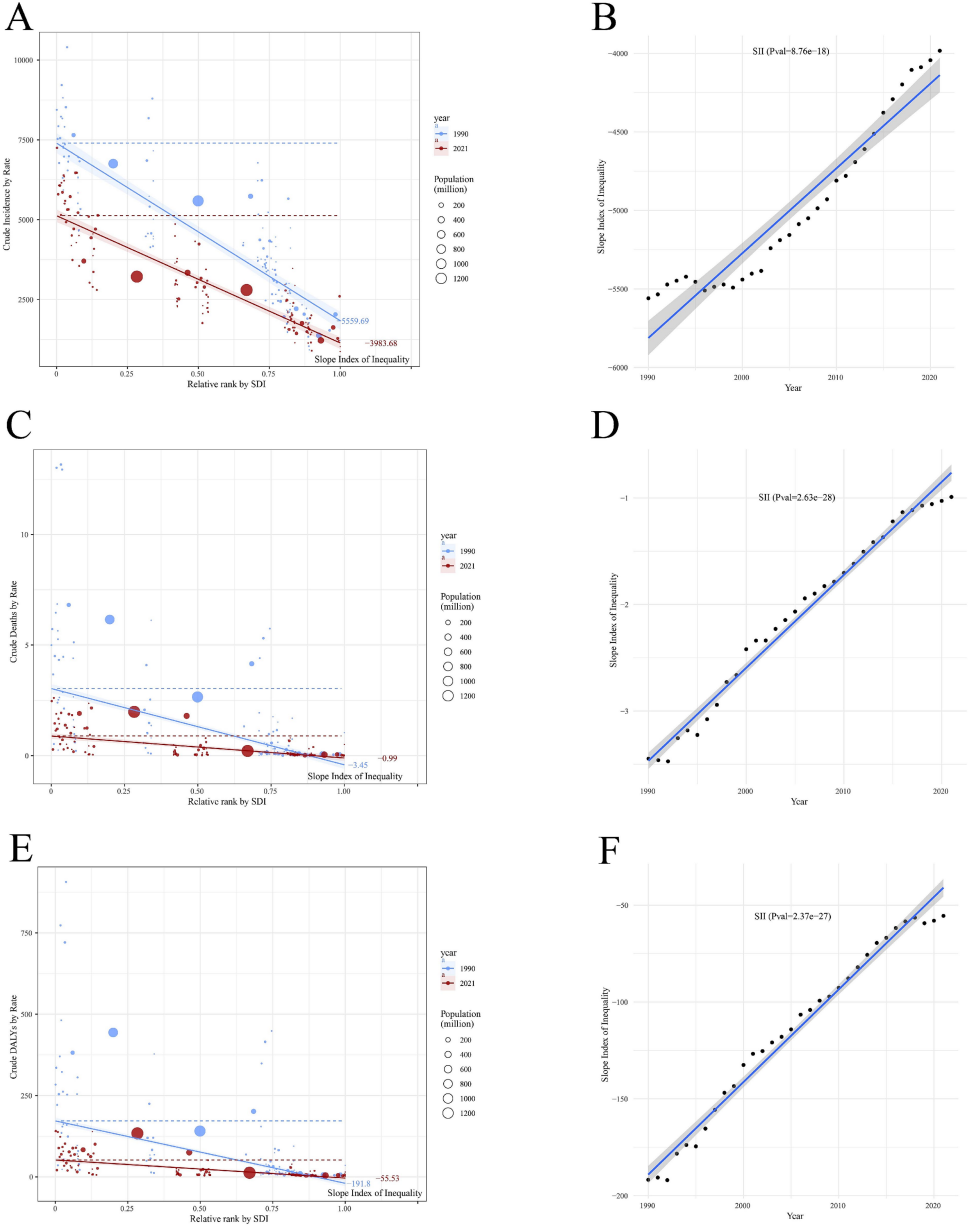
Trends in crude incidence **(A,B)**, mortality **(C,D)**, and DALYs **(E,F)** rates of acute viral hepatitis by SDI and SII from 1990 to 2021. Different circles represent different countries and territories, and the circle size represents population. SII, slope index of inequality; SDI, socio-demographic index.

Compared to 1990, the level of inequality in the incidence, mortality, and DALYs rate of acute viral hepatitis across countries and regions at different SDI levels decreased in 2021. Overall, as SDI levels increased, the incidence showed a declining trend, and the absolute value of the Slope Index of Inequality (SII) for 2021 was significantly lower than in 1990, with values of −3983.68 and −5559.69, respectively (Figure 10A; Supplementary Table S6). Moreover, the SII exhibited an increasing trend, and the regression fitting results showed highly significant statistical significance (*p* = 8.76e−18), indicating that over the past few decades, the inequality between countries and regions with different levels of social development has been steadily decreasing, and this trend is very stable (Figure 10B).

Similarly, the SII for the mortality rate of acute viral hepatitis in 2021 was higher than in 1990, with values of −0.99 and −3.45, respectively (Figure 10C; Supplementary Table S6). The increasing trend in the regression fitting results also showed highly significant statistical significance (*p* = 2.63e−28), suggesting that the inequality in the mortality rate of acute viral hepatitis between countries and regions with different levels of social development has steadily decreased over the past few decades (Figure 10D).

Likewise, the inequality in the DALYs of acute viral hepatitis has decreased, with the SII value rising from −191.8 in 1990 to −55.53 in 2021 (Figure 10E; Supplementary Table S6). Overall, the SII showed an increasing trend, indicating that the inequality in the DALYs burden of acute viral hepatitis between countries and regions with different levels of social development is narrowing (Figure 10F).

**Figure 11:**
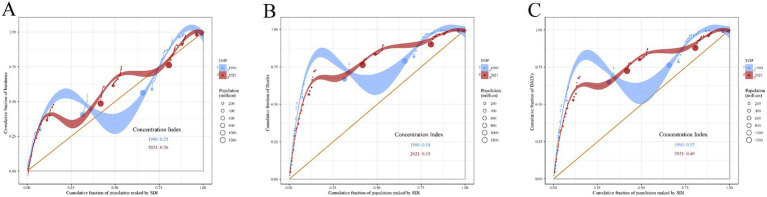
Concentration curves of acute viral hepatitis incidence **(A)**, mortality **(B)** and DALYs **(C)** by SDI in 1990 and 2021. The orange diagonal line represents perfect equality, where acute viral hepatitis incidence, mortality and DALYs would be equally distributed across all SDI levels. Shaded areas represent the 95% confidence intervals of CI. SDI, socio-demographic index; CI, concentration index.

In contrast, as shown in Figure 11A, the cumulative curve of the incidence burden of acute viral hepatitis is closer to the equality line (orange diagonal line), with the concentration index (CI) for 1990 being 0.25 and for 2021 being 0.26. This indicates that the inequality in the incidence of acute viral hepatitis based on socio-economic status has remained relatively stable (Figure 11A). Similarly, the CI for the mortality rate of acute viral hepatitis was 0.54 in 1990 and 0.53 in 2021 (Figure 11B). The cumulative curves for the DALYs burden of acute viral hepatitis in both 1990 and 2021 deviate from the equality line, with a CI of 0.52 in 1990 and 0.49 in 2021 (Figure 11C). These results suggest that from 1990 to 2021, the burden of acute viral hepatitis has been concentrated in low-SDI regions, and this inequality has persisted over the past few decades.

## Discussion

This study provides a comprehensive analysis of the time trends in the incidence, mortality, and DALYs of four types of acute viral hepatitis (AVH) at the global, national, and regional levels from 1990 to 2021. Overall, from 1990 to 2021, the global burden of AVH showed a declining trend, particularly in high-SDI regions. This change may be attributed to the widespread adoption of vaccination, improvements in sanitation, and strengthened public health interventions (5, 20). However, the burden of AVH remains high in low-SDI regions (e.g., the Federal Republic of Somalia and the Republic of Chad), with significantly higher ASIR and DALYs rates compared to other regions, highlighting the ongoing challenge of global health inequality.

The burden of AVH incidence and mortality in 1990 and 2021 was primarily concentrated in low-SDI regions (CI > 0.2). We believe that this unequal disease burden is closely related to the uneven distribution of medical resources and differences in education levels. Low-SDI regions often face limited medical facilities, inadequate diagnostic technologies, and a lack of treatment resources, which makes early diagnosis and timely treatment difficult, thereby increasing the incidence and mortality of AVH. At the same time, residents in these areas typically lack sufficient health education and preventive knowledge, limiting their participation in early prevention and vaccination for hepatitis B. However, the degree of health inequality between countries and regions with different levels of social development has been continuously decreasing, and the trend remains very stable. The inequality in the DALYs burden of AVH has also declined. Consistent with previous studies, AHA and AHB are the major types of AVH in terms of incidence and mortality (21), accounting for more than 70% of global cases. From 1990 to 2021, the proportion of AHA decreased, while the proportion of AHB increased, with the combined share of both stabilizing. However, the independent proportions of these two types were greatly influenced by regional differences. A similar distribution is observed for AVH mortality and DALYs. For instance, the incidence of AHA is highest in sub-Saharan Africa (with 80% of AHA cases occurring in children), which may be one reason why regions with high fertility rates also have higher AHA incidence. On the other hand, AHB incidence is increasing, and the ASIR and ASMR in high-SDI regions are typically higher than in low-SDI regions, consistent with prior research findings (6). This phenomenon is also related to differences in transmission routes and risk exposures across regions: AHA primarily spreads via the fecal-oral route, which is more prevalent in low-SDI regions with poorer sanitation conditions (22, 23), while AHB transmission is associated with blood or sexual contact, and medical procedures (e.g., blood transfusions, injections) and sexual behavior patterns in high-SDI regions may exacerbate its transmission risk (24). Additionally, it may be related to certain socio-economic factors, such as changes in lifestyle, high-risk groups for hepatitis B, and the impact of migration, all of which could contribute to the hepatitis B burden in high-SDI regions. The low prevalence of AHE in Asia contrasts with its high prevalence in Western countries, highlighting differences in regional pathogen distribution and health policies. This study also found that AHC incidence is the lowest among the four types of AVH, with the highest occurrence in Central Europe, Eastern Europe, and Central Asia. Meanwhile, the incidence and mortality rates of AVH are higher in populations under 20 years of age and over 80 years of age, but DALYs show a consistent decline with age. Male AVH incidence, mortality, and DALYs burden are significantly higher than in females, which is consistent with previous research findings (1, 2).

The results from the ARIMA model show that the number of AVH cases is expected to decrease from 250,774,458.4 in 2021 to 235,195,964.4 in 2031, with the number of AVH-related deaths decreasing from 71,846.39 in 2021 to 44,157.67 in 2031. The DALYs for AVH will continue to decline, from 10,982,836.2 in 2021 to 10,785,356.1 in 2031. One possible reason for this phenomenon is the global improvement in health conditions and the widespread adoption of vaccination (17). Meanwhile, the absolute value of the Slope Index of Inequality (SII) for AVH incidence and mortality has consistently and steadily decreased, indicating that the inequality in the burden of AVH between countries and regions with different levels of social development is shrinking. The decline in AVH cases and mortality will have a positive impact on global health strategies. This trend calls for public health policymakers in all regions, especially in low-SDI areas, to strengthen vaccination efforts, improve sanitation infrastructure, enhance health education levels, and promote community awareness of AVH prevention to reduce the disease burden. Furthermore, decomposition analysis indicates that from 1990 to 2021, the growth in AVH incidence across all regions globally was primarily driven by population growth, while the decline in mortality and DALYs was mainly influenced by epidemiological changes. We speculate that this may be related to population expansion and the accumulation of past infections (25), as well as improvements in health conditions and the widespread adoption of vaccination brought about by modernization processes (11, 19, 26). In particular, the widespread vaccination against hepatitis B has played a significant role in reducing AVH-related deaths and disease burden. According to estimates by the World Health Organization, in the past few decades, the promotion of the hepatitis B vaccine has led to a significant decline in global hepatitis B infection rates. The infection rate in children dropped from approximately 5% in the 1980s to below 1% by 2019. Strong support from the Global Alliance for Vaccines and Immunization (GAVI) has increased the global coverage of the hepatitis B vaccine from around 30% in 2000 to 85% in 2019, significantly reducing liver complications and mortality associated with hepatitis B (27).

Age-period-cohort analysis reveals that the incidence of AVH decreases and then increases with age, reaching its lowest point around 60 years old before continuing to rise. The incidence is higher in populations under 20 years of age and over 80 years of age. In contrast, AVH mortality rates are lower across all age groups, though they are slightly higher in individuals under 20 and over 80. Some studies suggest that age is a risk factor for AVH, and the incidence and prognosis of AVH vary at different age stages. The increased incidence in older individuals may be attributed to immune system decline (13, 14, 28). Additionally, differences in sanitation and the immature immune system in children may contribute to AVH incidence in this group (3, 15, 16). The period effect refers to the impact of medical conditions, diagnostic technologies, and changes in economic and cultural factors during specific periods on the burden of AVH. The cohort effect emphasizes the risks associated with socioeconomic, behavioral, and environmental exposures during early life and the different birth cohorts. The results of this study show a decrease in the period effect on AVH incidence, mortality, and DALYs, reflecting the effectiveness of global health policies (e.g., hepatitis B vaccination promotion). At the same time, early birth cohorts exhibit higher risks for AVH incidence, mortality, and DALYs compared to more recent cohorts, suggesting generational improvements in public health interventions and health awareness.

Notably, in all age groups across different SDI regions and years, male populations have a higher burden of AVH than females. This may be linked to higher-risk behaviors in males (such as alcohol abuse and high-risk sexual behavior) and differences in the utilization of medical resources, consistent with findings from several previous studies (1, 21, 29–31). In response to this phenomenon, it is recommended to strengthen health education for high-risk male populations in high-SDI regions, optimize early screening strategies, and promote regular health check-ups for high-risk groups. At the same time, attention should be given to the health management of immigrant populations and socially marginalized groups in high-SDI areas, ensuring they have equal access to medical resources and services. Furthermore, it is advised to improve the rational allocation of medical resources in high-SDI regions to enhance the efficiency of healthcare systems and ensure that a broader population benefits.

Health inequality analysis shows that the global burden of acute viral hepatitis (AVH) exhibits significant socioeconomic gradient disparities, with low-SDI countries facing a higher AVH burden. Although the Slope Index of Inequality (SII) for AVH incidence, mortality, and DALYs in 2021 was significantly higher than in 1990, indicating a reduction in health inequality between low-SDI and high-SDI regions, the concentration index reveals that the burden of acute hepatitis remains more pronounced in low-SDI regions. In 2021, low-SDI countries had a higher age-standardized incidence rate (ASIR), such as Somalia with 6,768.8 cases per 100,000 people, while high-SDI countries like Australia had a much lower rate of 1,456.7 cases per 100,000 people. This gap is likely closely related to differences in vaccination coverage and healthcare resources: some low-SDI regions have hepatitis B vaccination coverage rates below 50%, while most high-SDI countries generally exceed 90% (32). It is noteworthy that although the Slope Index of Inequality (SII) for global age-standardized incidence rate (ASIR) increased from −5,559.69 in 1990 to −3,983.68 in 2021, the concentration index (CI) for 1990 and 2021 was 0.25 and 0.26, respectively, indicating that inequality persists. This suggests that groups with lower socioeconomic status are still more likely to be exposed to risk factors (such as occupational exposure or unsafe injections). Regional differences in subtype distribution further exacerbate inequality. For instance, the proportion of deaths due to Hepatitis E (AHE) in sub-Saharan Africa increased from 12% in 1990 to 38% in 2021, which is directly related to the region’s weak healthcare infrastructure and high foodborne transmission risks (33, 34). In higher-SDI countries, despite the overall decline in incidence, the infection rate of Hepatitis C (AHC) among gay men is several times higher than that of the general population (35), highlighting the impact of social behavioral factors on health equity. Different types of occupational exposures have become new drivers of inequality. Hepatitis E virus (HEV), primarily transmitted through the fecal-oral route, can spread through contaminated water sources, undercooked meat (especially pork liver and pork), and cross-contamination of cooking utensils. Since pigs are a key natural host for HEV, workers in the related food industries face occupational exposure risks. Previous studies have shown that individuals working in veterinary medicine, meat inspection, slaughterhouses, and other similar fields have significantly higher HEV seropositivity rates than the general population (36). In the healthcare sector, since Hepatitis B Virus (HBV) is primarily transmitted through blood and bodily fluids, and Hepatitis C Virus (HCV) spreads mainly through blood, both can be transmitted through needlesticks, mucosal exposure, or contact with broken skin when exposed to patient infection sources, thereby increasing occupational exposure risks. Several studies have shown that healthcare workers have significantly higher HBV infection rates compared to the general population, and the occupational exposure risk to HCV is equally concerning (37, 38). Additionally, the GBD 2021 data on acute hepatitis does not include data related to HDV, which may lead to an underestimation of the actual burden of HBV and HDV co-infection, especially in regions with a high prevalence of HDV. Future research should consider integrating HDV data and exploring the co-infection of HBV and HDV to obtain a more comprehensive disease burden assessment.

Finally, the outbreak of the COVID-19 pandemic has had a profound impact on global public health systems, particularly in the diagnosis and treatment of chronic diseases and infectious diseases (39). Although our study primarily focuses on the epidemiological trends of AVH, the pandemic may have had potential interference with the accuracy of the data and the prediction results. For example, global lockdown measures and the redistribution of medical resources during the pandemic disrupted routine health services in many regions to varying degrees, which could have led to delays or missed diagnoses of AVH, thereby affecting the reporting of related data. Additionally, the COVID-19 pandemic may have caused some patients to delay or interrupt their treatments, which could also impact the mortality and disease burden estimates associated with AVH. While the occurrence of the COVID-19 pandemic is undoubtedly regrettable, as an uncontrollable factor, the disruption it caused to global health systems was inevitable. Nonetheless, based on previous GBD research data, we are still able to make certain judgments from historical trends, providing valuable insights for future AVH healthcare policy decisions.

In the future, to alleviate global health inequalities related to acute viral hepatitis (AVH), a multi-level intervention strategy must be implemented. The international community should prioritize supporting low-SDI countries in strengthening their healthcare system resilience, expanding vaccination coverage to the 90% target recommended by the WHO through technical assistance and financial support (32), and enhancing the rapid diagnostic capabilities of primary healthcare institutions. For example, promoting new low-cost HEV diagnostic tools (40, 41) can help overcome delays in diagnosis and treatment caused by resource shortages. At the same time, a cross-national data collaboration network should be established, drawing on the experiences of the global HIV monitoring system. Blockchain technology can be used to improve the timeliness and completeness of case reporting in low-resource areas, preventing data bias and reducing policy blind spots. For high-SDI countries, intervention models targeting high-risk populations should be optimized. An example is the Netherlands’ HCV elimination program for men who have sex with men (42), which combines digital health tools for targeted screening. For low-SDI regions, environmental governance and public health actions should be promoted in tandem, as seen in India’s “Clean Ganges Program,” which integrates water quality improvement with hepatitis prevention efforts (43). Only through the organic integration of global resource redistribution and localized strategies can the structural roots of health inequality be systematically addressed.

The limitations of this study include:

1. The GBD data relies on medical records reported by each country, and there may be underreporting or misreporting in low-SDI regions.
2. The ARIMA model assumes that future trends will follow historical patterns, which could underestimate the impact of sudden public health events (e.g., pandemics).
3. The age-period-cohort analysis carries a risk of ecological fallacy and requires further validation with individual-level data.

## Conclusion

While global AVH burden is decreasing, significant disparities persist, warranting tailored interventions to enhance resource equity in high-SDI regions and strengthen healthcare infrastructure in low-SDI areas.

## Data Availability

Publicly available datasets were analyzed in this study. This data can be found at: http://ghdx.healthdata.org/gbd-results-tool.
