# Supplementary material for: Global, regional, and national burden of acute hepatitis (1990–2021): a systematic analysis of the global burden of disease in 2021
Source: Front Med (Lausanne). 2025 Sep 10;12:1620371. doi: 10.3389/fmed.2025.1620371 (PMC12457326; doi:10.3389/fmed.2025.1620371)
Supplement: Supplementary file 1 [file Table_1.DOCX]

Table S1. The incident cases and age standardized incidence of Acute hepatitis caused by different reasons from 1990 to 2021.

| Cause | Location | 1990 | 2021 | 1990 | 2021 | 1990-2021 |
| --- | --- | --- | --- | --- | --- | --- |
|  |  | Incident cases  No. ×10^3^ (95% UI) | ASIR per 100,000  No. (95% UI) | Incident cases  No. ×10^3^ (95% UI) | ASIR per 100,000  No. (95% UI) | EAPC  No. (95% CI) |
| Acute hepatitis | American Samoa | 2162.9 (1988.5-2350.7) | 3764.3 (3427.2-4136.3) | 1478 (1351.1-1605) | 3113.3 (2850-3383.2) | -0.45 (-2.97-2.13) |
| Acute hepatitis | Antigua and Barbuda | 1889.3 (1788.9-1988.8) | 3004.9 (2838.6-3166) | 1908.2 (1804-2003.1) | 2689.8 (2532.9-2848) | -0.73 (-3.08-1.68) |
| Acute hepatitis | Arab Republic of Egypt | 3851410.7 (3334589.6-4429564.7) | 6397.6 (5204.9-7858.5) | 3990451.4 (3701498.2-4272134.8) | 3485.2 (3211.7-3755) | -1.35 (-3.54-0.88) |
| Acute hepatitis | Argentine Republic | 899858.7 (837982.7-964215) | 2648.8 (2470.2-2836) | 882107 (820935.3-935745) | 2253.1 (2085.3-2410.8) | -0.42 (-3.45-2.7) |
| Acute hepatitis | Australia | 320657.1 (290775.7-359974.5) | 2013.8 (1830.9-2242.4) | 407037.1 (367822.1-448653.8) | 1769.4 (1622.3-1930.9) | -0.51 (-2.65-1.68) |
| Acute hepatitis | Barbados | 6605.6 (6217.9-6963.5) | 2859.9 (2686.6-3017.2) | 5375.3 (5090.4-5666.6) | 2608.6 (2449.8-2766.5) | -0.57 (-2.97-1.89) |
| Acute hepatitis | Belize | 8226 (7722.7-8699) | 3201.9 (3003.2-3392.2) | 12660.9 (12053.1-13298.5) | 2941.8 (2789.3-3104.7) | -0.55 (-2.82-1.78) |
| Acute hepatitis | Bermuda | 1410.4 (1332.6-1486.4) | 2835.7 (2665.3-2987.6) | 1020.7 (961.6-1077.4) | 2605.9 (2442.6-2752.9) | -0.63 (-3.06-1.85) |
| Acute hepatitis | Bolivarian Republic of Venezuela | 702600.3 (663542.9-743294.8) | 2975.6 (2802.8-3151.1) | 651761.1 (615633.1-689087.4) | 2756 (2602-2915) | -0.51 (-3.05-2.1) |
| Acute hepatitis | Bosnia and Herzegovina | 111094.3 (103893.3-118524.9) | 2642.9 (2465.1-2827.3) | 53500.1 (49873-56884.5) | 2207.3 (2039.5-2354.4) | -0.57 (-3.15-2.07) |
| Acute hepatitis | Brunei Darussalam | 7023.3 (6236.3-7904.3) | 2491 (2226-2800.9) | 8023.2 (7316.7-8953.4) | 1884 (1712.1-2080.2) | -0.81 (-2.87-1.29) |
| Acute hepatitis | Burkina Faso | 755405.6 (684450-828891.9) | 6403.8 (5568.1-7375.7) | 1381831.3 (1282730.7-1495226.3) | 4976.2 (4467.6-5567.7) | -0.66 (-2.95-1.69) |
| Acute hepatitis | Canada | 418632.6 (379741-461455.3) | 1638.8 (1502.9-1801.6) | 480276.7 (438260.9-537113.8) | 1456.7 (1332.2-1601.5) | -0.24 (-2.51-2.09) |
| Acute hepatitis | Central African Republic | 223158.6 (201345.1-247352) | 6180.5 (5401.9-6992.5) | 337670.5 (304134.4-371426.1) | 5098.7 (4478.5-5754.4) | -0.46 (-2.59-1.73) |
| Acute hepatitis | Commonwealth of Dominica | 2548.8 (2407.4-2689) | 3081.9 (2910.1-3256.2) | 1482 (1401-1557.1) | 2790.1 (2611.8-2954.8) | -0.6 (-2.94-1.79) |
| Acute hepatitis | Commonwealth of the Bahamas | 8254.9 (7765.8-8677.7) | 2972.5 (2790-3135.3) | 8578.9 (8104.8-9036.4) | 2698.3 (2526.7-2855) | -0.6 (-2.92-1.79) |
| Acute hepatitis | Cook Islands | 696 (634-767.8) | 3343.6 (3004.7-3742.3) | 406.6 (371.5-440.9) | 2697.8 (2483.4-2904.4) | -0.78 (-3.18-1.68) |
| Acute hepatitis | Czech Republic | 192671.1 (177996.9-207887.5) | 2191.7 (2024-2363.3) | 152028.1 (142080.9-162320.7) | 1905.6 (1768.3-2046.1) | -0.45 (-2.68-1.82) |
| Acute hepatitis | Democratic People's Republic of Korea | 1118591.9 (957516.2-1318121.5) | 5215.7 (4438.2-6172.4) | 985767.3 (818698.5-1164735.9) | 4014 (3483.5-4577.7) | -0.67 (-3.12-1.86) |
| Acute hepatitis | Democratic Republic of Sao Tome and Principe | 8612 (7792.4-9433.2) | 5901.9 (5189.8-6693) | 10847.7 (9908.6-11949.7) | 4718.9 (4262.7-5225.9) | -0.69 (-3.1-1.79) |
| Acute hepatitis | Democratic Republic of the Congo | 3052855.1 (2746207.2-3389477.8) | 6188.2 (5311.9-7178.2) | 5802858.3 (5220815.7-6378607.6) | 5552 (4838.5-6284.7) | -0.35 (-2.52-1.87) |
| Acute hepatitis | Democratic Republic of Timor-Leste | 37910.6 (34542.3-41654.5) | 3691.8 (3304.6-4106.6) | 50935.9 (46238.2-55850.9) | 3153.4 (2845.5-3481.4) | -0.54 (-2.76-1.73) |
| Acute hepatitis | Democratic Socialist Republic of Sri Lanka | 514766.6 (474077.6-558167.3) | 2832 (2613.1-3074.2) | 479814.9 (443491.5-515590.7) | 2345.4 (2155.4-2513.8) | -0.61 (-2.68-1.5) |
| Acute hepatitis | Dominican Republic | 298228.3 (282247.2-315112) | 3339.3 (3155.7-3527.1) | 329079.5 (311920.5-346388.8) | 3062.3 (2898.8-3225.5) | -0.49 (-2.76-1.84) |
| Acute hepatitis | Eastern Republic of Uruguay | 74472.8 (70064.5-78767.7) | 2561.9 (2401.8-2716.2) | 60481.6 (56823.6-64027.4) | 2245.9 (2080.2-2406.5) | -0.48 (-3.3-2.42) |
| Acute hepatitis | Federal Democratic Republic of Ethiopia | 4050705.3 (3593732.3-4576260.5) | 6389.9 (5236.4-7768.2) | 5956769.1 (5434279.8-6488903.9) | 4496.9 (4041.8-4953.8) | -0.9 (-3.11-1.35) |
| Acute hepatitis | Federal Democratic Republic of Nepal | 1626204.9 (1447644.8-1817346) | 6397.5 (5406.5-7521.8) | 1037780.5 (974463.7-1097266.7) | 3271.5 (3079.7-3461) | -1.14 (-3.81-1.6) |
| Acute hepatitis | Federal Republic of Germany | 841500.6 (785142.9-895364.8) | 1198.9 (1109.7-1282) | 830968.9 (778746.8-885061.8) | 1143 (1046.1-1239.8) | -0.26 (-2.49-2.02) |
| Acute hepatitis | Federal Republic of Nigeria | 8836611.2 (7767507.3-10054054.9) | 9326.5 (7560.6-11596) | 14577268 (13417046-15930365.3) | 5332.9 (4785.6-5953.6) | -1.48 (-3.87-0.98) |
| Acute hepatitis | Federal Republic of Somalia | 760057.4 (674152.3-863890.5) | 7929.1 (6537.6-9550.7) | 1833184.6 (1656523.2-2009603.7) | 6768.8 (5854.1-7765.1) | -0.37 (-2.84-2.15) |
| Acute hepatitis | Federated States of Micronesia | 4360.9 (3939.3-4869.4) | 3718.8 (3305.3-4182.3) | 2791.3 (2550.7-3078.4) | 2709.2 (2475-3002.7) | -0.67 (-2.87-1.57) |
| Acute hepatitis | Federative Republic of Brazil | 5156735.3 (4847116.1-5463279.1) | 3112.7 (2911.6-3310.9) | 4732384.5 (4433121.9-5012365.3) | 2439.7 (2274.5-2584.7) | -1.04 (-3.34-1.31) |
| Acute hepatitis | French Republic | 854053 (794800-909398.9) | 1663.3 (1540.2-1778.6) | 795818.6 (742596.7-845348.9) | 1418.1 (1308.8-1522.7) | -0.51 (-3.01-2.05) |
| Acute hepatitis | Gabonese Republic | 59867.6 (54641.6-65202.5) | 4885.3 (4356.5-5475.7) | 81233.5 (74527.3-87861.6) | 4070.1 (3719-4434) | -0.6 (-2.52-1.36) |
| Acute hepatitis | Georgia | 165486.4 (156399.1-174985.1) | 3296.3 (3109.7-3485) | 86281.7 (81752.5-90673.8) | 3035.3 (2868-3186.2) | -0.46 (-2.44-1.56) |
| Acute hepatitis | Grand Duchy of Luxembourg | 4875.8 (4485.6-5295.5) | 1422 (1302.2-1546.3) | 7087.3 (6566.8-7633.9) | 1221.3 (1121.3-1313.9) | -0.68 (-2.91-1.6) |
| Acute hepatitis | Greenland | 1205.6 (1100.8-1323.2) | 2109 (1930.5-2304.5) | 987.9 (899.6-1083.5) | 1982.6 (1812.5-2165.5) | -0.28 (-2.74-2.25) |
| Acute hepatitis | Grenada | 3326.5 (3123.9-3518.2) | 3122.3 (2930.4-3309.9) | 2369.2 (2244.9-2500.1) | 2728.1 (2560.3-2888.4) | -0.81 (-3.1-1.53) |
| Acute hepatitis | Guam | 5628.6 (5162.8-6189.1) | 3825.2 (3514.1-4167.2) | 4675.7 (4314.8-5039.3) | 3183.1 (2956.1-3406.3) | -0.46 (-2.98-2.12) |
| Acute hepatitis | Hashemite Kingdom of Jordan | 183190.6 (168128.8-197725.7) | 3705.2 (3349.4-4104.8) | 374654.1 (351672.5-399002.9) | 3067.3 (2870.9-3278.4) | -0.82 (-3.26-1.67) |
| Acute hepatitis | Hellenic Republic | 191717.8 (176375.4-208599.2) | 2082.9 (1925.3-2252.9) | 148167.7 (135488.8-162853.1) | 1747.1 (1603.9-1897) | -0.45 (-3.13-2.3) |
| Acute hepatitis | Hungary | 181356.3 (170185-192071) | 2041.6 (1899.9-2174.4) | 146372.2 (137910.1-154535.4) | 2019.7 (1885.7-2143.9) | -0.19 (-2.35-2.02) |
| Acute hepatitis | Independent State of Papua New Guinea | 249431.8 (219243.5-282579.4) | 5122.8 (4399.8-5924.3) | 535794.1 (489413.4-589217) | 4372.5 (3909.9-4932.3) | -0.42 (-2.85-2.06) |
| Acute hepatitis | Independent State of Samoa | 7874.7 (7153-8789.3) | 3979.7 (3563.1-4562.4) | 8186.3 (7541.5-8952.5) | 3448.6 (3156.1-3815) | -0.26 (-2.65-2.2) |
| Acute hepatitis | Ireland | 54130.4 (49819.6-58711.3) | 1563.9 (1440.3-1695.8) | 59553.6 (55367.1-64348.3) | 1361.7 (1257.2-1478.2) | -0.43 (-2.8-2) |
| Acute hepatitis | Islamic Republic of Afghanistan | 1096056.1 (893162.9-1329036.9) | 10120.8 (7584.5-13197.3) | 1848343.9 (1696015-2013256.2) | 5096.4 (4440.8-5867.8) | -1.33 (-4.18-1.6) |
| Acute hepatitis | Islamic Republic of Iran | 2516967.9 (2373956.4-2658886.5) | 3520.2 (3296.5-3752.7) | 2126178.1 (2006353.1-2235217.8) | 2749.8 (2597.7-2898) | -1.14 (-3.65-1.44) |
| Acute hepatitis | Islamic Republic of Mauritania | 201286.5 (175015.3-229332.3) | 9385.7 (7504.4-11457.9) | 278829.8 (253200.3-307855.4) | 5692.6 (5027.8-6458.2) | -1.08 (-3.72-1.64) |
| Acute hepatitis | Islamic Republic of Pakistan | 8123887.4 (6905731.3-9750891.4) | 6190.8 (4755.7-8129) | 10154445.8 (9185068.9-11179387.5) | 3838.5 (3403.5-4292.4) | -0.99 (-3.17-1.25) |
| Acute hepatitis | Jamaica | 79890.8 (75167.7-84710.5) | 2921.1 (2744.5-3100.4) | 62586.5 (58989.4-66510.9) | 2650.2 (2481-2822.2) | -0.58 (-2.94-1.84) |
| Acute hepatitis | Japan | 2557493.8 (2349321.2-2782322.2) | 2156.3 (1996-2341.5) | 2077242.9 (1902837.6-2279527) | 1970.4 (1812.7-2132.2) | -0.62 (-2.67-1.47) |
| Acute hepatitis | Kingdom of Bahrain | 20320.7 (19003.1-21687.7) | 3658.7 (3432.8-3914.6) | 40941.5 (38213.5-43351.5) | 3096.7 (2918.7-3276.7) | -0.57 (-2.98-1.9) |
| Acute hepatitis | Kingdom of Belgium | 132919.1 (124740.3-141432.8) | 1539.5 (1438-1647.8) | 135969.9 (127388.4-144527.1) | 1421.9 (1326.8-1523.9) | -0.38 (-2.58-1.87) |
| Acute hepatitis | Kingdom of Bhutan | 37262.2 (32708.8-42802.8) | 4938.1 (4081.4-5986.4) | 21969.3 (20239.4-23623.7) | 3172.7 (2953.8-3378.6) | -0.7 (-3.16-1.82) |
| Acute hepatitis | Kingdom of Cambodia | 922352.6 (803804.2-1038076.1) | 8765.9 (7117.7-10711.3) | 660220.6 (592663.8-733411.3) | 3750.7 (3374-4178.6) | -1.93 (-4.29-0.49) |
| Acute hepatitis | Kingdom of Denmark | 53496.5 (49333.7-58093.5) | 1136.8 (1048.1-1231.1) | 58760 (54269.8-63276.3) | 1121.2 (1032.9-1212.6) | -0.05 (-2.19-2.13) |
| Acute hepatitis | Kingdom of Eswatini | 54446.5 (49645.8-59734.9) | 5052.5 (4481.4-5746.4) | 50044.6 (46138.9-53886.1) | 3876.4 (3549.6-4220.1) | -0.63 (-3.01-1.8) |
| Acute hepatitis | Kingdom of Lesotho | 97297.1 (88726.1-106529.4) | 5049.6 (4460.9-5681.5) | 84216.9 (76168.2-92298.2) | 4230.2 (3816-4664.5) | -0.38 (-2.78-2.08) |
| Acute hepatitis | Kingdom of Morocco | 1083572.4 (1005385.5-1159001.3) | 3565.1 (3285-3855.5) | 1072654.5 (1012991-1133780.3) | 3040.9 (2864-3220.5) | -0.53 (-2.93-1.92) |
| Acute hepatitis | Kingdom of Norway | 52331.2 (48905-56164.3) | 1382.3 (1283.9-1488.6) | 58538.9 (54657.2-62468) | 1251.1 (1155.4-1347.7) | -0.57 (-3.02-1.95) |
| Acute hepatitis | Kingdom of Saudi Arabia | 783669.9 (723740-844157.8) | 4391.8 (3877.6-4922.7) | 961701.8 (903799.1-1023415.7) | 2835.1 (2653.2-3002.8) | -1.12 (-3.09-0.89) |
| Acute hepatitis | Kingdom of Spain | 694422.5 (627275.4-765736.5) | 1933.1 (1764.6-2106.9) | 636064.9 (579500.6-703562.6) | 1571.8 (1442.2-1714.3) | -0.54 (-2.94-1.93) |
| Acute hepatitis | Kingdom of Sweden | 79068.7 (73445.4-85200.7) | 1009.9 (924.1-1105.2) | 87203.6 (80721.9-93555.2) | 911.4 (833-990.9) | -0.08 (-2.33-2.23) |
| Acute hepatitis | Kingdom of Thailand | 1745581.1 (1584130.9-1924147.7) | 2945.2 (2676.5-3221.7) | 1398373.2 (1244710.5-1553936) | 2610.3 (2387.5-2826.9) | -0.43 (-2.66-1.84) |
| Acute hepatitis | Kingdom of the Netherlands | 163662.1 (152693.3-175051) | 1205.8 (1117-1306.9) | 172659.5 (161626.2-183736.8) | 1159.4 (1071.7-1253.4) | -0.23 (-2.69-2.28) |
| Acute hepatitis | Kingdom of Tonga | 5952.4 (5397.9-6465.5) | 5792.8 (5096.9-6373.7) | 4070.2 (3771.9-4370.4) | 3586.8 (3309.8-3885.5) | -1.07 (-3.54-1.46) |
| Acute hepatitis | Kyrgyz Republic | 204982.7 (192925.8-217785.9) | 3841.8 (3589.3-4101.7) | 248465.4 (234599.1-262292.6) | 3304.2 (3116.3-3502.4) | -0.41 (-2.34-1.57) |
| Acute hepatitis | Lao People's Democratic Republic | 229794 (209057.3-251822.6) | 4643.3 (4073.6-5282.7) | 262095.3 (235320.4-290788.7) | 3297.6 (2953.2-3687.4) | -1.02 (-3.31-1.33) |
| Acute hepatitis | Lebanese Republic | 116174.4 (108924.9-123527.2) | 3433.1 (3214.1-3658.6) | 151397.6 (142511.5-159883.1) | 3006.3 (2838.2-3174) | -0.49 (-3.12-2.22) |
| Acute hepatitis | Malaysia | 679390.8 (633876.2-726228.4) | 3345.8 (3127.1-3567.3) | 789434.5 (740262.2-834429.5) | 2650.9 (2473.3-2821.2) | -0.71 (-2.81-1.44) |
| Acute hepatitis | Mongolia | 146316.9 (136741-156822) | 5747.5 (5284.9-6198.3) | 162172.3 (151512.7-172662.6) | 4563.9 (4273.6-4851) | -0.49 (-2.47-1.54) |
| Acute hepatitis | Montenegro | 14611 (13541.7-15662.4) | 2477.8 (2290.2-2658.6) | 10818.8 (10091.7-11510.9) | 2289.9 (2123-2458) | -0.41 (-2.84-2.09) |
| Acute hepatitis | New Zealand | 60445.6 (53664.5-68008.1) | 1830.6 (1632.1-2045.3) | 72620.5 (66057.8-79853.6) | 1553.6 (1416.5-1696.6) | -0.45 (-2.63-1.77) |
| Acute hepatitis | North Macedonia | 53034.7 (49522-56599.2) | 2784.8 (2591.6-2975) | 39102.8 (36406.1-41531.7) | 2401.1 (2225.1-2570.5) | -0.49 (-2.9-1.97) |
| Acute hepatitis | Northern Mariana Islands | 2140.8 (1946.8-2361) | 4499.9 (4115.7-4916.6) | 1684.6 (1537-1846.1) | 3687.8 (3403.3-3984.4) | -0.4 (-2.94-2.21) |
| Acute hepatitis | Palestine | 105392.1 (99003.6-111843.6) | 3676.9 (3420.1-3925.7) | 189158 (178129.6-199518.2) | 3295.9 (3106.4-3486.2) | -0.54 (-2.92-1.89) |
| Acute hepatitis | People's Democratic Republic of Algeria | 1108289.2 (1042012.9-1174830.6) | 3486.7 (3268.2-3711.6) | 1455089.6 (1372698-1534260.4) | 3204.8 (3027.6-3375.8) | -0.39 (-2.8-2.09) |
| Acute hepatitis | People's Republic of Bangladesh | 8349749.1 (6713280.3-10297683.3) | 7230.4 (5183.5-9734.6) | 6099025.1 (5619198.5-6522312.1) | 3812.8 (3529.9-4074.5) | -1.28 (-3.86-1.38) |
| Acute hepatitis | People's Republic of China | 65757649.2 (58813343.5-72787653.5) | 5493.6 (4952.4-6048.2) | 39808670.7 (35070968-45585505.5) | 3301.1 (2985.1-3671.5) | -1.62 (-4.05-0.88) |
| Acute hepatitis | Plurinational State of Bolivia | 269300.3 (253699.1-285264.7) | 3052.5 (2868.7-3232.9) | 358582.3 (342289.7-375112.3) | 2963.1 (2827.5-3101.4) | -0.28 (-2.74-2.24) |
| Acute hepatitis | Portuguese Republic | 168725.8 (157440.2-182140) | 1911.7 (1772.4-2079.7) | 128357.5 (118983.8-138142.9) | 1536.8 (1415.7-1667.5) | -0.63 (-2.98-1.78) |
| Acute hepatitis | Principality of Andorra | 725.9 (665.3-802.9) | 1455.2 (1336.8-1590) | 937.5 (859.3-1027.1) | 1259.6 (1154.3-1363.1) | -0.55 (-2.75-1.7) |
| Acute hepatitis | Principality of Monaco | 340.2 (313.6-369.6) | 1386.9 (1284.9-1511.5) | 383.5 (355.8-413.4) | 1193 (1098-1295.1) | -0.53 (-2.8-1.79) |
| Acute hepatitis | Puerto Rico | 100093.7 (94074.7-105838.3) | 2865.1 (2689-3034.6) | 53197 (50607.4-55735.6) | 2596.6 (2441-2747.2) | -0.81 (-3.15-1.59) |
| Acute hepatitis | Republic of Albania | 120628.9 (114016.8-127316.9) | 3212.6 (3039.5-3388.3) | 56503.8 (53763.3-59186.1) | 2937.6 (2771.3-3092.4) | -0.69 (-3.21-1.89) |
| Acute hepatitis | Republic of Angola | 1068927.9 (963149-1168163.7) | 7672.7 (6691.7-8689.5) | 2115816.3 (1899813.1-2314241.8) | 5255.8 (4501.4-5966.4) | -0.92 (-3.1-1.3) |
| Acute hepatitis | Republic of Armenia | 119215.3 (111537.8-126726.1) | 3298.1 (3090.6-3506.5) | 71753.4 (67100.1-76003.6) | 2921.5 (2731.1-3092.5) | -0.36 (-2.33-1.64) |
| Acute hepatitis | Republic of Austria | 103625.6 (95804.1-112214.4) | 1522.4 (1407.2-1652) | 103607.3 (95938.9-110884.3) | 1372.4 (1265.1-1487.8) | -0.6 (-2.91-1.77) |
| Acute hepatitis | Republic of Azerbaijan | 315515.7 (299179.9-333006) | 3853.8 (3641.9-4086.8) | 324507.1 (305086.3-343488.4) | 3512.8 (3305.8-3699.2) | -0.35 (-2.3-1.64) |
| Acute hepatitis | Republic of Belarus | 248702.6 (234141.3-264003.3) | 2786.6 (2613-2955.4) | 151252.8 (140801.3-161200.2) | 2304.1 (2131.8-2473.8) | -0.72 (-3.05-1.67) |
| Acute hepatitis | Republic of Benin | 351400.1 (322106.2-379536.8) | 5636.8 (4979.7-6338.7) | 754598.5 (700566.3-812414.1) | 4464.4 (4048.7-4921.5) | -0.63 (-2.94-1.73) |
| Acute hepatitis | Republic of Botswana | 82008 (75320.7-89750.7) | 4804 (4298.1-5313.2) | 90544.7 (83383.3-98003.3) | 3731.8 (3454.3-4034.4) | -0.77 (-3.1-1.61) |
| Acute hepatitis | Republic of Bulgaria | 242672.4 (221249.5-265378.9) | 3493.7 (3213.8-3765.3) | 133060.2 (120619.9-147239.5) | 2988.5 (2791.5-3209) | -0.72 (-3.28-1.9) |
| Acute hepatitis | Republic of Burundi | 350334.1 (326804.9-373752.9) | 4376.5 (3984-4807.1) | 680441 (642974.8-719478.5) | 3865.8 (3606.2-4140.3) | -0.45 (-2.42-1.55) |
| Acute hepatitis | Republic of Cabo Verde | 22412.2 (20253.7-24573.4) | 4958.4 (4335.6-5574.2) | 21677.5 (19202.8-24635.2) | 4035.8 (3613.5-4534.2) | -0.73 (-3.06-1.65) |
| Acute hepatitis | Republic of Cameroon | 712728.2 (658297.9-772185.8) | 5065.8 (4508.9-5659.7) | 1659052.8 (1545519.8-1774537.2) | 4297.8 (3922.4-4702.1) | -0.55 (-2.75-1.7) |
| Acute hepatitis | Republic of Chad | 508664.3 (454316.5-559666.1) | 6953.9 (5834.4-8151.2) | 1286499.7 (1158006.5-1407633.2) | 5756.9 (4893.4-6586.8) | -0.45 (-2.87-2.02) |
| Acute hepatitis | Republic of Chile | 410680.9 (390041.1-430900.1) | 2915.9 (2769-3054.9) | 378509.5 (357141.4-398751.5) | 2496.6 (2327.1-2654) | -0.38 (-3.01-2.32) |
| Acute hepatitis | Republic of Colombia | 1419593.5 (1299275.1-1578646.8) | 3680.8 (3359.3-4103.8) | 1235320.3 (1127014.4-1344448.9) | 2897.8 (2665.8-3121.4) | -0.92 (-3.39-1.62) |
| Acute hepatitis | Republic of Costa Rica | 108420.2 (102075.1-115130.2) | 2916.8 (2744.8-3099.1) | 106267.5 (100649.7-111601.2) | 2750.1 (2594.9-2897) | -0.59 (-3.06-1.95) |
| Acute hepatitis | Republic of C么te d'Ivoire | 876623.3 (801222.5-954355) | 5776.8 (5046.6-6489.7) | 1482555.7 (1360109.5-1609835.1) | 4402.6 (3953.1-4895.8) | -0.66 (-3-1.74) |
| Acute hepatitis | Republic of Croatia | 106174.7 (98191.6-115280.7) | 2558.6 (2364.3-2765.6) | 68108 (62990.1-73298.8) | 2180.9 (2013.3-2340.2) | -0.78 (-3.09-1.6) |
| Acute hepatitis | Republic of Cuba | 289259 (273465.8-305181.6) | 2888.4 (2722.7-3048.1) | 198751.9 (188043.6-209154) | 2596.5 (2435-2753.9) | -0.82 (-3.26-1.67) |
| Acute hepatitis | Republic of Cyprus | 11487.9 (10266.5-12733.4) | 1508.8 (1349.5-1665.3) | 15436.7 (14208.8-16706) | 1209.2 (1107.3-1313.4) | -0.74 (-3.01-1.58) |
| Acute hepatitis | Republic of Djibouti | 24198.5 (22223.4-26152.3) | 4495.9 (4040.9-4931.3) | 56703.3 (51939.4-61927.4) | 4103.1 (3748.4-4499.9) | -0.35 (-2.55-1.89) |
| Acute hepatitis | Republic of Ecuador | 360314.6 (341260.2-379011.3) | 2917 (2776.8-3064) | 521665.9 (495925.1-546426.3) | 2960.6 (2808.4-3106.3) | -0.17 (-2.7-2.42) |
| Acute hepatitis | Republic of El Salvador | 219600.4 (206531.6-232836.7) | 3112.2 (2907.8-3317.9) | 180615.6 (170999.2-190387.4) | 2853.4 (2696.5-3013.6) | -0.75 (-3.18-1.75) |
| Acute hepatitis | Republic of Equatorial Guinea | 25073.4 (23555.9-26641.2) | 4110.9 (3817.6-4443) | 65312.1 (61473-69140.3) | 3642.7 (3423.4-3872.6) | -0.63 (-2.56-1.34) |
| Acute hepatitis | Republic of Estonia | 32916.3 (30657.6-35268.5) | 2439.2 (2262.9-2618.8) | 20299.4 (18878.8-21589.9) | 2148.9 (1978-2291.2) | -0.63 (-2.86-1.66) |
| Acute hepatitis | Republic of Fiji | 28411.3 (26279.2-30633.6) | 3262.4 (3011-3522.3) | 25545.1 (23822.8-27435.4) | 2738.1 (2551.5-2942.7) | -0.63 (-2.99-1.79) |
| Acute hepatitis | Republic of Finland | 52032.8 (47215.3-57085.5) | 1107.4 (1006-1213.3) | 49611.2 (45358.6-53956.6) | 994.7 (903.9-1087.4) | -0.08 (-2.13-2.02) |
| Acute hepatitis | Republic of Ghana | 1224788.4 (1101667.9-1376182.7) | 7119.1 (6030.1-8368.4) | 1758726.3 (1605566.4-1941908) | 4598 (4111.9-5227.1) | -1.01 (-3.4-1.45) |
| Acute hepatitis | Republic of Guatemala | 447262.2 (418090.1-476281.6) | 3491.2 (3226.1-3742.2) | 530585.2 (492017.7-567144.7) | 3260.1 (3030.1-3497.6) | -0.59 (-2.92-1.79) |
| Acute hepatitis | Republic of Guinea | 493158.3 (442140.5-547886.8) | 7038.5 (5942.7-8285.1) | 858172.6 (782404.9-939125.4) | 5375.3 (4757.1-6087.1) | -0.63 (-3.05-1.86) |
| Acute hepatitis | Republic of Guinea-Bissau | 88833.3 (78746.2-98697.9) | 8034.6 (6528.6-9641.6) | 130975.5 (118534.9-145536.7) | 5514.8 (4822-6244.4) | -0.87 (-3.49-1.83) |
| Acute hepatitis | Republic of Guyana | 33354.7 (31380.6-35477.7) | 3432 (3222.1-3658.7) | 22695.6 (21350.6-24030.5) | 2965.2 (2788.3-3145.2) | -0.6 (-2.8-1.65) |
| Acute hepatitis | Republic of Haiti | 298356.6 (280346.8-315541.5) | 3465.9 (3262.1-3669.8) | 481005.4 (455951.3-507768.2) | 3279.4 (3111-3464.8) | -0.33 (-2.47-1.85) |
| Acute hepatitis | Republic of Honduras | 210906.9 (197937.2-224591.4) | 3143.2 (2947.9-3348.7) | 306020.6 (288083.9-323882.8) | 2778.4 (2608.8-2947.7) | -0.68 (-2.98-1.67) |
| Acute hepatitis | Republic of Iceland | 2342.7 (2142.9-2543.2) | 946.1 (868.1-1025.9) | 3054.7 (2816.5-3303.2) | 933.2 (855.4-1009.7) | -0.07 (-2.17-2.06) |
| Acute hepatitis | Republic of India | 57629613.3 (48742880.5-68200538.7) | 5744 (4687.1-7039.2) | 45503271.2 (41702237.5-48934506.7) | 3488 (3227.4-3730.5) | -1.13 (-3.66-1.46) |
| Acute hepatitis | Republic of Indonesia | 10607134.6 (9528909.5-11743074.2) | 5473.1 (4768.4-6202.1) | 9314179.3 (8617138.6-10036656.7) | 3526.6 (3290.1-3776.9) | -0.97 (-3.27-1.39) |
| Acute hepatitis | Republic of Iraq | 841853.8 (784372.5-903433.5) | 3418.1 (3099.7-3773.6) | 1305593.1 (1238471.4-1367415.7) | 2976.8 (2823.1-3125.2) | -0.4 (-2.74-2) |
| Acute hepatitis | Republic of Italy | 1159053.4 (1051584.5-1289046.1) | 2273.3 (2078.5-2504.7) | 860818.8 (785778.5-950663.8) | 1840.1 (1697.9-1990.2) | -1.34 (-3.48-0.85) |
| Acute hepatitis | Republic of Kazakhstan | 561744.7 (533959.4-592705.4) | 3165.4 (3010.5-3344.2) | 527386.1 (493505.8-557672.4) | 2782.3 (2604.9-2937.1) | -0.34 (-2.44-1.81) |
| Acute hepatitis | Republic of Kenya | 1585944.9 (1457638.1-1718517) | 5111.1 (4563.9-5705.6) | 2220052.5 (2042297.4-2398334.3) | 4013.4 (3676.4-4378) | -0.62 (-2.84-1.64) |
| Acute hepatitis | Republic of Kiribati | 5325.8 (4862.4-5791.5) | 6560.7 (5872.6-7299.1) | 5517.4 (5050.8-5992) | 4308.2 (3918.2-4722.7) | -0.75 (-3.32-1.88) |
| Acute hepatitis | Republic of Korea | 1527324.6 (1399248.5-1689708) | 3435.7 (3159.6-3759.4) | 1151897.2 (1058671.1-1262066.6) | 2461.3 (2276.6-2646.9) | -0.92 (-3.45-1.68) |
| Acute hepatitis | Republic of Latvia | 51917.2 (47844.2-55884.8) | 2325.5 (2137.1-2503.8) | 27074.1 (25135-28904.3) | 2133.8 (1964.4-2283.7) | -0.58 (-2.95-1.84) |
| Acute hepatitis | Republic of Liberia | 194692.8 (175713.9-213388.3) | 6635.9 (5674.5-7561.8) | 339810.3 (310078.7-372696.3) | 5611.8 (5019.5-6272.4) | -0.56 (-3.01-1.95) |
| Acute hepatitis | Republic of Lithuania | 77443.5 (71398.7-82870.3) | 2405.3 (2210.4-2582.6) | 40084.3 (37258-42569.2) | 2176.1 (2008.7-2319.4) | -0.6 (-2.85-1.7) |
| Acute hepatitis | Republic of Madagascar | 831241.7 (753905-909679.7) | 5041.8 (4442.7-5656.2) | 1517890.8 (1395207.1-1648378.1) | 4312.2 (3863.5-4820.2) | -0.48 (-2.83-1.93) |
| Acute hepatitis | Republic of Malawi | 684125.5 (628450.1-743881.6) | 5005.7 (4418.4-5666.3) | 957687.4 (885916-1031987.9) | 4079.8 (3732.2-4462.3) | -0.75 (-2.93-1.48) |
| Acute hepatitis | Republic of Maldives | 10328.6 (9543.7-11243.9) | 3367.5 (3084.7-3686.1) | 11780.6 (10819.3-12923.6) | 2546.9 (2348.6-2761.4) | -1.05 (-3.11-1.06) |
| Acute hepatitis | Republic of Mali | 586967.1 (541862.2-634577.8) | 4894.1 (4364.8-5510) | 1397016.8 (1305181.5-1500152.7) | 4311.9 (3913.2-4744.5) | -0.51 (-2.71-1.75) |
| Acute hepatitis | Republic of Malta | 5517.1 (5111.8-5952) | 1608.1 (1486.1-1741.9) | 5062.9 (4719.6-5421.4) | 1430.5 (1319.8-1555.6) | -0.61 (-3.06-1.9) |
| Acute hepatitis | Republic of Mauritius | 38568.3 (35496-42193.2) | 3367.1 (3107.7-3670.8) | 26686.6 (23692.9-29519.8) | 2641.8 (2410.3-2864) | -0.8 (-2.88-1.32) |
| Acute hepatitis | Republic of Moldova | 160840.1 (144909.7-179336.2) | 3676.1 (3320.6-4096.1) | 77560.9 (69335-88080.3) | 2878 (2640.1-3135.2) | -0.7 (-2.73-1.37) |
| Acute hepatitis | Republic of Mozambique | 1005777.6 (911918.2-1099687.5) | 5880.6 (5177.7-6651.6) | 1820462.3 (1684724.5-1956862.6) | 4699.5 (4189.2-5239.1) | -0.55 (-2.94-1.89) |
| Acute hepatitis | Republic of Namibia | 87411.8 (80215.7-94334.8) | 4828.6 (4362.5-5319.2) | 109147.3 (100068.7-118664.5) | 4091.2 (3740.3-4476.5) | -0.47 (-2.9-2.02) |
| Acute hepatitis | Republic of Nauru | 483.8 (438.1-532.2) | 4088.1 (3665.2-4559.3) | 380.8 (349.7-410) | 3130.4 (2834.7-3420.3) | -0.64 (-3-1.78) |
| Acute hepatitis | Republic of Nicaragua | 177543.1 (167030.1-188614.4) | 3007.9 (2794-3200.7) | 193027.2 (184074.6-202691) | 2835.6 (2703.2-2983.2) | -0.65 (-3.07-1.82) |
| Acute hepatitis | Republic of Niue | 87.3 (79.6-97) | 3596.5 (3248.5-4029.8) | 40.7 (36.8-44.7) | 2712.8 (2489.3-2948.1) | -0.83 (-3.2-1.6) |
| Acute hepatitis | Republic of Palau | 631.8 (572.4-692.4) | 4008.5 (3632.8-4379.4) | 487.5 (438.6-546.4) | 3097.3 (2838.5-3383.4) | -0.63 (-3.04-1.83) |
| Acute hepatitis | Republic of Panama | 81508.4 (76732.6-86337.9) | 2917.8 (2745.4-3091.8) | 110919.9 (105151.5-116704.5) | 2762.3 (2613.3-2915) | -0.52 (-3.1-2.12) |
| Acute hepatitis | Republic of Paraguay | 150420.7 (140304.6-160509.7) | 2904.7 (2730.8-3083.8) | 175075.5 (162136.3-187541) | 2440.3 (2257.5-2614.7) | -0.59 (-3.01-1.89) |
| Acute hepatitis | Republic of Peru | 887439 (829755.9-942497.9) | 3319 (3081.7-3544.8) | 1099174.4 (1037150.9-1160752.1) | 3146.3 (2970.7-3316.3) | -0.42 (-2.96-2.19) |
| Acute hepatitis | Republic of Poland | 932615.6 (879342.3-989146) | 2652 (2487-2811.2) | 570719.3 (537520.9-605484.1) | 2067.6 (1913.7-2212.9) | -1.21 (-3.51-1.14) |
| Acute hepatitis | Republic of Rwanda | 490121.4 (445736.3-541214.2) | 5194 (4452.7-6143.4) | 597914.6 (553938.9-640262.7) | 3804.7 (3480.9-4141.9) | -1.15 (-3.3-1.05) |
| Acute hepatitis | Republic of San Marino | 290.4 (266.4-317.4) | 1370 (1260.1-1495.2) | 336.3 (310.5-361.9) | 1193.2 (1095.8-1294.2) | -0.37 (-2.77-2.08) |
| Acute hepatitis | Republic of Senegal | 561808.8 (511925.1-609835.4) | 5601.3 (4866.8-6286.4) | 843952.5 (763301.1-926203.8) | 4609.5 (4057.1-5199.3) | -0.51 (-3.03-2.07) |
| Acute hepatitis | Republic of Serbia | 236788.4 (222276-250083.9) | 2910.2 (2720.4-3074.9) | 161843 (152111.7-170392.9) | 2741.4 (2573.2-2903) | -0.43 (-2.94-2.15) |
| Acute hepatitis | Republic of Seychelles | 2770.8 (2549.4-3036.7) | 3621.4 (3303.6-3981.3) | 2592.5 (2364.3-2839.4) | 2677 (2445.8-2907.2) | -0.76 (-3-1.53) |
| Acute hepatitis | Republic of Sierra Leone | 302692.1 (272923-335384.4) | 6117.2 (5159.9-7174.8) | 488271.2 (442595.1-537180.4) | 4648 (4115.7-5266.2) | -0.71 (-3.04-1.67) |
| Acute hepatitis | Republic of Singapore | 82123.6 (74067.7-91509.9) | 2703.3 (2468.2-2981.5) | 114798.2 (104170.4-125857.8) | 2081.7 (1917.2-2248.6) | -0.69 (-2.76-1.43) |
| Acute hepatitis | Republic of Slovenia | 43799.7 (40792.5-47043.2) | 2603.4 (2421.9-2802.7) | 34111.7 (31569.8-36470.5) | 2316.3 (2152.8-2475.9) | -0.61 (-3-1.84) |
| Acute hepatitis | Republic of South Africa | 1605102.9 (1492825.7-1719075.5) | 3599.3 (3346-3853.6) | 1643722.9 (1527109.6-1756179.1) | 3112.6 (2915.6-3312.1) | -0.64 (-2.9-1.66) |
| Acute hepatitis | Republic of South Sudan | 384474.9 (350838.5-418795.2) | 4926 (4343-5499.8) | 587457.1 (536240.4-640283.9) | 4728 (4210.1-5237) | -0.18 (-2.4-2.08) |
| Acute hepatitis | Republic of Sudan | 1706506.7 (1514185.2-1917575.7) | 7467.7 (6095.8-9088.7) | 2042926.8 (1867317.7-2204819.8) | 4200.2 (3788.8-4628.4) | -1.25 (-3.87-1.44) |
| Acute hepatitis | Republic of Suriname | 13635.2 (12857.6-14385.8) | 3124.1 (2937.3-3305.5) | 14697.6 (13857-15531.4) | 2834 (2665.1-2999.6) | -0.59 (-2.86-1.75) |
| Acute hepatitis | Republic of Tajikistan | 277441.9 (262710.9-292220.3) | 3726.4 (3502.4-3947.8) | 415381.3 (391792-439081.7) | 3477.7 (3281.9-3684.3) | -0.33 (-2.25-1.62) |
| Acute hepatitis | Republic of the Congo | 162084.6 (147044.2-177993.9) | 5429.2 (4795.1-6144.8) | 258677.2 (233718-285762.7) | 4338.4 (3873.7-4846.5) | -0.62 (-2.68-1.48) |
| Acute hepatitis | Republic of the Gambia | 65193 (60237.3-70098.3) | 5172.7 (4655.6-5718.7) | 112606 (106752.8-118654.9) | 3967.5 (3707.4-4216.3) | -0.72 (-3.04-1.66) |
| Acute hepatitis | Republic of the Marshall Islands | 2769.6 (2552.9-2990.3) | 4764.2 (4315-5252.2) | 2218 (2049.1-2412.2) | 3810.8 (3522.1-4161.8) | -0.53 (-2.98-1.98) |
| Acute hepatitis | Republic of the Niger | 698801.9 (629594.4-771498.5) | 7109 (5993.9-8313) | 1757047.2 (1603047-1906058.2) | 5441.2 (4663.7-6180.2) | -0.6 (-2.99-1.86) |
| Acute hepatitis | Republic of the Philippines | 3629674 (3267342-4026747.8) | 5068.2 (4476.4-5719.7) | 4176193.3 (3763122.2-4655356.4) | 3597.6 (3233.1-4037) | -0.78 (-3.19-1.7) |
| Acute hepatitis | Republic of the Union of Myanmar | 1491554.8 (1388608.6-1591311.5) | 3245.9 (3006.3-3494.8) | 1527961.5 (1424436.5-1629522.4) | 2739.3 (2549-2927.5) | -0.6 (-2.65-1.49) |
| Acute hepatitis | Republic of Trinidad and Tobago | 39665.1 (37380.6-41904.5) | 2966.6 (2790.3-3143.8) | 28893.2 (27120.3-30440.8) | 2656.6 (2481.4-2812.9) | -0.62 (-2.96-1.77) |
| Acute hepatitis | Republic of Tunisia | 350382.4 (326283-376387) | 3597.3 (3318.1-3893.4) | 314160.4 (294948.4-332675.5) | 3004.5 (2830.4-3179.5) | -0.53 (-3-2) |
| Acute hepatitis | Republic of Turkey | 2213312.2 (2071158.6-2372435.2) | 3427.2 (3179.7-3697.8) | 2041197.4 (1914305.5-2152008) | 2778.7 (2600-2936.5) | -0.63 (-3.22-2.04) |
| Acute hepatitis | Republic of Uganda | 1306192.7 (1207696-1411427.6) | 5198.4 (4623.5-5817.8) | 2478960 (2310677-2658135.4) | 4340.9 (3930.5-4779.9) | -0.7 (-2.84-1.5) |
| Acute hepatitis | Republic of Uzbekistan | 1306413.1 (1215734.8-1397220.4) | 5038.9 (4627.6-5451.3) | 1450758 (1338819.7-1563104.5) | 4027.2 (3717.4-4341.3) | -0.47 (-2.53-1.64) |
| Acute hepatitis | Republic of Vanuatu | 8729.2 (7861.9-9619.3) | 4762.1 (4185.7-5362.4) | 13413 (12366.7-14597.8) | 3819.5 (3469.2-4226.2) | -0.43 (-2.84-2.03) |
| Acute hepatitis | Republic of Yemen | 1256443 (1130465.5-1398957.9) | 7606.5 (6303-9010.9) | 1586128.2 (1465936.1-1697388.4) | 4131.6 (3779.1-4510.7) | -1.27 (-4-1.55) |
| Acute hepatitis | Republic of Zambia | 552044.9 (505966.3-594950.5) | 5185.3 (4565.7-5777.5) | 994785.8 (938396.8-1053572.8) | 4114 (3833.9-4410.1) | -0.73 (-2.92-1.52) |
| Acute hepatitis | Republic of Zimbabwe | 909584.3 (807751.6-1013322.6) | 7321.7 (6175.4-8422.6) | 1008625 (896295.5-1123702.4) | 5871.7 (5045.1-6741.3) | -0.29 (-2.95-2.45) |
| Acute hepatitis | Romania | 712063.8 (647003.5-782940.6) | 3358.2 (3055.7-3678.6) | 357426.3 (321052.4-398771.2) | 2389.6 (2194.3-2622.6) | -0.97 (-3.21-1.32) |
| Acute hepatitis | Russian Federation | 3338909 (3120530.7-3564162.1) | 2526.9 (2356.3-2697) | 2543916.8 (2373051.6-2701733.4) | 2405.7 (2241.4-2554.3) | -0.38 (-2.78-2.08) |
| Acute hepatitis | Saint Kitts and Nevis | 1430.5 (1342-1512) | 3098.2 (2902-3286.7) | 1222 (1155.6-1292.2) | 2728.2 (2557.3-2886.4) | -0.84 (-3.12-1.49) |
| Acute hepatitis | Saint Lucia | 5141.3 (4845.9-5433) | 3108.4 (2933.9-3287) | 3657.1 (3452.7-3871.1) | 2766.7 (2585.7-2941.8) | -0.7 (-2.99-1.65) |
| Acute hepatitis | Saint Vincent and the Grenadines | 4044.1 (3814.8-4252.1) | 3117.8 (2934.7-3284.3) | 2632.3 (2476.6-2769.4) | 2801.4 (2621-2969.4) | -0.65 (-2.98-1.73) |
| Acute hepatitis | Slovak Republic | 128568.1 (119420.4-138253.9) | 2641.4 (2451.6-2841.8) | 94507.6 (87411.6-100748.8) | 2318 (2141.3-2481.8) | -0.6 (-2.96-1.82) |
| Acute hepatitis | Socialist Republic of Viet Nam | 4221886.7 (3837644-4663763.6) | 5558 (4975.8-6240.1) | 3815836.5 (3388701.9-4289254.6) | 3940.1 (3535.6-4379.5) | -0.91 (-3.41-1.65) |
| Acute hepatitis | Solomon Islands | 21398.3 (19260.8-23581.1) | 5325.5 (4669.7-6035.8) | 28640.9 (25965.3-31304.4) | 3679.6 (3252.8-4143.6) | -0.63 (-3.09-1.89) |
| Acute hepatitis | State of Eritrea | 244422.4 (220900.2-267946.3) | 5449.8 (4717.6-6295.2) | 337710.2 (306405.8-369137.3) | 4337.7 (3848-4808.3) | -0.64 (-2.84-1.61) |
| Acute hepatitis | State of Israel | 75233.9 (69486.5-81669.3) | 1490.7 (1382.8-1612.6) | 121885 (112261.9-131086.6) | 1300.3 (1194.5-1401.2) | -0.72 (-3.2-1.81) |
| Acute hepatitis | State of Kuwait | 54743.5 (51673.6-57858.6) | 2850.5 (2691.6-3005.1) | 95887.3 (90999.7-101061.6) | 2626.1 (2473.9-2766.8) | -0.58 (-3.02-1.92) |
| Acute hepatitis | State of Libya | 184529.3 (173344.7-195600.3) | 3487.4 (3227.9-3745.7) | 179260.7 (168367.8-189431.4) | 3015.6 (2840.6-3180) | -0.57 (-3.05-1.98) |
| Acute hepatitis | State of Qatar | 16762 (15646.8-18017.7) | 3607.2 (3388.5-3845.8) | 77164.4 (71306.7-82989.2) | 3077.1 (2902-3246.7) | -0.6 (-2.99-1.84) |
| Acute hepatitis | Sultanate of Oman | 119544.8 (105384.9-134816.7) | 5943 (4739.3-7354.3) | 142808.5 (132554.1-153891.4) | 3150.9 (2943.9-3380.5) | -1.4 (-3.92-1.18) |
| Acute hepatitis | Swiss Confederation | 76141.6 (69962.4-82782.9) | 1219.1 (1127.4-1323.5) | 94810.2 (87060.9-102816.2) | 1203.7 (1098.7-1310.6) | -0.05 (-2.17-2.13) |
| Acute hepatitis | Syrian Arab Republic | 687842.3 (640873.8-733952.1) | 4229.5 (3884.3-4611) | 434601.4 (408970.9-459172) | 3422.2 (3219.8-3611.7) | -0.58 (-3.02-1.93) |
| Acute hepatitis | Taiwan (Province of China) | 1153842.6 (1027238.2-1310936.6) | 5449.7 (4877.9-6128.4) | 614660.7 (533999.7-716032.9) | 2953.6 (2694-3262.6) | -1.15 (-3.49-1.25) |
| Acute hepatitis | Togolese Republic | 264989.1 (239131.3-290158.8) | 5518.6 (4833.1-6356.7) | 456892.4 (414645.9-497915) | 4719 (4183.7-5233.8) | -0.46 (-2.82-1.95) |
| Acute hepatitis | Tokelau | 71.4 (65.5-79) | 4128.1 (3748-4609.8) | 41.1 (37.5-45) | 3167.2 (2891-3467.6) | -0.7 (-3.13-1.78) |
| Acute hepatitis | Turkmenistan | 193521.5 (182360.1-204480.5) | 4012.4 (3765-4246.9) | 177556.6 (168445.8-187638.4) | 3334.8 (3160.7-3524.3) | -0.39 (-2.34-1.59) |
| Acute hepatitis | Tuvalu | 476.7 (433.9-528.2) | 4434.2 (3990.7-4952.3) | 449.9 (407.1-497.7) | 3566.5 (3223.3-3970.2) | -0.4 (-2.83-2.08) |
| Acute hepatitis | Ukraine | 1031683.1 (964128.9-1093228.4) | 2340.3 (2176.5-2483.8) | 673404.1 (627599.6-713058) | 2321.6 (2150.6-2468.9) | 0.21 (-1.85-2.31) |
| Acute hepatitis | Union of the Comoros | 29621.2 (27117.3-32236.1) | 4619.2 (4138-5199) | 31503.4 (28874.4-34062.5) | 3964.9 (3618.6-4297) | -0.57 (-2.74-1.66) |
| Acute hepatitis | United Arab Emirates | 61042 (57137.9-65062.3) | 2948.5 (2776.9-3137.7) | 184646.3 (171111.8-197978.3) | 2691.5 (2536.2-2844.5) | -0.52 (-2.92-1.95) |
| Acute hepatitis | United Kingdom of Great Britain and Northern Ireland | 774949.9 (723129.4-830555.2) | 1526.9 (1419.3-1646.5) | 842455 (787648.8-900115.6) | 1443.8 (1339.3-1549.2) | 0.07 (-2.13-2.32) |
| Acute hepatitis | United Mexican States | 3263931.4 (3066279.4-3456005.8) | 2949.9 (2772.2-3122.1) | 3122824.2 (2971264.2-3275674) | 2764.7 (2625.1-2910.5) | -0.66 (-3.18-1.92) |
| Acute hepatitis | United Republic of Tanzania | 1719774.1 (1584836.2-1851099.1) | 4841.6 (4321.3-5347.9) | 2946006.8 (2769185.9-3125464.9) | 4028.4 (3732.2-4334) | -0.55 (-2.62-1.57) |
| Acute hepatitis | United States of America | 3500662.2 (3262149.1-3733946.9) | 1497.6 (1391.1-1609.9) | 4058302.7 (3807657.2-4307372) | 1465.1 (1354.7-1569.2) | -0.49 (-2.81-1.89) |
| Acute hepatitis | United States Virgin Islands | 3181.5 (2997-3368.1) | 2912.8 (2742.6-3083.6) | 1475.1 (1396.4-1565.7) | 2619 (2461.7-2783.5) | -0.71 (-3.07-1.7) |
| Acute hepatitis A | American Samoa | 1263.6 (1157.3-1368) | 1949.2 (1806.4-2091.1) | 908.7 (841-975.3) | 1965.1 (1812.9-2122.3) | -0.45 (-2.97-2.13) |
| Acute hepatitis A | Antigua and Barbuda | 1479.9 (1396.4-1563) | 2344.7 (2208.2-2483.5) | 1490.9 (1410.6-1564.5) | 2213.8 (2062-2356.5) | -0.73 (-3.08-1.68) |
| Acute hepatitis A | Arab Republic of Egypt | 2634969.3 (2178487-3164562.9) | 4181.3 (3111.7-5541.2) | 2868586.6 (2680713.6-3065003.7) | 2322.4 (2181.4-2480.3) | -1.35 (-3.54-0.88) |
| Acute hepatitis A | Argentine Republic | 807854.6 (747527.4-867436) | 2368.1 (2194.5-2540.3) | 771614.8 (718516.1-822339.8) | 2023.4 (1869.5-2179.6) | -0.42 (-3.45-2.7) |
| Acute hepatitis A | Australia | 177660.7 (161989.7-192998.9) | 1155.2 (1046.3-1264.4) | 252047.2 (232625.8-271954.1) | 1211.6 (1105.6-1323.6) | -0.51 (-2.65-1.68) |
| Acute hepatitis A | Barbados | 5166.5 (4871.8-5428) | 2287.2 (2140.6-2419.5) | 4146.1 (3918.5-4375.6) | 2162.7 (2001.7-2310.9) | -0.57 (-2.97-1.89) |
| Acute hepatitis A | Belize | 6663.9 (6252.4-7109.4) | 2439.2 (2282.7-2601.2) | 10245.6 (9737.8-10780.1) | 2383.7 (2250.8-2523.5) | -0.55 (-2.82-1.78) |
| Acute hepatitis A | Bermuda | 1082.9 (1019.1-1147) | 2263.6 (2122.5-2411.2) | 764.2 (722.4-802.3) | 2159.6 (2012.4-2296.8) | -0.63 (-3.06-1.85) |
| Acute hepatitis A | Bolivarian Republic of Venezuela | 603020 (567056.9-640155) | 2482.9 (2333.3-2631.8) | 558800.5 (526823.4-591772.7) | 2401.3 (2255.7-2550.8) | -0.51 (-3.05-2.1) |
| Acute hepatitis A | Bosnia and Herzegovina | 78008.6 (72157.4-84315.8) | 1904 (1750.4-2074.3) | 35183.3 (32374.8-37680.9) | 1644.4 (1495.2-1785.3) | -0.57 (-3.15-2.07) |
| Acute hepatitis A | Brunei Darussalam | 3894.3 (3512.2-4305.4) | 1291.7 (1176.8-1419.6) | 5132 (4669.5-5583.9) | 1254.6 (1135.1-1378.4) | -0.81 (-2.87-1.29) |
| Acute hepatitis A | Burkina Faso | 455903.3 (420767-495048.8) | 3192.4 (2711-3722.9) | 932485.1 (874580.2-992236.8) | 2679.1 (2472.9-2899.7) | -0.66 (-2.95-1.69) |
| Acute hepatitis A | Canada | 240033.1 (220099.9-259515) | 968.6 (881.6-1057) | 289818.2 (267952.8-313769.5) | 953.1 (860.7-1056.5) | -0.24 (-2.51-2.09) |
| Acute hepatitis A | Central African Republic | 137288.8 (127939.3-146885.8) | 3205.6 (2959.9-3472.3) | 196201.3 (184745.6-208215.3) | 2556.4 (2400-2713.1) | -0.46 (-2.59-1.73) |
| Acute hepatitis A | Commonwealth of Dominica | 2018.9 (1902.7-2137.7) | 2383.8 (2244.5-2521.7) | 1138 (1081.5-1193.1) | 2263.6 (2116.3-2414.4) | -0.6 (-2.94-1.79) |
| Acute hepatitis A | Commonwealth of the Bahamas | 6433.5 (6035.5-6815.8) | 2302.2 (2150.3-2454.9) | 6652 (6292.6-7012.4) | 2190.7 (2038.3-2350.5) | -0.6 (-2.92-1.79) |
| Acute hepatitis A | Cook Islands | 420.4 (387-451.7) | 1907.2 (1760.8-2045.6) | 266.4 (246.1-285.4) | 1941.2 (1782.2-2097.2) | -0.78 (-3.18-1.68) |
| Acute hepatitis A | Czech Republic | 129819.7 (117897.9-140355.6) | 1542 (1391.2-1680.2) | 104630.2 (96708-112765.3) | 1438.8 (1313-1576) | -0.45 (-2.68-1.82) |
| Acute hepatitis A | Democratic People's Republic of Korea | 443872.9 (409975.3-478595.3) | 2029 (1874.8-2187.1) | 363661.1 (337264.6-388605.8) | 1793.3 (1639.6-1943.9) | -0.67 (-3.12-1.86) |
| Acute hepatitis A | Democratic Republic of Sao Tome and Principe | 5035.9 (4677.6-5398.6) | 2838.6 (2530.6-3138.1) | 6344.1 (6023.3-6703.7) | 2484.8 (2352.7-2635.7) | -0.69 (-3.1-1.79) |
| Acute hepatitis A | Democratic Republic of the Congo | 1596028.9 (1494919.3-1706603.5) | 2522 (2359.9-2679.6) | 3116306.4 (2949639.7-3292439) | 2463.6 (2332.4-2601.2) | -0.35 (-2.52-1.87) |
| Acute hepatitis A | Democratic Republic of Timor-Leste | 25029.7 (22846.5-27165.4) | 2180 (2005.9-2340.8) | 34494.6 (31602.1-37358.5) | 1979 (1819.2-2135.9) | -0.54 (-2.76-1.73) |
| Acute hepatitis A | Democratic Socialist Republic of Sri Lanka | 309408.6 (283162.5-336774.5) | 1646.4 (1505.4-1800.7) | 280232.9 (258712.4-304639.6) | 1465.5 (1342.5-1607.2) | -0.61 (-2.68-1.5) |
| Acute hepatitis A | Dominican Republic | 235143.9 (221373-250760.6) | 2497.2 (2352.8-2657.7) | 256735.9 (241800.1-271192.4) | 2407.5 (2262-2549.4) | -0.49 (-2.76-1.84) |
| Acute hepatitis A | Eastern Republic of Uruguay | 65648.8 (61145-69647) | 2281.1 (2120.8-2426.9) | 52286.6 (48532.3-55693.9) | 2021.1 (1859-2179.2) | -0.48 (-3.3-2.42) |
| Acute hepatitis A | Federal Democratic Republic of Ethiopia | 2811468.5 (2420776.5-3303488.4) | 4053.5 (3016.2-5405.1) | 4025681.6 (3647567.6-4403257.2) | 2741.5 (2397.1-3106) | -0.9 (-3.11-1.35) |
| Acute hepatitis A | Federal Democratic Republic of Nepal | 1445125.8 (1262827.9-1634295.1) | 5454.1 (4454.2-6581) | 792089.2 (739776.6-844775.3) | 2473.8 (2308.1-2642.2) | -1.14 (-3.81-1.6) |
| Acute hepatitis A | Federal Republic of Germany | 623189.8 (576527.9-668247) | 922.3 (838.9-1004.9) | 637659.6 (588996.2-685440.9) | 944.2 (849.9-1037.5) | -0.26 (-2.49-2.02) |
| Acute hepatitis A | Federal Republic of Nigeria | 5190793.3 (4355763.5-6358755) | 5411.8 (3912.9-7500.7) | 8171515.5 (7695438.3-8666890.6) | 2498.3 (2326.3-2688.8) | -1.48 (-3.87-0.98) |
| Acute hepatitis A | Federal Republic of Somalia | 456623.8 (399445.5-520380.7) | 4279.7 (3264-5471.8) | 1078125.9 (985722.2-1156794.7) | 3411.2 (2865.7-3956.2) | -0.37 (-2.84-2.15) |
| Acute hepatitis A | Federated States of Micronesia | 2092.8 (1891.2-2303.3) | 1534.4 (1401.3-1669.7) | 1513.1 (1388.5-1633.6) | 1425.6 (1310.2-1540.3) | -0.67 (-2.87-1.57) |
| Acute hepatitis A | Federative Republic of Brazil | 3733788.1 (3525910.3-3931311.1) | 2166.9 (2037.8-2289.6) | 3680443.1 (3460999.4-3896130.3) | 1975.4 (1839.5-2108.6) | -1.04 (-3.34-1.31) |
| Acute hepatitis A | French Republic | 653980.6 (607153.2-701411.5) | 1316.4 (1207.1-1423) | 606817.7 (562757.4-647698.1) | 1157.5 (1051.9-1257) | -0.51 (-3.01-2.05) |
| Acute hepatitis A | Gabonese Republic | 35494.9 (33362.6-37713.3) | 2523.5 (2365.4-2680.6) | 51024.8 (48143.6-53823.6) | 2382.7 (2250.3-2516.5) | -0.6 (-2.52-1.36) |
| Acute hepatitis A | Georgia | 115726.8 (109535.1-121442.5) | 2371.6 (2235.2-2499.8) | 60210.6 (56739.5-63937.7) | 2285.9 (2140.3-2431.5) | -0.46 (-2.44-1.56) |
| Acute hepatitis A | Grand Duchy of Luxembourg | 3330.4 (3080.1-3587.9) | 1010.3 (919.5-1105.9) | 5167.3 (4750.8-5551) | 956.4 (862.6-1046.6) | -0.68 (-2.91-1.6) |
| Acute hepatitis A | Greenland | 854.7 (786.2-925.7) | 1510.3 (1382.5-1643.2) | 714.4 (660.3-774.2) | 1482.9 (1354.8-1623.8) | -0.28 (-2.74-2.25) |
| Acute hepatitis A | Grenada | 2639.1 (2464.4-2809.1) | 2379.9 (2238.2-2529.1) | 1862.1 (1757.3-1963.6) | 2223.1 (2068.9-2363) | -0.81 (-3.1-1.53) |
| Acute hepatitis A | Guam | 2916.8 (2684.6-3164.9) | 1889 (1737.2-2042) | 2602.1 (2394.9-2792.4) | 1934.1 (1774.5-2082.4) | -0.46 (-2.98-2.12) |
| Acute hepatitis A | Hashemite Kingdom of Jordan | 134192 (124114.9-143547.1) | 2418.5 (2214.4-2614.2) | 292506.7 (277253.6-308697) | 2363 (2230.6-2510.2) | -0.82 (-3.26-1.67) |
| Acute hepatitis A | Hellenic Republic | 116664.5 (107881.1-126081.7) | 1365.4 (1247.8-1496.3) | 93332.8 (87221.9-99443.3) | 1301.1 (1181.1-1424.4) | -0.45 (-3.13-2.3) |
| Acute hepatitis A | Hungary | 111056.7 (102646.8-119637.3) | 1331.1 (1217.5-1443.4) | 87324.4 (80972.9-93201.8) | 1352.6 (1234.2-1468.5) | -0.19 (-2.35-2.02) |
| Acute hepatitis A | Independent State of Papua New Guinea | 126976.5 (117820.2-136753.9) | 2318.1 (2152.7-2498.5) | 318609.3 (296805.2-341570.2) | 2357.1 (2199-2515.4) | -0.42 (-2.85-2.06) |
| Acute hepatitis A | Independent State of Samoa | 4352.4 (3983.8-4746.7) | 1903.8 (1758.1-2060.5) | 5061.6 (4575.4-5524.8) | 1937.3 (1769.2-2098) | -0.26 (-2.65-2.2) |
| Acute hepatitis A | Ireland | 42015.1 (38352.1-45565.5) | 1223.1 (1116.2-1330.1) | 46106.4 (42420.5-49845.5) | 1094.5 (994.2-1196.8) | -0.43 (-2.8-2) |
| Acute hepatitis A | Islamic Republic of Afghanistan | 958166 (754022.6-1192988.8) | 8637.2 (6073.1-11695.7) | 1496674.6 (1348961.4-1645152) | 3807.7 (3195.1-4565.7) | -1.33 (-4.18-1.6) |
| Acute hepatitis A | Islamic Republic of Iran | 1827530 (1720126.1-1934770.4) | 2269.7 (2126.2-2434.2) | 1538844.1 (1451306.6-1615445.5) | 2118.4 (1989.3-2243.6) | -1.14 (-3.65-1.44) |
| Acute hepatitis A | Islamic Republic of Mauritania | 115817.4 (97412.9-139742.9) | 5190.4 (3772.5-7249.5) | 158381.7 (148805.3-168731.8) | 2678.5 (2456.6-2908.1) | -1.08 (-3.72-1.64) |
| Acute hepatitis A | Islamic Republic of Pakistan | 6317544 (5120783.4-7853414.8) | 4657 (3281.5-6533.8) | 7510813.8 (6649905.4-8418792.9) | 2721.3 (2348.1-3149.7) | -0.99 (-3.17-1.25) |
| Acute hepatitis A | Jamaica | 63486.8 (59506.5-67895.2) | 2275.9 (2134.1-2437.5) | 49165 (46328-52009.8) | 2167.3 (2014.9-2325.7) | -0.58 (-2.94-1.84) |
| Acute hepatitis A | Japan | 1035539.6 (947341.6-1113129.1) | 969 (875.3-1054.7) | 947717.6 (886541.8-1011919.8) | 1122.3 (1019.7-1225) | -0.62 (-2.67-1.47) |
| Acute hepatitis A | Kingdom of Bahrain | 13583.4 (12685.4-14604.5) | 2332.9 (2177.1-2512.7) | 26946 (25447.1-28335.8) | 2259.3 (2125.2-2394.2) | -0.57 (-2.98-1.9) |
| Acute hepatitis A | Kingdom of Belgium | 95873 (89650.8-101851.6) | 1134.7 (1049-1216.6) | 100995.5 (93952.3-108082.1) | 1110.3 (1016.2-1210.5) | -0.38 (-2.58-1.87) |
| Acute hepatitis A | Kingdom of Bhutan | 28995.3 (24743.5-34256.2) | 3634 (2900.1-4623) | 15749.8 (14462.3-16952.5) | 2371.8 (2204.4-2543.9) | -0.7 (-3.16-1.82) |
| Acute hepatitis A | Kingdom of Cambodia | 654099.1 (556021-777224.4) | 6401.1 (4820.7-8412.9) | 405837.3 (371702.6-440789.5) | 2296.3 (2097.2-2505.1) | -1.93 (-4.29-0.49) |
| Acute hepatitis A | Kingdom of Denmark | 34333.7 (31499-36865.9) | 755.3 (681.5-819.1) | 37957.2 (35086.5-40988.2) | 750 (678.6-826.3) | -0.05 (-2.19-2.13) |
| Acute hepatitis A | Kingdom of Eswatini | 35598.5 (32771.8-38494.1) | 2752.4 (2472.6-3037.6) | 36444.3 (33767.5-38969.2) | 2615 (2420.6-2801.9) | -0.63 (-3.01-1.8) |
| Acute hepatitis A | Kingdom of Lesotho | 61659.9 (56628.9-66929.9) | 2740.9 (2460.6-3050.5) | 55575.6 (50565.1-60052.4) | 2684.9 (2438.8-2918.8) | -0.38 (-2.78-2.08) |
| Acute hepatitis A | Kingdom of Morocco | 841737.4 (777453.5-907682.5) | 2580.9 (2333.9-2845) | 810348.6 (762498.8-859455.6) | 2346.5 (2202.9-2496.5) | -0.53 (-2.93-1.92) |
| Acute hepatitis A | Kingdom of Norway | 38376.8 (35314-41472.2) | 1043.4 (946.2-1135.7) | 45939 (42486-49491.8) | 1027.6 (926.5-1121.6) | -0.57 (-3.02-1.95) |
| Acute hepatitis A | Kingdom of Saudi Arabia | 568567.2 (518364.1-619767.4) | 3022.9 (2576.8-3503.4) | 647668.7 (611167.6-681363) | 2023.3 (1871.7-2163.8) | -1.12 (-3.09-0.89) |
| Acute hepatitis A | Kingdom of Spain | 377894.4 (362682.5-395761) | 1116.6 (1058.9-1177.3) | 388456 (362565.5-413421.3) | 1144.3 (1042-1252.3) | -0.54 (-2.94-1.93) |
| Acute hepatitis A | Kingdom of Sweden | 56735.5 (51806.5-61713.6) | 742.2 (667-820.3) | 61611.5 (56292.2-66804.6) | 669.3 (597.8-738.8) | -0.08 (-2.33-2.23) |
| Acute hepatitis A | Kingdom of Thailand | 831930.5 (773391.7-893044.3) | 1373.7 (1267.9-1483.9) | 715526.4 (662608.5-774534.4) | 1690.9 (1541.3-1859.1) | -0.43 (-2.66-1.84) |
| Acute hepatitis A | Kingdom of the Netherlands | 128632.5 (118801.5-138689.2) | 974.3 (887.3-1066.5) | 133834 (124366.7-144182.6) | 954.3 (869.6-1050.1) | -0.23 (-2.69-2.28) |
| Acute hepatitis A | Kingdom of Tonga | 3624.7 (3265.9-4016.3) | 3277.3 (2814.3-3761.7) | 2757.4 (2523.5-2988.1) | 2206.2 (2033.4-2380.7) | -1.07 (-3.54-1.46) |
| Acute hepatitis A | Kyrgyz Republic | 149392.2 (140374.7-158574.5) | 2667.9 (2506.5-2848) | 183376 (172041.2-194163) | 2381 (2239-2517.1) | -0.41 (-2.34-1.57) |
| Acute hepatitis A | Lao People's Democratic Republic | 150002.9 (136176.9-166201.9) | 2864.5 (2515.2-3286.7) | 162256.4 (149567.1-175738.4) | 1995.2 (1835.7-2162.7) | -1.02 (-3.31-1.33) |
| Acute hepatitis A | Lebanese Republic | 87360.6 (81162.6-94194.1) | 2426.9 (2251.7-2625.5) | 104096.3 (98643.3-110129.9) | 2220.9 (2084.4-2359.4) | -0.49 (-3.12-2.22) |
| Acute hepatitis A | Malaysia | 449480.9 (414689.2-483719.9) | 2023.9 (1873.6-2169.7) | 527274.9 (487703.3-568643.9) | 1815.9 (1660.3-1973.6) | -0.71 (-2.81-1.44) |
| Acute hepatitis A | Mongolia | 76899.5 (72419.3-81473.4) | 2506.4 (2365.1-2647.7) | 88575 (83154.7-94230.3) | 2394.7 (2253.5-2542.2) | -0.49 (-2.47-1.54) |
| Acute hepatitis A | Montenegro | 11063.3 (10143.9-11880.9) | 1899.9 (1733.8-2049.5) | 8159.7 (7549.4-8766.8) | 1823.5 (1666.9-1982.2) | -0.41 (-2.84-2.09) |
| Acute hepatitis A | New Zealand | 31893.9 (29259.9-34698.4) | 991.6 (905.5-1082.1) | 46819.8 (42877.8-50692.1) | 1074.8 (969.4-1178.5) | -0.45 (-2.63-1.77) |
| Acute hepatitis A | North Macedonia | 38310.1 (35276.1-40991.3) | 2041.6 (1869.9-2196) | 26352.5 (24450.3-28147.5) | 1810.3 (1651.3-1967.5) | -0.49 (-2.9-1.97) |
| Acute hepatitis A | Northern Mariana Islands | 911.7 (847.6-980) | 1876.1 (1727.9-2025.6) | 769.3 (709.8-824.8) | 1942.1 (1782.6-2097.8) | -0.4 (-2.94-2.21) |
| Acute hepatitis A | Palestine | 81039.3 (75432.2-87142.6) | 2408.5 (2220.6-2591) | 154017.8 (144364.6-163696.4) | 2482.5 (2318.9-2645.3) | -0.54 (-2.92-1.89) |
| Acute hepatitis A | People's Democratic Republic of Algeria | 842393.2 (782874.8-901929) | 2369 (2193.2-2542.1) | 1071620.5 (1006605.6-1131219.2) | 2337.3 (2196.8-2465.9) | -0.39 (-2.8-2.09) |
| Acute hepatitis A | People's Republic of Bangladesh | 6629163.5 (4937275.7-8469269.7) | 5685.5 (3702.3-8199.3) | 4104339.8 (3706237.9-4470238.8) | 2610.8 (2368.9-2842.5) | -1.28 (-3.86-1.38) |
| Acute hepatitis A | People's Republic of China | 30531305.7 (28419939.1-32850029.9) | 2613.6 (2419.9-2829.6) | 19209160.8 (17977606.4-20381836.6) | 1903.6 (1758.4-2046.4) | -1.62 (-4.05-0.88) |
| Acute hepatitis A | Plurinational State of Bolivia | 230083.2 (214938.7-245202.5) | 2452.1 (2282.4-2616.8) | 298557.1 (282857.2-314825.1) | 2448.6 (2316.2-2583.6) | -0.28 (-2.74-2.24) |
| Acute hepatitis A | Portuguese Republic | 124229 (114896.8-133789.2) | 1457.7 (1330.9-1583.3) | 93731.9 (87375-100060.1) | 1244.6 (1129.2-1363.1) | -0.63 (-2.98-1.78) |
| Acute hepatitis A | Principality of Andorra | 478.9 (439.3-520.2) | 1004.6 (910.1-1105.8) | 638.1 (594.8-678.9) | 955.2 (862.8-1045.3) | -0.55 (-2.75-1.7) |
| Acute hepatitis A | Principality of Monaco | 229 (214.3-245.6) | 1003.9 (910.4-1102.6) | 275.2 (256.7-293.4) | 954.2 (858.3-1048.9) | -0.53 (-2.8-1.79) |
| Acute hepatitis A | Puerto Rico | 77635 (72994.8-81989.9) | 2244.1 (2101.2-2379.6) | 39877.5 (37874-41834.1) | 2160.4 (2008.4-2310.1) | -0.81 (-3.15-1.59) |
| Acute hepatitis A | Republic of Albania | 93925.8 (88172.1-100150.9) | 2421.4 (2276.4-2582.1) | 41990.1 (39892-44386.2) | 2403.1 (2257.3-2569.5) | -0.69 (-3.21-1.89) |
| Acute hepatitis A | Republic of Angola | 688693.1 (645832.2-733203.9) | 4111 (3836-4395.3) | 1230483.2 (1153740.9-1309824.5) | 2449.7 (2310-2602.1) | -0.92 (-3.1-1.3) |
| Acute hepatitis A | Republic of Armenia | 82218 (76404.8-87854.8) | 2234.8 (2082.3-2385.7) | 46578.9 (43695.9-49448.7) | 2100.8 (1949-2252.1) | -0.36 (-2.33-1.64) |
| Acute hepatitis A | Republic of Austria | 72954.1 (67297.3-78499.8) | 1115.4 (1013.9-1215.7) | 76296.9 (70918.9-81596) | 1087.5 (989.3-1190) | -0.6 (-2.91-1.77) |
| Acute hepatitis A | Republic of Azerbaijan | 212511.2 (201386-224540.1) | 2471.6 (2346.2-2613.4) | 199028 (188069.6-209952.4) | 2342.7 (2197.6-2491.8) | -0.35 (-2.3-1.64) |
| Acute hepatitis A | Republic of Belarus | 199149.2 (187470.9-210266.7) | 2291.3 (2145.3-2430.6) | 120618.3 (112202.6-128524.8) | 1970.5 (1808.2-2126.8) | -0.72 (-3.05-1.67) |
| Acute hepatitis A | Republic of Benin | 234136.5 (215891.3-252424.4) | 3214.8 (2753.8-3772) | 531149.9 (499005-565961.3) | 2595.7 (2421.3-2785.1) | -0.63 (-2.94-1.73) |
| Acute hepatitis A | Republic of Botswana | 52944.6 (48533.5-56734.1) | 2679 (2422.3-2917) | 62367.3 (57856.8-66772.3) | 2574.6 (2395.1-2755.3) | -0.77 (-3.1-1.61) |
| Acute hepatitis A | Republic of Bulgaria | 144627.7 (135973.4-153434.2) | 2278 (2118.1-2436.3) | 89255.7 (84497.5-94262.2) | 2366.9 (2204.3-2518.7) | -0.72 (-3.28-1.9) |
| Acute hepatitis A | Republic of Burundi | 261847 (243053.5-281759.5) | 2892.2 (2560.9-3257.2) | 533766.5 (501385.7-567748.5) | 2703.7 (2510-2896) | -0.45 (-2.42-1.55) |
| Acute hepatitis A | Republic of Cabo Verde | 13915.5 (12992.9-14855.3) | 2615.4 (2404.8-2828.1) | 12309.4 (11633.6-13042.1) | 2456.7 (2323.3-2606.5) | -0.73 (-3.06-1.65) |
| Acute hepatitis A | Republic of Cameroon | 457756.8 (423924.3-492774.3) | 2666.7 (2407.9-2928) | 1145521 (1079210.8-1215739) | 2530.2 (2378-2699.3) | -0.55 (-2.75-1.7) |
| Acute hepatitis A | Republic of Chad | 316005.1 (284819.2-350210.3) | 3820.3 (3050.9-4723.6) | 847395.2 (785638.3-915059.5) | 3153.4 (2742.8-3648.9) | -0.45 (-2.87-2.02) |
| Acute hepatitis A | Republic of Chile | 355209.2 (335785.1-374568.7) | 2499.2 (2365.8-2634.1) | 309453.3 (289772.4-327891.4) | 2152.2 (1987.7-2308.6) | -0.38 (-3.01-2.32) |
| Acute hepatitis A | Republic of Colombia | 971064.5 (910983.7-1032847.3) | 2404.6 (2260.7-2550) | 920538.5 (870311.6-973592.9) | 2266.9 (2115.8-2419.4) | -0.92 (-3.39-1.62) |
| Acute hepatitis A | Republic of Costa Rica | 91047.2 (85574-96513.7) | 2380 (2241.9-2520.1) | 89190.3 (84663.4-93826.2) | 2390.9 (2250-2540.2) | -0.59 (-3.06-1.95) |
| Acute hepatitis A | Republic of C么te d'Ivoire | 552783.2 (512195-599101.4) | 3108.1 (2685.9-3608.4) | 1003168.8 (944002.3-1067629.3) | 2551.2 (2395.2-2728.5) | -0.66 (-3-1.74) |
| Acute hepatitis A | Republic of Croatia | 64971.5 (59901.4-69849.1) | 1681.1 (1531.2-1825.3) | 41535.9 (38245.7-44602.4) | 1566.4 (1423.9-1708) | -0.78 (-3.09-1.6) |
| Acute hepatitis A | Republic of Cuba | 228342.6 (215966.2-241793.6) | 2328.9 (2188-2473.5) | 158577.7 (149593.3-167920.7) | 2207 (2054.4-2364.1) | -0.82 (-3.26-1.67) |
| Acute hepatitis A | Republic of Cyprus | 7123.9 (6429.9-7859.3) | 951.8 (856-1052.6) | 10621.4 (9737.8-11530.7) | 907.6 (814.1-1002.8) | -0.74 (-3.01-1.58) |
| Acute hepatitis A | Republic of Djibouti | 16594.9 (15186-17782.6) | 2771.1 (2502.6-3045.3) | 38204.6 (35698-40619.2) | 2674.3 (2487.8-2857.6) | -0.35 (-2.55-1.89) |
| Acute hepatitis A | Republic of Ecuador | 298095.7 (280391.2-315627.4) | 2308.9 (2173.5-2439.9) | 428177.4 (405635.2-451836.5) | 2440.1 (2305.5-2583.6) | -0.17 (-2.7-2.42) |
| Acute hepatitis A | Republic of El Salvador | 185029.4 (172795.2-197646.8) | 2519.8 (2349.5-2696.2) | 156394.4 (147772.5-164627.2) | 2475.5 (2331.7-2615.8) | -0.75 (-3.18-1.75) |
| Acute hepatitis A | Republic of Equatorial Guinea | 17934.7 (16808.1-19123) | 2524.6 (2367-2682) | 46337.8 (43790.9-48969.7) | 2398 (2262.9-2541.7) | -0.63 (-2.56-1.34) |
| Acute hepatitis A | Republic of Estonia | 24848.2 (23058.3-26607.8) | 1905.1 (1757.5-2051.9) | 15292.7 (14131.6-16385.6) | 1767.4 (1613.5-1910.2) | -0.63 (-2.86-1.66) |
| Acute hepatitis A | Republic of Fiji | 18985.1 (17531.3-20396.4) | 2049.7 (1895.5-2201.5) | 18863.7 (17450.8-20246.5) | 2013.5 (1864.3-2163.3) | -0.63 (-2.99-1.79) |
| Acute hepatitis A | Republic of Finland | 31403.5 (28791.6-34203.4) | 682.4 (618.2-749.3) | 29380.5 (27118.8-31541.1) | 601.3 (544.7-657.6) | -0.08 (-2.13-2.02) |
| Acute hepatitis A | Republic of Ghana | 712727.9 (633651.6-807148.7) | 3847.6 (3100.8-4884.6) | 1127765.2 (1058682.3-1201446) | 2610.6 (2424.1-2810.9) | -1.01 (-3.4-1.45) |
| Acute hepatitis A | Republic of Guatemala | 361587.7 (336323.3-387928.8) | 2572.4 (2374.3-2766.1) | 434281.6 (401923.5-464896.6) | 2651.3 (2459.3-2845.8) | -0.59 (-2.92-1.79) |
| Acute hepatitis A | Republic of Guinea | 308079.9 (272966.5-348239.8) | 3932.2 (3127.4-5007.5) | 535022.9 (502498.4-573810.6) | 2839.6 (2576-3180.4) | -0.63 (-3.05-1.86) |
| Acute hepatitis A | Republic of Guinea-Bissau | 53641.9 (47241.5-61196.1) | 4555.3 (3434.2-5984.7) | 81250.3 (75798.2-87027.2) | 2948.7 (2634.7-3317.1) | -0.87 (-3.49-1.83) |
| Acute hepatitis A | Republic of Guyana | 25401.8 (23877.5-27032.2) | 2479.8 (2334-2623.6) | 17758.3 (16566.6-18916.9) | 2321.6 (2160.1-2478) | -0.6 (-2.8-1.65) |
| Acute hepatitis A | Republic of Haiti | 232772.4 (218233.6-249240.7) | 2504.3 (2351.8-2654.6) | 373308.2 (350751.1-398230.3) | 2475.4 (2333.9-2631.7) | -0.33 (-2.47-1.85) |
| Acute hepatitis A | Republic of Honduras | 174041 (162192.2-186608.3) | 2431.8 (2292.5-2582.4) | 260818.4 (243395.7-277226.4) | 2319.6 (2162.4-2471.3) | -0.68 (-2.98-1.67) |
| Acute hepatitis A | Republic of Iceland | 1523.3 (1385.4-1668) | 621.8 (565.1-682) | 2037.1 (1862.3-2224.8) | 631.5 (569-699) | -0.07 (-2.17-2.06) |
| Acute hepatitis A | Republic of India | 45822160.7 (37350045-56237987.2) | 4439.9 (3411.1-5722.3) | 30657172.1 (27705540.1-33374280) | 2455.8 (2249.2-2657.3) | -1.13 (-3.66-1.46) |
| Acute hepatitis A | Republic of Indonesia | 7129620.5 (6297251.2-8135601.7) | 3631.5 (3048-4350.1) | 5714328.8 (5262247.5-6201676.6) | 2264.9 (2094.8-2438.6) | -0.97 (-3.27-1.39) |
| Acute hepatitis A | Republic of Iraq | 724011.6 (664079.2-784459.3) | 2736.5 (2398-3089.7) | 1085878.5 (1026282.3-1147968.3) | 2407.1 (2264.5-2554.6) | -0.4 (-2.74-2) |
| Acute hepatitis A | Republic of Italy | 579359.1 (543350.4-615444) | 1253 (1160.6-1353) | 568715 (532650-601713.8) | 1435.1 (1318.4-1557) | -1.34 (-3.48-0.85) |
| Acute hepatitis A | Republic of Kazakhstan | 430538.8 (404939.9-458728.6) | 2380 (2240.8-2531.1) | 404974.3 (374250.2-433672.3) | 2150.6 (1987.9-2296.8) | -0.34 (-2.44-1.81) |
| Acute hepatitis A | Republic of Kenya | 1033999.6 (947307.9-1131889.3) | 2800.6 (2414.9-3226.1) | 1549351.8 (1417166.1-1680084.8) | 2608.7 (2365.5-2855.8) | -0.62 (-2.84-1.64) |
| Acute hepatitis A | Republic of Kiribati | 3333.1 (3034.8-3620.5) | 3915.3 (3431.3-4432.3) | 3349.8 (3111.2-3593.4) | 2488.1 (2308-2694.8) | -0.75 (-3.32-1.88) |
| Acute hepatitis A | Republic of Korea | 720402.3 (667316-772932.1) | 1696 (1566.8-1827.1) | 501741.4 (467588.3-534457.1) | 1547.5 (1402.1-1687.8) | -0.92 (-3.45-1.68) |
| Acute hepatitis A | Republic of Latvia | 41593.7 (38282.5-44894.9) | 1915 (1752.2-2076.6) | 21683.6 (20001.7-23212.2) | 1834.1 (1677.4-1987.3) | -0.58 (-2.95-1.84) |
| Acute hepatitis A | Republic of Liberia | 110828.1 (101559-120485.7) | 3135.4 (2682.6-3683.3) | 190510.2 (177711.5-204244.4) | 2696.5 (2468.1-2926.3) | -0.56 (-3.01-1.95) |
| Acute hepatitis A | Republic of Lithuania | 60229.2 (55816.5-64736.5) | 1915.8 (1761.2-2071) | 31061.5 (28907.6-33283.5) | 1829.8 (1674.2-1984.5) | -0.6 (-2.85-1.7) |
| Acute hepatitis A | Republic of Madagascar | 544106.1 (500711.1-586454) | 2865.9 (2533.4-3214.3) | 1049838.1 (984249.9-1126975.2) | 2674.9 (2481.2-2878.4) | -0.48 (-2.83-1.93) |
| Acute hepatitis A | Republic of Malawi | 486965.8 (446508.2-529344.5) | 3095.7 (2637.4-3586.4) | 714376 (667674.7-761170.9) | 2721.5 (2508.4-2916.8) | -0.75 (-2.93-1.48) |
| Acute hepatitis A | Republic of Maldives | 7370.5 (6745.6-8038.6) | 2190.5 (2022.6-2363) | 7987 (7444.2-8528.1) | 1871 (1717.4-2026.6) | -1.05 (-3.11-1.06) |
| Acute hepatitis A | Republic of Mali | 389718.2 (363917-416228.8) | 2661.5 (2438.5-2877.4) | 1011784 (950445.6-1080450.8) | 2559.6 (2398.2-2734) | -0.51 (-2.71-1.75) |
| Acute hepatitis A | Republic of Malta | 4335.2 (3977-4699.4) | 1279.9 (1167.4-1398.1) | 4037.7 (3756.4-4344) | 1219.2 (1108.4-1341.8) | -0.61 (-3.06-1.9) |
| Acute hepatitis A | Republic of Mauritius | 23972.4 (22256.1-25498.2) | 2089.7 (1932-2237.7) | 15853.2 (14659.3-17029.2) | 1804.3 (1638.8-1961.2) | -0.8 (-2.88-1.32) |
| Acute hepatitis A | Republic of Moldova | 92472 (85872.7-99429.5) | 2139.4 (1983-2302.4) | 44700.4 (41918.4-47252.5) | 2013.2 (1860.6-2169.4) | -0.7 (-2.73-1.37) |
| Acute hepatitis A | Republic of Mozambique | 624271 (569744.7-675432.9) | 3000.2 (2607.8-3432) | 1286217.2 (1204594.1-1364620) | 2688.2 (2493.5-2868.4) | -0.55 (-2.94-1.89) |
| Acute hepatitis A | Republic of Namibia | 55757 (51675.7-59681.1) | 2657.3 (2425.6-2897.1) | 72292.5 (67366.9-77159.3) | 2579.1 (2400.1-2752.4) | -0.47 (-2.9-2.02) |
| Acute hepatitis A | Republic of Nauru | 276.1 (252.5-298.9) | 2006.1 (1857.8-2149.3) | 266.8 (244-289.4) | 1996.8 (1835.2-2159.6) | -0.64 (-3-1.78) |
| Acute hepatitis A | Republic of Nicaragua | 155667.4 (144477.9-166626.1) | 2513 (2312-2696.6) | 169654 (160850.8-179245.7) | 2480.8 (2346.8-2629.9) | -0.65 (-3.07-1.82) |
| Acute hepatitis A | Republic of Niue | 49.7 (45.3-53.6) | 1938.5 (1775.5-2089.3) | 27 (25.1-28.9) | 1962.5 (1810.5-2111.8) | -0.83 (-3.2-1.6) |
| Acute hepatitis A | Republic of Palau | 343.8 (318.9-369.5) | 2183.4 (2001.6-2369.7) | 267.6 (246.5-287.7) | 2029.8 (1868.7-2184.3) | -0.63 (-3.04-1.83) |
| Acute hepatitis A | Republic of Panama | 70495.4 (65810.1-75214) | 2479.5 (2307.1-2648.8) | 97776.7 (92392.4-103013.7) | 2452.1 (2307.3-2595.8) | -0.52 (-3.1-2.12) |
| Acute hepatitis A | Republic of Paraguay | 122234.3 (114047.8-130967.3) | 2220.4 (2076.4-2365.8) | 138962.3 (128771.8-148513.1) | 1937.8 (1789.2-2079) | -0.59 (-3.01-1.89) |
| Acute hepatitis A | Republic of Peru | 682717.8 (634683.5-725108.2) | 2410.5 (2233.9-2561.5) | 834114.5 (787336.2-876942.7) | 2427.4 (2292.5-2554.6) | -0.42 (-2.96-2.19) |
| Acute hepatitis A | Republic of Poland | 619703.4 (572667.4-665879.4) | 1823.3 (1675.7-1962.7) | 414213.5 (385088-443139.2) | 1625.8 (1487.9-1762.2) | -1.21 (-3.51-1.14) |
| Acute hepatitis A | Republic of Rwanda | 369884.9 (332387.9-412063.3) | 3636 (2971.8-4513.9) | 456998.1 (425940.4-487683.4) | 2723.8 (2522.6-2928.7) | -1.15 (-3.3-1.05) |
| Acute hepatitis A | Republic of San Marino | 203.6 (188.7-220.4) | 1014.7 (922.5-1107.6) | 241.3 (223.9-258) | 957.6 (865.9-1048.5) | -0.37 (-2.77-2.08) |
| Acute hepatitis A | Republic of Senegal | 347307.2 (319725.6-373989.7) | 2766.5 (2467.3-3075) | 551551.3 (515452.4-587239.7) | 2571.5 (2387.6-2749.1) | -0.51 (-3.03-2.07) |
| Acute hepatitis A | Republic of Serbia | 177238.4 (166635.1-188170.2) | 2262.1 (2110.9-2422.1) | 118215.9 (111978.7-124598.2) | 2226.7 (2069.6-2378.2) | -0.43 (-2.94-2.15) |
| Acute hepatitis A | Republic of Seychelles | 1671.8 (1524.9-1838.5) | 2143.3 (1928.7-2387.4) | 1610.3 (1480.5-1751.1) | 1817.1 (1656.8-1989.8) | -0.76 (-3-1.53) |
| Acute hepatitis A | Republic of Sierra Leone | 197177.8 (177563.8-218393.8) | 3608.2 (2928.7-4400.2) | 321140.1 (301484.3-344011.7) | 2688.6 (2487.2-2918.3) | -0.71 (-3.04-1.67) |
| Acute hepatitis A | Republic of Singapore | 33049 (30601.1-35490) | 1167.1 (1070.5-1265.2) | 53435.8 (49708.9-57388.7) | 1214 (1098-1340.2) | -0.69 (-2.76-1.43) |
| Acute hepatitis A | Republic of Slovenia | 28526.2 (26450.1-30742) | 1797.6 (1653.6-1957) | 22486.7 (20817.9-24093) | 1750.5 (1597.2-1901.2) | -0.61 (-3-1.84) |
| Acute hepatitis A | Republic of South Africa | 1164933.6 (1068989.5-1265519.8) | 2441.6 (2233.3-2659.5) | 1252263.2 (1147969.2-1360454.5) | 2427.8 (2244.6-2618.3) | -0.64 (-2.9-1.66) |
| Acute hepatitis A | Republic of South Sudan | 259538.2 (238038.4-280146.7) | 2864.8 (2553.2-3191) | 407565.1 (374331.4-437265) | 2851 (2563.5-3122.1) | -0.18 (-2.4-2.08) |
| Acute hepatitis A | Republic of Sudan | 1274509.5 (1113897.8-1469604.4) | 5311.5 (4053-6747.9) | 1434386.2 (1333381-1533393.5) | 2711.9 (2487.2-2934.3) | -1.25 (-3.87-1.44) |
| Acute hepatitis A | Republic of Suriname | 10728.1 (10137.1-11302.1) | 2409.3 (2275.4-2544) | 11567.5 (10877.7-12257.2) | 2283 (2134.7-2434.3) | -0.59 (-2.86-1.75) |
| Acute hepatitis A | Republic of Tajikistan | 206188.8 (193799.4-219841.8) | 2529.6 (2385.5-2679.2) | 312650 (295592.9-331278.7) | 2505.3 (2383.4-2645.1) | -0.33 (-2.25-1.62) |
| Acute hepatitis A | Republic of the Congo | 96579.7 (90498.2-103243) | 2840.1 (2600.6-3109.8) | 156601.9 (148227.6-165236.8) | 2454.5 (2321.7-2594.4) | -0.62 (-2.68-1.48) |
| Acute hepatitis A | Republic of the Gambia | 42240.8 (39492.9-45066.8) | 2650.5 (2437.1-2869.9) | 84225.3 (79560.4-89024.8) | 2510 (2355.5-2652.8) | -0.72 (-3.04-1.66) |
| Acute hepatitis A | Republic of the Marshall Islands | 1742.3 (1643.5-1849) | 2528.9 (2366.5-2713.8) | 1448.6 (1368.6-1539.7) | 2432.5 (2290.5-2596) | -0.53 (-2.98-1.98) |
| Acute hepatitis A | Republic of the Niger | 422141.5 (385037.1-462910.9) | 3719.5 (2985.7-4577.6) | 1152456.7 (1080588-1235091.6) | 2796.5 (2529.9-3087.9) | -0.6 (-2.99-1.86) |
| Acute hepatitis A | Republic of the Philippines | 1671976.6 (1540037.6-1809912.5) | 2082.2 (1910.4-2261.8) | 2200842.6 (2044174.8-2362854.1) | 1850.1 (1714.9-1991.2) | -0.78 (-3.19-1.7) |
| Acute hepatitis A | Republic of the Union of Myanmar | 1070595.1 (981651.5-1165643.3) | 2256.7 (2043.6-2493.1) | 1072843.9 (988159.3-1157067.1) | 1930 (1770.7-2085.4) | -0.6 (-2.65-1.49) |
| Acute hepatitis A | Republic of Trinidad and Tobago | 31353 (29484.6-33074.8) | 2309.9 (2170.3-2441.7) | 22166.2 (20819.1-23467.9) | 2146.7 (1990.7-2295) | -0.62 (-2.96-1.77) |
| Acute hepatitis A | Republic of Tunisia | 253032.1 (236055.5-271969.2) | 2427.4 (2250.3-2615.1) | 228787.4 (216871.5-241617) | 2344.2 (2211.4-2487.3) | -0.53 (-3-2) |
| Acute hepatitis A | Republic of Turkey | 1606022.2 (1500041.8-1711074.5) | 2359.8 (2195.6-2534.7) | 1420596.5 (1338169.2-1501474) | 2076.8 (1929.9-2213.6) | -0.63 (-3.22-2.04) |
| Acute hepatitis A | Republic of Uganda | 895337.7 (823650-957602.7) | 2975 (2610.8-3385.5) | 1839413 (1704409.6-1962524.8) | 2739.7 (2505.1-2958.9) | -0.7 (-2.84-1.5) |
| Acute hepatitis A | Republic of Uzbekistan | 847088 (790308.1-902771.8) | 2918.3 (2699.9-3133.8) | 907211.1 (855198.4-965561.8) | 2498.9 (2364.8-2652.1) | -0.47 (-2.53-1.64) |
| Acute hepatitis A | Republic of Vanuatu | 4790.6 (4426-5166.4) | 2192.4 (2042.9-2335.8) | 8423.4 (7758.1-9035.8) | 2163.1 (2006.1-2309.4) | -0.43 (-2.84-2.03) |
| Acute hepatitis A | Republic of Yemen | 961394.8 (850776.3-1084039.7) | 5360.6 (4156.7-6736.1) | 1114753.9 (1047323.8-1182551.2) | 2578.6 (2389.6-2769.1) | -1.27 (-4-1.55) |
| Acute hepatitis A | Republic of Zambia | 393855.2 (359018-427924.4) | 3216.9 (2763.9-3726.9) | 743518.3 (694683.6-790262.1) | 2698.5 (2497.3-2887.9) | -0.73 (-2.92-1.52) |
| Acute hepatitis A | Republic of Zimbabwe | 439270.9 (406058.3-471909.6) | 2804.2 (2521.3-3098.2) | 580771.6 (536079.2-628451.5) | 2844.7 (2567.5-3157.4) | -0.29 (-2.95-2.45) |
| Acute hepatitis A | Romania | 383240.6 (356159-410002) | 1908 (1762.7-2052.9) | 202939.2 (187490.8-217356.2) | 1618.2 (1477-1752) | -0.97 (-3.21-1.32) |
| Acute hepatitis A | Russian Federation | 2522061.3 (2356863.6-2681624.1) | 1971.3 (1829.4-2107.3) | 1975516.8 (1856397.1-2091293.5) | 2016.8 (1875.2-2150.9) | -0.38 (-2.78-2.08) |
| Acute hepatitis A | Saint Kitts and Nevis | 1102.7 (1031.3-1163.3) | 2337.2 (2187-2469.7) | 915.3 (864.9-967.7) | 2204.4 (2042.2-2362.2) | -0.84 (-3.12-1.49) |
| Acute hepatitis A | Saint Lucia | 4077.6 (3823.3-4314.1) | 2378.5 (2229.2-2516.7) | 2722.6 (2580.8-2873.3) | 2225.2 (2065.6-2379.9) | -0.7 (-2.99-1.65) |
| Acute hepatitis A | Saint Vincent and the Grenadines | 3214.7 (3043-3378) | 2414.6 (2281.6-2549.6) | 2051 (1933.8-2160.7) | 2276.6 (2118.6-2426.6) | -0.65 (-2.98-1.73) |
| Acute hepatitis A | Slovak Republic | 86355.6 (79499.8-93472.1) | 1826.2 (1665.8-1985.5) | 64147.9 (59170.6-68753.1) | 1761.2 (1600.9-1915.8) | -0.6 (-2.96-1.82) |
| Acute hepatitis A | Socialist Republic of Viet Nam | 2287698.9 (2102632.4-2499906) | 2819.8 (2512.9-3178.5) | 1818859.3 (1675783.3-1950576.3) | 2057.3 (1882.5-2209.1) | -0.91 (-3.41-1.65) |
| Acute hepatitis A | Solomon Islands | 11172.4 (10299.3-12035.3) | 2373.7 (2186.3-2565.8) | 18599.3 (17165.9-20035) | 2152.6 (2000.8-2305.3) | -0.63 (-3.09-1.89) |
| Acute hepatitis A | State of Eritrea | 165030 (149278.7-180900.1) | 3251.5 (2733.8-3801.7) | 239713.2 (221138.5-256633) | 2811.3 (2548.5-3054.6) | -0.64 (-2.84-1.61) |
| Acute hepatitis A | State of Israel | 60905.8 (55480.4-67142.5) | 1201.2 (1096.9-1320) | 102788.7 (93332-111343.5) | 1109.2 (1006.6-1203.5) | -0.72 (-3.2-1.81) |
| Acute hepatitis A | State of Kuwait | 44549.7 (41620.9-47254.5) | 2255.8 (2107.9-2394.3) | 76739.6 (72397.3-81139) | 2241.3 (2095.3-2376) | -0.58 (-3.02-1.92) |
| Acute hepatitis A | State of Libya | 142488.7 (132797.7-152103.7) | 2441.6 (2246.6-2650.1) | 126346.9 (119487.1-132752) | 2291.6 (2158.5-2432.3) | -0.57 (-3.05-1.98) |
| Acute hepatitis A | State of Qatar | 11004.9 (10203-11839.5) | 2324.7 (2164.7-2494) | 50744.4 (47698.6-53652.8) | 2250.2 (2111.3-2385.8) | -0.6 (-2.99-1.84) |
| Acute hepatitis A | Sultanate of Oman | 90235.6 (77891.7-104064.5) | 4420.1 (3310.3-5731.3) | 107578.9 (100799.8-114545.5) | 2416.1 (2260.2-2584.1) | -1.4 (-3.92-1.18) |
| Acute hepatitis A | Swiss Confederation | 49583.4 (45347.3-53348.5) | 825.7 (741.4-901.3) | 63116.6 (58139-67912.8) | 850.1 (765.2-932.4) | -0.05 (-2.17-2.13) |
| Acute hepatitis A | Syrian Arab Republic | 501076.2 (462912.5-540594) | 2663 (2384.9-2961.3) | 290702.8 (274921.7-306737.9) | 2380 (2232.3-2521.5) | -0.58 (-3.02-1.93) |
| Acute hepatitis A | Taiwan (Province of China) | 359175.7 (331836.5-387193.8) | 1813.9 (1664.1-1968.7) | 246667.7 (229385.8-264014.2) | 1681.2 (1526.4-1840.4) | -1.15 (-3.49-1.25) |
| Acute hepatitis A | Togolese Republic | 158201.3 (147476.4-168770.7) | 2638.3 (2422.3-2849) | 280749.4 (264761.6-298378.5) | 2534 (2380.2-2697.6) | -0.46 (-2.82-1.95) |
| Acute hepatitis A | Tokelau | 38.4 (35.3-41.3) | 2014 (1869.3-2157.4) | 25.2 (23.3-26.9) | 1990 (1836.5-2133.9) | -0.7 (-3.13-1.78) |
| Acute hepatitis A | Turkmenistan | 146483.5 (136774.6-157224.3) | 2831.1 (2647.2-3024.5) | 126749.5 (119544.7-135027.6) | 2364.8 (2230.6-2519.4) | -0.39 (-2.34-1.59) |
| Acute hepatitis A | Tuvalu | 248.8 (228.2-269.3) | 2070.9 (1925.3-2215.4) | 261.8 (242-280.7) | 2016.4 (1862.4-2164.6) | -0.4 (-2.83-2.08) |
| Acute hepatitis A | Ukraine | 785298 (728711.5-843895.5) | 1840 (1691-1986.7) | 481494.1 (448457.8-509850.5) | 1820.4 (1673.1-1957.7) | 0.21 (-1.85-2.31) |
| Acute hepatitis A | Union of the Comoros | 20759.2 (19158.1-22275.2) | 2838.5 (2546.8-3148.6) | 21975.7 (20344.9-23537.3) | 2689.8 (2487.7-2882) | -0.57 (-2.74-1.66) |
| Acute hepatitis A | United Arab Emirates | 48848.2 (45631.7-52144.1) | 2306.6 (2152.4-2482.3) | 131275.3 (122304.9-140147.3) | 2249.3 (2103.3-2393.6) | -0.52 (-2.92-1.95) |
| Acute hepatitis A | United Kingdom of Great Britain and Northern Ireland | 564614.1 (521204.7-607574.6) | 1143.8 (1043.3-1242) | 595967.9 (551683.2-639749.2) | 1076.2 (977.6-1173.1) | 0.07 (-2.13-2.32) |
| Acute hepatitis A | United Mexican States | 2809114.1 (2612293.9-3009474.9) | 2445.7 (2266.6-2624.5) | 2694553.3 (2548848.7-2848877.7) | 2423.9 (2290.2-2562.8) | -0.66 (-3.18-1.92) |
| Acute hepatitis A | United Republic of Tanzania | 1202575.4 (1100295.6-1295100.1) | 2924.7 (2579.1-3275.9) | 2195373.4 (2061283.9-2331187) | 2653.9 (2480.1-2831.3) | -0.55 (-2.62-1.57) |
| Acute hepatitis A | United States of America | 2753619.7 (2521823.7-2979219.8) | 1198.3 (1090.3-1305.1) | 3300515.7 (3044330.4-3537131) | 1241.4 (1126.6-1346.8) | -0.49 (-2.81-1.89) |
| Acute hepatitis A | United States Virgin Islands | 2514.5 (2359.1-2681.5) | 2289.5 (2149.3-2440.9) | 1114.4 (1049-1181.4) | 2167.8 (2009.5-2320.5) | -0.71 (-3.07-1.7) |
| Acute hepatitis B | American Samoa | 744.8 (609.5-917.9) | 1543.8 (1233.8-1927.5) | 439.8 (340.9-552.2) | 877.9 (678.9-1107.6) | -0.45 (-2.97-2.13) |
| Acute hepatitis B | Antigua and Barbuda | 205.4 (156.6-262.5) | 333.4 (255.9-424.5) | 178.5 (125.2-235) | 174.3 (123.8-228.3) | -0.73 (-3.08-1.68) |
| Acute hepatitis B | Arab Republic of Egypt | 884149.4 (672260.2-1109628.8) | 1624.8 (1214.2-2081.9) | 654093.7 (455870.7-874833.8) | 694.9 (479.7-927) | -1.35 (-3.54-0.88) |
| Acute hepatitis B | Argentine Republic | 65096.3 (53623.4-76916.8) | 198.6 (163.4-234.7) | 72557.9 (58312.3-91172.5) | 147.9 (119.2-185.4) | -0.42 (-3.45-2.7) |
| Acute hepatitis B | Australia | 113932.1 (86921.8-145310.5) | 676.2 (528.7-850.1) | 110117.5 (79790.7-149371) | 382.9 (279.4-508.4) | -0.51 (-2.65-1.68) |
| Acute hepatitis B | Barbados | 674.6 (498.7-922.2) | 263.7 (198.1-357.4) | 485.2 (332.5-665.1) | 151.8 (108.1-205.4) | -0.57 (-2.97-1.89) |
| Acute hepatitis B | Belize | 797.1 (615.1-1031.7) | 417.5 (323.1-531.2) | 1001.3 (719.9-1352.4) | 233.3 (169.6-314.4) | -0.55 (-2.82-1.78) |
| Acute hepatitis B | Bermuda | 160 (107.6-213.2) | 262.7 (190-339.9) | 111.1 (76.4-149.8) | 160.2 (114.1-212.2) | -0.63 (-3.06-1.85) |
| Acute hepatitis B | Bolivarian Republic of Venezuela | 62854.7 (46580.2-82941) | 306.7 (223-415) | 46857.3 (31484.2-68028.1) | 174.8 (118.5-247.3) | -0.51 (-3.05-2.1) |
| Acute hepatitis B | Bosnia and Herzegovina | 21616.8 (17872.5-26103.5) | 465.9 (390.4-556.3) | 11377 (9115.5-13994.8) | 301.7 (244.2-371.3) | -0.57 (-3.15-2.07) |
| Acute hepatitis B | Brunei Darussalam | 2541.5 (1883.3-3365.8) | 969.8 (720.3-1264) | 1964.2 (1417.6-2757.1) | 398.3 (286-551.4) | -0.81 (-2.87-1.29) |
| Acute hepatitis B | Burkina Faso | 240894.3 (186835-300885.8) | 2746.6 (2093.7-3522.4) | 332226.6 (256141.1-439958) | 1881.7 (1458.7-2515.8) | -0.66 (-2.95-1.69) |
| Acute hepatitis B | Canada | 144744.7 (110620.5-180551.4) | 539.3 (426.1-655.5) | 142716.6 (107709.4-188551.2) | 373.7 (291.5-479.8) | -0.24 (-2.51-2.09) |
| Acute hepatitis B | Central African Republic | 68728.1 (49732-90277.8) | 2444 (1710.1-3235.2) | 109416.9 (78976.1-140674.4) | 2029.8 (1421.2-2650.3) | -0.46 (-2.59-1.73) |
| Acute hepatitis B | Commonwealth of Dominica | 269.1 (208-348.9) | 364.4 (276.8-472.4) | 160.2 (112.8-217.6) | 223.4 (156.4-298.1) | -0.6 (-2.94-1.79) |
| Acute hepatitis B | Commonwealth of the Bahamas | 955.3 (728.8-1238.6) | 349.3 (269-447.5) | 866 (626.1-1201) | 206.5 (151.3-282.8) | -0.6 (-2.92-1.79) |
| Acute hepatitis B | Cook Islands | 218.3 (163.4-282.5) | 1158.5 (850.2-1521.9) | 98.9 (70.7-127.4) | 493.3 (365.8-636) | -0.78 (-3.18-1.68) |
| Acute hepatitis B | Czech Republic | 36996.3 (28227.8-46321) | 366.1 (284.1-451.4) | 23668 (18251.5-29332.4) | 192.9 (150-244.4) | -0.45 (-2.68-1.82) |
| Acute hepatitis B | Democratic People's Republic of Korea | 597410.5 (434037.7-784026) | 2820.2 (2054.5-3720.6) | 548272.4 (387530.4-731569.6) | 1863.6 (1364.2-2454.5) | -0.67 (-3.12-1.86) |
| Acute hepatitis B | Democratic Republic of Sao Tome and Principe | 3030.4 (2321.6-3794.4) | 2692.9 (2039.4-3396.6) | 3739.1 (2870.7-4793.3) | 1899.9 (1483.4-2415.4) | -0.69 (-3.1-1.79) |
| Acute hepatitis B | Democratic Republic of the Congo | 1208715.3 (887888.6-1533364.3) | 3124.9 (2258.2-4123.2) | 2170073.4 (1641227.7-2740047.9) | 2580.1 (1876-3294.6) | -0.35 (-2.52-1.87) |
| Acute hepatitis B | Democratic Republic of Timor-Leste | 9694 (7029.8-12693.5) | 1192.7 (836.3-1583.8) | 11781 (8468.1-15691.3) | 879.9 (627.1-1178.2) | -0.54 (-2.76-1.73) |
| Acute hepatitis B | Democratic Socialist Republic of Sri Lanka | 152679.6 (123438.7-186981) | 889.2 (724.8-1077.6) | 144160.2 (118037.1-175946.3) | 602.6 (494.3-738.6) | -0.61 (-2.68-1.5) |
| Acute hepatitis B | Dominican Republic | 35371.8 (28306-43653.3) | 495.7 (399.1-610.1) | 36713.9 (29019.3-46983.8) | 326.7 (260-417.5) | -0.49 (-2.76-1.84) |
| Acute hepatitis B | Eastern Republic of Uruguay | 5102.2 (4164.7-6125.3) | 163.5 (133.3-196.7) | 4159.8 (3261.9-5179.5) | 110.1 (86.3-136.8) | -0.48 (-3.3-2.42) |
| Acute hepatitis B | Federal Democratic Republic of Ethiopia | 924101.8 (727236.7-1149301.3) | 1823.2 (1387.3-2266) | 1344662.1 (1024274.6-1714624.3) | 1281.9 (991.3-1613.1) | -0.9 (-3.11-1.35) |
| Acute hepatitis B | Federal Democratic Republic of Nepal | 101730.1 (90296.2-117361.8) | 599.6 (531.2-687.4) | 138200.2 (118386.2-161094.9) | 462.7 (400.6-534) | -1.14 (-3.81-1.6) |
| Acute hepatitis B | Federal Republic of Germany | 125381.2 (98183.4-153950.6) | 158.7 (128.1-192.3) | 88897 (65800.7-110804.3) | 87.1 (67.3-108.3) | -0.26 (-2.49-2.02) |
| Acute hepatitis B | Federal Republic of Nigeria | 3103957.5 (2482944.4-3784282.4) | 3430.1 (2669-4224.1) | 5246098.3 (4195907.7-6510087.5) | 2415.1 (1886.4-3026) | -1.48 (-3.87-0.98) |
| Acute hepatitis B | Federal Republic of Somalia | 256469.4 (197112.7-327027.2) | 3168.7 (2353.6-4135.4) | 632465.6 (486333.1-784629.1) | 2891.5 (2127.8-3670) | -0.37 (-2.84-2.15) |
| Acute hepatitis B | Federated States of Micronesia | 1860.9 (1494-2338.7) | 1870 (1485.6-2341.1) | 960.8 (740.9-1215.5) | 974 (754.3-1237.1) | -0.67 (-2.87-1.57) |
| Acute hepatitis B | Federative Republic of Brazil | 1106162.2 (906857.1-1339219.9) | 733.4 (600.2-888.8) | 632700.1 (460661.5-827829.7) | 264 (197.6-346) | -1.04 (-3.34-1.31) |
| Acute hepatitis B | French Republic | 138939.7 (111278.5-172329) | 243.1 (195.4-294.7) | 113666 (89131.2-144597.5) | 161 (125-204.1) | -0.51 (-3.01-2.05) |
| Acute hepatitis B | Gabonese Republic | 17970.1 (13957.8-22883.3) | 1788.3 (1337.6-2344.1) | 20626 (14953.7-26446.3) | 1184.1 (849.3-1527) | -0.6 (-2.52-1.36) |
| Acute hepatitis B | Georgia | 29876.1 (23864.6-36943.9) | 536.3 (432.7-660.2) | 14161.9 (11379.1-17183.4) | 364.3 (292.6-439.4) | -0.46 (-2.44-1.56) |
| Acute hepatitis B | Grand Duchy of Luxembourg | 1089 (821.6-1410.4) | 289.6 (221.5-364.1) | 1196.6 (830.1-1614.3) | 152.7 (110.1-207) | -0.68 (-2.91-1.6) |
| Acute hepatitis B | Greenland | 276.9 (198.5-365.4) | 458.7 (333.9-597.1) | 202.4 (135.9-277) | 362.8 (253.2-491.6) | -0.28 (-2.74-2.25) |
| Acute hepatitis B | Grenada | 349.1 (261.2-452) | 396.1 (288.6-514.1) | 217.1 (142.5-308.9) | 196.9 (130.2-278.6) | -0.81 (-3.1-1.53) |
| Acute hepatitis B | Guam | 2328.4 (1921-2816) | 1666.2 (1403.8-1972.1) | 1684.5 (1374.5-2025.6) | 981.7 (812.8-1161.4) | -0.46 (-2.98-2.12) |
| Acute hepatitis B | Hashemite Kingdom of Jordan | 40009.2 (31210.4-51535.6) | 1052.6 (801.3-1389.1) | 56523.6 (41176.3-74961.9) | 484.7 (352-635.3) | -0.82 (-3.26-1.67) |
| Acute hepatitis B | Hellenic Republic | 64794.8 (50509.8-77599.7) | 617.2 (497.2-732.1) | 43273 (33107.8-57720.5) | 342.4 (270-441.6) | -0.45 (-3.13-2.3) |
| Acute hepatitis B | Hungary | 43411.6 (37589.6-50298.6) | 411.5 (356-479.2) | 38331 (33695.7-43991.6) | 396.1 (345.9-456.3) | -0.19 (-2.35-2.02) |
| Acute hepatitis B | Independent State of Papua New Guinea | 104460.8 (74210.9-135647.4) | 2455 (1728.9-3250.4) | 174570.2 (132471.2-223454.3) | 1669 (1219.2-2196.4) | -0.42 (-2.85-2.06) |
| Acute hepatitis B | Independent State of Samoa | 2917.2 (2334.5-3782.9) | 1782.2 (1411.6-2339.7) | 2395.7 (1934.1-3027.2) | 1212.9 (976.2-1552.9) | -0.26 (-2.65-2.2) |
| Acute hepatitis B | Ireland | 8105.8 (6009.7-10497.7) | 228.3 (172.3-295.4) | 7880.3 (5646.1-10599.2) | 156.8 (113.8-208.1) | -0.43 (-2.8-2) |
| Acute hepatitis B | Islamic Republic of Afghanistan | 104920.6 (85895.3-124705.2) | 1175.5 (956-1394.9) | 258277.8 (208051.5-319250.1) | 1006.3 (817.9-1218.8) | -1.33 (-4.18-1.6) |
| Acute hepatitis B | Islamic Republic of Iran | 537959.4 (452579.1-630615.6) | 1013 (857.3-1186.1) | 407543.5 (333280.7-495530.9) | 404.7 (334.6-485.2) | -1.14 (-3.65-1.44) |
| Acute hepatitis B | Islamic Republic of Mauritania | 76403.6 (59672.8-92800.9) | 3827.5 (2925.1-4764.8) | 104034 (80124.4-131701.5) | 2687 (2019.9-3440.3) | -1.08 (-3.72-1.64) |
| Acute hepatitis B | Islamic Republic of Pakistan | 1153984.8 (932260.5-1401533.8) | 1052.1 (838.8-1281.4) | 1438548.8 (1107284.5-1854705.6) | 649.7 (491.7-830.4) | -0.99 (-3.17-1.25) |
| Acute hepatitis B | Jamaica | 8057.2 (6081.2-10363) | 324.5 (246.6-413.2) | 5597 (3507.6-8167.1) | 180.8 (114.8-262.2) | -0.58 (-2.94-1.84) |
| Acute hepatitis B | Japan | 1186554.6 (1004723.6-1403448.2) | 909.7 (780.5-1058) | 799990.6 (632223.2-1008097) | 605.5 (496.8-748.5) | -0.62 (-2.67-1.47) |
| Acute hepatitis B | Kingdom of Bahrain | 5597.1 (4752.2-6708.8) | 1090.5 (941.9-1268.3) | 11037.5 (8895.6-13215.4) | 621.5 (509.2-729.9) | -0.57 (-2.98-1.9) |
| Acute hepatitis B | Kingdom of Belgium | 20210.6 (15320.7-25928.5) | 210.5 (163.3-260.9) | 14816.3 (10281.3-19601.8) | 116 (81.8-152.2) | -0.38 (-2.58-1.87) |
| Acute hepatitis B | Kingdom of Bhutan | 6885.1 (5085.5-8616) | 1093.8 (803.5-1388.6) | 4421.6 (3273.7-5720.1) | 545.3 (401.5-702.4) | -0.7 (-3.16-1.82) |
| Acute hepatitis B | Kingdom of Cambodia | 208024.8 (158713.4-265395.5) | 1940.8 (1416.1-2585.2) | 189905.3 (126299-263888.7) | 1080 (732-1479) | -1.93 (-4.29-0.49) |
| Acute hepatitis B | Kingdom of Denmark | 13566.4 (10395.6-17454) | 270.5 (213.9-338.3) | 14025.6 (10843.4-17739.6) | 261.5 (208.5-322.6) | -0.05 (-2.19-2.13) |
| Acute hepatitis B | Kingdom of Eswatini | 15679.8 (11859.3-19701.6) | 1971.1 (1490.7-2532.5) | 9615.5 (7007.9-12762.5) | 932.9 (689.4-1235.4) | -0.63 (-3.01-1.8) |
| Acute hepatitis B | Kingdom of Lesotho | 29733.2 (23781.7-37424.6) | 1977.4 (1542.5-2526.1) | 22048.2 (16295.4-28943.6) | 1204.6 (883-1581.8) | -0.38 (-2.78-2.08) |
| Acute hepatitis B | Kingdom of Morocco | 177736.1 (148009.8-213354.4) | 736.3 (611.7-874) | 179177.2 (143844.2-216277.3) | 463.3 (372.4-558.1) | -0.53 (-2.93-1.92) |
| Acute hepatitis B | Kingdom of Norway | 9071.8 (7036.3-11532.2) | 222.8 (175-278.5) | 6638.4 (4803.6-8874.4) | 114.6 (82.9-155.8) | -0.57 (-3.02-1.95) |
| Acute hepatitis B | Kingdom of Saudi Arabia | 142321.9 (113274-178485.8) | 939.5 (747.9-1175) | 195020.5 (150005.7-245002.7) | 460.4 (364-571.6) | -1.12 (-3.09-0.89) |
| Acute hepatitis B | Kingdom of Spain | 267075.5 (200404.9-339544.4) | 686.8 (532.2-856.7) | 189682.3 (136925.5-247612.2) | 315.1 (239.5-402.2) | -0.54 (-2.94-1.93) |
| Acute hepatitis B | Kingdom of Sweden | 12753.9 (9972.2-15969.1) | 157.2 (124.9-193.8) | 13474.2 (10569.9-17453.5) | 131.4 (102.2-169.6) | -0.08 (-2.33-2.23) |
| Acute hepatitis B | Kingdom of Thailand | 753345.1 (611761-907311.5) | 1287.6 (1057.9-1536.7) | 544716.7 (402070.8-693915.6) | 660.6 (500.5-833.6) | -0.43 (-2.66-1.84) |
| Acute hepatitis B | Kingdom of the Netherlands | 22019.8 (18268-26524.7) | 142.7 (119.3-168.9) | 21935.3 (18035.8-26247.1) | 115 (94.7-135.6) | -0.23 (-2.69-2.28) |
| Acute hepatitis B | Kingdom of Tonga | 1952.6 (1585-2358.2) | 2204.1 (1808.8-2636.7) | 942.2 (764-1151.2) | 1071.7 (870.8-1309.1) | -1.07 (-3.54-1.46) |
| Acute hepatitis B | Kyrgyz Republic | 32596.8 (24439.4-39790.8) | 739.9 (558.1-903) | 32663.2 (24918.5-42413.7) | 482.8 (371.6-629.3) | -0.41 (-2.34-1.57) |
| Acute hepatitis B | Lao People's Democratic Republic | 61951.7 (44156.9-81503.6) | 1445.9 (1003.2-1991) | 77276.9 (54207.8-102955.7) | 1007.1 (704.5-1355.2) | -1.02 (-3.31-1.33) |
| Acute hepatitis B | Lebanese Republic | 23156.7 (20060-26879.1) | 818.3 (711-946) | 38101.1 (31953.4-44651.9) | 616.5 (519.9-718) | -0.49 (-3.12-2.22) |
| Acute hepatitis B | Malaysia | 169932.8 (147425.8-201689.5) | 1024.1 (885.3-1211.1) | 181783.9 (152062.4-214273) | 556.9 (469.2-650.8) | -0.71 (-2.81-1.44) |
| Acute hepatitis B | Mongolia | 51737.6 (43139.2-60829.8) | 2617.5 (2209.6-3050) | 51163.2 (42653.6-61123.2) | 1544.9 (1298.8-1826.7) | -0.49 (-2.47-1.54) |
| Acute hepatitis B | Montenegro | 1875 (1452.3-2346.1) | 298.1 (234.9-369.4) | 1242.2 (927.4-1607.4) | 192.8 (147.5-242.9) | -0.41 (-2.84-2.09) |
| Acute hepatitis B | New Zealand | 23377.4 (17385.5-30112.7) | 681.9 (512.3-866.7) | 17484 (12571.7-23311.2) | 314.3 (233.2-408.8) | -0.45 (-2.63-1.77) |
| Acute hepatitis B | North Macedonia | 9273.4 (7496.8-11275.4) | 458.4 (374.6-554.2) | 7986.9 (6276.9-10163) | 314.9 (253.3-396.3) | -0.49 (-2.9-1.97) |
| Acute hepatitis B | Northern Mariana Islands | 1105.2 (929.8-1321.6) | 2343.6 (1996.6-2734.5) | 796.2 (648.5-955.4) | 1465.7 (1231.3-1742.4) | -0.4 (-2.94-2.21) |
| Acute hepatitis B | Palestine | 18830.9 (15981.8-22227.7) | 1013.7 (848.1-1197.1) | 23616.1 (19858-29247.7) | 582.7 (492.2-713.6) | -0.54 (-2.92-1.89) |
| Acute hepatitis B | People's Democratic Republic of Algeria | 204719.6 (176077.7-241310.5) | 881 (752.2-1044) | 288758.1 (240036.1-340075.4) | 649.8 (541.8-764.2) | -0.39 (-2.8-2.09) |
| Acute hepatitis B | People's Republic of Bangladesh | 1014016.4 (834395.4-1237002.1) | 1035.5 (842.1-1267.2) | 1185761.8 (957561.7-1405214.2) | 716.1 (581.8-846.5) | -1.28 (-3.86-1.38) |
| Acute hepatitis B | People's Republic of China | 29200406.7 (22793002.9-35818277.8) | 2378.2 (1865.7-2894) | 16032339.3 (11361942-21674277.2) | 978.3 (711.4-1302.2) | -1.62 (-4.05-0.88) |
| Acute hepatitis B | Plurinational State of Bolivia | 17235.8 (15492.6-19548.5) | 307.7 (276-349.3) | 26860.4 (22478.6-31287.7) | 235.5 (198.3-271.7) | -0.28 (-2.74-2.24) |
| Acute hepatitis B | Portuguese Republic | 31799.2 (24070.6-41042.4) | 325 (252.7-412.6) | 19510.5 (13211.1-26957) | 159.4 (114.8-216.3) | -0.63 (-2.98-1.78) |
| Acute hepatitis B | Principality of Andorra | 186.5 (136.4-246.2) | 328 (247-419.2) | 192.4 (133.6-270.6) | 182.9 (127-253.4) | -0.55 (-2.75-1.7) |
| Acute hepatitis B | Principality of Monaco | 75.5 (54.4-99.9) | 274.1 (204.7-342.3) | 57.6 (37.8-80.6) | 123.9 (87.7-167) | -0.53 (-2.8-1.79) |
| Acute hepatitis B | Puerto Rico | 10862.1 (8032.3-14334.7) | 296.9 (221-388.7) | 5096.6 (3660.7-6948.1) | 140.3 (107.6-180.9) | -0.81 (-3.15-1.59) |
| Acute hepatitis B | Republic of Albania | 16063 (13172.1-19208.5) | 490.9 (402.6-585.2) | 8239.7 (6607.6-10238.5) | 251.9 (202.5-318.6) | -0.69 (-3.21-1.89) |
| Acute hepatitis B | Republic of Angola | 311339.5 (212818-404065.8) | 2997.8 (1995.3-3968.8) | 694086.7 (492891.7-867270.9) | 2306.5 (1582.7-2987.4) | -0.92 (-3.1-1.3) |
| Acute hepatitis B | Republic of Armenia | 23593.8 (19459-28203.4) | 685 (567.8-819.4) | 15458.4 (12368.8-18898.2) | 440.3 (354.2-537) | -0.36 (-2.33-1.64) |
| Acute hepatitis B | Republic of Austria | 21502.2 (16006.4-27541.5) | 287.3 (223-357.3) | 16713.8 (11865.2-22186.6) | 173.3 (124.6-226.8) | -0.6 (-2.91-1.77) |
| Acute hepatitis B | Republic of Azerbaijan | 66702.4 (55440.7-79674.7) | 930.8 (781.6-1103.1) | 86731.6 (72629.7-101591.9) | 739.5 (624.2-862.3) | -0.35 (-2.3-1.64) |
| Acute hepatitis B | Republic of Belarus | 28340.4 (20827.5-38783.3) | 276.1 (205-368.4) | 12532.5 (7866.3-19312.1) | 108.9 (70.4-162.8) | -0.72 (-3.05-1.67) |
| Acute hepatitis B | Republic of Benin | 92299.8 (70032.1-113573.7) | 2023.1 (1544.8-2482.6) | 164903.2 (124660.9-218467.9) | 1506.6 (1153-1926.7) | -0.63 (-2.94-1.73) |
| Acute hepatitis B | Republic of Botswana | 23989.6 (18770.5-30503.1) | 1793.6 (1385.4-2255.5) | 20712.6 (15695.1-27103.8) | 839.4 (645.9-1088.4) | -0.77 (-3.1-1.61) |
| Acute hepatitis B | Republic of Bulgaria | 76783.5 (57393.6-98654.3) | 931 (720.8-1165.8) | 29029.5 (17854-42383.6) | 346.8 (235.7-485.6) | -0.72 (-3.28-1.9) |
| Acute hepatitis B | Republic of Burundi | 54185.9 (41833.5-68527.4) | 979 (749.1-1245.2) | 71520 (52249-93898.5) | 675.4 (501.1-873) | -0.45 (-2.42-1.55) |
| Acute hepatitis B | Republic of Cabo Verde | 6949.5 (4960-9000.1) | 1980.9 (1398.3-2586.9) | 7669.5 (5249.2-10743.8) | 1259 (863-1737.2) | -0.73 (-3.06-1.65) |
| Acute hepatitis B | Republic of Cameroon | 197902.2 (154870.6-243156.4) | 1969.2 (1514.2-2515.2) | 376585.7 (283900.1-471073) | 1396.3 (1057-1773.3) | -0.55 (-2.75-1.7) |
| Acute hepatitis B | Republic of Chad | 157364.6 (112166.9-198314.9) | 2690.4 (1925.5-3474.1) | 342678.7 (225333.3-449634.5) | 2191.5 (1469.1-2902.9) | -0.45 (-2.87-2.02) |
| Acute hepatitis B | Republic of Chile | 35984.7 (29940.9-42924.5) | 266.1 (223.8-313.7) | 43428.4 (34847.8-54631.1) | 205.7 (165.1-258.6) | -0.38 (-3.01-2.32) |
| Acute hepatitis B | Republic of Colombia | 373208.6 (276984-509896.7) | 1059 (781.9-1441) | 226177.8 (133554.9-334782.2) | 435.9 (263.8-635.3) | -0.92 (-3.39-1.62) |
| Acute hepatitis B | Republic of Costa Rica | 10298.8 (7484.8-13403.5) | 320.2 (230.9-416.8) | 8272.6 (5799.6-11480.9) | 158.7 (112.3-218.5) | -0.59 (-3.06-1.95) |
| Acute hepatitis B | Republic of C么te d'Ivoire | 265573.3 (206600.9-333962.7) | 2283.5 (1772-2906.7) | 366145.4 (257506.6-482960.1) | 1498.7 (1050.3-1978.1) | -0.66 (-3-1.74) |
| Acute hepatitis B | Republic of Croatia | 29287.2 (23323.5-37394.9) | 594.2 (480.6-747.3) | 17508.8 (13522.4-21841.4) | 345.5 (268.3-428.2) | -0.78 (-3.09-1.6) |
| Acute hepatitis B | Republic of Cuba | 27148.9 (19003.3-37910.5) | 238 (168.6-329.4) | 11775 (7381-17560.5) | 87.7 (58.1-128) | -0.82 (-3.26-1.67) |
| Acute hepatitis B | Republic of Cyprus | 3503.9 (2561.3-4472.3) | 443.1 (326.1-560.9) | 3364.5 (2485.2-4438.4) | 193.6 (145.7-252.5) | -0.74 (-3.01-1.58) |
| Acute hepatitis B | Republic of Djibouti | 5525.5 (4188.5-7206.5) | 1289.6 (964.6-1689.9) | 13149.2 (9104.5-17802.6) | 1015.5 (711.3-1378.6) | -0.35 (-2.55-1.89) |
| Acute hepatitis B | Republic of Ecuador | 30168.9 (26007.3-34941.4) | 327.1 (284.2-375.6) | 44918.2 (38348.9-52367.7) | 249.1 (213.1-290.3) | -0.17 (-2.7-2.42) |
| Acute hepatitis B | Republic of El Salvador | 20120.1 (14542.7-27647.7) | 351.6 (250.9-482.7) | 10357.4 (6753.6-14555) | 160.8 (104.8-225.2) | -0.75 (-3.18-1.75) |
| Acute hepatitis B | Republic of Equatorial Guinea | 4377 (3456.4-5451.9) | 1045.4 (797.8-1321.8) | 11829.1 (9561.5-14791.8) | 807.7 (665.3-989) | -0.63 (-2.56-1.34) |
| Acute hepatitis B | Republic of Estonia | 4937.4 (3727.7-6503.5) | 318.7 (242.2-412.1) | 2415.6 (1641.1-3370.2) | 162.8 (114.1-225.8) | -0.63 (-2.86-1.66) |
| Acute hepatitis B | Republic of Fiji | 6994.5 (5441.5-8721.4) | 928.1 (736.1-1151.9) | 4154.5 (3204-5367.6) | 449.8 (347.6-580.1) | -0.63 (-2.99-1.79) |
| Acute hepatitis B | Republic of Finland | 15085.7 (11060.4-19226.5) | 312.4 (239.8-393.7) | 13398.9 (10169.3-17058.3) | 280.9 (221.4-350.4) | -0.08 (-2.13-2.02) |
| Acute hepatitis B | Republic of Ghana | 435526.7 (346372.1-536830.9) | 2854 (2166.8-3568.5) | 494650.7 (365877.3-669916.8) | 1623.4 (1192.2-2213.3) | -1.01 (-3.4-1.45) |
| Acute hepatitis B | Republic of Guatemala | 55925.9 (43197.8-70144.8) | 635 (468.3-838.5) | 57374.4 (40160.3-81494.5) | 363 (254.9-517.4) | -0.59 (-2.92-1.79) |
| Acute hepatitis B | Republic of Guinea | 153668.9 (118214.8-193223) | 2688.8 (2060.9-3429.3) | 261600.5 (198930-335247) | 2153.6 (1624.7-2808.7) | -0.63 (-3.05-1.86) |
| Acute hepatitis B | Republic of Guinea-Bissau | 30465.1 (23243.2-39029.9) | 3100 (2254.7-3978) | 41378.4 (30819.7-54126.9) | 2219.6 (1659.8-2909.2) | -0.87 (-3.49-1.83) |
| Acute hepatitis B | Republic of Guyana | 4784.9 (3637.7-6189.3) | 590.5 (441.9-766.9) | 2398 (1773-3178.3) | 311.1 (231.1-411.9) | -0.6 (-2.8-1.65) |
| Acute hepatitis B | Republic of Haiti | 36472.8 (28877-46142) | 575.6 (451.8-732.7) | 58278.9 (43230.1-76958.9) | 444.7 (328.5-584.8) | -0.33 (-2.47-1.85) |
| Acute hepatitis B | Republic of Honduras | 22019.8 (16312.1-28967.2) | 452.2 (321.9-605.7) | 20946.8 (14265.3-29444.1) | 221.2 (147.6-315.6) | -0.68 (-2.98-1.67) |
| Acute hepatitis B | Republic of Iceland | 554.3 (414.4-708.7) | 218.1 (165.1-274.7) | 652.5 (499.7-826.5) | 199.4 (156-250.6) | -0.07 (-2.17-2.06) |
| Acute hepatitis B | Republic of India | 7202187.6 (5723515.2-8868735.7) | 833.9 (657.9-1024.2) | 8547673.5 (6630774.3-10743786.1) | 573.7 (450.5-716.1) | -1.13 (-3.66-1.46) |
| Acute hepatitis B | Republic of Indonesia | 2753194.2 (2230161.9-3337757) | 1487.5 (1207.6-1790.7) | 2745944.3 (2203128.4-3354436.4) | 925.6 (750.2-1121.2) | -0.97 (-3.27-1.39) |
| Acute hepatitis B | Republic of Iraq | 66019 (58390.3-75794.9) | 417.9 (371.9-476.7) | 119899.3 (102636.8-138116.3) | 320.1 (276.1-365.9) | -0.4 (-2.74-2) |
| Acute hepatitis B | Republic of Italy | 464662.6 (360476.9-584053.3) | 817 (640.7-1010.9) | 182725.8 (116409.5-264865.2) | 232.2 (158.4-315.9) | -1.34 (-3.48-0.85) |
| Acute hepatitis B | Republic of Kazakhstan | 74977.5 (63057.2-91375.9) | 457.2 (385.8-551.9) | 59355.9 (49476.6-72750.9) | 295.3 (246.5-360.9) | -0.34 (-2.44-1.81) |
| Acute hepatitis B | Republic of Kenya | 414357.7 (334177.4-500950.4) | 1835.6 (1463.7-2218.7) | 416935.5 (309539.4-539276.3) | 943.4 (701.4-1205.8) | -0.62 (-2.84-1.64) |
| Acute hepatitis B | Republic of Kiribati | 1704.1 (1303.3-2086.1) | 2323.9 (1768.8-2887) | 1738.9 (1322.8-2150.8) | 1494.8 (1140.1-1848.7) | -0.75 (-3.32-1.88) |
| Acute hepatitis B | Republic of Korea | 728876.1 (605875-869469.3) | 1547.5 (1307.9-1824.3) | 561855.4 (477564.3-662470.5) | 741.5 (635.1-873) | -0.92 (-3.45-1.68) |
| Acute hepatitis B | Republic of Latvia | 5169.6 (3470.6-7409.7) | 200.2 (137.6-281.7) | 1862.8 (1230.5-2830.7) | 88.9 (59.1-130.7) | -0.58 (-2.95-1.84) |
| Acute hepatitis B | Republic of Liberia | 71321.2 (51075.9-87133.8) | 3089.4 (2219.5-3809.4) | 126044.1 (97558.7-155918.7) | 2535.3 (1981.2-3154.4) | -0.56 (-3.01-1.95) |
| Acute hepatitis B | Republic of Lithuania | 9495.8 (6751.5-12929.2) | 263.2 (190.1-353.8) | 3594 (2326.5-5266.1) | 118.3 (80.7-167.6) | -0.6 (-2.85-1.7) |
| Acute hepatitis B | Republic of Madagascar | 229970.1 (169475.5-290466.5) | 1774.4 (1270.6-2374.7) | 342815.8 (241784-459114.1) | 1252.3 (845.6-1716.1) | -0.48 (-2.83-1.93) |
| Acute hepatitis B | Republic of Malawi | 145192.4 (111911.3-181650.3) | 1464.5 (1131.6-1848.1) | 153875.6 (108998.3-206755.3) | 947.5 (679.8-1246.8) | -0.75 (-2.93-1.48) |
| Acute hepatitis B | Republic of Maldives | 1916.7 (1468.4-2444.5) | 834.8 (623.2-1071.4) | 2586.3 (1740-3599.1) | 393.8 (276.6-532.3) | -1.05 (-3.11-1.06) |
| Acute hepatitis B | Republic of Mali | 149793.5 (117012.5-187253.2) | 1808.8 (1363.8-2342.2) | 271948.2 (205406.7-348573.7) | 1378.7 (1019.3-1801.2) | -0.51 (-2.71-1.75) |
| Acute hepatitis B | Republic of Malta | 777.8 (575.1-1013) | 215.8 (163.1-279) | 514.3 (330.8-720.3) | 103.1 (70.1-139.5) | -0.61 (-3.06-1.9) |
| Acute hepatitis B | Republic of Mauritius | 11207.4 (8694.1-14462.5) | 967.8 (760.4-1212.1) | 7976 (5379.1-10637.4) | 554 (394-718) | -0.8 (-2.88-1.32) |
| Acute hepatitis B | Republic of Moldova | 52193.3 (38000.2-69368.8) | 1165 (850.5-1539.9) | 22998.2 (14928.3-32915.2) | 504.6 (340.5-710.8) | -0.7 (-2.73-1.37) |
| Acute hepatitis B | Republic of Mozambique | 316028.9 (241309.8-393963.4) | 2470.2 (1852.9-3104.8) | 389162.7 (277976.6-508378.9) | 1615.8 (1149.5-2109.4) | -0.55 (-2.94-1.89) |
| Acute hepatitis B | Republic of Namibia | 26475.6 (21386.8-32026.2) | 1850.5 (1496-2290.8) | 28909.5 (21730.6-37068.4) | 1197.2 (902.9-1531.3) | -0.47 (-2.9-2.02) |
| Acute hepatitis B | Republic of Nauru | 171.1 (134-212.3) | 1785.3 (1377.7-2250.3) | 77.3 (57.6-98.9) | 835 (614.7-1092.5) | -0.64 (-3-1.78) |
| Acute hepatitis B | Republic of Nicaragua | 8619 (6666-10990.4) | 216.8 (163.5-277.8) | 7237.6 (5219.3-9955.2) | 108.9 (78.6-148.1) | -0.65 (-3.07-1.82) |
| Acute hepatitis B | Republic of Niue | 30.4 (23.6-39.2) | 1374.9 (1055.8-1780.3) | 9.5 (6.1-13) | 474.7 (320.5-638.1) | -0.83 (-3.2-1.6) |
| Acute hepatitis B | Republic of Palau | 244.2 (194.1-303.7) | 1542.7 (1213.3-1886.2) | 179 (135.1-234.5) | 783 (610.6-1012.6) | -0.63 (-3.04-1.83) |
| Acute hepatitis B | Republic of Panama | 5752.5 (4255.3-7541.4) | 228.5 (170.8-300.8) | 5066.3 (3340-7309.1) | 117.4 (78.1-168.5) | -0.52 (-3.1-2.12) |
| Acute hepatitis B | Republic of Paraguay | 20070.5 (15809.4-25079.9) | 497.5 (385.3-617.5) | 23980.3 (17509.8-31729.6) | 326.7 (243.1-429.2) | -0.59 (-3.01-1.89) |
| Acute hepatitis B | Republic of Peru | 133926.8 (109481.2-167626.8) | 620.8 (495.9-786.6) | 170143.5 (133904.1-216176.3) | 448.8 (355.5-565.9) | -0.42 (-2.96-2.19) |
| Acute hepatitis B | Republic of Poland | 194745.2 (172411.3-220580.2) | 498.5 (442-564.4) | 62724.4 (50686.7-77533.5) | 131.9 (107.4-164.9) | -1.21 (-3.51-1.14) |
| Acute hepatitis B | Republic of Rwanda | 81510.5 (56872.7-106321.8) | 1099.1 (757.5-1458.7) | 83228.4 (52856.2-117024) | 675.9 (435.1-953.6) | -1.15 (-3.3-1.05) |
| Acute hepatitis B | Republic of San Marino | 65.7 (49.7-86.2) | 270.8 (210.3-348.1) | 59.4 (39.7-82.4) | 143.7 (100.1-194.3) | -0.37 (-2.77-2.08) |
| Acute hepatitis B | Republic of Senegal | 184735.6 (147750.6-225969.5) | 2513.2 (1949.9-3101.3) | 236787.7 (168129-313204) | 1724.3 (1222.4-2275) | -0.51 (-3.03-2.07) |
| Acute hepatitis B | Republic of Serbia | 35024 (26507.2-44222.2) | 364.7 (282.4-458.1) | 23775.7 (17588.9-30389) | 240 (185.1-307) | -0.43 (-2.94-2.15) |
| Acute hepatitis B | Republic of Seychelles | 888.7 (701.7-1113.8) | 1204.7 (959.2-1501.6) | 734.6 (559.8-960.8) | 597.1 (457.5-776) | -0.76 (-3-1.53) |
| Acute hepatitis B | Republic of Sierra Leone | 84008.8 (59763.8-110177.4) | 2088.7 (1443.7-2756.6) | 127505.1 (88944.6-173546.9) | 1573.7 (1112.4-2187.7) | -0.71 (-3.04-1.67) |
| Acute hepatitis B | Republic of Singapore | 41625.2 (34487.3-50349) | 1255 (1050.2-1489.8) | 47950.8 (37992.1-58777.4) | 593 (478.6-720.7) | -0.69 (-2.76-1.43) |
| Acute hepatitis B | Republic of Slovenia | 10357.6 (8005.9-12990.9) | 523.6 (412.7-645) | 7091.5 (5469.9-8867.7) | 297.5 (232.9-371.8) | -0.61 (-3-1.84) |
| Acute hepatitis B | Republic of South Africa | 315098.2 (265634-376366.3) | 845.7 (703.8-1006.9) | 210954.9 (169359.9-261212.8) | 353.8 (285.8-432.2) | -0.64 (-2.9-1.66) |
| Acute hepatitis B | Republic of South Sudan | 93414.9 (68643.4-120086.6) | 1608 (1147.3-2097.8) | 130305.3 (94829.8-171285.1) | 1440.7 (1002.5-1912.2) | -0.18 (-2.4-2.08) |
| Acute hepatitis B | Republic of Sudan | 371258.5 (279302.5-469010.4) | 1874.1 (1376.1-2425.8) | 497368.6 (364790-637604.9) | 1234.3 (915.3-1566.3) | -1.25 (-3.87-1.44) |
| Acute hepatitis B | Republic of Suriname | 1498.9 (1138.5-1950.9) | 375.4 (283.4-488.1) | 1386.1 (992.7-1834.3) | 233.1 (167.3-309.7) | -0.59 (-2.86-1.75) |
| Acute hepatitis B | Republic of Tajikistan | 36974.8 (30089.8-45547.2) | 725.6 (584.6-897.7) | 49155.1 (36934.2-63255) | 509.8 (385-651.5) | -0.33 (-2.25-1.62) |
| Acute hepatitis B | Republic of the Congo | 51096.8 (37949.5-66381.1) | 2065.9 (1478.4-2720.3) | 74883.9 (52387.8-100798.3) | 1405.6 (996.1-1910.2) | -0.62 (-2.68-1.48) |
| Acute hepatitis B | Republic of the Gambia | 18670.3 (15173.1-23030.3) | 2161.5 (1721.7-2686.2) | 19189.2 (15767.8-22736.9) | 1119.1 (910.4-1334.5) | -0.72 (-3.04-1.66) |
| Acute hepatitis B | Republic of the Marshall Islands | 838.2 (665.8-1047.4) | 1918.1 (1499.7-2404) | 590.9 (444.8-767.6) | 1066.9 (818.1-1379.4) | -0.53 (-2.98-1.98) |
| Acute hepatitis B | Republic of the Niger | 230793.8 (174802.4-287096.2) | 2960.1 (2139-3828) | 475329.8 (344081.5-606452.1) | 2248.5 (1596.9-2924.6) | -0.6 (-2.99-1.86) |
| Acute hepatitis B | Republic of the Philippines | 1715463.8 (1369866.3-2106012.2) | 2659.6 (2088.1-3275.7) | 1622714.4 (1222705.4-2061041.2) | 1438.5 (1090.1-1822.7) | -0.78 (-3.19-1.7) |
| Acute hepatitis B | Republic of the Union of Myanmar | 265472.3 (219928.8-327267.6) | 647.9 (543.6-791.9) | 279586.7 (228468.2-346412.1) | 488.7 (401-602.6) | -0.6 (-2.65-1.49) |
| Acute hepatitis B | Republic of Trinidad and Tobago | 4136.4 (3165.3-5467.5) | 331.3 (253.4-431.8) | 3060.5 (2104.8-4142.4) | 210.5 (143.9-283.2) | -0.62 (-2.96-1.77) |
| Acute hepatitis B | Republic of Tunisia | 80102.6 (62234.3-97305.1) | 959.8 (749.3-1174.5) | 61761.9 (48259.9-76497.3) | 456.1 (357.1-561.1) | -0.53 (-3-2) |
| Acute hepatitis B | Republic of Turkey | 498900.4 (410804.6-609795.2) | 875.5 (723.3-1059.5) | 467465.1 (384239.1-562624.5) | 508.4 (421.6-610.2) | -0.63 (-3.22-2.04) |
| Acute hepatitis B | Republic of Uganda | 307030 (242638.8-384542.7) | 1740 (1288.5-2241.6) | 413222.5 (305957.2-527415.7) | 1160.9 (833.1-1517.7) | -0.7 (-2.84-1.5) |
| Acute hepatitis B | Republic of Uzbekistan | 345856.6 (279127.6-422726.9) | 1689.8 (1361.4-2070.7) | 387192 (293964.1-496317.8) | 1082 (826.2-1389.4) | -0.47 (-2.53-1.64) |
| Acute hepatitis B | Republic of Vanuatu | 3310.8 (2580.7-4101.2) | 2248.7 (1726.9-2836.5) | 3824.9 (3015.6-4833.8) | 1331.1 (1026.8-1721) | -0.43 (-2.84-2.03) |
| Acute hepatitis B | Republic of Yemen | 258152.4 (196758.9-317292.3) | 1984.2 (1471.5-2465.9) | 394476.1 (298715.2-489364.7) | 1317.4 (1014.9-1640.3) | -1.27 (-4-1.55) |
| Acute hepatitis B | Republic of Zambia | 116911.7 (90159.6-143957.7) | 1529.4 (1174.7-1919.1) | 162488.1 (127428.9-203795.7) | 1008.3 (797.2-1237.8) | -0.73 (-2.92-1.52) |
| Acute hepatitis B | Republic of Zimbabwe | 430090.1 (331380.8-537346.4) | 4183.1 (3099.2-5230) | 368576.5 (275000.1-477668.9) | 2678 (1938.2-3531.4) | -0.29 (-2.95-2.45) |
| Acute hepatitis B | Romania | 261954.4 (205475.7-330265.9) | 1136.8 (901.4-1416.1) | 109040.8 (78779.9-145519.6) | 476.8 (353.6-622.6) | -0.97 (-3.21-1.32) |
| Acute hepatitis B | Russian Federation | 568484.3 (421693.1-714882.6) | 380.1 (285.4-472.4) | 314430.9 (220830.3-426379.4) | 195.2 (139.6-263.6) | -0.38 (-2.78-2.08) |
| Acute hepatitis B | Saint Kitts and Nevis | 176.3 (138.6-229.5) | 420.2 (331.5-540.9) | 153.8 (110-207.6) | 220.3 (159.2-294.5) | -0.84 (-3.12-1.49) |
| Acute hepatitis B | Saint Lucia | 542.8 (423.2-681.7) | 388 (299.5-492.6) | 464.3 (326.8-618.5) | 232.9 (169.1-310.5) | -0.7 (-2.99-1.65) |
| Acute hepatitis B | Saint Vincent and the Grenadines | 416.2 (319.4-566.2) | 365.6 (277.6-487.6) | 259.2 (176-357.1) | 215.2 (145.8-293.6) | -0.65 (-2.98-1.73) |
| Acute hepatitis B | Slovak Republic | 28004 (21798.7-33643.3) | 529.1 (416.1-635.5) | 18436.4 (13750.5-23684.7) | 283.6 (214.5-360.9) | -0.6 (-2.96-1.82) |
| Acute hepatitis B | Socialist Republic of Viet Nam | 1681431.8 (1330902.7-2082975.7) | 2419.5 (1891.4-2999.2) | 1735812.1 (1323229.2-2181702.7) | 1600.3 (1235.8-2003.3) | -0.91 (-3.41-1.65) |
| Acute hepatitis B | Solomon Islands | 8865.6 (6981.1-11052.8) | 2642 (2003.2-3335.5) | 7500.4 (5200.2-10002.4) | 1206.3 (809.6-1637.5) | -0.63 (-3.09-1.89) |
| Acute hepatitis B | State of Eritrea | 60380.2 (45906.3-77978.4) | 1733.2 (1271.9-2268.6) | 66400.6 (42983.4-93231.4) | 1087.9 (722-1506.4) | -0.64 (-2.84-1.61) |
| Acute hepatitis B | State of Israel | 9488.1 (7556-11590.2) | 192.2 (152.5-235.7) | 9151.9 (7159.9-11699.6) | 92.3 (72.2-119.1) | -0.72 (-3.2-1.81) |
| Acute hepatitis B | State of Kuwait | 6762.5 (5532.8-8134.4) | 390.4 (325.1-460.4) | 10971.8 (8820.1-13488.4) | 189.1 (156.5-227.6) | -0.58 (-3.02-1.92) |
| Acute hepatitis B | State of Libya | 32058.7 (26128.8-38756.2) | 814.2 (664.2-985.6) | 39450.4 (31687.1-48371.9) | 512.9 (415.9-617.1) | -0.57 (-3.05-1.98) |
| Acute hepatitis B | State of Qatar | 4776.5 (3997-5759) | 1047.2 (915.5-1222.4) | 20892.1 (16712.1-25927.7) | 610.6 (517.5-715.5) | -0.6 (-2.99-1.84) |
| Acute hepatitis B | Sultanate of Oman | 24279 (18418.8-30638.2) | 1278.4 (972.2-1612.2) | 25872.6 (17942.1-35486.9) | 521.1 (379.1-685.6) | -1.4 (-3.92-1.18) |
| Acute hepatitis B | Swiss Confederation | 18353 (14128.5-23590.7) | 269.2 (209.4-336.6) | 20120 (14636.7-26561.2) | 228 (169.9-299.3) | -0.05 (-2.17-2.13) |
| Acute hepatitis B | Syrian Arab Republic | 153709.5 (131570.8-180808) | 1317.4 (1119.8-1548.3) | 113435.6 (93866.6-133261.9) | 812.4 (676.4-952.4) | -0.58 (-3.02-1.93) |
| Acute hepatitis B | Taiwan (Province of China) | 739932.8 (618744.1-893362.4) | 3347.9 (2806.4-4019.3) | 318677 (234567.3-413298) | 978.3 (762-1235.9) | -1.15 (-3.49-1.25) |
| Acute hepatitis B | Togolese Republic | 89104.1 (66652.1-112677.4) | 2492.7 (1848.6-3259.4) | 142840.6 (105467.2-183385.5) | 1829.5 (1330-2324.4) | -0.46 (-2.82-1.95) |
| Acute hepatitis B | Tokelau | 27.7 (22.5-34.6) | 1819.7 (1466-2268.8) | 12.1 (9.3-15.5) | 894.6 (687.1-1154.3) | -0.7 (-3.13-1.78) |
| Acute hepatitis B | Turkmenistan | 26410 (20342-32583.6) | 739.3 (569.2-898.1) | 27185 (21773.9-34164.1) | 518.5 (419.6-648) | -0.39 (-2.34-1.59) |
| Acute hepatitis B | Tuvalu | 194.6 (155.6-241.9) | 2058.6 (1614-2596.5) | 150 (112.9-193.8) | 1247.4 (940.9-1620) | -0.4 (-2.83-2.08) |
| Acute hepatitis B | Ukraine | 120842.7 (90019.3-155824.4) | 236.8 (180.7-302.9) | 91269.1 (67123.3-123022.3) | 208.8 (158.3-273.2) | 0.21 (-1.85-2.31) |
| Acute hepatitis B | Union of the Comoros | 6392.2 (4559.3-8269.2) | 1335.6 (941.6-1801.7) | 6361.3 (4339.6-8430.9) | 862.9 (597.9-1144) | -0.57 (-2.74-1.66) |
| Acute hepatitis B | United Arab Emirates | 8267.4 (6831.3-10344.3) | 425.3 (360.2-508.7) | 36316.2 (28181.5-46507.5) | 239.9 (198.9-292.9) | -0.52 (-2.92-1.95) |
| Acute hepatitis B | United Kingdom of Great Britain and Northern Ireland | 135755.1 (104905.7-172287.9) | 251.5 (198.5-312.6) | 155333.5 (122654.3-193821.6) | 238.1 (187-295.8) | 0.07 (-2.13-2.32) |
| Acute hepatitis B | United Mexican States | 213890 (178446-259789.4) | 247.7 (205.2-296) | 128477.4 (99591.7-163955.9) | 96.2 (75.7-121.9) | -0.66 (-3.18-1.92) |
| Acute hepatitis B | United Republic of Tanzania | 373872 (283957.9-457589.7) | 1456.3 (1079.7-1834.1) | 456702.8 (351711.7-601019.1) | 928.9 (720.9-1234.7) | -0.55 (-2.62-1.57) |
| Acute hepatitis B | United States of America | 371324.3 (295039.3-456015.3) | 145.1 (117.3-175.3) | 261835.3 (195925.4-337748.2) | 68.1 (52-86.6) | -0.49 (-2.81-1.89) |
| Acute hepatitis B | United States Virgin Islands | 324.7 (241.2-416.4) | 302.1 (224.7-390.7) | 144.7 (101.5-199.9) | 150.6 (113.9-199.1) | -0.71 (-3.07-1.7) |
| Acute hepatitis C | American Samoa | 29.4 (25.2-34.4) | 54.6 (47.3-62) | 27 (23.2-30.9) | 60.8 (53.4-68.7) | -0.45 (-2.97-2.13) |
| Acute hepatitis C | Antigua and Barbuda | 48.2 (42-55.4) | 82.6 (71.6-94.7) | 56.5 (47.7-65.6) | 66.4 (57.3-76.2) | -0.73 (-3.08-1.68) |
| Acute hepatitis C | Arab Republic of Egypt | 252356 (226269.5-282098) | 462.9 (416.3-514) | 331479.3 (285394.4-373733.8) | 346.2 (302.1-386.5) | -1.35 (-3.54-0.88) |
| Acute hepatitis C | Argentine Republic | 13025.1 (11372.7-14944.8) | 40.1 (34.9-46) | 19381.1 (16656.1-22128.9) | 39.9 (34.8-45.2) | -0.42 (-3.45-2.7) |
| Acute hepatitis C | Australia | 15058.5 (13171.4-17460.4) | 93.1 (81.5-106) | 23881.3 (20314.7-27592.8) | 85.6 (74.6-97.9) | -0.51 (-2.65-1.68) |
| Acute hepatitis C | Barbados | 174.2 (150.9-200.9) | 71.5 (62.1-81.9) | 189.9 (162.3-224.2) | 62.9 (54.7-72.7) | -0.57 (-2.97-1.89) |
| Acute hepatitis C | Belize | 194.1 (164.3-225.9) | 96.9 (83.5-111.6) | 329.4 (285.3-376.2) | 83.1 (72.1-94) | -0.55 (-2.82-1.78) |
| Acute hepatitis C | Bermuda | 41 (35.1-47.8) | 72.8 (63-83.7) | 36.8 (30.1-43.1) | 53.5 (45.9-61.5) | -0.63 (-3.06-1.85) |
| Acute hepatitis C | Bolivarian Republic of Venezuela | 15956.4 (13853.1-18344.6) | 81.8 (71.9-92.9) | 18096.8 (15674.6-20752.1) | 69.8 (61.1-79.6) | -0.51 (-3.05-2.1) |
| Acute hepatitis C | Bosnia and Herzegovina | 1303.8 (1128.3-1490.3) | 31.3 (27.4-35.4) | 1242.2 (1044.8-1443.9) | 31.2 (27.2-35.4) | -0.57 (-3.15-2.07) |
| Acute hepatitis C | Brunei Darussalam | 159.6 (138.2-186.3) | 81.4 (70.3-94.1) | 356.9 (296.4-421.8) | 84.8 (72.3-96.4) | -0.81 (-2.87-1.29) |
| Acute hepatitis C | Burkina Faso | 30978.2 (26060.8-36212.4) | 243.2 (212.3-275.5) | 53394.2 (44649.6-62333.9) | 194.4 (170.3-222.2) | -0.66 (-2.95-1.69) |
| Acute hepatitis C | Canada | 11555.9 (9753.1-13425.9) | 41.5 (35.7-47.8) | 17482.3 (14787.6-20248.9) | 39.7 (34.7-45.4) | -0.24 (-2.51-2.09) |
| Acute hepatitis C | Central African Republic | 6240 (5448.9-7152.7) | 224.1 (196.7-253.6) | 10686.3 (9313.8-12283.3) | 204.7 (178.2-233.6) | -0.46 (-2.59-1.73) |
| Acute hepatitis C | Commonwealth of Dominica | 62.3 (54.1-71.8) | 86 (74.7-98.1) | 41.1 (34.2-47.7) | 65.6 (56.8-75.2) | -0.6 (-2.94-1.79) |
| Acute hepatitis C | Commonwealth of the Bahamas | 195.5 (167.9-225.2) | 82 (70.7-94.1) | 235.4 (197.7-274.9) | 66.9 (57.6-76.5) | -0.6 (-2.92-1.79) |
| Acute hepatitis C | Cook Islands | 12.2 (10.5-14.1) | 64.3 (55.5-72.9) | 9.4 (8.1-10.8) | 55.8 (48.6-63.4) | -0.78 (-3.18-1.68) |
| Acute hepatitis C | Czech Republic | 5179.2 (4477.3-5929) | 53.2 (46.5-60) | 5275.9 (4501.8-6060.8) | 47 (41.4-53.1) | -0.45 (-2.68-1.82) |
| Acute hepatitis C | Democratic People's Republic of Korea | 18504.6 (15847.9-21299.3) | 85.1 (72.8-97.2) | 16944 (14830.7-19267.8) | 87.5 (75.7-100.3) | -0.67 (-3.12-1.86) |
| Acute hepatitis C | Democratic Republic of Sao Tome and Principe | 240.3 (203-282.1) | 171.5 (149.2-196.4) | 291.7 (251.2-336.2) | 139.8 (121.7-158.6) | -0.69 (-3.1-1.79) |
| Acute hepatitis C | Democratic Republic of the Congo | 104224.2 (89844.8-120228.2) | 255.7 (223.7-292.1) | 193799.8 (167229.1-221542.3) | 225.8 (194.7-260.6) | -0.35 (-2.52-1.87) |
| Acute hepatitis C | Democratic Republic of Timor-Leste | 1352.3 (1136.1-1581.6) | 127 (111.2-145.9) | 1615.2 (1374.5-1850.9) | 104.6 (90.6-118.4) | -0.54 (-2.76-1.73) |
| Acute hepatitis C | Democratic Socialist Republic of Sri Lanka | 17740.1 (15430.1-20542.1) | 105.1 (91.8-120.3) | 18139.8 (15745.7-20878.1) | 91.8 (80.1-105.1) | -0.61 (-2.68-1.5) |
| Acute hepatitis C | Dominican Republic | 7005.5 (6133-8053.7) | 97.7 (86.2-111.2) | 9214.6 (7960-10586.9) | 86.4 (75.4-99.1) | -0.49 (-2.76-1.84) |
| Acute hepatitis C | Eastern Republic of Uruguay | 1376.6 (1189.2-1585.7) | 41.1 (35.8-47.5) | 1670.6 (1435.1-1929.8) | 40.9 (35.7-46.7) | -0.48 (-3.3-2.42) |
| Acute hepatitis C | Federal Democratic Republic of Ethiopia | 82665.5 (71159.7-96843.7) | 174.6 (150.7-202.5) | 132645 (113564.3-154860.6) | 141.3 (123.4-161.1) | -0.9 (-3.11-1.35) |
| Acute hepatitis C | Federal Democratic Republic of Nepal | 10607.1 (9204-12148) | 56.3 (49.5-64) | 14522.9 (12759-16666.5) | 54 (46.8-61.7) | -1.14 (-3.81-1.6) |
| Acute hepatitis C | Federal Republic of Germany | 41303.1 (34645.2-47468.3) | 46.9 (40.6-53.7) | 46911.6 (39289.4-54362.5) | 41.7 (36.3-47.5) | -0.26 (-2.49-2.02) |
| Acute hepatitis C | Federal Republic of Nigeria | 266522.8 (227100.1-312661) | 237.2 (206.5-270.9) | 440800.4 (373273.8-518774.8) | 171.6 (148.9-196) | -1.48 (-3.87-0.98) |
| Acute hepatitis C | Federal Republic of Somalia | 15088.5 (12738.3-17605.7) | 185.5 (162.7-212.1) | 37115.1 (31856.8-43078.1) | 174.9 (152.4-200.3) | -0.37 (-2.84-2.15) |
| Acute hepatitis C | Federated States of Micronesia | 107.7 (91.1-126.4) | 87.9 (77-100.9) | 89.4 (77.4-102.7) | 93.9 (81.6-106.6) | -0.67 (-2.87-1.57) |
| Acute hepatitis C | Federative Republic of Brazil | 160655.9 (136605.8-189391.3) | 111.5 (95.9-129.5) | 212031.6 (180114-246145.9) | 98.8 (84.7-114) | -1.04 (-3.34-1.31) |
| Acute hepatitis C | French Republic | 27183 (23201.6-31557.3) | 42.8 (37-48.8) | 34571.5 (29050.4-40131.7) | 39.2 (33.6-44.4) | -0.51 (-3.01-2.05) |
| Acute hepatitis C | Gabonese Republic | 2999 (2594.6-3437.8) | 294.4 (255.4-335.5) | 3944.7 (3395-4550) | 231.1 (200.6-265.7) | -0.6 (-2.52-1.36) |
| Acute hepatitis C | Georgia | 9011.2 (7812.2-10229.3) | 179.2 (154.4-203) | 5518 (4800.6-6240.2) | 179.6 (157.5-203.8) | -0.46 (-2.44-1.56) |
| Acute hepatitis C | Grand Duchy of Luxembourg | 210.8 (177.5-243.8) | 51 (43.8-58.1) | 310.6 (252.5-362.1) | 41.6 (35.7-47.6) | -0.68 (-2.91-1.6) |
| Acute hepatitis C | Greenland | 26.4 (22.4-30.8) | 49.2 (42.7-56) | 25.7 (21.7-29.7) | 46.3 (40.1-52.9) | -0.28 (-2.74-2.25) |
| Acute hepatitis C | Grenada | 91.4 (78-105.2) | 99 (86.1-114.7) | 68.6 (57.8-78.4) | 71.4 (61.4-81.6) | -0.81 (-3.1-1.53) |
| Acute hepatitis C | Guam | 79.8 (68.9-91.9) | 59.1 (51.1-66.8) | 93.6 (81.8-106.1) | 60.7 (53.5-69.2) | -0.46 (-2.98-2.12) |
| Acute hepatitis C | Hashemite Kingdom of Jordan | 3568.5 (3034.9-4144.8) | 109.8 (94.7-125.8) | 10633.1 (9063.4-12317.2) | 99 (85.2-112) | -0.82 (-3.26-1.67) |
| Acute hepatitis C | Hellenic Republic | 3771.1 (3253.7-4360.8) | 32.4 (28.7-36.6) | 4565.9 (3751-5335) | 31.9 (27.2-36.5) | -0.45 (-3.13-2.3) |
| Acute hepatitis C | Hungary | 6108.9 (5314.2-6933.7) | 63.2 (56-71.2) | 4425.9 (3758.7-5127.9) | 43.7 (38.1-49.5) | -0.19 (-2.35-2.02) |
| Acute hepatitis C | Independent State of Papua New Guinea | 5452 (4720.9-6344.4) | 103.1 (91-117) | 12786 (10802.1-14879.2) | 101.5 (88.3-115.5) | -0.42 (-2.85-2.06) |
| Acute hepatitis C | Independent State of Samoa | 159.4 (134.6-187.4) | 80.2 (69.5-92.1) | 222.8 (192.5-259) | 91.4 (79.5-104.9) | -0.26 (-2.65-2.2) |
| Acute hepatitis C | Ireland | 1469.8 (1263-1703) | 40.8 (35.2-47.4) | 2262.9 (1917.4-2632.2) | 40 (34.3-45.6) | -0.43 (-2.8-2) |
| Acute hepatitis C | Islamic Republic of Afghanistan | 16412.7 (14234.9-18866.1) | 164.9 (142.3-190.2) | 40097.1 (34656.9-46317.7) | 141.6 (121.5-163) | -1.33 (-4.18-1.6) |
| Acute hepatitis C | Islamic Republic of Iran | 33436.4 (28562.6-39728) | 67.7 (57.7-78.2) | 48857.5 (41107.1-57698.4) | 60.1 (51.9-69.6) | -1.14 (-3.65-1.44) |
| Acute hepatitis C | Islamic Republic of Mauritania | 4041.4 (3440.4-4735) | 169.5 (147.2-193.9) | 5903 (5054.9-6935.7) | 129.3 (112-149) | -1.08 (-3.72-1.64) |
| Acute hepatitis C | Islamic Republic of Pakistan | 194495.8 (168254.8-225458.9) | 156.4 (137.7-177.2) | 335888 (290476.5-387107.5) | 147.5 (128.2-166.8) | -0.99 (-3.17-1.25) |
| Acute hepatitis C | Jamaica | 1753.8 (1522.1-2033.4) | 74.1 (64.5-85.2) | 1624.3 (1361.1-1874.8) | 63.1 (54.6-73) | -0.58 (-2.94-1.84) |
| Acute hepatitis C | Japan | 159522.9 (130120.2-190230.2) | 107.6 (91.6-125.8) | 166199.5 (135842.9-198946.6) | 73.5 (62.9-84.6) | -0.62 (-2.67-1.47) |
| Acute hepatitis C | Kingdom of Bahrain | 478.5 (413.5-565.6) | 111.1 (95.9-126) | 1296.8 (1056-1558.9) | 94.3 (81.4-107.4) | -0.57 (-2.98-1.9) |
| Acute hepatitis C | Kingdom of Belgium | 4608.7 (3986.2-5268.2) | 41.4 (36.5-46.7) | 6431.5 (5436-7432.4) | 44.3 (38.6-50.4) | -0.38 (-2.58-1.87) |
| Acute hepatitis C | Kingdom of Bhutan | 575 (499.8-657.3) | 96.6 (85.1-108.7) | 754.6 (665.1-855.5) | 115.6 (102-131.6) | -0.7 (-3.16-1.82) |
| Acute hepatitis C | Kingdom of Cambodia | 35460.1 (29511.8-40908.2) | 232 (199.2-263.2) | 31705.3 (27459.2-36412.8) | 186.6 (162-213.5) | -1.93 (-4.29-0.49) |
| Acute hepatitis C | Kingdom of Denmark | 2242.5 (1911.8-2601.5) | 40.1 (34.8-45.9) | 2846.1 (2345.3-3284.3) | 39.5 (34.3-44.7) | -0.05 (-2.19-2.13) |
| Acute hepatitis C | Kingdom of Eswatini | 904.4 (769.1-1056.8) | 115.8 (101.6-132.1) | 1206.9 (1050.5-1393.7) | 116.4 (102.1-132.8) | -0.63 (-3.01-1.8) |
| Acute hepatitis C | Kingdom of Lesotho | 1786.1 (1534.4-2093.4) | 116.5 (101.2-133.6) | 2109.3 (1815.7-2438.5) | 124.7 (108.9-142.7) | -0.38 (-2.78-2.08) |
| Acute hepatitis C | Kingdom of Morocco | 27684.9 (23930.8-31981.6) | 118.9 (102.3-136.8) | 38736.6 (33133.2-44987) | 107.8 (93.1-123.6) | -0.53 (-2.93-1.92) |
| Acute hepatitis C | Kingdom of Norway | 1527 (1263.5-1794.4) | 31.6 (26.9-36.7) | 1776.2 (1460.6-2096.4) | 26 (22.3-29.8) | -0.57 (-3.02-1.95) |
| Acute hepatitis C | Kingdom of Saudi Arabia | 27060.6 (23818.7-30922.7) | 195.3 (170.5-222.5) | 55308.1 (45351.2-66716.8) | 159.6 (139.4-181.3) | -1.12 (-3.09-0.89) |
| Acute hepatitis C | Kingdom of Spain | 26094.3 (22877.1-29563.9) | 64.3 (57.6-71.4) | 30554.9 (24894.3-35487.2) | 51 (44.5-57.8) | -0.54 (-2.94-1.93) |
| Acute hepatitis C | Kingdom of Sweden | 2831.9 (2355.3-3357.4) | 27 (23.2-31.2) | 3800.7 (3131.5-4517.7) | 27.5 (23.6-31.8) | -0.08 (-2.33-2.23) |
| Acute hepatitis C | Kingdom of Thailand | 57951.4 (50974.7-65926.1) | 108.7 (96.9-122.6) | 49434.7 (41819.9-56863.7) | 88.7 (77.6-101.4) | -0.43 (-2.66-1.84) |
| Acute hepatitis C | Kingdom of the Netherlands | 4834.8 (4081-5657.7) | 29.8 (25.6-34.4) | 6598.6 (5456.6-7735.2) | 28.7 (24.6-32.8) | -0.23 (-2.69-2.28) |
| Acute hepatitis C | Kingdom of Tonga | 105.5 (89.9-123.6) | 90.9 (80.1-103.4) | 114.5 (99.6-131.3) | 97 (85.2-110) | -1.07 (-3.54-1.46) |
| Acute hepatitis C | Kyrgyz Republic | 12073.1 (10360-13931.7) | 220.1 (190.9-254) | 17073.6 (14725.3-19619.7) | 230.3 (199.8-261.7) | -0.41 (-2.34-1.57) |
| Acute hepatitis C | Lao People's Democratic Republic | 7974.3 (6728.2-9248.3) | 141.1 (122.2-161.5) | 7839.4 (6778.1-8993.3) | 105 (91.8-119.3) | -1.02 (-3.31-1.33) |
| Acute hepatitis C | Lebanese Republic | 2346.1 (2045.1-2677.3) | 82.9 (71.8-94.3) | 3778.1 (3147.7-4384.3) | 66.4 (56.5-75.8) | -0.49 (-3.12-2.22) |
| Acute hepatitis C | Malaysia | 27048 (22976.7-31254.7) | 132.5 (115.5-149.9) | 31797.7 (27580.1-36581.5) | 115.6 (100.2-131.8) | -0.71 (-2.81-1.44) |
| Acute hepatitis C | Mongolia | 12004.3 (10252.9-13919.3) | 406.8 (355.9-463.5) | 14946.4 (12769.1-17286.3) | 410.1 (360.2-468.8) | -0.49 (-2.47-1.54) |
| Acute hepatitis C | Montenegro | 262.4 (225.4-298.7) | 44.4 (38.3-50.7) | 269.6 (228-314.1) | 43.4 (37.7-49.4) | -0.41 (-2.84-2.09) |
| Acute hepatitis C | New Zealand | 1803.8 (1549.8-2082.1) | 52.5 (45.1-60.4) | 3364.3 (2828-3917.7) | 58.3 (50.4-67) | -0.45 (-2.63-1.77) |
| Acute hepatitis C | North Macedonia | 912.8 (779.6-1034.9) | 48.8 (42.3-55.2) | 1011 (855-1187.1) | 47.2 (41.3-53.6) | -0.49 (-2.9-1.97) |
| Acute hepatitis C | Northern Mariana Islands | 28.8 (24.5-33.6) | 69.7 (60.5-79.5) | 30.3 (25.7-34.8) | 72.4 (63.1-82.1) | -0.4 (-2.94-2.21) |
| Acute hepatitis C | Palestine | 2327.9 (1991.2-2680.8) | 125.5 (109.4-143.6) | 4628.3 (3957.5-5343.2) | 107 (92-122.6) | -0.54 (-2.92-1.89) |
| Acute hepatitis C | People's Democratic Republic of Algeria | 24549.4 (21164.6-28295) | 110.7 (96.1-126.9) | 40951.2 (35060.2-47456.5) | 96.5 (82.6-109.9) | -0.39 (-2.8-2.09) |
| Acute hepatitis C | People's Republic of Bangladesh | 70516.8 (61557.6-79626.6) | 68.7 (60.4-77.3) | 77210.2 (66808.7-87963) | 53 (46-60.2) | -1.28 (-3.86-1.38) |
| Acute hepatitis C | People's Republic of China | 976188.4 (847445.1-1122754.1) | 89.1 (77.6-102.1) | 666818.3 (558915.2-771329.4) | 55.2 (48.2-63.3) | -1.62 (-4.05-0.88) |
| Acute hepatitis C | Plurinational State of Bolivia | 4268.2 (3674.8-4986.3) | 65.4 (57-75.3) | 6483.7 (5648.4-7403.6) | 58.1 (50.6-66.1) | -0.28 (-2.74-2.24) |
| Acute hepatitis C | Portuguese Republic | 6059.1 (5111.3-7076.2) | 58.3 (50.4-66.6) | 8117.1 (6755.2-9524.2) | 63.9 (55.3-72.7) | -0.63 (-2.98-1.78) |
| Acute hepatitis C | Principality of Andorra | 27.3 (23.2-31.8) | 51.7 (44.9-59.2) | 54.7 (45.2-64.4) | 51.6 (44.8-58.8) | -0.55 (-2.75-1.7) |
| Acute hepatitis C | Principality of Monaco | 15.5 (12.9-18.5) | 37.5 (32.3-42.9) | 24.7 (20.3-29.7) | 45 (39-51.7) | -0.53 (-2.8-1.79) |
| Acute hepatitis C | Puerto Rico | 3071 (2678.9-3501.7) | 87.6 (76.6-99.7) | 2251.1 (1886-2644.5) | 64 (55.6-73.3) | -0.81 (-3.15-1.59) |
| Acute hepatitis C | Republic of Albania | 1775.3 (1545.7-2038.4) | 57.5 (50.5-65.7) | 1292.9 (1096.4-1505.8) | 46.6 (40.9-53.2) | -0.69 (-3.21-1.89) |
| Acute hepatitis C | Republic of Angola | 31258 (26869.1-35702.8) | 283.3 (245.2-321.8) | 70771.5 (60430.3-81737.4) | 222.8 (191.9-257.3) | -0.92 (-3.1-1.3) |
| Acute hepatitis C | Republic of Armenia | 6010.5 (5175.3-6870.5) | 169.2 (145.8-192.7) | 4520.6 (3934.6-5142.5) | 178 (154.8-202.2) | -0.36 (-2.33-1.64) |
| Acute hepatitis C | Republic of Austria | 4067.5 (3428.5-4730.3) | 48.3 (41.6-55.7) | 4649.7 (3943.2-5394.3) | 40.9 (35.8-46.2) | -0.6 (-2.91-1.77) |
| Acute hepatitis C | Republic of Azerbaijan | 19190.3 (16443.2-22236) | 236.1 (204.9-269.4) | 19210.1 (16762.2-22075.3) | 223 (193.6-254.7) | -0.35 (-2.3-1.64) |
| Acute hepatitis C | Republic of Belarus | 7934.5 (6907-9063.2) | 82.5 (72.2-94.1) | 7481.3 (6268.7-8595.4) | 91.6 (79.9-103.1) | -0.72 (-3.05-1.67) |
| Acute hepatitis C | Republic of Benin | 11572.8 (9728.6-13729.8) | 187.7 (162.9-213.9) | 22973.2 (19406.7-26664.2) | 152.2 (131.9-172.9) | -0.63 (-2.94-1.73) |
| Acute hepatitis C | Republic of Botswana | 1484.9 (1277.1-1723.6) | 117.4 (102.8-133.5) | 2370 (2035.9-2756.7) | 108.5 (94.1-124.5) | -0.77 (-3.1-1.61) |
| Acute hepatitis C | Republic of Bulgaria | 4202.3 (3562.7-4832.8) | 50.4 (43.9-56.8) | 3401.2 (2890.5-4014.2) | 47.3 (41.8-53.7) | -0.72 (-3.28-1.9) |
| Acute hepatitis C | Republic of Burundi | 14548.4 (12461.7-16876.8) | 236.2 (208.5-267.5) | 26387.2 (22630.9-30418.6) | 202 (176-230.1) | -0.45 (-2.42-1.55) |
| Acute hepatitis C | Republic of Cabo Verde | 667 (567.4-775.5) | 162.3 (139.9-184.5) | 645.7 (552.2-756.1) | 126.6 (110.5-145.9) | -0.73 (-3.06-1.65) |
| Acute hepatitis C | Republic of Cameroon | 29342.4 (25288.6-34279.9) | 220 (194.1-250.2) | 55981.8 (47289.4-65087) | 162.6 (141.1-186.6) | -0.55 (-2.75-1.7) |
| Acute hepatitis C | Republic of Chad | 18042.2 (15358.2-21134.1) | 221.5 (194.4-251) | 44003.6 (37187.6-51096.9) | 191.5 (166.5-217.6) | -0.45 (-2.87-2.02) |
| Acute hepatitis C | Republic of Chile | 8122.6 (7145-9198.2) | 65.8 (57.8-75.2) | 12044.5 (10214-13901.3) | 58.4 (51.1-66.3) | -0.38 (-3.01-2.32) |
| Acute hepatitis C | Republic of Colombia | 28649.1 (24589.6-33163.5) | 84.5 (74.2-96.2) | 30843.2 (25912.6-35642) | 67.7 (57.6-78) | -0.92 (-3.39-1.62) |
| Acute hepatitis C | Republic of Costa Rica | 2717.6 (2358.6-3141.9) | 85.8 (75.3-97.2) | 3334.9 (2860.5-3829.1) | 75.1 (66.1-85.6) | -0.59 (-3.06-1.95) |
| Acute hepatitis C | Republic of C么te d'Ivoire | 24868.1 (21212.4-29133.8) | 170.9 (148.4-195.3) | 41730.5 (35496.7-48264.6) | 139.6 (120.8-159.2) | -0.66 (-3-1.74) |
| Acute hepatitis C | Republic of Croatia | 2331.3 (1991-2665.4) | 51.9 (45-59) | 1965.9 (1622.9-2284.8) | 42.4 (36.6-47.9) | -0.78 (-3.09-1.6) |
| Acute hepatitis C | Republic of Cuba | 7874.8 (6784.6-9044.5) | 77.9 (67.7-89) | 7398.7 (6207.2-8587.1) | 65.9 (58-75) | -0.82 (-3.26-1.67) |
| Acute hepatitis C | Republic of Cyprus | 329.5 (283.6-383.2) | 42.4 (36.7-48.6) | 592.4 (502.7-692.8) | 37.6 (32.4-43.3) | -0.74 (-3.01-1.58) |
| Acute hepatitis C | Republic of Djibouti | 524.7 (451.1-608) | 141.4 (123.7-160.7) | 1409.3 (1221.9-1629.5) | 125.6 (110.4-142.4) | -0.35 (-2.55-1.89) |
| Acute hepatitis C | Republic of Ecuador | 5163.6 (4490.1-5907.2) | 52.8 (46.1-59.7) | 8755.3 (7571.7-9976.9) | 50.7 (43.9-57.5) | -0.17 (-2.7-2.42) |
| Acute hepatitis C | Republic of El Salvador | 6189.4 (5375.8-7118.3) | 104.1 (91.3-118.8) | 5462.1 (4732.8-6242.3) | 86.9 (75.8-99.3) | -0.75 (-3.18-1.75) |
| Acute hepatitis C | Republic of Equatorial Guinea | 1204.3 (1034.5-1375.3) | 259.9 (227.2-296.2) | 2180.7 (1871.7-2550.3) | 169.8 (145.8-194.8) | -0.63 (-2.56-1.34) |
| Acute hepatitis C | Republic of Estonia | 1186 (1019.4-1351.4) | 80.9 (69.9-92.2) | 1086.3 (919.7-1248.4) | 86.9 (75-98.7) | -0.63 (-2.86-1.66) |
| Acute hepatitis C | Republic of Fiji | 501.9 (424.5-585.2) | 64.9 (56-74.4) | 559.1 (490-638.4) | 63.4 (55.9-71.8) | -0.63 (-2.99-1.79) |
| Acute hepatitis C | Republic of Finland | 2248.4 (1894.9-2643.1) | 41.4 (35.8-47.4) | 3072.3 (2522.8-3610.6) | 42.6 (36.3-48.7) | -0.08 (-2.13-2.02) |
| Acute hepatitis C | Republic of Ghana | 41616.7 (36538.3-47308.7) | 225.3 (200.8-254.8) | 59733.6 (51456.2-68758) | 168.3 (146.9-190.8) | -1.01 (-3.4-1.45) |
| Acute hepatitis C | Republic of Guatemala | 15528.2 (13385-17969.4) | 145.6 (127.5-164.9) | 17503.2 (15213.3-19904.8) | 113.9 (99.5-128.2) | -0.59 (-2.92-1.79) |
| Acute hepatitis C | Republic of Guinea | 14921 (12786.7-17404.2) | 196.4 (171.8-223.4) | 24563.6 (20976.8-28248.8) | 163.7 (141.4-186.9) | -0.63 (-3.05-1.86) |
| Acute hepatitis C | Republic of Guinea-Bissau | 1852.3 (1574.6-2168.7) | 159.1 (138.9-181.8) | 2681.4 (2299-3130) | 126.3 (109-143.9) | -0.87 (-3.49-1.83) |
| Acute hepatitis C | Republic of Guyana | 922.6 (788.6-1072.2) | 113.6 (98.9-129) | 682.8 (594.6-777.3) | 92.3 (81-104.8) | -0.6 (-2.8-1.65) |
| Acute hepatitis C | Republic of Haiti | 9986.6 (8468-11655.4) | 134.3 (117-154.2) | 14491.1 (12417-16690.1) | 111.6 (97.1-127.4) | -0.33 (-2.47-1.85) |
| Acute hepatitis C | Republic of Honduras | 7147.3 (6059.9-8358.2) | 123.8 (107.7-141.7) | 10561.8 (9168.7-12161.1) | 107.2 (92.9-121.6) | -0.68 (-2.98-1.67) |
| Acute hepatitis C | Republic of Iceland | 89.5 (77-103.9) | 34.8 (30.1-40.2) | 130.5 (110.8-151.7) | 32.1 (27.9-36.8) | -0.07 (-2.17-2.06) |
| Acute hepatitis C | Republic of India | 694623.5 (601542.2-803828.9) | 85.4 (74.4-97.6) | 851998.2 (741652.8-972121.1) | 69.1 (60.4-78.4) | -1.13 (-3.66-1.46) |
| Acute hepatitis C | Republic of Indonesia | 265928.3 (227961.5-310141.5) | 135.3 (118.4-155.4) | 296141.1 (258158.7-340958.9) | 119.4 (104.9-135.7) | -0.97 (-3.27-1.39) |
| Acute hepatitis C | Republic of Iraq | 24124.1 (20844.3-27939.8) | 136.2 (116.4-155.7) | 46776.1 (40183.3-54288.8) | 126.1 (108.5-144.8) | -0.4 (-2.74-2) |
| Acute hepatitis C | Republic of Italy | 66102.9 (55284.6-77190.1) | 108.1 (93.7-124.6) | 58335.8 (47910.7-69566.9) | 78.3 (67.8-90.4) | -1.34 (-3.48-0.85) |
| Acute hepatitis C | Republic of Kazakhstan | 19341 (16708.1-21853.3) | 114.1 (99.8-128.1) | 24620.5 (21495.4-27772.7) | 129.9 (113.8-148) | -0.34 (-2.44-1.81) |
| Acute hepatitis C | Republic of Kenya | 30555.6 (25957.4-36058.9) | 138.8 (120.6-160.3) | 57593.2 (49704.1-67152.9) | 132.7 (115.7-151.9) | -0.62 (-2.84-1.64) |
| Acute hepatitis C | Republic of Kiribati | 89 (76.1-103.6) | 98 (86-112.2) | 133.3 (114.3-154.3) | 105.3 (91.4-120.2) | -0.75 (-3.32-1.88) |
| Acute hepatitis C | Republic of Korea | 27538.3 (24123.4-31335.6) | 73.6 (64.5-83.2) | 44697.1 (37130.1-52548) | 60.6 (51.9-69.2) | -0.92 (-3.45-1.68) |
| Acute hepatitis C | Republic of Latvia | 1869.3 (1594.3-2123.3) | 75.2 (64.8-85.6) | 1383.6 (1173.3-1587.4) | 78.1 (67.9-88.9) | -0.58 (-2.95-1.84) |
| Acute hepatitis C | Republic of Liberia | 5740.9 (4871.5-6685) | 191.9 (168-219.5) | 8961.1 (7668.4-10466.7) | 160.7 (140.1-183) | -0.56 (-3.01-1.95) |
| Acute hepatitis C | Republic of Lithuania | 3113 (2688.4-3566.4) | 91.7 (79.3-105.5) | 2337.3 (1994.5-2679) | 95.6 (83.1-108.7) | -0.6 (-2.85-1.7) |
| Acute hepatitis C | Republic of Madagascar | 13124 (11460.3-15113.1) | 119.1 (103.5-136.3) | 25677 (22119.5-29713.8) | 105.8 (91.6-122.5) | -0.48 (-2.83-1.93) |
| Acute hepatitis C | Republic of Malawi | 17513.8 (15028.3-20198.5) | 177.1 (154.6-201.9) | 24894.2 (21380.7-28731.8) | 148.2 (128.3-169.3) | -0.75 (-2.93-1.48) |
| Acute hepatitis C | Republic of Maldives | 546.8 (458.2-648.6) | 167 (144-192.1) | 444.7 (377.9-518.9) | 113 (98.7-128.6) | -1.05 (-3.11-1.06) |
| Acute hepatitis C | Republic of Mali | 23180.9 (19681.6-27137.6) | 204 (178.5-234.1) | 44678.7 (37590.9-52327.1) | 155.2 (134.5-177) | -0.51 (-2.71-1.75) |
| Acute hepatitis C | Republic of Malta | 155.8 (131.4-182.2) | 41.4 (35.6-48) | 213 (175-250.5) | 37.6 (32.2-43) | -0.61 (-3.06-1.9) |
| Acute hepatitis C | Republic of Mauritius | 1533.2 (1329.9-1779.5) | 144.3 (126.3-165.5) | 1179.3 (1012.6-1356) | 122.7 (107.2-140.9) | -0.8 (-2.88-1.32) |
| Acute hepatitis C | Republic of Moldova | 5608.8 (4853.8-6442.8) | 129.1 (112.1-148.3) | 3715.4 (3122.5-4295.8) | 122.4 (106.8-139.5) | -0.7 (-2.73-1.37) |
| Acute hepatitis C | Republic of Mozambique | 17640.5 (15312.6-20190) | 135.9 (118.2-154.3) | 33516.7 (29041.8-38813.8) | 124.4 (108.5-141.5) | -0.55 (-2.94-1.89) |
| Acute hepatitis C | Republic of Namibia | 1467.4 (1261.5-1706.5) | 107.9 (95.1-123.6) | 2299.6 (1982.7-2664) | 103.8 (88.8-118.7) | -0.47 (-2.9-2.02) |
| Acute hepatitis C | Republic of Nauru | 9.6 (8.1-11.2) | 80 (69.6-91) | 10.3 (8.9-11.8) | 88.3 (77-100.6) | -0.64 (-3-1.78) |
| Acute hepatitis C | Republic of Nicaragua | 6750.2 (5804.8-7807.6) | 140.1 (123.5-157.5) | 7331.3 (6374.8-8479.4) | 114.8 (100.6-131.3) | -0.65 (-3.07-1.82) |
| Acute hepatitis C | Republic of Niue | 1.7 (1.4-1.9) | 68.4 (59.5-77.9) | 1 (0.9-1.2) | 66.1 (57.8-75.6) | -0.83 (-3.2-1.6) |
| Acute hepatitis C | Republic of Palau | 9.8 (8.4-11.5) | 69.1 (59.9-79.5) | 11.1 (9.4-13) | 75.2 (65.7-85.3) | -0.63 (-3.04-1.83) |
| Acute hepatitis C | Republic of Panama | 1799.6 (1553.3-2085) | 75 (64.9-86) | 2686.4 (2319.9-3051) | 64.6 (56-73.2) | -0.52 (-3.1-2.12) |
| Acute hepatitis C | Republic of Paraguay | 4077.5 (3529.5-4724.9) | 96.6 (83.7-111) | 6044.5 (5234.9-6921.3) | 89.2 (78.1-102) | -0.59 (-3.01-1.89) |
| Acute hepatitis C | Republic of Peru | 12162.5 (10678-13872) | 57.2 (50.4-65.3) | 16872.5 (14619.9-19338.5) | 48.2 (42.2-55) | -0.42 (-2.96-2.19) |
| Acute hepatitis C | Republic of Poland | 20442.3 (17681.8-23654.1) | 55.1 (48-63) | 17205 (14228.3-19940.6) | 44.1 (38.4-50.6) | -1.21 (-3.51-1.14) |
| Acute hepatitis C | Republic of Rwanda | 13452.6 (11643.9-15479) | 193.5 (168.7-220.6) | 16784.1 (14599.3-19086.9) | 143.3 (125-163.1) | -1.15 (-3.3-1.05) |
| Acute hepatitis C | Republic of San Marino | 10.7 (9-12.4) | 40.4 (34.6-46) | 18.2 (14.9-21.4) | 40.8 (35.1-47.1) | -0.37 (-2.77-2.08) |
| Acute hepatitis C | Republic of Senegal | 9909.6 (8529.9-11450) | 118.1 (104.1-133.9) | 17861 (15376.1-20735.3) | 113 (97.9-128.8) | -0.51 (-3.03-2.07) |
| Acute hepatitis C | Republic of Serbia | 4469.3 (3792.6-5117.6) | 48.6 (42.2-55.2) | 4401.5 (3752.2-5157.7) | 46.3 (40.6-52.2) | -0.43 (-2.94-2.15) |
| Acute hepatitis C | Republic of Seychelles | 79.3 (68.7-91.1) | 105.4 (92.1-120.4) | 90.2 (78.3-102.7) | 95.9 (84.3-108.3) | -0.76 (-3-1.53) |
| Acute hepatitis C | Republic of Sierra Leone | 9389.2 (7978.4-10907.3) | 183.6 (162.3-209.1) | 14091.6 (12067.4-16376.2) | 148.5 (128.1-169.5) | -0.71 (-3.04-1.67) |
| Acute hepatitis C | Republic of Singapore | 2024.5 (1699-2408.3) | 76.9 (65.4-88.8) | 5798.3 (4755.7-6893.9) | 81.3 (69.7-94.3) | -0.69 (-2.76-1.43) |
| Acute hepatitis C | Republic of Slovenia | 946.2 (809.8-1074.3) | 51.2 (44.4-58.1) | 1006.6 (848.9-1187.6) | 42.1 (37-47.5) | -0.61 (-3-1.84) |
| Acute hepatitis C | Republic of South Africa | 31390.6 (27299.4-36279.8) | 90.8 (78.3-104.8) | 47609.1 (40823.1-55346.7) | 89.6 (77.2-102.5) | -0.64 (-2.9-1.66) |
| Acute hepatitis C | Republic of South Sudan | 9012.7 (7751.8-10415.6) | 159.7 (139.3-182) | 13096.7 (11203.8-15134.6) | 145.3 (126-166.3) | -0.18 (-2.4-2.08) |
| Acute hepatitis C | Republic of Sudan | 26601.4 (22877.3-30642.4) | 138.9 (119.7-159.2) | 42884.1 (37109.5-49496.3) | 114.3 (99-131.6) | -1.25 (-3.87-1.44) |
| Acute hepatitis C | Republic of Suriname | 357 (306.3-413.1) | 94.4 (81.7-108.8) | 444.3 (378.9-512.8) | 79.7 (69-91.6) | -0.59 (-2.86-1.75) |
| Acute hepatitis C | Republic of Tajikistan | 18900.4 (16092.9-21757.4) | 240.5 (207.5-275.4) | 28017.9 (24059.8-32470.3) | 235.3 (204.7-269.2) | -0.33 (-2.25-1.62) |
| Acute hepatitis C | Republic of the Congo | 5679.2 (4898.1-6567.1) | 242.4 (209-280.8) | 10129.2 (8696.7-11946) | 202.4 (174.9-237.9) | -0.62 (-2.68-1.48) |
| Acute hepatitis C | Republic of the Gambia | 1507.6 (1289.6-1752.1) | 140.8 (124-160.6) | 2788.4 (2401.5-3232.9) | 120 (104.3-137.3) | -0.72 (-3.04-1.66) |
| Acute hepatitis C | Republic of the Marshall Islands | 52.8 (43.9-62.3) | 93.2 (81.6-105.9) | 52.2 (45.1-59.7) | 97.1 (84.9-110.1) | -0.53 (-2.98-1.98) |
| Acute hepatitis C | Republic of the Niger | 22440.4 (18892.7-26307.1) | 208.9 (181.3-237.2) | 54745.2 (46152-64289.9) | 175.4 (152.6-201) | -0.6 (-2.99-1.86) |
| Acute hepatitis C | Republic of the Philippines | 75516.6 (63703.9-89086.9) | 104 (89.9-120.5) | 97293.6 (82924.2-113087.4) | 89.1 (77-102.3) | -0.78 (-3.19-1.7) |
| Acute hepatitis C | Republic of the Union of Myanmar | 67666.8 (58132.5-78140.7) | 149.4 (130.5-169.7) | 70142.6 (60896.9-80231.9) | 131.2 (114-149.2) | -0.6 (-2.65-1.49) |
| Acute hepatitis C | Republic of Trinidad and Tobago | 960.5 (826-1117) | 82.2 (71.6-94.5) | 864.6 (731-1009.1) | 64.2 (55.4-73.7) | -0.62 (-2.96-1.77) |
| Acute hepatitis C | Republic of Tunisia | 7380 (6418.6-8459.3) | 100.6 (87.7-114.6) | 11949 (10103.8-13841.4) | 99.1 (85.5-111.8) | -0.53 (-3-2) |
| Acute hepatitis C | Republic of Turkey | 33902.9 (29451.5-38298.5) | 68 (59.2-76.9) | 50076.9 (42229.7-57820.2) | 59.2 (50.6-67.7) | -0.63 (-3.22-2.04) |
| Acute hepatitis C | Republic of Uganda | 36303.9 (30933.7-42451.3) | 195.3 (171.4-222.4) | 62913.6 (54282.4-73006.2) | 157.3 (137.3-180.1) | -0.7 (-2.84-1.5) |
| Acute hepatitis C | Republic of Uzbekistan | 59363.1 (51644.4-67646.2) | 215.1 (188.7-242.1) | 84110.7 (73485.7-94876.2) | 236.6 (207.2-267) | -0.47 (-2.53-1.64) |
| Acute hepatitis C | Republic of Vanuatu | 189.5 (158.6-221.9) | 95.5 (83.5-109.1) | 362.6 (310.7-418) | 103.5 (90.9-117.9) | -0.43 (-2.84-2.03) |
| Acute hepatitis C | Republic of Yemen | 23993.5 (20808.6-27515.3) | 178.5 (156.1-203.4) | 41811.6 (36252.8-48168.5) | 140.1 (120.7-159.4) | -1.27 (-4-1.55) |
| Acute hepatitis C | Republic of Zambia | 13345.7 (11503.8-15383.2) | 174.6 (152.4-197.3) | 24463.5 (21130.7-28133) | 146.3 (127.6-166.1) | -0.73 (-2.92-1.52) |
| Acute hepatitis C | Republic of Zimbabwe | 11779.9 (10184.3-13561.7) | 122.4 (106.8-138.5) | 19081.1 (16530.8-22016.4) | 134.7 (116.7-153.4) | -0.29 (-2.95-2.45) |
| Acute hepatitis C | Romania | 14925.5 (12802.3-17042.3) | 69.3 (60.7-78.2) | 11931 (10038.1-13836.3) | 62.6 (54.5-70.8) | -0.97 (-3.21-1.32) |
| Acute hepatitis C | Russian Federation | 111004.3 (96417.4-128390.7) | 79.2 (68.7-91.1) | 123521.2 (105136-141709.2) | 95.8 (82.7-110.2) | -0.38 (-2.78-2.08) |
| Acute hepatitis C | Saint Kitts and Nevis | 40.8 (35.5-46.3) | 99.2 (86.3-113) | 38.2 (32-45.2) | 69.1 (59.8-78.7) | -0.84 (-3.12-1.49) |
| Acute hepatitis C | Saint Lucia | 131.3 (113.4-151.9) | 95.9 (83.4-109.1) | 119.9 (99.6-140.4) | 71.6 (61.9-82.4) | -0.7 (-2.99-1.65) |
| Acute hepatitis C | Saint Vincent and the Grenadines | 95.3 (82.3-110.9) | 88.9 (77.6-101.7) | 76.2 (64.4-87.6) | 70.5 (61.4-80.4) | -0.65 (-2.98-1.73) |
| Acute hepatitis C | Slovak Republic | 2687.4 (2328.7-3042.5) | 53.4 (46.4-60.5) | 2550.7 (2164.2-2960.1) | 46.7 (40.9-53.1) | -0.6 (-2.96-1.82) |
| Acute hepatitis C | Socialist Republic of Viet Nam | 101402.8 (87391.6-117415.1) | 129 (112.9-147) | 88978.6 (78219.7-102314.5) | 97.5 (86.5-111.1) | -0.91 (-3.41-1.65) |
| Acute hepatitis C | Solomon Islands | 371.3 (315.4-433.9) | 85.4 (74.6-96.8) | 797.4 (685.8-921.9) | 102.1 (88.8-114.9) | -0.63 (-3.09-1.89) |
| Acute hepatitis C | State of Eritrea | 5591.9 (4793.7-6476.3) | 170.4 (149.1-195.1) | 8851.6 (7687.8-10146.5) | 150.7 (131.9-172.7) | -0.64 (-2.84-1.61) |
| Acute hepatitis C | State of Israel | 1866.7 (1621.6-2165.8) | 38.2 (33-44.2) | 3912.7 (3405.3-4474.8) | 37.3 (32.4-42.5) | -0.72 (-3.2-1.81) |
| Acute hepatitis C | State of Kuwait | 1212.3 (1032.6-1443.9) | 81.1 (69.2-93.5) | 3283.9 (2670.4-3937.9) | 75.6 (65.7-85.3) | -0.58 (-3.02-1.92) |
| Acute hepatitis C | State of Libya | 3787.9 (3271-4352.5) | 104.7 (91.1-119.8) | 5724.1 (4710.2-6807.1) | 88.7 (75.9-101.9) | -0.57 (-3.05-1.98) |
| Acute hepatitis C | State of Qatar | 422 (355.1-505.8) | 110.8 (95.9-125.8) | 2379.2 (1923.5-2876.1) | 95.4 (83.1-108) | -0.6 (-2.99-1.84) |
| Acute hepatitis C | Sultanate of Oman | 2087.5 (1783.8-2423) | 115.4 (99.7-130.9) | 3798 (3186.2-4502.6) | 92.1 (79-104) | -1.4 (-3.92-1.18) |
| Acute hepatitis C | Swiss Confederation | 3263.2 (2723.5-3772.8) | 43.4 (37.2-49.4) | 5024.6 (4185.1-5857.9) | 45.3 (39-51.8) | -0.05 (-2.17-2.13) |
| Acute hepatitis C | Syrian Arab Republic | 13808.5 (11880.2-15864.3) | 122.3 (105.1-140.1) | 14100.2 (11992.5-16313.8) | 108.1 (94-123.6) | -0.58 (-3.02-1.93) |
| Acute hepatitis C | Taiwan (Province of China) | 19412.7 (17833.8-21393.4) | 112.7 (103.7-124.2) | 16591.3 (14148.6-18888.8) | 104.4 (91.7-118) | -1.15 (-3.49-1.25) |
| Acute hepatitis C | Togolese Republic | 7599.9 (6396.6-9007.6) | 175.1 (152.7-199.3) | 12491.2 (10711.9-14334.8) | 144.2 (124.6-164.2) | -0.46 (-2.82-1.95) |
| Acute hepatitis C | Tokelau | 1.3 (1.1-1.5) | 76.3 (66.7-87.9) | 0.9 (0.8-1) | 72.3 (63-82.1) | -0.7 (-3.13-1.78) |
| Acute hepatitis C | Turkmenistan | 11102.5 (9594-12788.3) | 226.3 (197.7-257.2) | 12660.7 (10934.7-14424.8) | 241.8 (210.1-274.4) | -0.39 (-2.34-1.59) |
| Acute hepatitis C | Tuvalu | 9.6 (8.2-11.1) | 83.4 (72.9-95.3) | 11.1 (9.7-12.7) | 90.8 (79.3-102.9) | -0.4 (-2.83-2.08) |
| Acute hepatitis C | Ukraine | 48885.1 (42208-56649.7) | 102.6 (88.5-119) | 44356.2 (37783-51301.8) | 131.5 (113.5-153.5) | 0.21 (-1.85-2.31) |
| Acute hepatitis C | Union of the Comoros | 676.1 (581.5-782.2) | 151.7 (132.8-174.6) | 853.3 (742.9-970) | 126 (109.1-143.7) | -0.57 (-2.74-1.66) |
| Acute hepatitis C | United Arab Emirates | 1482.5 (1248.7-1772.1) | 91.6 (79.6-104) | 7645.8 (6006.5-9460.8) | 80.8 (70.3-92.2) | -0.52 (-2.92-1.95) |
| Acute hepatitis C | United Kingdom of Great Britain and Northern Ireland | 30640.1 (25864.9-35379.8) | 49.8 (43.2-57.2) | 39184.6 (32772.1-45915.1) | 48.3 (41.7-55.2) | 0.07 (-2.13-2.32) |
| Acute hepatitis C | United Mexican States | 76608.8 (63512.2-91286.8) | 87.6 (75.1-101.8) | 90444.6 (77381.6-105604.1) | 72.7 (62.8-83.7) | -0.66 (-3.18-1.92) |
| Acute hepatitis C | United Republic of Tanzania | 51778.9 (43764.2-59959.1) | 192 (166.4-218.1) | 102889 (89470.3-118171.7) | 183.3 (159.7-208.7) | -0.55 (-2.62-1.57) |
| Acute hepatitis C | United States of America | 128775.1 (110634.8-148622.6) | 49.8 (43.2-57.4) | 179843.2 (151918.8-206954.1) | 49.6 (43.3-57.1) | -0.49 (-2.81-1.89) |
| Acute hepatitis C | United States Virgin Islands | 84.2 (73.3-97.4) | 81.1 (70.9-92.5) | 62.4 (52.2-73.4) | 66.9 (57.9-76) | -0.71 (-3.07-1.7) |
| Acute hepatitis E | American Samoa | 125.1 (101-157) | 216.7 (178-263.5) | 102.6 (84.3-124.5) | 209.5 (172.9-249.9) | -0.45 (-2.97-2.13) |
| Acute hepatitis E | Antigua and Barbuda | 155.8 (124.9-191) | 244.3 (198.3-298.7) | 182.3 (147.3-221.1) | 235.4 (193.1-284.9) | -0.73 (-3.08-1.68) |
| Acute hepatitis E | Arab Republic of Egypt | 79936 (64946.8-97874.4) | 128.6 (104.8-153.7) | 136291.9 (113156.7-164395.7) | 121.7 (101.3-144.6) | -1.35 (-3.54-0.88) |
| Acute hepatitis E | Argentine Republic | 13882.6 (11605.8-16337.3) | 42 (35.1-49.1) | 18553.2 (15464.6-21787.2) | 41.8 (35-48.3) | -0.42 (-3.45-2.7) |
| Acute hepatitis E | Australia | 14005.7 (11690.2-16391.4) | 89.2 (75.1-104.8) | 20991.2 (17437.4-24610.6) | 89.4 (75-105.4) | -0.51 (-2.65-1.68) |
| Acute hepatitis E | Barbados | 590.3 (477.6-716.5) | 237.5 (191.6-288.6) | 554.1 (456.4-658.8) | 231.3 (185.9-278.8) | -0.57 (-2.97-1.89) |
| Acute hepatitis E | Belize | 570.8 (451.6-708) | 248.3 (200.9-304.1) | 1084.6 (876.9-1323.4) | 241.7 (198-293) | -0.55 (-2.82-1.78) |
| Acute hepatitis E | Bermuda | 126.4 (102.1-153.1) | 236.7 (192.7-285.7) | 108.5 (90.4-130) | 232.6 (191.1-285.3) | -0.63 (-3.06-1.85) |
| Acute hepatitis E | Bolivarian Republic of Venezuela | 20769.2 (17363.6-24282.9) | 104.2 (88-119.3) | 28006.5 (23506.3-32683) | 110.1 (92.1-129.3) | -0.51 (-3.05-2.1) |
| Acute hepatitis E | Bosnia and Herzegovina | 10165.1 (8292.1-12401.5) | 241.8 (197.5-295.8) | 5697.6 (4724.8-6833.4) | 230.1 (186.5-276.5) | -0.57 (-3.15-2.07) |
| Acute hepatitis E | Brunei Darussalam | 427.9 (350.4-519.4) | 148.1 (122.7-176.4) | 570.1 (467.9-686.5) | 146.3 (120.5-176.9) | -0.81 (-2.87-1.29) |
| Acute hepatitis E | Burkina Faso | 27629.9 (22245.4-34745.2) | 221.8 (180.8-272.1) | 63725.3 (50389.5-78863.1) | 221 (180-268.8) | -0.66 (-2.95-1.69) |
| Acute hepatitis E | Canada | 22298.9 (18612.8-26133.4) | 89.5 (74.7-104.7) | 30259.7 (25204.9-35684.7) | 90.2 (75.7-106.7) | -0.24 (-2.51-2.09) |
| Acute hepatitis E | Central African Republic | 10901.6 (8726.5-13526.3) | 306.8 (250.6-372.9) | 21365.9 (17147-26401.2) | 307.9 (251.6-370.5) | -0.46 (-2.59-1.73) |
| Acute hepatitis E | Commonwealth of Dominica | 198.6 (160.6-239.6) | 247.8 (201.8-297.9) | 142.8 (116.6-173.7) | 237.5 (194-288.8) | -0.6 (-2.94-1.79) |
| Acute hepatitis E | Commonwealth of the Bahamas | 670.6 (543.3-828.5) | 238.9 (195.8-291.1) | 825.5 (664.7-1010.1) | 234.2 (188.5-284) | -0.6 (-2.92-1.79) |
| Acute hepatitis E | Cook Islands | 45.1 (36.7-55.9) | 213.6 (175-259.8) | 31.9 (26.4-38.9) | 207.4 (170.7-251.5) | -0.78 (-3.18-1.68) |
| Acute hepatitis E | Czech Republic | 20675.9 (17017.2-24857.6) | 230.4 (186.9-281.4) | 18454 (15354.9-22197.2) | 227 (188.3-280.1) | -0.45 (-2.68-1.82) |
| Acute hepatitis E | Democratic People's Republic of Korea | 58803.9 (46419.5-71825.4) | 281.4 (223.2-345.7) | 56889.8 (45900.8-68231.4) | 269.7 (217.4-325.7) | -0.67 (-3.12-1.86) |
| Acute hepatitis E | Democratic Republic of Sao Tome and Principe | 305.4 (249.8-377.7) | 199 (165.6-239) | 472.8 (379.5-590.7) | 194.3 (160.5-239.1) | -0.69 (-3.1-1.79) |
| Acute hepatitis E | Democratic Republic of the Congo | 143886.7 (116230.9-178959.7) | 285.5 (238-347.1) | 322678.7 (257254.6-397272.5) | 282.5 (229.6-343.5) | -0.35 (-2.52-1.87) |
| Acute hepatitis E | Democratic Republic of Timor-Leste | 1834.6 (1482.9-2219.5) | 192.1 (157.3-227.8) | 3045.2 (2456.8-3693.4) | 190 (156.3-226.2) | -0.54 (-2.76-1.73) |
| Acute hepatitis E | Democratic Socialist Republic of Sri Lanka | 34938.4 (28345.2-42346.5) | 191.3 (157.6-229.8) | 37282 (30945-44160.4) | 185.5 (153.3-219.9) | -0.61 (-2.68-1.5) |
| Acute hepatitis E | Dominican Republic | 20707 (16544.3-25402.3) | 248.7 (201.9-301.9) | 26415.1 (21461.3-32161.8) | 241.7 (196.3-293.1) | -0.49 (-2.76-1.84) |
| Acute hepatitis E | Eastern Republic of Uruguay | 2345.2 (1973.2-2753.2) | 76.1 (64.2-89.6) | 2364.6 (1971.3-2788.8) | 73.7 (61.8-86.6) | -0.48 (-3.3-2.42) |
| Acute hepatitis E | Federal Democratic Republic of Ethiopia | 232469.5 (189961.1-287243.8) | 338.6 (277.5-411.5) | 453780.4 (367399.2-561511.9) | 332.2 (275.6-402.5) | -0.9 (-3.11-1.35) |
| Acute hepatitis E | Federal Democratic Republic of Nepal | 68741.9 (54638.5-84294.9) | 287.4 (233.1-349.1) | 92968.2 (74116.9-115431.7) | 281 (226-343.6) | -1.14 (-3.81-1.6) |
| Acute hepatitis E | Federal Republic of Germany | 51626.4 (42477.6-61144.4) | 70.9 (59.4-83.9) | 57500.8 (47215.6-68775.3) | 70 (59-82.6) | -0.26 (-2.49-2.02) |
| Acute hepatitis E | Federal Republic of Nigeria | 275337.5 (222909.3-338204.3) | 247.4 (204.6-297.3) | 718853.8 (580999.3-886740.2) | 247.8 (206.3-297.6) | -1.48 (-3.87-0.98) |
| Acute hepatitis E | Federal Republic of Somalia | 31875.8 (25582.3-39799.2) | 295.2 (240.9-360.2) | 85477.9 (67259.7-107137.8) | 291.2 (235.1-358.4) | -0.37 (-2.84-2.15) |
| Acute hepatitis E | Federated States of Micronesia | 299.6 (239.7-370.9) | 226.5 (186.6-272) | 228.1 (184.5-278.2) | 215.8 (177.1-258.4) | -0.67 (-2.87-1.57) |
| Acute hepatitis E | Federative Republic of Brazil | 156129.1 (129446.4-185978.6) | 100.9 (84.8-117.3) | 207209.6 (174655.8-239228.6) | 101.5 (85.3-117.6) | -1.04 (-3.34-1.31) |
| Acute hepatitis E | French Republic | 33949.7 (28190-39660.2) | 61.1 (51-71.2) | 40763.4 (34210-48198.7) | 60.5 (50.5-71.7) | -0.51 (-3.01-2.05) |
| Acute hepatitis E | Gabonese Republic | 3403.5 (2788.7-4209.9) | 279.1 (232.1-337.7) | 5638.1 (4525.2-6932.2) | 272.2 (221.7-331) | -0.6 (-2.52-1.36) |
| Acute hepatitis E | Georgia | 10872.4 (8983.8-13059.3) | 209.3 (172.8-251.4) | 6391.2 (5296.5-7582.7) | 205.4 (169.1-247.5) | -0.46 (-2.44-1.56) |
| Acute hepatitis E | Grand Duchy of Luxembourg | 245.7 (202.5-288.3) | 71.1 (59.4-83.9) | 412.7 (340-489) | 70.5 (59.1-82.9) | -0.68 (-2.91-1.6) |
| Acute hepatitis E | Greenland | 47.5 (39.7-56.1) | 90.9 (76.8-106.5) | 45.4 (37.9-53.1) | 90.5 (75.1-106.7) | -0.28 (-2.74-2.25) |
| Acute hepatitis E | Grenada | 246.9 (201.5-302.4) | 247.2 (204-300.4) | 221.4 (179.7-268.9) | 236.7 (191.8-287.7) | -0.81 (-3.1-1.53) |
| Acute hepatitis E | Guam | 303.6 (246.1-364.9) | 210.9 (173.1-251.5) | 295.5 (242.3-354.3) | 206.5 (168.2-250.4) | -0.46 (-2.98-2.12) |
| Acute hepatitis E | Hashemite Kingdom of Jordan | 5420.8 (4408.3-6724.2) | 124.3 (103.6-146.9) | 14990.6 (12352.2-18133.1) | 120.7 (101.2-144.9) | -0.82 (-3.26-1.67) |
| Acute hepatitis E | Hellenic Republic | 6487.5 (5533.6-7549.6) | 67.9 (58.7-77.8) | 6996 (5697.7-8374.3) | 71.7 (60.2-84.3) | -0.45 (-3.13-2.3) |
| Acute hepatitis E | Hungary | 20779.1 (17319.8-24989.5) | 235.9 (194.9-285.5) | 16290.8 (13584.7-19259.8) | 227.4 (185.8-272.6) | -0.19 (-2.35-2.02) |
| Acute hepatitis E | Independent State of Papua New Guinea | 12542.6 (10080.1-15626) | 246.7 (202-303.5) | 29828.6 (23603.5-36750.3) | 244.8 (198.2-299.4) | -0.42 (-2.85-2.06) |
| Acute hepatitis E | Independent State of Samoa | 445.7 (355.9-559.6) | 213.5 (175.6-258.2) | 506.3 (415.1-626.4) | 207 (169.9-251.8) | -0.26 (-2.65-2.2) |
| Acute hepatitis E | Ireland | 2539.7 (2128-3012.3) | 71.7 (60.2-85) | 3304 (2739.7-3874.2) | 70.4 (59.4-82.7) | -0.43 (-2.8-2) |
| Acute hepatitis E | Islamic Republic of Afghanistan | 16556.9 (13243.5-20574.9) | 143.2 (118.1-172.7) | 53294.5 (42739.9-66507.2) | 140.8 (117-170.4) | -1.33 (-4.18-1.6) |
| Acute hepatitis E | Islamic Republic of Iran | 118042.1 (96305.9-145353.6) | 169.8 (140-202) | 130933.1 (107520.1-154573.5) | 166.6 (137.3-197.5) | -1.14 (-3.65-1.44) |
| Acute hepatitis E | Islamic Republic of Mauritania | 5024.2 (4067.5-6219.4) | 198.2 (163.3-238.6) | 10511.1 (8565.4-12938.4) | 197.8 (164.2-237.1) | -1.08 (-3.72-1.64) |
| Acute hepatitis E | Islamic Republic of Pakistan | 457862.8 (369558.2-563188.5) | 325.3 (269.8-397.1) | 869195.3 (696068.7-1062281.4) | 320 (260.5-385.2) | -0.99 (-3.17-1.25) |
| Acute hepatitis E | Jamaica | 6593 (5249.9-8163.8) | 246.5 (199.1-303.6) | 6200.2 (5008.1-7539.8) | 239 (193.2-290.5) | -0.58 (-2.94-1.84) |
| Acute hepatitis E | Japan | 175876.7 (148794.6-207894.8) | 170 (140.8-200.9) | 163335.2 (138840.8-191808.6) | 169.1 (139.9-200) | -0.62 (-2.67-1.47) |
| Acute hepatitis E | Kingdom of Bahrain | 661.6 (527.4-811.1) | 124.2 (102.8-147.6) | 1661.2 (1340.4-2042.1) | 121.5 (101.3-144.6) | -0.57 (-2.98-1.9) |
| Acute hepatitis E | Kingdom of Belgium | 12226.8 (10101.7-14475.6) | 152.9 (123.6-185.1) | 13726.7 (11437-16413.4) | 151.3 (121.5-185) | -0.38 (-2.58-1.87) |
| Acute hepatitis E | Kingdom of Bhutan | 806.8 (692.5-922.5) | 113.7 (97.7-128.7) | 1043.3 (847.6-1267.7) | 140 (116-167.5) | -0.7 (-3.16-1.82) |
| Acute hepatitis E | Kingdom of Cambodia | 24768.6 (20187.1-30571.8) | 192 (159.4-233.6) | 32772.6 (26754-40189.3) | 187.7 (154.6-227.3) | -1.93 (-4.29-0.49) |
| Acute hepatitis E | Kingdom of Denmark | 3353.9 (2785.1-3954) | 70.9 (59.3-83.3) | 3931.1 (3232.8-4679.6) | 70.2 (58.6-82.7) | -0.05 (-2.19-2.13) |
| Acute hepatitis E | Kingdom of Eswatini | 2263.9 (1818.2-2782.8) | 213.2 (175.7-254.6) | 2777.9 (2265-3420.8) | 212.1 (175.6-256.7) | -0.63 (-3.01-1.8) |
| Acute hepatitis E | Kingdom of Lesotho | 4117.9 (3334.7-5069.6) | 214.8 (177.6-258.9) | 4483.8 (3591.7-5454.1) | 215.9 (177.6-258.2) | -0.38 (-2.78-2.08) |
| Acute hepatitis E | Kingdom of Morocco | 36414 (29896.2-44265.9) | 128.9 (106.3-154.2) | 44392 (36514.8-52710.6) | 123.3 (102.9-146.6) | -0.53 (-2.93-1.92) |
| Acute hepatitis E | Kingdom of Norway | 3355.5 (2822.1-3932.1) | 84.5 (70.5-98.8) | 4185.3 (3480.4-4889.3) | 82.9 (70.4-97) | -0.57 (-3.02-1.95) |
| Acute hepatitis E | Kingdom of Saudi Arabia | 45720.2 (36970.8-55976.8) | 234.2 (193.7-284.1) | 63704.6 (48771.6-79604.6) | 191.9 (150.6-236) | -1.12 (-3.09-0.89) |
| Acute hepatitis E | Kingdom of Spain | 23358.3 (19418.2-27534.9) | 65.4 (55.3-77.1) | 27371.7 (22261.1-33022.9) | 61.4 (51.4-72.2) | -0.54 (-2.94-1.93) |
| Acute hepatitis E | Kingdom of Sweden | 6747.4 (5652.4-7899.4) | 83.5 (70.1-97.9) | 8317.1 (6854.1-9686.1) | 83.1 (70.3-97.9) | -0.08 (-2.33-2.23) |
| Acute hepatitis E | Kingdom of Thailand | 102354.2 (82172.3-127042.1) | 175.2 (144.5-211.3) | 88695.5 (74176.1-105343) | 170.1 (141.8-202.7) | -0.43 (-2.66-1.84) |
| Acute hepatitis E | Kingdom of the Netherlands | 8175 (6733.5-9618.8) | 58.9 (48.8-69.4) | 10291.6 (8366.7-12376.9) | 61.4 (51.2-72.4) | -0.23 (-2.69-2.28) |
| Acute hepatitis E | Kingdom of Tonga | 269.5 (217-327) | 220.5 (179.2-261.4) | 256.1 (208.5-308.6) | 212 (173.9-252.3) | -1.07 (-3.54-1.46) |
| Acute hepatitis E | Kyrgyz Republic | 10920.6 (8906.2-13390.2) | 213.9 (176.9-258.2) | 15352.6 (12601.7-18581.2) | 210.1 (174.1-251.9) | -0.41 (-2.34-1.57) |
| Acute hepatitis E | Lao People's Democratic Republic | 9865.2 (8108.9-12092.8) | 191.8 (160.6-232.2) | 14722.6 (11918.3-17661.7) | 190.2 (157.4-226.6) | -1.02 (-3.31-1.33) |
| Acute hepatitis E | Lebanese Republic | 3311 (2754.3-3974.7) | 105 (87.5-124.8) | 5422.1 (4481.9-6391.9) | 102.6 (85.4-120.6) | -0.49 (-3.12-2.22) |
| Acute hepatitis E | Malaysia | 32929.2 (26876.2-40226.8) | 165.3 (137.4-198.8) | 48577.9 (39943.4-58735.9) | 162.5 (134.5-193.2) | -0.71 (-2.81-1.44) |
| Acute hepatitis E | Mongolia | 5675.4 (4566.1-6898.1) | 216.8 (177.9-259.6) | 7487.7 (6223.4-9140.7) | 214.2 (178.7-260) | -0.49 (-2.47-1.54) |
| Acute hepatitis E | Montenegro | 1410.3 (1165.6-1721.4) | 235.3 (194.3-285.6) | 1147.3 (951.5-1373.2) | 230.3 (188-279) | -0.41 (-2.84-2.09) |
| Acute hepatitis E | New Zealand | 3370.5 (2814.6-3988.6) | 104.8 (87-123.4) | 4952.4 (4141.7-5815.4) | 106.2 (87.7-125.5) | -0.45 (-2.63-1.77) |
| Acute hepatitis E | North Macedonia | 4538.4 (3762.2-5538.1) | 236 (194.6-287.2) | 3752.4 (3101.3-4560.8) | 228.7 (187.3-284.2) | -0.49 (-2.9-1.97) |
| Acute hepatitis E | Northern Mariana Islands | 95.1 (75-115) | 210.5 (171.4-254.5) | 88.8 (73.6-106.7) | 207.5 (170.8-252.4) | -0.4 (-2.94-2.21) |
| Acute hepatitis E | Palestine | 3193.9 (2592.7-3964.5) | 129.2 (106.5-154) | 6895.8 (5556.5-8512.8) | 123.7 (102.2-148.9) | -0.54 (-2.92-1.89) |
| Acute hepatitis E | People's Democratic Republic of Algeria | 36627 (29732-45164.6) | 125.9 (104.3-150) | 53759.8 (44287.8-64829.4) | 121.2 (100.3-145.4) | -0.39 (-2.8-2.09) |
| Acute hepatitis E | People's Republic of Bangladesh | 636052.5 (522509.2-763184.2) | 440.7 (365.1-524.7) | 731713.4 (604822-884543.2) | 433 (359.6-519.8) | -1.28 (-3.86-1.38) |
| Acute hepatitis E | People's Republic of China | 5049748.3 (4038586.2-6218765.6) | 412.7 (336.9-502.6) | 3900352.3 (3209997.8-4724583.5) | 364 (297.4-447.6) | -1.62 (-4.05-0.88) |
| Acute hepatitis E | Plurinational State of Bolivia | 17713.2 (14403.2-21832.4) | 227.4 (188.9-274.1) | 26681.1 (21733-32490.9) | 220.9 (181-267.5) | -0.28 (-2.74-2.24) |
| Acute hepatitis E | Portuguese Republic | 6638.5 (5540.1-7845.9) | 70.8 (59.5-83.6) | 6997.9 (5635.9-8377.5) | 68.9 (58.2-82.2) | -0.63 (-2.98-1.78) |
| Acute hepatitis E | Principality of Andorra | 33.1 (27.3-39.3) | 70.9 (59.7-84.1) | 52.2 (42.6-62.6) | 69.9 (58.6-82) | -0.55 (-2.75-1.7) |
| Acute hepatitis E | Principality of Monaco | 20.2 (16.3-24.4) | 71.4 (60.1-84.9) | 26 (20.9-31.1) | 69.9 (58.5-82) | -0.53 (-2.8-1.79) |
| Acute hepatitis E | Puerto Rico | 8525.5 (6894.5-10371.7) | 236.5 (192-286) | 5971.8 (4840.9-7141.2) | 232 (188.2-279.4) | -0.81 (-3.15-1.59) |
| Acute hepatitis E | Republic of Albania | 8864.8 (7161.8-10901.4) | 242.8 (198.2-296.2) | 4981.1 (4086.6-5916.7) | 235.9 (193.4-283.9) | -0.69 (-3.21-1.89) |
| Acute hepatitis E | Republic of Angola | 37637.3 (30558.1-46310.1) | 280.5 (230.4-338.5) | 120474.8 (97599.9-149717.1) | 276.9 (225.7-334.7) | -0.92 (-3.1-1.3) |
| Acute hepatitis E | Republic of Armenia | 7393 (6033.8-8930.3) | 209.1 (172.2-251.1) | 5195.4 (4347.8-6114.7) | 202.4 (168.2-242.4) | -0.36 (-2.33-1.64) |
| Acute hepatitis E | Republic of Austria | 5101.8 (4242.3-6011.8) | 71.5 (60-84.3) | 5946.9 (4857.8-7076) | 70.7 (59.6-83.7) | -0.6 (-2.91-1.77) |
| Acute hepatitis E | Republic of Azerbaijan | 17111.8 (13878.1-20924.1) | 215.3 (176.7-261.3) | 19537.3 (16080.9-23338.5) | 207.6 (172-247.4) | -0.35 (-2.3-1.64) |
| Acute hepatitis E | Republic of Belarus | 13278.4 (11080-15862.1) | 136.7 (113.6-162.4) | 10620.7 (8777.1-12461.8) | 133 (111.2-158.4) | -0.72 (-3.05-1.67) |
| Acute hepatitis E | Republic of Benin | 13391 (10769.3-16740.2) | 211.2 (174-255.4) | 35572.3 (28136.7-44777.2) | 209.9 (172.6-259.1) | -0.63 (-2.94-1.73) |
| Acute hepatitis E | Republic of Botswana | 3588.8 (2847.1-4524.9) | 214 (173-263.9) | 5094.8 (4096.9-6172.7) | 209.3 (171.7-252) | -0.77 (-3.1-1.61) |
| Acute hepatitis E | Republic of Bulgaria | 17058.9 (14221.7-20566) | 234.3 (194.2-283.2) | 11373.7 (9464.3-13546.4) | 227.6 (188.4-275.9) | -0.72 (-3.28-1.9) |
| Acute hepatitis E | Republic of Burundi | 19752.8 (16656.6-23214.4) | 269.2 (231.6-316.1) | 48767.2 (39070.5-60666.5) | 284.8 (233-345.6) | -0.45 (-2.42-1.55) |
| Acute hepatitis E | Republic of Cabo Verde | 880.2 (700.6-1095.6) | 200 (162.9-242.6) | 1052.9 (850.8-1282.1) | 193.5 (157.7-231.7) | -0.73 (-3.06-1.65) |
| Acute hepatitis E | Republic of Cameroon | 27726.9 (22341.1-34296.3) | 209.9 (174-256.5) | 80964.2 (64147-99444.5) | 208.7 (170-249.5) | -0.55 (-2.75-1.7) |
| Acute hepatitis E | Republic of Chad | 17252.5 (13766-21366) | 221.6 (181.3-268.4) | 52422.2 (41538-65426.8) | 220.5 (179.4-265.1) | -0.45 (-2.87-2.02) |
| Acute hepatitis E | Republic of Chile | 11364.4 (9524-13422.4) | 84.7 (72-98.8) | 13583.4 (11357.9-16011.1) | 80.3 (67.9-95.4) | -0.38 (-3.01-2.32) |
| Acute hepatitis E | Republic of Colombia | 46671.3 (37571.4-56090.8) | 132.7 (109.8-156.8) | 57760.8 (47984.9-69598.9) | 127.4 (107.1-151.7) | -0.92 (-3.39-1.62) |
| Acute hepatitis E | Republic of Costa Rica | 4356.6 (3604-5218.3) | 130.8 (109.7-154.4) | 5469.7 (4560.7-6510.7) | 125.3 (104.3-148.3) | -0.59 (-3.06-1.95) |
| Acute hepatitis E | Republic of C么te d'Ivoire | 33398.7 (26952.1-41394.1) | 214.3 (176.5-259) | 71510.9 (57373.4-88495.6) | 213.2 (174.6-260.9) | -0.66 (-3-1.74) |
| Acute hepatitis E | Republic of Croatia | 9584.8 (7893.1-11606) | 231.4 (189.1-282.3) | 7097.4 (5898.6-8624.5) | 226.5 (186.4-276.6) | -0.78 (-3.09-1.6) |
| Acute hepatitis E | Republic of Cuba | 25892.8 (20685.6-31484.4) | 243.6 (197.2-293) | 21000.6 (17332.4-25137.1) | 235.9 (191.3-286.1) | -0.82 (-3.26-1.67) |
| Acute hepatitis E | Republic of Cyprus | 530.6 (443.3-628.5) | 71.5 (59.9-84.1) | 858.3 (702.2-1015.7) | 70.5 (58.1-83.1) | -0.74 (-3.01-1.58) |
| Acute hepatitis E | Republic of Djibouti | 1553.4 (1247.3-1909.8) | 293.9 (241.1-355.3) | 3940.3 (3197-4799.4) | 287.6 (235.7-348.6) | -0.35 (-2.55-1.89) |
| Acute hepatitis E | Republic of Ecuador | 26886.3 (21556.1-32614.1) | 228.2 (186.5-274.4) | 39814.9 (32704.5-48579.6) | 220.8 (182.3-268.4) | -0.17 (-2.7-2.42) |
| Acute hepatitis E | Republic of El Salvador | 8261.4 (6635.9-10085.2) | 136.7 (112.6-163.3) | 8401.7 (6996.9-9921.6) | 130.2 (108.6-153.1) | -0.75 (-3.18-1.75) |
| Acute hepatitis E | Republic of Equatorial Guinea | 1557.4 (1255.6-1922.3) | 281.1 (231.6-344.1) | 4964.5 (3895.8-6062) | 267.2 (215.7-321.1) | -0.63 (-2.56-1.34) |
| Acute hepatitis E | Republic of Estonia | 1944.7 (1634.2-2311.7) | 134.5 (113-159.1) | 1504.8 (1260.5-1791.8) | 131.8 (110.8-156.9) | -0.63 (-2.86-1.66) |
| Acute hepatitis E | Republic of Fiji | 1929.9 (1547.3-2348.8) | 219.8 (179.3-263.5) | 1967.7 (1596.2-2403.4) | 211.4 (172.6-256.2) | -0.63 (-2.99-1.79) |
| Acute hepatitis E | Republic of Finland | 3295.1 (2737.9-3869.8) | 71.2 (59.5-83.1) | 3759.6 (3059.4-4488) | 69.9 (58.6-82.6) | -0.08 (-2.13-2.02) |
| Acute hepatitis E | Republic of Ghana | 34917 (28449.5-42021.5) | 192.1 (159.3-228.4) | 76576.8 (61879.5-94170.9) | 195.7 (161.4-237.9) | -1.01 (-3.4-1.45) |
| Acute hepatitis E | Republic of Guatemala | 14220.4 (11575.3-17290.9) | 138.2 (116-162.8) | 21425.9 (17569.1-25857.4) | 131.9 (109.6-155.7) | -0.59 (-2.92-1.79) |
| Acute hepatitis E | Republic of Guinea | 16488.5 (13408.3-20388.8) | 221.1 (181.3-268.7) | 36985.6 (29315.3-45586.2) | 218.4 (178-263.7) | -0.63 (-3.05-1.86) |
| Acute hepatitis E | Republic of Guinea-Bissau | 2873.9 (2305.2-3533.4) | 220.2 (181.8-265.3) | 5665.4 (4544.9-7022.3) | 220.2 (182.2-269.8) | -0.87 (-3.49-1.83) |
| Acute hepatitis E | Republic of Guyana | 2245.5 (1787.3-2750.9) | 248.1 (201-300) | 1856.5 (1478-2244.6) | 240.2 (192.2-290.9) | -0.6 (-2.8-1.65) |
| Acute hepatitis E | Republic of Haiti | 19124.9 (15284.6-23708) | 251.8 (204.4-306) | 34927.2 (27872.7-42709.3) | 247.7 (200.2-301.8) | -0.33 (-2.47-1.85) |
| Acute hepatitis E | Republic of Honduras | 7698.9 (6236.7-9376) | 135.4 (112.6-160.6) | 13693.6 (11145.6-16454.5) | 130.4 (108.1-154.8) | -0.68 (-2.98-1.67) |
| Acute hepatitis E | Republic of Iceland | 175.6 (146.6-207.6) | 71.4 (59.4-84.3) | 234.6 (195.7-276.3) | 70.2 (58.8-82.9) | -0.07 (-2.17-2.06) |
| Acute hepatitis E | Republic of India | 3910641.6 (3184843.5-4799843.9) | 384.8 (317.4-463.8) | 5446427.4 (4453991.6-6579339) | 389.4 (321.2-467.3) | -1.13 (-3.66-1.46) |
| Acute hepatitis E | Republic of Indonesia | 458391.6 (372463.3-557771.4) | 218.8 (182.1-261.4) | 557765.1 (459487.7-668521.2) | 216.7 (178.5-258.4) | -0.97 (-3.27-1.39) |
| Acute hepatitis E | Republic of Iraq | 27699.1 (22396.7-33768.3) | 127.5 (106.2-151.9) | 53039.1 (43555.6-64667.3) | 123.4 (102.7-146.8) | -0.4 (-2.74-2) |
| Acute hepatitis E | Republic of Italy | 48928.8 (40905.9-57632.6) | 95.3 (79.8-110.3) | 51042.1 (42063.1-61234.1) | 94.6 (79.2-110.9) | -1.34 (-3.48-0.85) |
| Acute hepatitis E | Republic of Kazakhstan | 36887.4 (30214.1-45182) | 214.2 (177-261.8) | 38435.4 (31928.4-46180.6) | 206.5 (171.4-247.5) | -0.34 (-2.44-1.81) |
| Acute hepatitis E | Republic of Kenya | 107032.1 (86632.7-132955.2) | 336 (276.3-406.4) | 196172 (158376.8-242149.1) | 328.5 (269.3-396.6) | -0.62 (-2.84-1.64) |
| Acute hepatitis E | Republic of Kiribati | 199.6 (161.3-239.3) | 223.6 (184.6-267.4) | 295.3 (238.2-357.7) | 220 (181.1-265.7) | -0.75 (-3.32-1.88) |
| Acute hepatitis E | Republic of Korea | 50508 (41323-61740.6) | 118.6 (98.4-142.1) | 43603.3 (36155.3-52333.3) | 111.6 (93.3-132.7) | -0.92 (-3.45-1.68) |
| Acute hepatitis E | Republic of Latvia | 3284.6 (2767.8-3938.2) | 135.2 (112.4-161.8) | 2144.2 (1793.1-2531.1) | 132.7 (110.6-156.6) | -0.58 (-2.95-1.84) |
| Acute hepatitis E | Republic of Liberia | 6802.6 (5512-8321.5) | 219.2 (180.6-265.1) | 14294.8 (11510.3-17515.4) | 219.3 (180.1-264.8) | -0.56 (-3.01-1.95) |
| Acute hepatitis E | Republic of Lithuania | 4605.5 (3833.8-5441.7) | 134.7 (112.4-159.7) | 3091.5 (2603.6-3690.2) | 132.4 (110.4-157.4) | -0.6 (-2.85-1.7) |
| Acute hepatitis E | Republic of Madagascar | 44041.5 (35214.8-54682) | 282.3 (229.2-340.8) | 99560 (80182.3-123898.3) | 279.1 (228.8-340.7) | -0.48 (-2.83-1.93) |
| Acute hepatitis E | Republic of Malawi | 34453.5 (27970.1-43350.7) | 268.4 (219.9-327.4) | 64541.6 (50818.1-80918.6) | 262.6 (211.3-321.3) | -0.75 (-2.93-1.48) |
| Acute hepatitis E | Republic of Maldives | 494.6 (392.9-612.7) | 175.2 (142.5-209.5) | 762.6 (602.4-927) | 169.2 (138.4-201.1) | -1.05 (-3.11-1.06) |
| Acute hepatitis E | Republic of Mali | 24274.5 (19565.5-29546.5) | 219.8 (181.3-265.2) | 68605.9 (54942.6-84135) | 218.4 (180.6-263) | -0.51 (-2.71-1.75) |
| Acute hepatitis E | Republic of Malta | 248.3 (206.9-292.1) | 70.9 (59.5-83.1) | 297.8 (242.4-358.8) | 70.6 (58.8-83.1) | -0.61 (-3.06-1.9) |
| Acute hepatitis E | Republic of Mauritius | 1855.3 (1496.2-2254.9) | 165.2 (134.5-197.6) | 1678.1 (1393.1-2017.4) | 160.8 (133.5-192.6) | -0.8 (-2.88-1.32) |
| Acute hepatitis E | Republic of Moldova | 10566 (8729.6-12947.9) | 242.7 (199.9-297.7) | 6146.9 (5128.2-7328.5) | 237.9 (194.3-290.2) | -0.7 (-2.73-1.37) |
| Acute hepatitis E | Republic of Mozambique | 47837.2 (38599.6-58419) | 274.4 (223.5-328.6) | 111565.7 (89115.9-139233.1) | 271.1 (222-329.6) | -0.55 (-2.94-1.89) |
| Acute hepatitis E | Republic of Namibia | 3711.9 (3002.6-4591) | 212.8 (176.7-259.1) | 5645.7 (4523.8-6860.7) | 211.2 (173.3-253.6) | -0.47 (-2.9-2.02) |
| Acute hepatitis E | Republic of Nauru | 26.9 (21.6-32.8) | 216.7 (176.6-260.9) | 26.3 (21.4-31.8) | 210.3 (173.6-252.4) | -0.64 (-3-1.78) |
| Acute hepatitis E | Republic of Nicaragua | 6506.5 (5291.5-7996.2) | 137.9 (113.8-162.5) | 8804.3 (7305.9-10511) | 131.1 (109.9-154.7) | -0.65 (-3.07-1.82) |
| Acute hepatitis E | Republic of Niue | 5.5 (4.5-6.7) | 214.6 (177.9-258) | 3.1 (2.6-3.8) | 209.4 (173.7-249.6) | -0.83 (-3.2-1.6) |
| Acute hepatitis E | Republic of Palau | 33.9 (27.4-41.5) | 213.2 (175.4-256.6) | 29.9 (24.6-35.9) | 209.3 (171.6-253.5) | -0.63 (-3.04-1.83) |
| Acute hepatitis E | Republic of Panama | 3460.9 (2827.2-4221.1) | 134.9 (111.5-161.8) | 5390.6 (4534.2-6416.6) | 128.2 (107.8-152.5) | -0.52 (-3.1-2.12) |
| Acute hepatitis E | Republic of Paraguay | 4038.5 (3344.8-4795.4) | 90.1 (76.4-105) | 6088.4 (5043.7-7277.4) | 86.5 (72-101.9) | -0.59 (-3.01-1.89) |
| Acute hepatitis E | Republic of Peru | 58631.9 (47624.8-72119.6) | 230.5 (189.6-279.2) | 78043.8 (64359.8-94148.5) | 221.9 (182.5-267.8) | -0.42 (-2.96-2.19) |
| Acute hepatitis E | Republic of Poland | 97724.7 (81431.1-116901.8) | 275.1 (227.7-330) | 76576.3 (64470.5-90776.5) | 265.8 (221.4-320.2) | -1.21 (-3.51-1.14) |
| Acute hepatitis E | Republic of Rwanda | 25273.4 (20020.3-31089.6) | 265.4 (216.4-322.9) | 40904.1 (32740.1-50773.7) | 261.6 (211.8-318.1) | -1.15 (-3.3-1.05) |
| Acute hepatitis E | Republic of San Marino | 10.4 (8.8-12.5) | 44.1 (37.3-51.7) | 17.4 (14.3-20.8) | 51.1 (42.9-59.5) | -0.37 (-2.77-2.08) |
| Acute hepatitis E | Republic of Senegal | 19856.3 (15998.5-24845.8) | 203.5 (168.1-251) | 37752.5 (30429.3-45927.7) | 200.8 (164.8-242.2) | -0.51 (-3.03-2.07) |
| Acute hepatitis E | Republic of Serbia | 20056.7 (16449.7-24136.9) | 234.8 (192.2-285.7) | 15449.9 (12862-18453.3) | 228.2 (189.1-276.7) | -0.43 (-2.94-2.15) |
| Acute hepatitis E | Republic of Seychelles | 131 (108.2-157.7) | 168 (138.8-200) | 157.3 (130.2-185.3) | 167 (137.3-197.5) | -0.76 (-3-1.53) |
| Acute hepatitis E | Republic of Sierra Leone | 12116.3 (9700.4-14848.4) | 236.7 (193-287.7) | 25534.4 (20255.6-31837.7) | 237.2 (191.7-287.8) | -0.71 (-3.04-1.67) |
| Acute hepatitis E | Republic of Singapore | 5424.9 (4670.8-6312.2) | 204.4 (178-235.1) | 7613.3 (6290.8-8954.5) | 193.4 (158.3-235.2) | -0.69 (-2.76-1.43) |
| Acute hepatitis E | Republic of Slovenia | 3969.7 (3290.7-4772.2) | 231 (190.5-276.9) | 3526.9 (2932.6-4225.2) | 226.2 (184.6-279.4) | -0.61 (-3-1.84) |
| Acute hepatitis E | Republic of South Africa | 93680.5 (76741-114967.2) | 221.3 (183.9-264.9) | 132895.8 (109601.8-161010.3) | 241.4 (199.3-291.8) | -0.64 (-2.9-1.66) |
| Acute hepatitis E | Republic of South Sudan | 22509.1 (17798.4-28179.9) | 293.5 (236.5-358) | 36490 (28924.4-45128) | 290.9 (236.2-348.8) | -0.18 (-2.4-2.08) |
| Acute hepatitis E | Republic of Sudan | 34137.3 (27439.7-41756) | 143.1 (119-172) | 68287.8 (54386.6-84421.4) | 139.8 (114.7-167.6) | -1.25 (-3.87-1.44) |
| Acute hepatitis E | Republic of Suriname | 1051.1 (836.3-1303.5) | 245 (198.8-300.7) | 1299.7 (1067.3-1579.7) | 238.2 (194.4-291.3) | -0.59 (-2.86-1.75) |
| Acute hepatitis E | Republic of Tajikistan | 15377.9 (12271-18807) | 230.8 (190-276.4) | 25558.3 (20649.5-31409) | 227.3 (187.3-276) | -0.33 (-2.25-1.62) |
| Acute hepatitis E | Republic of the Congo | 8728.9 (6997.5-10883.6) | 280.8 (229.2-341.5) | 17062.1 (13699.9-21340.2) | 275.8 (225.2-338) | -0.62 (-2.68-1.48) |
| Acute hepatitis E | Republic of the Gambia | 2774.3 (2236.5-3421.6) | 219.8 (181.7-266.7) | 6403.1 (5177.1-7945.8) | 218.4 (179.7-266) | -0.72 (-3.04-1.66) |
| Acute hepatitis E | Republic of the Marshall Islands | 136.3 (109.2-169.5) | 224 (182.8-269.8) | 126.3 (101.9-154.6) | 214.4 (174.3-259.9) | -0.53 (-2.98-1.98) |
| Acute hepatitis E | Republic of the Niger | 23426.1 (18445.6-29648.7) | 220.6 (181.5-269.7) | 74515.5 (59548.6-93193.6) | 220.8 (182.3-264.8) | -0.6 (-2.99-1.86) |
| Acute hepatitis E | Republic of the Philippines | 166717.1 (135357.9-203339.8) | 222.4 (184.2-265.7) | 255342.6 (209412.5-308767.1) | 219.9 (180.9-263.8) | -0.78 (-3.19-1.7) |
| Acute hepatitis E | Republic of the Union of Myanmar | 87820.6 (70600.8-108049.8) | 191.9 (156.5-232.6) | 105388.4 (85700.4-127726.5) | 189.4 (154.8-228) | -0.6 (-2.65-1.49) |
| Acute hepatitis E | Republic of Trinidad and Tobago | 3215.2 (2553.2-3947) | 243.3 (194.9-296.6) | 2801.9 (2286.2-3383.3) | 235.2 (192.9-283.7) | -0.62 (-2.96-1.77) |
| Acute hepatitis E | Republic of Tunisia | 9867.7 (8096.3-12025.8) | 109.6 (91.6-130.4) | 11662.1 (9622.5-13640.7) | 105.1 (87.6-122.6) | -0.53 (-3-2) |
| Acute hepatitis E | Republic of Turkey | 74486.6 (66433.3-82474.8) | 124 (109.9-137.4) | 103058.9 (84758.9-125972) | 134.3 (110.4-162) | -0.63 (-3.22-2.04) |
| Acute hepatitis E | Republic of Uganda | 67521.1 (54114.6-84098.8) | 288.1 (231.7-350.2) | 163410.9 (131009.8-202554.3) | 283.1 (232.5-340.4) | -0.7 (-2.84-1.5) |
| Acute hepatitis E | Republic of Uzbekistan | 54105.4 (43452-65832.1) | 215.7 (176.1-262.7) | 72244.3 (59462.9-88059.8) | 209.7 (173-253.7) | -0.47 (-2.53-1.64) |
| Acute hepatitis E | Republic of Vanuatu | 438.3 (351.8-547.8) | 225.6 (184-275.2) | 802.1 (649.4-979.1) | 221.8 (182.2-266.8) | -0.43 (-2.84-2.03) |
| Acute hepatitis E | Republic of Yemen | 12902.3 (10862.6-14994.7) | 83.2 (70.7-95.3) | 35086.7 (28319.4-42406.6) | 95.6 (79-112) | -1.27 (-4-1.55) |
| Acute hepatitis E | Republic of Zambia | 27932.4 (22021.6-35165.3) | 264.4 (213.8-322.2) | 64315.8 (51690.9-80012.7) | 260.8 (215.1-317.6) | -0.73 (-2.92-1.52) |
| Acute hepatitis E | Republic of Zimbabwe | 28443.4 (22749.8-35377.6) | 211.9 (175.8-257.7) | 40195.7 (32344.3-49698.7) | 214.3 (176.5-259.6) | -0.29 (-2.95-2.45) |
| Acute hepatitis E | Romania | 51943.3 (43027.7-63155.1) | 244.2 (201.6-296.9) | 33515.3 (28149-40030.2) | 232 (192.4-283.2) | -0.97 (-3.21-1.32) |
| Acute hepatitis E | Russian Federation | 137359.1 (115754.2-159317.4) | 96.4 (81.3-112) | 130447.9 (109377.5-153368.3) | 97.8 (82.6-113.6) | -0.38 (-2.78-2.08) |
| Acute hepatitis E | Saint Kitts and Nevis | 110.7 (88.5-135.7) | 241.5 (195.8-290.7) | 114.7 (93-137.1) | 234.3 (192-288) | -0.84 (-3.12-1.49) |
| Acute hepatitis E | Saint Lucia | 389.6 (311.2-483.5) | 246 (199.3-301.7) | 350.2 (284.2-424.4) | 236.9 (191.2-287.8) | -0.7 (-2.99-1.65) |
| Acute hepatitis E | Saint Vincent and the Grenadines | 317.9 (256.3-391) | 248.7 (205.3-299.1) | 245.9 (199.3-296.1) | 239.2 (193.5-290.3) | -0.65 (-2.98-1.73) |
| Acute hepatitis E | Slovak Republic | 11521.2 (9572.1-13912.4) | 232.6 (192.3-278.2) | 9372.6 (7877.1-11288.2) | 226.5 (187.4-277.1) | -0.6 (-2.96-1.82) |
| Acute hepatitis E | Socialist Republic of Viet Nam | 151353.1 (122927.3-185932.1) | 189.8 (157.2-228.6) | 172186.5 (141611.5-206042.7) | 184.9 (152.6-222.1) | -0.91 (-3.41-1.65) |
| Acute hepatitis E | Solomon Islands | 989 (790.5-1251.7) | 224.5 (184.5-273.8) | 1743.8 (1403.5-2138) | 218.7 (178.5-265) | -0.63 (-3.09-1.89) |
| Acute hepatitis E | State of Eritrea | 13420.3 (10722.8-16388.3) | 294.8 (240.9-358.3) | 22744.8 (18288.6-28065.2) | 287.8 (236.6-350.3) | -0.64 (-2.84-1.61) |
| Acute hepatitis E | State of Israel | 2973.3 (2494.4-3497.4) | 59.1 (49.8-69.2) | 6031.7 (5070.4-7196) | 61.5 (51.3-73.5) | -0.72 (-3.2-1.81) |
| Acute hepatitis E | State of Kuwait | 2218.9 (1784.7-2702.6) | 123.2 (102.2-147.5) | 4892 (3855.7-6001.9) | 120.1 (98.8-142.9) | -0.58 (-3.02-1.92) |
| Acute hepatitis E | State of Libya | 6194 (5048.9-7574.1) | 127 (105.6-151.4) | 7739.3 (6265.3-9358) | 122.4 (101.3-146.4) | -0.57 (-3.05-1.98) |
| Acute hepatitis E | State of Qatar | 558.5 (441.3-683.9) | 124.5 (102.8-148.1) | 3148.7 (2419.1-4038) | 120.9 (100.1-144.8) | -0.6 (-2.99-1.84) |
| Acute hepatitis E | Sultanate of Oman | 2942.7 (2379.8-3612.9) | 129.1 (105.5-153.9) | 5559 (4464.2-6821.3) | 121.7 (100.3-144.8) | -1.4 (-3.92-1.18) |
| Acute hepatitis E | Swiss Confederation | 4942 (4092.3-5784.4) | 80.8 (67.9-94.6) | 6549.1 (5432.1-7637.3) | 80.4 (68.3-94.1) | -0.05 (-2.17-2.13) |
| Acute hepatitis E | Syrian Arab Republic | 19248.1 (15594.3-23686.6) | 126.8 (105.9-150.3) | 16362.9 (13298.5-19704.6) | 121.8 (100.2-144.3) | -0.58 (-3.02-1.93) |
| Acute hepatitis E | Taiwan (Province of China) | 35321.3 (27469.9-43103.4) | 175.1 (138.5-210.2) | 32724.7 (27369.9-39013.8) | 189.8 (157.2-227.7) | -1.15 (-3.49-1.25) |
| Acute hepatitis E | Togolese Republic | 10083.8 (8095.1-12639.7) | 212.5 (174.2-256.9) | 20811.2 (16719.4-25734) | 211.4 (173.6-256.2) | -0.46 (-2.82-1.95) |
| Acute hepatitis E | Tokelau | 4 (3.3-4.9) | 218.1 (178.1-263.6) | 2.9 (2.4-3.5) | 210.3 (173.3-253.7) | -0.7 (-3.13-1.78) |
| Acute hepatitis E | Turkmenistan | 9525.5 (7739.3-11708.8) | 215.7 (178-261.1) | 10961.5 (8990.4-13451.6) | 209.7 (172.7-257.8) | -0.39 (-2.34-1.59) |
| Acute hepatitis E | Tuvalu | 23.7 (19.3-28.5) | 221.4 (181.7-263.7) | 27 (22.1-33) | 211.9 (174.4-257.1) | -0.4 (-2.83-2.08) |
| Acute hepatitis E | Ukraine | 76657.4 (64355.1-91196) | 160.9 (133.2-192.1) | 56284.7 (46764.9-66256.2) | 160.9 (134.2-191.8) | 0.21 (-1.85-2.31) |
| Acute hepatitis E | Union of the Comoros | 1793.7 (1428.5-2239.9) | 293.4 (238.5-357.6) | 2313.1 (1861.8-2867.2) | 286.2 (232.5-350.4) | -0.57 (-2.74-1.66) |
| Acute hepatitis E | United Arab Emirates | 2444 (1947.3-2976.4) | 125 (103.5-148.4) | 9409 (7227.3-12284.8) | 121.5 (100.6-146) | -0.52 (-2.92-1.95) |
| Acute hepatitis E | United Kingdom of Great Britain and Northern Ireland | 43940.7 (37021.3-51539) | 81.8 (69.6-94.7) | 51969 (43361.9-60586.7) | 81.2 (68.8-94.6) | 0.07 (-2.13-2.32) |
| Acute hepatitis E | United Mexican States | 164318.5 (135088.3-199894.3) | 168.9 (141.2-199.8) | 209348.9 (173425.3-250636) | 171.8 (143.3-205.2) | -0.66 (-3.18-1.92) |
| Acute hepatitis E | United Republic of Tanzania | 91547.7 (74734-114723.9) | 268.6 (223.8-326.3) | 191041.6 (155256.8-237402.5) | 262.3 (216.6-322.4) | -0.55 (-2.62-1.57) |
| Acute hepatitis E | United States of America | 246943.1 (207562.7-286130.7) | 104.5 (87.2-121.9) | 316108.5 (267661.5-370570.8) | 106 (88.9-124.5) | -0.49 (-2.81-1.89) |
| Acute hepatitis E | United States Virgin Islands | 258.2 (208.9-314.3) | 240.1 (194.8-294.2) | 153.6 (127-184.8) | 233.8 (189.6-283.2) | -0.71 (-3.07-1.7) |

Table S2. The number of Acute hepatitis deaths and age standardized mortality of Acute hepatitis caused by different reasons from 1990 to 2021.

| cause | location | 1990 | | 2021 | | 1990-2021 |
| --- | --- | --- | --- | --- | --- | --- |
|  |  | Number of deaths  No. ×10^3^ (95% UI) | ASMR per 100,000  No. (95% UI) | Number of deaths  No. ×10^3^ (95% UI) | ASMR per 100,000  No. (95% UI) | EAPC  No. (95% CI) |
| Acute hepatitis | American Samoa | 0.1 (0.1-0.1) | 0.3 (0.2-0.3) | 0.1 (0.1-0.1) | 0.1 (0.1-0.2) | -0.15 (-0.25--0.04) |
| Acute hepatitis | Antigua and Barbuda | 0 (0-0) | 0.1 (0-0.1) | 0 (0-0) | 0 (0-0) | -0.07 (-0.09--0.04) |
| Acute hepatitis | Arab Republic of Egypt | 5589.3 (2389.5-8253.3) | 17.3 (7.2-26.4) | 2435.6 (928.7-4461.3) | 3.7 (1.4-6.6) | -2.56 (-4.13--0.97) |
| Acute hepatitis | Argentine Republic | 38.6 (28.4-79.7) | 0.1 (0.1-0.2) | 11.7 (9.8-13.7) | 0 (0-0) | -0.13 (-0.17--0.09) |
| Acute hepatitis | Australia | 3.1 (2.8-3.5) | 0 (0-0) | 4.1 (3.4-5) | 0 (0-0) | -0.02 (-0.03--0.01) |
| Acute hepatitis | Barbados | 0.5 (0.4-0.6) | 0.2 (0.2-0.2) | 0.4 (0.3-0.5) | 0.1 (0.1-0.1) | -0.11 (-0.19--0.02) |
| Acute hepatitis | Belize | 0.2 (0.1-0.2) | 0.1 (0.1-0.1) | 0.1 (0.1-0.1) | 0 (0-0) | -0.11 (-0.15--0.07) |
| Acute hepatitis | Bermuda | 0 (0-0) | 0 (0-0) | 0 (0-0) | 0 (0-0) | -0.02 (-0.03--0.01) |
| Acute hepatitis | Bolivarian Republic of Venezuela | 16.4 (12.4-25.7) | 0.1 (0.1-0.2) | 20.9 (15.5-28.1) | 0.1 (0.1-0.1) | -0.02 (-0.07-0.03) |
| Acute hepatitis | Bosnia and Herzegovina | 8.4 (5-13.9) | 0.2 (0.1-0.3) | 4.3 (2.6-6.7) | 0.1 (0.1-0.1) | -0.15 (-0.26--0.04) |
| Acute hepatitis | Brunei Darussalam | 4.4 (3.4-5.5) | 2.6 (2-3.3) | 1.4 (1.1-1.8) | 0.3 (0.3-0.4) | -1.79 (-2.35--1.23) |
| Acute hepatitis | Burkina Faso | 350.1 (266.2-465.3) | 5 (3.9-6.4) | 331.4 (206.8-486.2) | 2.2 (1.5-3) | -1.31 (-2.29--0.33) |
| Acute hepatitis | Canada | 10.9 (9.7-12.1) | 0 (0-0) | 7 (5.9-8.4) | 0 (0-0) | -0.05 (-0.07--0.03) |
| Acute hepatitis | Central African Republic | 187.1 (97-338) | 7.4 (4.3-11.3) | 103.6 (54.4-171.3) | 2.5 (1.3-4.1) | -1.7 (-2.69--0.69) |
| Acute hepatitis | Commonwealth of Dominica | 0.1 (0-0.1) | 0.1 (0.1-0.1) | 0 (0-0) | 0 (0-0.1) | -0.07 (-0.11--0.03) |
| Acute hepatitis | Commonwealth of the Bahamas | 0.2 (0.1-0.2) | 0.1 (0.1-0.1) | 0.1 (0.1-0.1) | 0 (0-0) | -0.07 (-0.11--0.04) |
| Acute hepatitis | Cook Islands | 0 (0-0) | 0 (0-0) | 0 (0-0) | 0 (0-0) | -0.04 (-0.05--0.02) |
| Acute hepatitis | Czech Republic | 11.8 (10.1-13.6) | 0.1 (0.1-0.1) | 4.3 (3.3-5.5) | 0 (0-0) | -0.06 (-0.1--0.03) |
| Acute hepatitis | Democratic People's Republic of Korea | 264.6 (141.2-412.2) | 1.4 (0.8-2.2) | 58 (36-88.5) | 0.2 (0.1-0.3) | -1.11 (-1.46--0.76) |
| Acute hepatitis | Democratic Republic of Sao Tome and Principe | 1.8 (1.2-2.5) | 1.9 (1.3-2.7) | 0.5 (0.4-0.8) | 0.4 (0.3-0.5) | -1.31 (-1.78--0.84) |
| Acute hepatitis | Democratic Republic of the Congo | 249.4 (141.4-384.4) | 0.6 (0.4-1.1) | 175.6 (87.9-292.1) | 0.3 (0.1-0.5) | -0.35 (-0.6--0.1) |
| Acute hepatitis | Democratic Republic of Timor-Leste | 4.4 (1.7-9.3) | 0.7 (0.2-1.3) | 2.1 (0.7-4) | 0.2 (0.1-0.4) | -0.61 (-0.81--0.42) |
| Acute hepatitis | Democratic Socialist Republic of Sri Lanka | 77.5 (47-111.6) | 0.6 (0.3-0.8) | 24.3 (13.7-36.8) | 0.1 (0.1-0.1) | -0.61 (-0.77--0.45) |
| Acute hepatitis | Dominican Republic | 6.7 (4.9-9.4) | 0.1 (0.1-0.1) | 7.3 (5.2-10.1) | 0.1 (0-0.1) | -0.08 (-0.14--0.02) |
| Acute hepatitis | Eastern Republic of Uruguay | 1.4 (1.1-1.7) | 0 (0-0.1) | 0.4 (0.3-0.5) | 0 (0-0) | -0.05 (-0.06--0.03) |
| Acute hepatitis | Federal Democratic Republic of Ethiopia | 3120.1 (2150.3-4386.1) | 10.6 (7.5-15.4) | 2886.4 (2088.2-3779.9) | 4.6 (3.3-5.9) | -1.93 (-2.95--0.89) |
| Acute hepatitis | Federal Democratic Republic of Nepal | 1545.8 (864.1-2488) | 7.2 (4.5-11) | 551.9 (390.7-757.8) | 2 (1.4-2.7) | -1.48 (-2.49--0.46) |
| Acute hepatitis | Federal Republic of Germany | 73.5 (61.9-85.1) | 0.1 (0.1-0.1) | 35.1 (28.8-41.5) | 0 (0-0) | -0.07 (-0.09--0.04) |
| Acute hepatitis | Federal Republic of Nigeria | 11977.3 (5277.1-19160) | 16 (6.7-26.9) | 3169.7 (1639.4-4980.5) | 1.8 (1-2.8) | -4.49 (-5.68--3.29) |
| Acute hepatitis | Federal Republic of Somalia | 981.7 (448.3-1671) | 22.1 (10.1-37.6) | 1919.7 (854-3234.7) | 17.9 (8.4-30.7) | -0.59 (-2.19-1.04) |
| Acute hepatitis | Federated States of Micronesia | 0.6 (0.4-1) | 0.8 (0.5-1.3) | 0.2 (0.1-0.3) | 0.2 (0.1-0.3) | -0.65 (-0.85--0.45) |
| Acute hepatitis | Federative Republic of Brazil | 259.5 (229.5-326.4) | 0.2 (0.2-0.3) | 148.3 (134.3-169) | 0.1 (0.1-0.1) | -0.18 (-0.26--0.1) |
| Acute hepatitis | French Republic | 22.7 (20.1-24.9) | 0 (0-0) | 18.8 (15-22.4) | 0 (0-0) | -0.04 (-0.06--0.02) |
| Acute hepatitis | Gabonese Republic | 15 (7.7-27.7) | 2 (1-4) | 2.9 (1.3-6) | 0.2 (0.1-0.5) | -1.26 (-1.67--0.85) |
| Acute hepatitis | Georgia | 24.7 (20.5-29.2) | 0.5 (0.4-0.6) | 1.7 (1.2-2.3) | 0 (0-0.1) | -0.41 (-0.52--0.29) |
| Acute hepatitis | Grand Duchy of Luxembourg | 0.1 (0.1-0.1) | 0 (0-0) | 0.1 (0-0.1) | 0 (0-0) | -0.02 (-0.03--0.01) |
| Acute hepatitis | Greenland | 0 (0-0.1) | 0.1 (0.1-0.2) | 0 (0-0) | 0 (0-0) | -0.15 (-0.19--0.11) |
| Acute hepatitis | Grenada | 0.2 (0.1-0.3) | 0.2 (0.1-0.3) | 0.1 (0-0.1) | 0.1 (0-0.1) | -0.16 (-0.24--0.09) |
| Acute hepatitis | Guam | 0.1 (0.1-0.1) | 0.1 (0.1-0.1) | 0.2 (0.1-0.2) | 0.1 (0.1-0.1) | -0.02 (-0.08-0.04) |
| Acute hepatitis | Hashemite Kingdom of Jordan | 26.5 (16.5-37.6) | 1.4 (0.9-2) | 17.2 (11.9-24.7) | 0.2 (0.2-0.3) | -1.09 (-1.4--0.78) |
| Acute hepatitis | Hellenic Republic | 11.2 (9.8-12.8) | 0.1 (0.1-0.1) | 11.7 (9.8-14) | 0.1 (0-0.1) | -0.09 (-0.18--0.01) |
| Acute hepatitis | Hungary | 7.7 (6.6-8.9) | 0.1 (0.1-0.1) | 2.9 (2.1-4) | 0 (0-0) | -0.04 (-0.07--0.01) |
| Acute hepatitis | Independent State of Papua New Guinea | 95.8 (41.6-165.4) | 2.9 (1.1-5.1) | 57 (20.5-106.3) | 0.6 (0.2-1.2) | -1.69 (-2.17--1.2) |
| Acute hepatitis | Independent State of Samoa | 0.3 (0.3-0.4) | 0.3 (0.2-0.3) | 0.2 (0.2-0.3) | 0.1 (0.1-0.2) | -0.19 (-0.29--0.08) |
| Acute hepatitis | Ireland | 0.7 (0.6-0.8) | 0 (0-0) | 0.8 (0.6-1) | 0 (0-0) | -0.02 (-0.03--0.01) |
| Acute hepatitis | Islamic Republic of Afghanistan | 2671.8 (1846.2-3695.3) | 32.4 (21.3-45.9) | 1607.2 (1081.6-2335.8) | 10.8 (7.1-16.2) | -3 (-4.94--1.03) |
| Acute hepatitis | Islamic Republic of Iran | 787.3 (560.6-1047) | 2.2 (1.5-2.9) | 447.1 (377.4-526.1) | 0.6 (0.5-0.7) | -1.18 (-1.71--0.64) |
| Acute hepatitis | Islamic Republic of Mauritania | 192.8 (79.6-339.4) | 14 (5.8-24.7) | 47.8 (27-78.2) | 1.7 (1-2.9) | -3.17 (-4.32--2.01) |
| Acute hepatitis | Islamic Republic of Pakistan | 7770.4 (5312.1-10510.9) | 8.6 (6-12) | 6906.1 (4761.9-9806) | 4.1 (2.8-5.9) | -1.06 (-2.17-0.07) |
| Acute hepatitis | Jamaica | 1.9 (1.5-2.5) | 0.1 (0.1-0.1) | 1.7 (1.2-2.2) | 0.1 (0-0.1) | -0.05 (-0.09--0.01) |
| Acute hepatitis | Japan | 81.4 (75.5-85.8) | 0.1 (0.1-0.1) | 68.1 (58.5-75.8) | 0 (0-0) | -0.21 (-0.32--0.1) |
| Acute hepatitis | Kingdom of Bahrain | 6.5 (4.6-8.9) | 3.2 (2.3-4.5) | 8.3 (5.1-12.5) | 0.9 (0.5-1.3) | -1.58 (-2.34--0.81) |
| Acute hepatitis | Kingdom of Belgium | 7.2 (6.2-8.2) | 0.1 (0-0.1) | 4.6 (3.9-5.4) | 0 (0-0) | -0.06 (-0.09--0.03) |
| Acute hepatitis | Kingdom of Bhutan | 30.7 (14.4-55.5) | 5.2 (2.7-9.1) | 7.8 (4.1-12.7) | 1.1 (0.6-1.8) | -1.92 (-2.71--1.13) |
| Acute hepatitis | Kingdom of Cambodia | 1061.4 (793.4-1374.3) | 16.6 (12-21.8) | 368.5 (258.7-529.9) | 2.6 (1.8-3.7) | -3.96 (-5.07--2.84) |
| Acute hepatitis | Kingdom of Denmark | 2.6 (2.3-3) | 0 (0-0) | 2 (1.6-2.3) | 0 (0-0) | -0.05 (-0.08--0.02) |
| Acute hepatitis | Kingdom of Eswatini | 17.5 (9.2-28.1) | 4.3 (2.2-6.8) | 9.9 (5.7-15.3) | 1.4 (0.8-2) | -1.35 (-2.23--0.47) |
| Acute hepatitis | Kingdom of Lesotho | 44.8 (24.7-68.7) | 4.6 (2.6-7.1) | 31.3 (19.5-45.5) | 2.5 (1.6-3.6) | -0.63 (-1.66-0.42) |
| Acute hepatitis | Kingdom of Morocco | 373.8 (234.4-578.4) | 2.1 (1.3-3.5) | 285.1 (189.6-429) | 0.8 (0.5-1.2) | -0.94 (-1.48--0.39) |
| Acute hepatitis | Kingdom of Norway | 0.3 (0.2-0.3) | 0 (0-0) | 0.8 (0.7-0.9) | 0 (0-0) | 0 (-0.01-0.01) |
| Acute hepatitis | Kingdom of Saudi Arabia | 694.5 (355.7-1174.3) | 9.1 (4.4-16) | 140.6 (102.2-193.6) | 0.5 (0.4-0.7) | -2.76 (-3.4--2.12) |
| Acute hepatitis | Kingdom of Spain | 7.5 (6.6-8.4) | 0 (0-0) | 6.5 (5.2-7.8) | 0 (0-0) | -0.02 (-0.03--0.01) |
| Acute hepatitis | Kingdom of Sweden | 0.4 (0.3-0.4) | 0 (0-0) | 0.9 (0.8-1.1) | 0 (0-0) | 0 (0-0.01) |
| Acute hepatitis | Kingdom of Thailand | 338.3 (247-455.4) | 0.8 (0.6-1) | 207.3 (157.9-269) | 0.2 (0.2-0.3) | -0.42 (-0.73--0.12) |
| Acute hepatitis | Kingdom of the Netherlands | 2.6 (2.3-2.9) | 0 (0-0) | 2.5 (2.1-3) | 0 (0-0) | -0.02 (-0.03--0.01) |
| Acute hepatitis | Kingdom of Tonga | 9.3 (6.7-12.2) | 12.8 (9.1-17) | 2.7 (1.9-3.8) | 3 (2.1-4.2) | -2.7 (-3.75--1.65) |
| Acute hepatitis | Kyrgyz Republic | 132.9 (106-159.9) | 2.7 (2.2-3.3) | 11.6 (7.2-17.6) | 0.2 (0.1-0.3) | -1.66 (-2.1--1.21) |
| Acute hepatitis | Lao People's Democratic Republic | 183.5 (115.2-265.7) | 5.7 (3.5-8.6) | 46.5 (27.8-75.9) | 0.8 (0.5-1.3) | -2.97 (-3.67--2.26) |
| Acute hepatitis | Lebanese Republic | 21.3 (13.2-30.2) | 0.9 (0.6-1.3) | 14.9 (11.4-18.8) | 0.2 (0.2-0.3) | -0.6 (-0.83--0.37) |
| Acute hepatitis | Malaysia | 171.4 (130.4-221) | 1.5 (1.1-2) | 212.6 (166.9-270.5) | 0.7 (0.6-0.9) | -0.54 (-1.13-0.05) |
| Acute hepatitis | Mongolia | 41.8 (21.5-76.2) | 1.7 (0.9-3) | 5.5 (3.7-8) | 0.2 (0.1-0.2) | -1.28 (-1.69--0.87) |
| Acute hepatitis | Montenegro | 0.1 (0-0.1) | 0 (0-0) | 0 (0-0.1) | 0 (0-0) | -0.01 (-0.01-0) |
| Acute hepatitis | New Zealand | 0.7 (0.6-0.8) | 0 (0-0) | 0.1 (0-0.1) | 0 (0-0) | -0.03 (-0.04--0.02) |
| Acute hepatitis | North Macedonia | 1.7 (1.1-2.8) | 0.1 (0.1-0.2) | 0.9 (0.5-1.6) | 0 (0-0.1) | -0.07 (-0.11--0.02) |
| Acute hepatitis | Northern Mariana Islands | 0.1 (0.1-0.2) | 0.3 (0.2-0.5) | 0.1 (0.1-0.1) | 0.2 (0.1-0.2) | -0.22 (-0.36--0.08) |
| Acute hepatitis | Palestine | 15.4 (8.8-23.4) | 1.4 (0.7-2.2) | 7.4 (5.7-9.4) | 0.3 (0.2-0.3) | -0.96 (-1.27--0.65) |
| Acute hepatitis | People's Democratic Republic of Algeria | 302.5 (207.2-418.5) | 1.9 (1.3-2.7) | 240.2 (154.1-346.3) | 0.7 (0.4-1) | -0.82 (-1.34--0.28) |
| Acute hepatitis | People's Republic of Bangladesh | 7434.9 (6093.6-8711.1) | 9.8 (8.2-11.6) | 3140.3 (2305.9-4206.4) | 2.2 (1.6-2.9) | -2.05 (-3.06--1.02) |
| Acute hepatitis | People's Republic of China | 31234 (26123.3-36925.6) | 3.2 (2.7-3.8) | 3017.8 (2353.6-3957.8) | 0.2 (0.1-0.2) | -2.13 (-2.58--1.67) |
| Acute hepatitis | Plurinational State of Bolivia | 16.6 (10.9-23.7) | 0.3 (0.2-0.4) | 10.7 (7.6-14.7) | 0.1 (0.1-0.1) | -0.19 (-0.31--0.08) |
| Acute hepatitis | Portuguese Republic | 11.1 (10-12.4) | 0.1 (0.1-0.1) | 7.6 (6.3-9.1) | 0 (0-0) | -0.14 (-0.22--0.07) |
| Acute hepatitis | Principality of Andorra | 0.4 (0.2-0.6) | 0.8 (0.5-1.2) | 0.5 (0.3-0.7) | 0.4 (0.2-0.5) | -0.36 (-0.62--0.1) |
| Acute hepatitis | Principality of Monaco | 0 (0-0) | 0 (0-0) | 0 (0-0) | 0 (0-0) | 0 (-0.01-0.01) |
| Acute hepatitis | Puerto Rico | 3.7 (3-4.4) | 0.1 (0.1-0.1) | 1.5 (1.2-1.8) | 0 (0-0) | -0.12 (-0.17--0.07) |
| Acute hepatitis | Republic of Albania | 6.3 (4.2-9.3) | 0.2 (0.1-0.3) | 1.3 (0.7-2.2) | 0 (0-0.1) | -0.15 (-0.21--0.09) |
| Acute hepatitis | Republic of Angola | 1329.4 (649.1-2406.5) | 14.6 (7.6-22.1) | 331.6 (167.2-547.9) | 1.5 (0.8-2.2) | -3.85 (-4.97--2.71) |
| Acute hepatitis | Republic of Armenia | 14.6 (10.4-20.2) | 0.4 (0.3-0.6) | 0.9 (0.6-1.4) | 0 (0-0) | -0.38 (-0.5--0.27) |
| Acute hepatitis | Republic of Austria | 2.1 (1.8-2.3) | 0 (0-0) | 1.8 (1.5-2.2) | 0 (0-0) | -0.02 (-0.04--0.01) |
| Acute hepatitis | Republic of Azerbaijan | 79.6 (60-103.1) | 1 (0.8-1.3) | 14.6 (8.3-24.1) | 0.2 (0.1-0.3) | -0.84 (-1.1--0.58) |
| Acute hepatitis | Republic of Belarus | 32.9 (28.2-37.5) | 0.4 (0.3-0.4) | 13.9 (10-18.2) | 0.1 (0.1-0.2) | -0.24 (-0.4--0.09) |
| Acute hepatitis | Republic of Benin | 122.4 (93.3-161.6) | 3.6 (2.7-4.7) | 70.8 (43.7-107.3) | 0.8 (0.5-1.3) | -1.65 (-2.33--0.97) |
| Acute hepatitis | Republic of Botswana | 17.9 (9.8-27.7) | 2.6 (1.4-3.9) | 9.1 (5.6-13.6) | 0.5 (0.3-0.8) | -1.32 (-1.84--0.8) |
| Acute hepatitis | Republic of Bulgaria | 8.6 (6.5-11.2) | 0.1 (0.1-0.1) | 6 (4.3-8.4) | 0.1 (0-0.1) | -0.04 (-0.11-0.02) |
| Acute hepatitis | Republic of Burundi | 133 (54.4-287.2) | 3.6 (1.4-7.5) | 149.7 (69.3-266.1) | 2 (1-3.6) | -0.65 (-1.33-0.03) |
| Acute hepatitis | Republic of Cabo Verde | 4.6 (3.6-5.9) | 1.8 (1.4-2.3) | 2.4 (1.8-3.3) | 0.5 (0.4-0.6) | -1.25 (-1.88--0.62) |
| Acute hepatitis | Republic of Cameroon | 62.4 (35-133.5) | 0.9 (0.5-1.9) | 56.6 (26.8-105.2) | 0.3 (0.1-0.5) | -0.47 (-0.71--0.23) |
| Acute hepatitis | Republic of Chad | 301 (207.7-408.9) | 6.9 (4.7-9.7) | 437.9 (306.9-625) | 4.3 (2.9-6.3) | -0.82 (-1.97-0.35) |
| Acute hepatitis | Republic of Chile | 8.8 (7.7-10.1) | 0.1 (0.1-0.1) | 2 (1.7-2.4) | 0 (0-0) | -0.09 (-0.11--0.06) |
| Acute hepatitis | Republic of Colombia | 24.6 (20.8-28.7) | 0.1 (0.1-0.1) | 15 (12-19.2) | 0 (0-0) | -0.07 (-0.11--0.03) |
| Acute hepatitis | Republic of Costa Rica | 1.6 (1.3-2.1) | 0.1 (0.1-0.1) | 1.4 (1.2-1.7) | 0 (0-0) | -0.04 (-0.07--0.02) |
| Acute hepatitis | Republic of Côte d'Ivoire | 252.2 (169.7-346.7) | 3.3 (2.3-4.5) | 102.9 (64.8-151.3) | 0.6 (0.4-0.9) | -1.5 (-2.08--0.91) |
| Acute hepatitis | Republic of Croatia | 0.6 (0.5-0.7) | 0 (0-0) | 0.3 (0.2-0.4) | 0 (0-0) | -0.01 (-0.01-0) |
| Acute hepatitis | Republic of Cuba | 9.5 (7.8-12.4) | 0.1 (0.1-0.1) | 2.5 (2-3) | 0 (0-0) | -0.11 (-0.15--0.07) |
| Acute hepatitis | Republic of Cyprus | 0.4 (0.4-0.5) | 0.1 (0-0.1) | 0.3 (0.2-0.5) | 0 (0-0) | -0.06 (-0.08--0.03) |
| Acute hepatitis | Republic of Djibouti | 6.7 (4.2-10.5) | 2.7 (1.8-4.1) | 16.1 (10.6-24.1) | 1.9 (1.3-2.8) | -0.53 (-1.29-0.23) |
| Acute hepatitis | Republic of Ecuador | 10.7 (9-13.1) | 0.1 (0.1-0.1) | 7.3 (5.9-9) | 0 (0-0.1) | -0.1 (-0.16--0.04) |
| Acute hepatitis | Republic of El Salvador | 6.7 (4.8-8.9) | 0.1 (0.1-0.1) | 2.6 (1.8-3.6) | 0 (0-0.1) | -0.08 (-0.12--0.04) |
| Acute hepatitis | Republic of Equatorial Guinea | 1 (0.6-2.2) | 0.3 (0.1-0.5) | 0.5 (0.2-1) | 0.1 (0-0.1) | -0.26 (-0.33--0.18) |
| Acute hepatitis | Republic of Estonia | 3.4 (3-3.8) | 0.2 (0.2-0.3) | 0.3 (0.3-0.4) | 0 (0-0) | -0.27 (-0.35--0.19) |
| Acute hepatitis | Republic of Fiji | 4.1 (2.6-5.7) | 0.7 (0.4-1) | 1.1 (0.8-1.6) | 0.1 (0.1-0.2) | -0.6 (-0.79--0.42) |
| Acute hepatitis | Republic of Finland | 1.3 (1.1-1.6) | 0 (0-0) | 0.8 (0.6-0.9) | 0 (0-0) | -0.02 (-0.03--0.01) |
| Acute hepatitis | Republic of Ghana | 612.7 (402.7-877.3) | 6.1 (3.9-8.9) | 142.9 (101.1-211.8) | 0.6 (0.4-0.9) | -2.87 (-3.53--2.21) |
| Acute hepatitis | Republic of Guatemala | 18.1 (12-29) | 0.2 (0.1-0.3) | 9.6 (7.3-12.7) | 0.1 (0.1-0.1) | -0.13 (-0.22--0.04) |
| Acute hepatitis | Republic of Guinea | 387.4 (238.6-549.6) | 7.9 (4.7-11.3) | 223.6 (149.8-333.1) | 2.6 (1.7-3.9) | -1.66 (-2.74--0.56) |
| Acute hepatitis | Republic of Guinea-Bissau | 56.8 (36.9-81.3) | 8.5 (5.7-12.2) | 29.8 (21-41.6) | 2.5 (1.7-3.4) | -1.85 (-2.92--0.77) |
| Acute hepatitis | Republic of Guyana | 2.8 (2.4-3.2) | 0.4 (0.4-0.5) | 0.8 (0.6-1.2) | 0.1 (0.1-0.2) | -0.22 (-0.33--0.1) |
| Acute hepatitis | Republic of Haiti | 62.3 (33-113.1) | 0.8 (0.5-1.4) | 44.6 (26.8-69.3) | 0.4 (0.2-0.5) | -0.43 (-0.73--0.14) |
| Acute hepatitis | Republic of Honduras | 27.2 (18.1-39.8) | 0.5 (0.4-0.7) | 15.8 (10.8-23.3) | 0.2 (0.1-0.3) | -0.34 (-0.52--0.15) |
| Acute hepatitis | Republic of Iceland | 0 (0-0.1) | 0 (0-0) | 0.1 (0-0.1) | 0 (0-0) | -0.02 (-0.03--0.01) |
| Acute hepatitis | Republic of India | 52498.5 (44755-61547.7) | 6 (5.2-7) | 27993.3 (20210.2-40166.5) | 2.2 (1.6-3.2) | -1.15 (-2.29-0.01) |
| Acute hepatitis | Republic of Indonesia | 7684.5 (4900.8-11278.9) | 5.9 (3.6-8.8) | 5011.8 (3988.5-6485.2) | 1.9 (1.5-2.4) | -1.75 (-2.55--0.94) |
| Acute hepatitis | Republic of Iraq | 272 (203.3-362.3) | 2.3 (1.7-3.1) | 189.7 (129.7-254.5) | 0.7 (0.5-0.9) | -1.09 (-1.63--0.54) |
| Acute hepatitis | Republic of Italy | 15.1 (13.5-16.2) | 0 (0-0) | 38.2 (32.2-43.6) | 0 (0-0) | 0.02 (-0.02-0.07) |
| Acute hepatitis | Republic of Kazakhstan | 189.4 (149.7-236.4) | 1.1 (0.9-1.4) | 17.4 (11.8-24.9) | 0.1 (0.1-0.1) | -1.08 (-1.38--0.77) |
| Acute hepatitis | Republic of Kenya | 479.1 (397.1-590.1) | 3.5 (2.7-4.4) | 624.4 (478.4-821.2) | 2 (1.5-2.8) | -0.62 (-1.4-0.17) |
| Acute hepatitis | Republic of Kiribati | 4.6 (3.4-5.9) | 7.2 (5.5-9.3) | 2.9 (2-3.9) | 2.7 (1.9-3.7) | -1.47 (-2.54--0.38) |
| Acute hepatitis | Republic of Korea | 114.5 (88.5-145.1) | 0.3 (0.3-0.4) | 13.8 (10.4-17.9) | 0 (0-0) | -0.39 (-0.49--0.28) |
| Acute hepatitis | Republic of Latvia | 19.8 (17.5-22) | 0.8 (0.7-0.9) | 2 (1.6-2.4) | 0.1 (0.1-0.1) | -0.75 (-1.01--0.49) |
| Acute hepatitis | Republic of Liberia | 64.8 (47.8-85.2) | 3.4 (2.5-4.7) | 48 (30.1-76.6) | 1.4 (0.9-2.1) | -1.43 (-2.24--0.61) |
| Acute hepatitis | Republic of Lithuania | 7.8 (6.7-9) | 0.2 (0.2-0.3) | 0.9 (0.7-1.1) | 0 (0-0) | -0.24 (-0.33--0.15) |
| Acute hepatitis | Republic of Madagascar | 253.2 (212.8-298.9) | 3.1 (2.6-3.7) | 203.8 (121.7-303.6) | 1.1 (0.7-1.7) | -1.15 (-1.81--0.48) |
| Acute hepatitis | Republic of Malawi | 424.4 (324-528.3) | 6 (4.6-7.5) | 376 (276.7-504.5) | 3.3 (2.5-4.3) | -1.4 (-2.58--0.2) |
| Acute hepatitis | Republic of Maldives | 1.9 (1.2-2.8) | 1.2 (0.8-1.7) | 0.5 (0.4-0.7) | 0.1 (0.1-0.2) | -1.03 (-1.28--0.78) |
| Acute hepatitis | Republic of Mali | 45.6 (23.9-102.8) | 0.7 (0.4-1.6) | 67.4 (31.7-125.2) | 0.5 (0.2-0.9) | -0.14 (-0.39-0.12) |
| Acute hepatitis | Republic of Malta | 0.1 (0.1-0.1) | 0 (0-0) | 0.1 (0.1-0.1) | 0 (0-0) | -0.03 (-0.05--0.01) |
| Acute hepatitis | Republic of Mauritius | 2.5 (2.1-3) | 0.3 (0.2-0.4) | 0.3 (0.2-0.3) | 0 (0-0) | -0.36 (-0.44--0.27) |
| Acute hepatitis | Republic of Moldova | 29 (23.8-35) | 0.7 (0.6-0.8) | 2.6 (2-3.2) | 0.1 (0.1-0.1) | -0.55 (-0.77--0.33) |
| Acute hepatitis | Republic of Mozambique | 764.2 (502.4-1129.6) | 8 (5.2-11.2) | 375.1 (245.1-546.5) | 2.1 (1.4-3.2) | -2.32 (-3.41--1.21) |
| Acute hepatitis | Republic of Namibia | 15.8 (9.3-22.8) | 2 (1.2-2.9) | 11.8 (7-18.2) | 0.7 (0.5-1.1) | -0.94 (-1.51--0.37) |
| Acute hepatitis | Republic of Nauru | 0.1 (0-0.1) | 0.8 (0.5-1) | 0 (0-0) | 0.2 (0.1-0.3) | -0.63 (-0.88--0.37) |
| Acute hepatitis | Republic of Nicaragua | 13.7 (9.3-19.9) | 0.3 (0.2-0.4) | 3 (2.2-4) | 0.1 (0-0.1) | -0.24 (-0.33--0.15) |
| Acute hepatitis | Republic of Niue | 0 (0-0) | 0.3 (0.2-0.4) | 0 (0-0) | 0.1 (0.1-0.2) | -0.27 (-0.37--0.17) |
| Acute hepatitis | Republic of Palau | 0.2 (0.2-0.3) | 1.8 (1.2-2.5) | 0.1 (0.1-0.2) | 0.7 (0.5-0.9) | -0.78 (-1.23--0.33) |
| Acute hepatitis | Republic of Panama | 1.8 (1.5-2.3) | 0.1 (0.1-0.1) | 1.5 (1.1-1.9) | 0 (0-0) | -0.06 (-0.1--0.02) |
| Acute hepatitis | Republic of Paraguay | 5.3 (3.1-8.3) | 0.2 (0.1-0.2) | 4.2 (2.9-6) | 0.1 (0-0.1) | -0.1 (-0.18--0.03) |
| Acute hepatitis | Republic of Peru | 34.9 (26.1-45.6) | 0.2 (0.1-0.2) | 15 (10.8-19.8) | 0 (0-0.1) | -0.17 (-0.24--0.11) |
| Acute hepatitis | Republic of Poland | 3.9 (3.5-4.4) | 0 (0-0) | 5.6 (4.7-6.4) | 0 (0-0) | -0.01 (-0.03-0.01) |
| Acute hepatitis | Republic of Rwanda | 321.2 (243.7-418.2) | 6.9 (5.1-9.2) | 166.5 (117.5-233.6) | 1.9 (1.4-2.6) | -2.55 (-3.5--1.6) |
| Acute hepatitis | Republic of San Marino | 0.2 (0.2-0.3) | 0.8 (0.7-1) | 0.2 (0.1-0.2) | 0.3 (0.2-0.4) | -0.54 (-0.8--0.28) |
| Acute hepatitis | Republic of Senegal | 62.9 (38.7-93.1) | 1.2 (0.7-1.8) | 44.6 (24.5-71) | 0.4 (0.2-0.7) | -0.66 (-1--0.31) |
| Acute hepatitis | Republic of Serbia | 8.8 (5.9-12.7) | 0.1 (0.1-0.1) | 4.4 (2.8-6) | 0 (0-0) | -0.1 (-0.15--0.04) |
| Acute hepatitis | Republic of Seychelles | 1.3 (0.9-1.9) | 2.2 (1.5-3.3) | 0.7 (0.6-1) | 0.6 (0.5-0.8) | -1.17 (-1.61--0.73) |
| Acute hepatitis | Republic of Sierra Leone | 218.6 (132.7-323.4) | 6.4 (3.8-9.3) | 74.2 (48.2-106.9) | 1.3 (0.8-1.8) | -2.14 (-3.05--1.21) |
| Acute hepatitis | Republic of Singapore | 0.6 (0.5-0.6) | 0 (0-0) | 0.5 (0.4-0.6) | 0 (0-0) | -0.07 (-0.11--0.04) |
| Acute hepatitis | Republic of Slovenia | 1.1 (1-1.3) | 0.1 (0-0.1) | 0.5 (0.4-0.6) | 0 (0-0) | -0.04 (-0.06--0.02) |
| Acute hepatitis | Republic of South Africa | 212.2 (155.6-276.5) | 0.8 (0.6-1) | 187.2 (146.3-236.5) | 0.4 (0.3-0.5) | -0.35 (-0.66--0.04) |
| Acute hepatitis | Republic of South Sudan | 168.8 (122-249.5) | 4.4 (3-6.5) | 252.6 (160.5-372.5) | 4.4 (2.8-6.6) | 0.18 (-0.79-1.17) |
| Acute hepatitis | Republic of Sudan | 2635 (1484.4-3946.1) | 19 (9.5-30.5) | 935.9 (397.6-1643.8) | 3.8 (1.5-6.7) | -3.04 (-4.51--1.54) |
| Acute hepatitis | Republic of Suriname | 0.6 (0.4-0.8) | 0.2 (0.1-0.2) | 0.4 (0.3-0.5) | 0.1 (0.1-0.1) | -0.13 (-0.2--0.06) |
| Acute hepatitis | Republic of Tajikistan | 236.6 (183.4-304.8) | 3.4 (2.7-4.2) | 95.7 (50.2-185.7) | 0.9 (0.5-1.7) | -1.38 (-2.09--0.66) |
| Acute hepatitis | Republic of the Congo | 123.2 (43.1-234.8) | 6.2 (2.4-11.5) | 36.3 (15.2-66.9) | 0.9 (0.4-1.6) | -2.35 (-3.24--1.46) |
| Acute hepatitis | Republic of the Gambia | 4.4 (2.3-7.8) | 0.7 (0.4-1.3) | 3.3 (1.4-7) | 0.2 (0.1-0.5) | -0.43 (-0.61--0.26) |
| Acute hepatitis | Republic of the Marshall Islands | 0.2 (0.1-0.3) | 0.6 (0.3-0.9) | 0.1 (0.1-0.1) | 0.2 (0.1-0.3) | -0.5 (-0.71--0.3) |
| Acute hepatitis | Republic of the Niger | 400.7 (289.8-553.4) | 6.7 (4.8-9) | 271.1 (160.6-432.7) | 2 (1.2-3.1) | -1.64 (-2.55--0.71) |
| Acute hepatitis | Republic of the Philippines | 1145.4 (951.7-1364.6) | 2.2 (1.8-2.6) | 162.8 (133.1-192.8) | 0.2 (0.1-0.2) | -1.72 (-2.09--1.34) |
| Acute hepatitis | Republic of the Union of Myanmar | 474.9 (337.1-706.7) | 1.5 (1.1-2.2) | 248.7 (168.8-353.8) | 0.5 (0.3-0.7) | -1.04 (-1.44--0.63) |
| Acute hepatitis | Republic of Trinidad and Tobago | 1.7 (1.3-2.1) | 0.2 (0.1-0.2) | 1.2 (0.9-1.7) | 0.1 (0.1-0.1) | -0.12 (-0.19--0.05) |
| Acute hepatitis | Republic of Tunisia | 110.6 (70.3-167.1) | 1.8 (1.2-2.8) | 72.7 (45.5-109.9) | 0.6 (0.4-0.8) | -0.8 (-1.27--0.33) |
| Acute hepatitis | Republic of Turkey | 743.2 (519.9-1017.7) | 1.4 (1-1.9) | 195 (149.6-251.9) | 0.2 (0.2-0.3) | -1.09 (-1.4--0.77) |
| Acute hepatitis | Republic of Uganda | 777.9 (438.2-1287.9) | 7.4 (4.4-12.3) | 596.2 (394.5-857.7) | 2.5 (1.8-3.4) | -2.18 (-3.25--1.1) |
| Acute hepatitis | Republic of Uzbekistan | 1112.4 (950.1-1305.9) | 4.3 (3.8-5) | 88.8 (68.2-113.4) | 0.3 (0.2-0.3) | -2.21 (-2.82--1.6) |
| Acute hepatitis | Republic of Vanuatu | 1.6 (0.7-2.6) | 1.4 (0.6-2.4) | 0.9 (0.4-1.5) | 0.3 (0.1-0.6) | -1.01 (-1.34--0.67) |
| Acute hepatitis | Republic of Yemen | 1774.6 (874.7-2925.2) | 20.5 (8.9-36.1) | 632 (304.6-1079.1) | 3.5 (1.6-6) | -3.72 (-5.19--2.23) |
| Acute hepatitis | Republic of Zambia | 407 (283.9-551.9) | 7.9 (5.4-10.5) | 241 (161.8-329.9) | 2.1 (1.5-2.9) | -2.33 (-3.46--1.2) |
| Acute hepatitis | Republic of Zimbabwe | 262.1 (74.6-476.7) | 5.3 (1.6-9.7) | 285.8 (129.5-500.1) | 3.2 (1.5-5.5) | -0.29 (-1.02-0.44) |
| Acute hepatitis | Romania | 16.7 (11.7-24.3) | 0.1 (0.1-0.1) | 6.1 (4.7-8) | 0 (0-0) | -0.08 (-0.11--0.05) |
| Acute hepatitis | Russian Federation | 140.7 (125.7-152) | 0.1 (0.1-0.1) | 29.2 (25.4-32.3) | 0 (0-0) | -0.16 (-0.23--0.09) |
| Acute hepatitis | Saint Kitts and Nevis | 0.1 (0.1-0.1) | 0.2 (0.2-0.3) | 0 (0-0) | 0.1 (0-0.1) | -0.23 (-0.31--0.15) |
| Acute hepatitis | Saint Lucia | 0.2 (0.1-0.3) | 0.2 (0.1-0.2) | 0.1 (0.1-0.1) | 0 (0-0) | -0.18 (-0.24--0.12) |
| Acute hepatitis | Saint Vincent and the Grenadines | 0 (0-0) | 0 (0-0) | 0 (0-0) | 0 (0-0) | -0.02 (-0.06-0.02) |
| Acute hepatitis | Slovak Republic | 1.8 (1.2-2.5) | 0 (0-0) | 1.5 (0.8-2.5) | 0 (0-0) | -0.02 (-0.04-0.01) |
| Acute hepatitis | Socialist Republic of Viet Nam | 1620.5 (1084.7-2370.9) | 3.6 (2.3-5.3) | 347.9 (258.9-467.3) | 0.3 (0.3-0.5) | -2.11 (-2.61--1.62) |
| Acute hepatitis | Solomon Islands | 9 (3.2-16.8) | 3.7 (1.2-6.9) | 3.5 (1.5-6.2) | 0.6 (0.3-1.1) | -1.81 (-2.37--1.25) |
| Acute hepatitis | State of Eritrea | 111.3 (83.3-151.3) | 5.5 (4.1-7.7) | 117.2 (79-173.2) | 2.8 (1.9-4) | -1.01 (-1.93--0.08) |
| Acute hepatitis | State of Israel | 4.1 (3.5-4.9) | 0.1 (0.1-0.1) | 2.5 (2.1-2.9) | 0 (0-0) | -0.09 (-0.14--0.05) |
| Acute hepatitis | State of Kuwait | 1 (0.8-1.1) | 0.1 (0.1-0.1) | 0.9 (0.7-1.1) | 0 (0-0) | -0.14 (-0.18--0.09) |
| Acute hepatitis | State of Libya | 54.2 (24.3-98.4) | 2.3 (1-4.3) | 47.3 (32.6-71.2) | 0.8 (0.6-1.2) | -0.91 (-1.45--0.36) |
| Acute hepatitis | State of Qatar | 2.8 (2.1-3.6) | 2 (1.5-2.5) | 4.8 (3.2-7) | 0.4 (0.3-0.6) | -1.3 (-1.75--0.86) |
| Acute hepatitis | Sultanate of Oman | 69.6 (41.5-99.8) | 7.8 (4.4-11.3) | 26.7 (19.5-36.5) | 1.2 (0.9-1.6) | -1.65 (-2.66--0.63) |
| Acute hepatitis | Swiss Confederation | 7.5 (6.7-8.3) | 0.1 (0.1-0.1) | 5.7 (4.8-6.8) | 0 (0-0) | -0.11 (-0.16--0.06) |
| Acute hepatitis | Syrian Arab Republic | 129 (89.7-180.5) | 1.7 (1.1-2.6) | 32.9 (21.4-49.3) | 0.3 (0.2-0.4) | -1.14 (-1.5--0.79) |
| Acute hepatitis | Taiwan (Province of China) | 6.7 (5.8-7.5) | 0 (0-0) | 1.8 (1.4-2.3) | 0 (0-0) | -0.06 (-0.08--0.04) |
| Acute hepatitis | Togolese Republic | 14.2 (7.8-25.9) | 0.6 (0.3-1.1) | 18.7 (8.7-34) | 0.3 (0.2-0.6) | -0.21 (-0.44-0.03) |
| Acute hepatitis | Tokelau | 0 (0-0) | 0.6 (0.3-0.8) | 0 (0-0) | 0.3 (0.2-0.4) | -0.44 (-0.62--0.25) |
| Acute hepatitis | Turkmenistan | 212.5 (178.6-253.4) | 4.7 (4.1-5.6) | 40.8 (26.9-59.9) | 0.8 (0.5-1.2) | -1.81 (-2.44--1.18) |
| Acute hepatitis | Tuvalu | 0.1 (0.1-0.1) | 0.9 (0.6-1.2) | 0 (0-0) | 0.2 (0.1-0.3) | -0.71 (-0.93--0.48) |
| Acute hepatitis | Ukraine | 143.1 (126-158.6) | 0.3 (0.3-0.3) | 31.4 (21-42.5) | 0.1 (0-0.1) | -0.22 (-0.37--0.08) |
| Acute hepatitis | Union of the Comoros | 8.4 (5.4-11.9) | 2.8 (1.9-3.9) | 10 (6.5-14.8) | 1.7 (1.1-2.5) | -0.71 (-1.4--0.01) |
| Acute hepatitis | United Arab Emirates | 11.3 (7.7-16.2) | 1.8 (1.3-2.5) | 37.1 (25.6-53.3) | 0.8 (0.6-1.1) | -0.43 (-0.95-0.1) |
| Acute hepatitis | United Kingdom of Great Britain and Northern Ireland | 3.1 (2.8-3.2) | 0 (0-0) | 10 (8.9-10.7) | 0 (0-0) | 0.01 (0-0.02) |
| Acute hepatitis | United Mexican States | 259 (234.3-289.1) | 0.3 (0.3-0.3) | 149.7 (127.9-174.6) | 0.1 (0.1-0.2) | -0.15 (-0.28--0.03) |
| Acute hepatitis | United Republic of Tanzania | 869.9 (664-1101.3) | 4.7 (3.6-5.9) | 538.4 (360.2-742.7) | 1.4 (1-1.9) | -1.7 (-2.49--0.91) |
| Acute hepatitis | United States of America | 174.9 (160.1-185.6) | 0.1 (0.1-0.1) | 133.8 (116.6-146.4) | 0 (0-0) | -0.08 (-0.11--0.05) |
| Acute hepatitis | United States Virgin Islands | 0 (0-0.1) | 0 (0-0.1) | 0 (0-0) | 0 (0-0) | -0.03 (-0.05--0.01) |
| Acute hepatitis A | American Samoa | 0 (0-0) | 0.1 (0-0.1) | 0 (0-0) | 0 (0-0) | -0.15 (-0.25--0.04) |
| Acute hepatitis A | Antigua and Barbuda | 0 (0-0) | 0 (0-0) | 0 (0-0) | 0 (0-0) | -0.07 (-0.09--0.04) |
| Acute hepatitis A | Arab Republic of Egypt | 2409.4 (869.3-4347.1) | 6.7 (2.3-13) | 278 (67.9-783.8) | 0.4 (0.1-1.1) | -2.56 (-4.13--0.97) |
| Acute hepatitis A | Argentine Republic | 22.1 (15.1-45.1) | 0.1 (0-0.1) | 4 (3.2-5) | 0 (0-0) | -0.13 (-0.17--0.09) |
| Acute hepatitis A | Australia | 0.8 (0.6-0.9) | 0 (0-0) | 0.6 (0.4-0.8) | 0 (0-0) | -0.02 (-0.03--0.01) |
| Acute hepatitis A | Barbados | 0.3 (0.2-0.4) | 0.1 (0.1-0.1) | 0.1 (0.1-0.1) | 0 (0-0) | -0.11 (-0.19--0.02) |
| Acute hepatitis A | Belize | 0.1 (0.1-0.1) | 0.1 (0-0.1) | 0 (0-0) | 0 (0-0) | -0.11 (-0.15--0.07) |
| Acute hepatitis A | Bermuda | 0 (0-0) | 0 (0-0) | 0 (0-0) | 0 (0-0) | -0.02 (-0.03--0.01) |
| Acute hepatitis A | Bolivarian Republic of Venezuela | 12.8 (9.6-20.5) | 0.1 (0.1-0.1) | 11.5 (7.9-16.7) | 0 (0-0.1) | -0.02 (-0.07-0.03) |
| Acute hepatitis A | Bosnia and Herzegovina | 0.9 (0.4-2) | 0 (0-0) | 0.2 (0.1-0.4) | 0 (0-0) | -0.15 (-0.26--0.04) |
| Acute hepatitis A | Brunei Darussalam | 0.4 (0.2-0.7) | 0.2 (0.1-0.3) | 0.1 (0-0.1) | 0 (0-0) | -1.79 (-2.35--1.23) |
| Acute hepatitis A | Burkina Faso | 92.8 (48-173) | 1.4 (0.7-2.5) | 53.5 (21.6-110) | 0.4 (0.1-0.7) | -1.31 (-2.29--0.33) |
| Acute hepatitis A | Canada | 2.7 (2.2-3.4) | 0 (0-0) | 3.9 (3.1-4.9) | 0 (0-0) | -0.05 (-0.07--0.03) |
| Acute hepatitis A | Central African Republic | 74.2 (23.4-167.3) | 2 (0.6-5.1) | 30.8 (7.1-68.8) | 0.6 (0.1-1.3) | -1.7 (-2.69--0.69) |
| Acute hepatitis A | Commonwealth of Dominica | 0 (0-0.1) | 0.1 (0-0.1) | 0 (0-0) | 0 (0-0) | -0.07 (-0.11--0.03) |
| Acute hepatitis A | Commonwealth of the Bahamas | 0.1 (0.1-0.1) | 0 (0-0.1) | 0 (0-0) | 0 (0-0) | -0.07 (-0.11--0.04) |
| Acute hepatitis A | Cook Islands | 0 (0-0) | 0 (0-0) | 0 (0-0) | 0 (0-0) | -0.04 (-0.05--0.02) |
| Acute hepatitis A | Czech Republic | 9.5 (8.1-11.1) | 0.1 (0.1-0.1) | 2.2 (1.6-3) | 0 (0-0) | -0.06 (-0.1--0.03) |
| Acute hepatitis A | Democratic People's Republic of Korea | 50.8 (16.1-130.7) | 0.3 (0.1-0.7) | 7.1 (2.3-17.6) | 0 (0-0.1) | -1.11 (-1.46--0.76) |
| Acute hepatitis A | Democratic Republic of Sao Tome and Principe | 0.6 (0.3-1) | 0.7 (0.4-1.1) | 0.1 (0.1-0.2) | 0.1 (0-0.1) | -1.31 (-1.78--0.84) |
| Acute hepatitis A | Democratic Republic of the Congo | 151.5 (78.8-254.9) | 0.3 (0.2-0.5) | 52.7 (11.8-116.2) | 0.1 (0-0.2) | -0.35 (-0.6--0.1) |
| Acute hepatitis A | Democratic Republic of Timor-Leste | 2.7 (0.7-6.9) | 0.4 (0.1-0.9) | 0.9 (0.2-2) | 0.1 (0-0.2) | -0.61 (-0.81--0.42) |
| Acute hepatitis A | Democratic Socialist Republic of Sri Lanka | 31.4 (16.4-53.5) | 0.2 (0.1-0.4) | 4 (2-6.9) | 0 (0-0) | -0.61 (-0.77--0.45) |
| Acute hepatitis A | Dominican Republic | 3.5 (2.3-5.5) | 0 (0-0.1) | 1.3 (0.9-1.9) | 0 (0-0) | -0.08 (-0.14--0.02) |
| Acute hepatitis A | Eastern Republic of Uruguay | 1 (0.8-1.3) | 0 (0-0) | 0.2 (0.2-0.3) | 0 (0-0) | -0.05 (-0.06--0.03) |
| Acute hepatitis A | Federal Democratic Republic of Ethiopia | 1437 (679.6-2492.2) | 4 (1.7-7.9) | 831.6 (286.3-2182.4) | 1.2 (0.4-3.3) | -1.93 (-2.95--0.89) |
| Acute hepatitis A | Federal Democratic Republic of Nepal | 1400.3 (751-2251.5) | 6.3 (3.4-9.7) | 290.5 (163.3-502.3) | 1 (0.6-1.8) | -1.48 (-2.49--0.46) |
| Acute hepatitis A | Federal Republic of Germany | 13.7 (9.8-17.4) | 0 (0-0) | 10.8 (8.4-13.5) | 0 (0-0) | -0.07 (-0.09--0.04) |
| Acute hepatitis A | Federal Republic of Nigeria | 3096.5 (1247.2-5838.5) | 4.4 (1.9-8.1) | 476.8 (230.6-871.6) | 0.3 (0.1-0.5) | -4.49 (-5.68--3.29) |
| Acute hepatitis A | Federal Republic of Somalia | 292.1 (116.4-677.6) | 4.7 (1.6-13.5) | 301.8 (71.8-995.9) | 2.4 (0.5-9.5) | -0.59 (-2.19-1.04) |
| Acute hepatitis A | Federated States of Micronesia | 0.1 (0-0.3) | 0.1 (0-0.3) | 0 (0-0) | 0 (0-0) | -0.65 (-0.85--0.45) |
| Acute hepatitis A | Federative Republic of Brazil | 173.7 (153.2-218.7) | 0.1 (0.1-0.2) | 38.2 (31.6-46.9) | 0 (0-0) | -0.18 (-0.26--0.1) |
| Acute hepatitis A | French Republic | 7.1 (6.1-8.3) | 0 (0-0) | 8.8 (6.8-11) | 0 (0-0) | -0.04 (-0.06--0.02) |
| Acute hepatitis A | Gabonese Republic | 3.2 (0.9-6.1) | 0.3 (0.1-0.6) | 0.4 (0.2-0.9) | 0 (0-0.1) | -1.26 (-1.67--0.85) |
| Acute hepatitis A | Georgia | 2.1 (1-3.6) | 0 (0-0.1) | 0.2 (0.1-0.3) | 0 (0-0) | -0.41 (-0.52--0.29) |
| Acute hepatitis A | Grand Duchy of Luxembourg | 0 (0-0) | 0 (0-0) | 0 (0-0) | 0 (0-0) | -0.02 (-0.03--0.01) |
| Acute hepatitis A | Greenland | 0 (0-0) | 0 (0-0.1) | 0 (0-0) | 0 (0-0) | -0.15 (-0.19--0.11) |
| Acute hepatitis A | Grenada | 0.1 (0-0.1) | 0.1 (0-0.1) | 0 (0-0) | 0 (0-0) | -0.16 (-0.24--0.09) |
| Acute hepatitis A | Guam | 0 (0-0) | 0 (0-0) | 0 (0-0) | 0 (0-0) | -0.02 (-0.08-0.04) |
| Acute hepatitis A | Hashemite Kingdom of Jordan | 7.6 (4.3-12.8) | 0.4 (0.2-0.7) | 3.9 (2.2-6.5) | 0 (0-0.1) | -1.09 (-1.4--0.78) |
| Acute hepatitis A | Hellenic Republic | 0.7 (0.6-1) | 0 (0-0) | 1.1 (0.8-1.6) | 0 (0-0) | -0.09 (-0.18--0.01) |
| Acute hepatitis A | Hungary | 3.8 (3.2-4.4) | 0 (0-0) | 0.6 (0.4-0.8) | 0 (0-0) | -0.04 (-0.07--0.01) |
| Acute hepatitis A | Independent State of Papua New Guinea | 36.3 (13.4-85.6) | 0.9 (0.3-2.5) | 26 (8.1-54.5) | 0.3 (0.1-0.6) | -1.69 (-2.17--1.2) |
| Acute hepatitis A | Independent State of Samoa | 0.1 (0-0.2) | 0.1 (0-0.1) | 0 (0-0.1) | 0 (0-0) | -0.19 (-0.29--0.08) |
| Acute hepatitis A | Ireland | 0.4 (0.3-0.5) | 0 (0-0) | 0.6 (0.5-0.8) | 0 (0-0) | -0.02 (-0.03--0.01) |
| Acute hepatitis A | Islamic Republic of Afghanistan | 1138.4 (477.4-1939.3) | 12.2 (4.3-22.7) | 641.7 (279-1149.2) | 3.3 (1.2-6.4) | -3 (-4.94--1.03) |
| Acute hepatitis A | Islamic Republic of Iran | 246.2 (155-352.1) | 0.5 (0.3-0.9) | 30.5 (19-47.5) | 0 (0-0.1) | -1.18 (-1.71--0.64) |
| Acute hepatitis A | Islamic Republic of Mauritania | 50.2 (19.3-119.2) | 3.9 (1.4-9.2) | 8.5 (3.9-15.3) | 0.3 (0.1-0.6) | -3.17 (-4.32--2.01) |
| Acute hepatitis A | Islamic Republic of Pakistan | 5878.1 (3502.5-8644.9) | 6.1 (3.4-9.1) | 3032.7 (1339.8-6332.9) | 1.7 (0.7-3.8) | -1.06 (-2.17-0.07) |
| Acute hepatitis A | Jamaica | 1.3 (1-1.7) | 0.1 (0-0.1) | 0.5 (0.3-0.7) | 0 (0-0) | -0.05 (-0.09--0.01) |
| Acute hepatitis A | Japan | 13.8 (12.3-15.8) | 0 (0-0) | 4.8 (3.8-6.2) | 0 (0-0) | -0.21 (-0.32--0.1) |
| Acute hepatitis A | Kingdom of Bahrain | 1.1 (0.6-1.8) | 0.5 (0.3-0.9) | 0.5 (0.3-1.1) | 0.1 (0-0.1) | -1.58 (-2.34--0.81) |
| Acute hepatitis A | Kingdom of Belgium | 1.5 (1.1-2) | 0 (0-0) | 1.4 (1.1-1.9) | 0 (0-0) | -0.06 (-0.09--0.03) |
| Acute hepatitis A | Kingdom of Bhutan | 25.6 (10.4-49.9) | 4 (1.8-7.6) | 2.7 (1.2-7.1) | 0.4 (0.2-1) | -1.92 (-2.71--1.13) |
| Acute hepatitis A | Kingdom of Cambodia | 633.2 (402.3-928.9) | 9.4 (5.7-14.2) | 143.6 (77.1-247) | 1 (0.6-1.8) | -3.96 (-5.07--2.84) |
| Acute hepatitis A | Kingdom of Denmark | 0.7 (0.6-0.9) | 0 (0-0) | 0.7 (0.6-0.9) | 0 (0-0) | -0.05 (-0.08--0.02) |
| Acute hepatitis A | Kingdom of Eswatini | 6.1 (3.3-11.2) | 1.1 (0.5-2) | 2.1 (1-3.6) | 0.3 (0.1-0.5) | -1.35 (-2.23--0.47) |
| Acute hepatitis A | Kingdom of Lesotho | 12.7 (5.3-26.6) | 1.1 (0.4-2.5) | 7 (3.1-14.2) | 0.5 (0.2-1.1) | -0.63 (-1.66-0.42) |
| Acute hepatitis A | Kingdom of Morocco | 176.4 (93.8-308.5) | 0.9 (0.4-1.7) | 46.9 (21.7-93.8) | 0.1 (0.1-0.3) | -0.94 (-1.48--0.39) |
| Acute hepatitis A | Kingdom of Norway | 0.1 (0.1-0.1) | 0 (0-0) | 0.2 (0.1-0.2) | 0 (0-0) | 0 (-0.01-0.01) |
| Acute hepatitis A | Kingdom of Saudi Arabia | 240.9 (95.9-491.5) | 2.9 (1-6.5) | 21.4 (12.3-34.4) | 0.1 (0-0.1) | -2.76 (-3.4--2.12) |
| Acute hepatitis A | Kingdom of Spain | 0.9 (0.7-1.2) | 0 (0-0) | 1.1 (0.8-1.6) | 0 (0-0) | -0.02 (-0.03--0.01) |
| Acute hepatitis A | Kingdom of Sweden | 0 (0-0.1) | 0 (0-0) | 0.1 (0.1-0.2) | 0 (0-0) | 0 (0-0.01) |
| Acute hepatitis A | Kingdom of Thailand | 26.6 (12.3-47) | 0.1 (0-0.1) | 25.6 (18.1-36.4) | 0 (0-0) | -0.42 (-0.73--0.12) |
| Acute hepatitis A | Kingdom of the Netherlands | 1.1 (0.9-1.3) | 0 (0-0) | 0.9 (0.7-1.2) | 0 (0-0) | -0.02 (-0.03--0.01) |
| Acute hepatitis A | Kingdom of Tonga | 2.7 (1.5-4.5) | 3.5 (1.9-6) | 0.8 (0.4-1.2) | 0.8 (0.5-1.2) | -2.7 (-3.75--1.65) |
| Acute hepatitis A | Kyrgyz Republic | 74.2 (53.7-95.2) | 1.5 (1.1-1.9) | 4.2 (2.5-6.8) | 0.1 (0-0.1) | -1.66 (-2.1--1.21) |
| Acute hepatitis A | Lao People's Democratic Republic | 88.4 (37.9-145.4) | 2.4 (1-4.4) | 14.5 (6.1-25.8) | 0.2 (0.1-0.4) | -2.97 (-3.67--2.26) |
| Acute hepatitis A | Lebanese Republic | 12.5 (6.6-18.3) | 0.5 (0.3-0.8) | 5.3 (3.9-7.1) | 0.1 (0.1-0.1) | -0.6 (-0.83--0.37) |
| Acute hepatitis A | Malaysia | 9.2 (5.9-14.4) | 0.1 (0-0.1) | 4.7 (3.1-6.9) | 0 (0-0) | -0.54 (-1.13-0.05) |
| Acute hepatitis A | Mongolia | 14.3 (5.7-28.2) | 0.5 (0.2-1) | 1.1 (0.7-1.7) | 0 (0-0.1) | -1.28 (-1.69--0.87) |
| Acute hepatitis A | Montenegro | 0 (0-0) | 0 (0-0) | 0 (0-0) | 0 (0-0) | -0.01 (-0.01-0) |
| Acute hepatitis A | New Zealand | 0.2 (0.2-0.3) | 0 (0-0) | 0 (0-0) | 0 (0-0) | -0.03 (-0.04--0.02) |
| Acute hepatitis A | North Macedonia | 0.7 (0.4-1.1) | 0 (0-0.1) | 0 (0-0.1) | 0 (0-0) | -0.07 (-0.11--0.02) |
| Acute hepatitis A | Northern Mariana Islands | 0 (0-0) | 0.1 (0-0.1) | 0 (0-0) | 0 (0-0) | -0.22 (-0.36--0.08) |
| Acute hepatitis A | Palestine | 5 (2.3-9.4) | 0.4 (0.2-0.8) | 1.6 (1.1-2.1) | 0.1 (0-0.1) | -0.96 (-1.27--0.65) |
| Acute hepatitis A | People's Democratic Republic of Algeria | 84.3 (45.4-160.1) | 0.4 (0.2-0.9) | 21.5 (9.5-48.6) | 0.1 (0-0.1) | -0.82 (-1.34--0.28) |
| Acute hepatitis A | People's Republic of Bangladesh | 6115.7 (4680.8-7837.4) | 7.7 (5.8-10.1) | 1683.1 (963.4-2762.5) | 1.2 (0.7-1.9) | -2.05 (-3.06--1.02) |
| Acute hepatitis A | People's Republic of China | 12334.2 (8462.8-17323.3) | 1.2 (0.8-1.7) | 376.4 (247.5-542.9) | 0 (0-0) | -2.13 (-2.58--1.67) |
| Acute hepatitis A | Plurinational State of Bolivia | 13.7 (9-19.5) | 0.2 (0.1-0.3) | 6.7 (4.8-9.2) | 0.1 (0-0.1) | -0.19 (-0.31--0.08) |
| Acute hepatitis A | Portuguese Republic | 0.7 (0.6-0.8) | 0 (0-0) | 0.7 (0.5-0.9) | 0 (0-0) | -0.14 (-0.22--0.07) |
| Acute hepatitis A | Principality of Andorra | 0.1 (0.1-0.2) | 0.3 (0.1-0.5) | 0.1 (0.1-0.2) | 0.1 (0-0.2) | -0.36 (-0.62--0.1) |
| Acute hepatitis A | Principality of Monaco | 0 (0-0) | 0 (0-0) | 0 (0-0) | 0 (0-0) | 0 (-0.01-0.01) |
| Acute hepatitis A | Puerto Rico | 2.1 (1.6-2.7) | 0.1 (0-0.1) | 0.5 (0.4-0.6) | 0 (0-0) | -0.12 (-0.17--0.07) |
| Acute hepatitis A | Republic of Albania | 3.4 (2.2-5.2) | 0.1 (0.1-0.1) | 0.2 (0.1-0.3) | 0 (0-0) | -0.15 (-0.21--0.09) |
| Acute hepatitis A | Republic of Angola | 505 (123.1-1229.4) | 3.3 (0.9-8.2) | 49.9 (8.1-131.5) | 0.1 (0-0.4) | -3.85 (-4.97--2.71) |
| Acute hepatitis A | Republic of Armenia | 3.1 (2.1-4.7) | 0.1 (0.1-0.1) | 0.1 (0-0.1) | 0 (0-0) | -0.38 (-0.5--0.27) |
| Acute hepatitis A | Republic of Austria | 0.6 (0.5-0.7) | 0 (0-0) | 0.7 (0.6-0.9) | 0 (0-0) | -0.02 (-0.04--0.01) |
| Acute hepatitis A | Republic of Azerbaijan | 22.1 (13.2-32.5) | 0.3 (0.2-0.4) | 1.5 (0.8-3.1) | 0 (0-0) | -0.84 (-1.1--0.58) |
| Acute hepatitis A | Republic of Belarus | 9.5 (6.1-12.2) | 0.1 (0.1-0.1) | 3.1 (2.2-4.4) | 0 (0-0) | -0.24 (-0.4--0.09) |
| Acute hepatitis A | Republic of Benin | 39.3 (23.6-68.6) | 1.2 (0.8-2.1) | 16.6 (8.5-29.9) | 0.2 (0.1-0.4) | -1.65 (-2.33--0.97) |
| Acute hepatitis A | Republic of Botswana | 5.8 (3-10.4) | 0.7 (0.3-1.3) | 1.7 (1.1-2.7) | 0.1 (0.1-0.2) | -1.32 (-1.84--0.8) |
| Acute hepatitis A | Republic of Bulgaria | 4.3 (3.1-5.8) | 0.1 (0-0.1) | 1 (0.6-1.4) | 0 (0-0) | -0.04 (-0.11-0.02) |
| Acute hepatitis A | Republic of Burundi | 48 (15.4-109.1) | 1.1 (0.3-2.6) | 35.6 (7.6-118.7) | 0.4 (0.1-1.5) | -0.65 (-1.33-0.03) |
| Acute hepatitis A | Republic of Cabo Verde | 0.4 (0.2-0.9) | 0.2 (0.1-0.3) | 0.2 (0.1-0.3) | 0 (0-0.1) | -1.25 (-1.88--0.62) |
| Acute hepatitis A | Republic of Cameroon | 28.5 (13.8-60.4) | 0.4 (0.2-0.9) | 17.7 (7.5-35.9) | 0.1 (0-0.2) | -0.47 (-0.71--0.23) |
| Acute hepatitis A | Republic of Chad | 87.2 (46.1-164.2) | 2.1 (1.1-4.1) | 93.3 (47.1-168.8) | 1 (0.5-1.7) | -0.82 (-1.97-0.35) |
| Acute hepatitis A | Republic of Chile | 7.2 (6.1-8.5) | 0.1 (0.1-0.1) | 0.8 (0.6-1) | 0 (0-0) | -0.09 (-0.11--0.06) |
| Acute hepatitis A | Republic of Colombia | 17.9 (15.1-21.3) | 0.1 (0.1-0.1) | 6.8 (5.2-8.7) | 0 (0-0) | -0.07 (-0.11--0.03) |
| Acute hepatitis A | Republic of Costa Rica | 0.9 (0.7-1.3) | 0 (0-0) | 0.6 (0.5-0.8) | 0 (0-0) | -0.04 (-0.07--0.02) |
| Acute hepatitis A | Republic of Côte d'Ivoire | 74.7 (43-119.4) | 1 (0.6-1.7) | 25.1 (14.3-42) | 0.1 (0.1-0.2) | -1.5 (-2.08--0.91) |
| Acute hepatitis A | Republic of Croatia | 0.3 (0.2-0.3) | 0 (0-0) | 0 (0-0) | 0 (0-0) | -0.01 (-0.01-0) |
| Acute hepatitis A | Republic of Cuba | 6.5 (5.2-8.4) | 0.1 (0-0.1) | 0.6 (0.5-0.8) | 0 (0-0) | -0.11 (-0.15--0.07) |
| Acute hepatitis A | Republic of Cyprus | 0.2 (0.1-0.2) | 0 (0-0) | 0.1 (0.1-0.1) | 0 (0-0) | -0.06 (-0.08--0.03) |
| Acute hepatitis A | Republic of Djibouti | 2.3 (1-4.7) | 0.8 (0.3-1.6) | 3.3 (1.2-7.9) | 0.4 (0.1-0.9) | -0.53 (-1.29-0.23) |
| Acute hepatitis A | Republic of Ecuador | 7.4 (5.9-9.3) | 0.1 (0.1-0.1) | 4.9 (3.9-6.1) | 0 (0-0) | -0.1 (-0.16--0.04) |
| Acute hepatitis A | Republic of El Salvador | 3.2 (2-4.7) | 0.1 (0-0.1) | 0.5 (0.3-0.8) | 0 (0-0) | -0.08 (-0.12--0.04) |
| Acute hepatitis A | Republic of Equatorial Guinea | 0.7 (0.3-1.5) | 0.1 (0.1-0.3) | 0.1 (0-0.3) | 0 (0-0) | -0.26 (-0.33--0.18) |
| Acute hepatitis A | Republic of Estonia | 1.3 (1.1-1.6) | 0.1 (0.1-0.1) | 0.1 (0.1-0.2) | 0 (0-0) | -0.27 (-0.35--0.19) |
| Acute hepatitis A | Republic of Fiji | 1.5 (0.8-2.5) | 0.2 (0.1-0.4) | 0.3 (0.2-0.4) | 0 (0-0.1) | -0.6 (-0.79--0.42) |
| Acute hepatitis A | Republic of Finland | 0.4 (0.3-0.5) | 0 (0-0) | 0.4 (0.3-0.5) | 0 (0-0) | -0.02 (-0.03--0.01) |
| Acute hepatitis A | Republic of Ghana | 189.9 (120.1-284.9) | 2.1 (1.3-3.2) | 62.1 (36.6-97.4) | 0.3 (0.2-0.4) | -2.87 (-3.53--2.21) |
| Acute hepatitis A | Republic of Guatemala | 13.7 (8.6-22.2) | 0.1 (0.1-0.2) | 6.3 (4.8-8.4) | 0 (0-0.1) | -0.13 (-0.22--0.04) |
| Acute hepatitis A | Republic of Guinea | 106 (52.7-194.1) | 2.3 (1.2-4.2) | 44.4 (23.8-78.5) | 0.5 (0.3-1) | -1.66 (-2.74--0.56) |
| Acute hepatitis A | Republic of Guinea-Bissau | 18.7 (10.7-32.7) | 3 (1.8-5.3) | 7.8 (4.2-12.9) | 0.7 (0.4-1.1) | -1.85 (-2.92--0.77) |
| Acute hepatitis A | Republic of Guyana | 2.5 (2.1-2.9) | 0.4 (0.3-0.4) | 0.2 (0.1-0.3) | 0 (0-0) | -0.22 (-0.33--0.1) |
| Acute hepatitis A | Republic of Haiti | 53.5 (25.8-96.3) | 0.7 (0.3-1.1) | 29.3 (16-46.7) | 0.2 (0.1-0.3) | -0.43 (-0.73--0.14) |
| Acute hepatitis A | Republic of Honduras | 18.6 (9.9-29.6) | 0.3 (0.2-0.5) | 7.6 (4.6-11.7) | 0.1 (0.1-0.2) | -0.34 (-0.52--0.15) |
| Acute hepatitis A | Republic of Iceland | 0 (0-0) | 0 (0-0) | 0 (0-0) | 0 (0-0) | -0.02 (-0.03--0.01) |
| Acute hepatitis A | Republic of India | 44426.6 (36410.9-53855.4) | 5 (4.1-6) | 13658.8 (8508.1-24357.8) | 1.1 (0.7-2) | -1.15 (-2.29-0.01) |
| Acute hepatitis A | Republic of Indonesia | 4950.4 (2843-7417.6) | 3.7 (2.1-5.7) | 1940.9 (1094.2-3355.3) | 0.8 (0.4-1.3) | -1.75 (-2.55--0.94) |
| Acute hepatitis A | Republic of Iraq | 170.5 (116.8-235.1) | 1.4 (0.9-1.9) | 62 (40.5-86.8) | 0.2 (0.2-0.3) | -1.09 (-1.63--0.54) |
| Acute hepatitis A | Republic of Italy | 1.1 (0.8-1.5) | 0 (0-0) | 8.4 (6.6-10.3) | 0 (0-0) | 0.02 (-0.02-0.07) |
| Acute hepatitis A | Republic of Kazakhstan | 30.1 (19.9-41.8) | 0.2 (0.1-0.3) | 0.8 (0.6-1.2) | 0 (0-0) | -1.08 (-1.38--0.77) |
| Acute hepatitis A | Republic of Kenya | 217.9 (140.9-354.2) | 1.4 (0.8-2.3) | 197.1 (112.4-317.6) | 0.7 (0.4-1.1) | -0.62 (-1.4-0.17) |
| Acute hepatitis A | Republic of Kiribati | 2.5 (1.8-3.4) | 3.7 (2.7-5) | 1.4 (0.9-2) | 1.3 (0.9-1.8) | -1.47 (-2.54--0.38) |
| Acute hepatitis A | Republic of Korea | 22.7 (14.4-34.4) | 0.1 (0-0.1) | 3.5 (2.4-5.1) | 0 (0-0) | -0.39 (-0.49--0.28) |
| Acute hepatitis A | Republic of Latvia | 4 (3.1-5.3) | 0.2 (0.1-0.2) | 0.7 (0.5-0.9) | 0 (0-0) | -0.75 (-1.01--0.49) |
| Acute hepatitis A | Republic of Liberia | 20.7 (12.2-34.4) | 1.2 (0.7-1.9) | 11.4 (5.6-22.8) | 0.3 (0.2-0.6) | -1.43 (-2.24--0.61) |
| Acute hepatitis A | Republic of Lithuania | 1.3 (1-1.8) | 0 (0-0) | 0.1 (0.1-0.1) | 0 (0-0) | -0.24 (-0.33--0.15) |
| Acute hepatitis A | Republic of Madagascar | 106.6 (60.5-183) | 1.1 (0.6-2.1) | 58.2 (21.2-123.8) | 0.3 (0.1-0.6) | -1.15 (-1.81--0.48) |
| Acute hepatitis A | Republic of Malawi | 166.2 (88.7-306) | 1.8 (0.8-3.8) | 74.8 (28.2-163.4) | 0.6 (0.2-1.4) | -1.4 (-2.58--0.2) |
| Acute hepatitis A | Republic of Maldives | 0.9 (0.5-1.4) | 0.5 (0.2-0.8) | 0.1 (0.1-0.1) | 0 (0-0) | -1.03 (-1.28--0.78) |
| Acute hepatitis A | Republic of Mali | 21.3 (9.2-51.6) | 0.3 (0.1-0.8) | 21.7 (8.1-49) | 0.2 (0.1-0.4) | -0.14 (-0.39-0.12) |
| Acute hepatitis A | Republic of Malta | 0 (0-0.1) | 0 (0-0) | 0.1 (0-0.1) | 0 (0-0) | -0.03 (-0.05--0.01) |
| Acute hepatitis A | Republic of Mauritius | 1.7 (1.3-2.2) | 0.2 (0.2-0.3) | 0.1 (0.1-0.1) | 0 (0-0) | -0.36 (-0.44--0.27) |
| Acute hepatitis A | Republic of Moldova | 1.1 (0.5-1.9) | 0 (0-0) | 0.2 (0.1-0.2) | 0 (0-0) | -0.55 (-0.77--0.33) |
| Acute hepatitis A | Republic of Mozambique | 266.2 (126.2-555.3) | 2 (0.8-4.9) | 77.8 (26.9-177.2) | 0.4 (0.1-0.9) | -2.32 (-3.41--1.21) |
| Acute hepatitis A | Republic of Namibia | 5.2 (2.9-9) | 0.6 (0.3-1.1) | 2.2 (1.2-3.5) | 0.1 (0.1-0.2) | -0.94 (-1.51--0.37) |
| Acute hepatitis A | Republic of Nauru | 0 (0-0) | 0.2 (0.1-0.4) | 0 (0-0) | 0.1 (0-0.1) | -0.63 (-0.88--0.37) |
| Acute hepatitis A | Republic of Nicaragua | 10.5 (7.1-15.3) | 0.2 (0.1-0.3) | 1.2 (0.9-1.7) | 0 (0-0) | -0.24 (-0.33--0.15) |
| Acute hepatitis A | Republic of Niue | 0 (0-0) | 0.1 (0.1-0.2) | 0 (0-0) | 0 (0-0.1) | -0.27 (-0.37--0.17) |
| Acute hepatitis A | Republic of Palau | 0.1 (0.1-0.2) | 0.8 (0.5-1.2) | 0.1 (0-0.1) | 0.3 (0.2-0.4) | -0.78 (-1.23--0.33) |
| Acute hepatitis A | Republic of Panama | 1.2 (1-1.6) | 0.1 (0-0.1) | 0.7 (0.5-0.9) | 0 (0-0) | -0.06 (-0.1--0.02) |
| Acute hepatitis A | Republic of Paraguay | 2 (1.1-3.4) | 0.1 (0-0.1) | 0.5 (0.3-0.9) | 0 (0-0) | -0.1 (-0.18--0.03) |
| Acute hepatitis A | Republic of Peru | 24.7 (18.2-33.2) | 0.1 (0.1-0.1) | 8 (5.7-10.9) | 0 (0-0) | -0.17 (-0.24--0.11) |
| Acute hepatitis A | Republic of Poland | 1.4 (1.2-1.7) | 0 (0-0) | 2.5 (2-2.9) | 0 (0-0) | -0.01 (-0.03-0.01) |
| Acute hepatitis A | Republic of Rwanda | 156.2 (91.9-248.8) | 2.8 (1.5-4.9) | 49.7 (21.3-98.3) | 0.6 (0.2-1.1) | -2.55 (-3.5--1.6) |
| Acute hepatitis A | Republic of San Marino | 0.1 (0.1-0.2) | 0.5 (0.3-0.6) | 0.1 (0-0.1) | 0.1 (0.1-0.2) | -0.54 (-0.8--0.28) |
| Acute hepatitis A | Republic of Senegal | 22.8 (11.9-44.9) | 0.5 (0.3-0.9) | 11.4 (4.9-19.6) | 0.1 (0-0.2) | -0.66 (-1--0.31) |
| Acute hepatitis A | Republic of Serbia | 0.6 (0.3-1) | 0 (0-0) | 0.2 (0.1-0.3) | 0 (0-0) | -0.1 (-0.15--0.04) |
| Acute hepatitis A | Republic of Seychelles | 0.5 (0.3-0.8) | 0.8 (0.4-1.3) | 0.2 (0.1-0.4) | 0.2 (0.1-0.3) | -1.17 (-1.61--0.73) |
| Acute hepatitis A | Republic of Sierra Leone | 62.1 (31.3-110) | 1.9 (1.1-3.3) | 18.7 (10.3-33.3) | 0.3 (0.2-0.6) | -2.14 (-3.05--1.21) |
| Acute hepatitis A | Republic of Singapore | 0.3 (0.3-0.4) | 0 (0-0) | 0.2 (0.1-0.2) | 0 (0-0) | -0.07 (-0.11--0.04) |
| Acute hepatitis A | Republic of Slovenia | 0.4 (0.4-0.5) | 0 (0-0) | 0.1 (0.1-0.1) | 0 (0-0) | -0.04 (-0.06--0.02) |
| Acute hepatitis A | Republic of South Africa | 63 (48.4-86.7) | 0.2 (0.1-0.3) | 31.8 (23.7-43.5) | 0.1 (0-0.1) | -0.35 (-0.66--0.04) |
| Acute hepatitis A | Republic of South Sudan | 59.9 (29.7-106.2) | 1.2 (0.5-2.5) | 71 (26.3-183.3) | 1 (0.3-3) | 0.18 (-0.79-1.17) |
| Acute hepatitis A | Republic of Sudan | 1360.9 (696.2-2359.9) | 7.9 (3.1-16.7) | 322.6 (97.3-698.3) | 1.2 (0.3-2.7) | -3.04 (-4.51--1.54) |
| Acute hepatitis A | Republic of Suriname | 0.2 (0.1-0.2) | 0 (0-0.1) | 0 (0-0.1) | 0 (0-0) | -0.13 (-0.2--0.06) |
| Acute hepatitis A | Republic of Tajikistan | 51.9 (22.1-90.2) | 0.7 (0.3-1.1) | 9.9 (4-21.8) | 0.1 (0-0.2) | -1.38 (-2.09--0.66) |
| Acute hepatitis A | Republic of the Congo | 38.1 (6-126.2) | 1.5 (0.2-5.1) | 6.6 (0.9-19.2) | 0.2 (0-0.4) | -2.35 (-3.24--1.46) |
| Acute hepatitis A | Republic of the Gambia | 1.8 (0.8-3.7) | 0.3 (0.1-0.6) | 1.1 (0.4-2.5) | 0.1 (0-0.2) | -0.43 (-0.61--0.26) |
| Acute hepatitis A | Republic of the Marshall Islands | 0.1 (0-0.2) | 0.3 (0.1-0.6) | 0 (0-0.1) | 0.1 (0-0.1) | -0.5 (-0.71--0.3) |
| Acute hepatitis A | Republic of the Niger | 119.2 (56.5-236.3) | 2.1 (1.1-3.9) | 62.2 (25.1-130.2) | 0.5 (0.2-0.9) | -1.64 (-2.55--0.71) |
| Acute hepatitis A | Republic of the Philippines | 496.4 (350.2-644.8) | 0.9 (0.6-1.2) | 44.8 (35.2-59.3) | 0 (0-0.1) | -1.72 (-2.09--1.34) |
| Acute hepatitis A | Republic of the Union of Myanmar | 291.3 (120.1-512.6) | 0.9 (0.4-1.5) | 116.8 (53-192.1) | 0.2 (0.1-0.4) | -1.04 (-1.44--0.63) |
| Acute hepatitis A | Republic of Trinidad and Tobago | 0.9 (0.7-1.1) | 0.1 (0.1-0.1) | 0.3 (0.2-0.4) | 0 (0-0) | -0.12 (-0.19--0.05) |
| Acute hepatitis A | Republic of Tunisia | 28.9 (14.7-56.7) | 0.4 (0.2-0.9) | 7 (2.8-17.3) | 0.1 (0-0.1) | -0.8 (-1.27--0.33) |
| Acute hepatitis A | Republic of Turkey | 345 (213.2-519.6) | 0.6 (0.4-0.9) | 26.8 (16.9-41.6) | 0 (0-0) | -1.09 (-1.4--0.77) |
| Acute hepatitis A | Republic of Uganda | 237.9 (109.9-466.3) | 1.6 (0.6-3.8) | 144.9 (61.9-357.1) | 0.5 (0.2-1.3) | -2.18 (-3.25--1.1) |
| Acute hepatitis A | Republic of Uzbekistan | 548.1 (433.1-707.7) | 2.1 (1.6-2.6) | 23.5 (17.5-30.8) | 0.1 (0.1-0.1) | -2.21 (-2.82--1.6) |
| Acute hepatitis A | Republic of Vanuatu | 0.6 (0.2-1.4) | 0.5 (0.1-1.2) | 0.4 (0.1-0.8) | 0.1 (0-0.3) | -1.01 (-1.34--0.67) |
| Acute hepatitis A | Republic of Yemen | 865.9 (372.7-1722.6) | 7.2 (2.2-17.8) | 180.8 (70.8-369.3) | 0.9 (0.3-1.9) | -3.72 (-5.19--2.23) |
| Acute hepatitis A | Republic of Zambia | 148.9 (77.2-269.3) | 2.3 (1-4.7) | 52.6 (24.4-94.9) | 0.4 (0.2-0.8) | -2.33 (-3.46--1.2) |
| Acute hepatitis A | Republic of Zimbabwe | 72.9 (23.2-146.8) | 1.2 (0.4-2.3) | 90.8 (39.5-168.1) | 0.9 (0.4-1.6) | -0.29 (-1.02-0.44) |
| Acute hepatitis A | Romania | 4.5 (3-7.1) | 0 (0-0) | 1.8 (1.3-2.6) | 0 (0-0) | -0.08 (-0.11--0.05) |
| Acute hepatitis A | Russian Federation | 12.2 (6.9-18.5) | 0 (0-0) | 3.1 (2.6-3.7) | 0 (0-0) | -0.16 (-0.23--0.09) |
| Acute hepatitis A | Saint Kitts and Nevis | 0 (0-0) | 0.1 (0.1-0.1) | 0 (0-0) | 0 (0-0) | -0.23 (-0.31--0.15) |
| Acute hepatitis A | Saint Lucia | 0.1 (0.1-0.1) | 0.1 (0-0.1) | 0 (0-0) | 0 (0-0) | -0.18 (-0.24--0.12) |
| Acute hepatitis A | Saint Vincent and the Grenadines | 0 (0-0) | 0 (0-0) | 0 (0-0) | 0 (0-0) | -0.02 (-0.06-0.02) |
| Acute hepatitis A | Slovak Republic | 0.5 (0.3-0.8) | 0 (0-0) | 0.1 (0.1-0.2) | 0 (0-0) | -0.02 (-0.04-0.01) |
| Acute hepatitis A | Socialist Republic of Viet Nam | 781.4 (460.6-1189.1) | 1.7 (1-2.6) | 103.3 (56.4-155.2) | 0.1 (0.1-0.2) | -2.11 (-2.61--1.62) |
| Acute hepatitis A | Solomon Islands | 2.8 (0.7-8) | 1 (0.2-3) | 1.2 (0.3-2.9) | 0.2 (0-0.5) | -1.81 (-2.37--1.25) |
| Acute hepatitis A | State of Eritrea | 45.5 (21.6-77.8) | 1.9 (0.8-3.6) | 32.6 (11.6-72.9) | 0.8 (0.3-1.6) | -1.01 (-1.93--0.08) |
| Acute hepatitis A | State of Israel | 0.2 (0.2-0.3) | 0 (0-0) | 0.3 (0.2-0.4) | 0 (0-0) | -0.09 (-0.14--0.05) |
| Acute hepatitis A | State of Kuwait | 0.2 (0.1-0.3) | 0 (0-0) | 0.1 (0.1-0.2) | 0 (0-0) | -0.14 (-0.18--0.09) |
| Acute hepatitis A | State of Libya | 16.2 (6-28.8) | 0.6 (0.2-1.1) | 4 (1.5-10.4) | 0.1 (0-0.2) | -0.91 (-1.45--0.36) |
| Acute hepatitis A | State of Qatar | 0.7 (0.5-1) | 0.5 (0.4-0.7) | 0.6 (0.4-0.9) | 0.1 (0-0.1) | -1.3 (-1.75--0.86) |
| Acute hepatitis A | Sultanate of Oman | 66.1 (39.8-94.1) | 7.3 (4.2-10.7) | 22.6 (17.2-30.3) | 1 (0.8-1.4) | -1.65 (-2.66--0.63) |
| Acute hepatitis A | Swiss Confederation | 1.5 (1.2-2) | 0 (0-0) | 1.8 (1.4-2.2) | 0 (0-0) | -0.11 (-0.16--0.06) |
| Acute hepatitis A | Syrian Arab Republic | 95.7 (67.4-130) | 1.2 (0.8-1.8) | 19.8 (12.2-30.4) | 0.2 (0.1-0.2) | -1.14 (-1.5--0.79) |
| Acute hepatitis A | Taiwan (Province of China) | 0.8 (0.5-1.3) | 0 (0-0) | 0.2 (0.1-0.3) | 0 (0-0) | -0.06 (-0.08--0.04) |
| Acute hepatitis A | Togolese Republic | 6.5 (3-13) | 0.3 (0.1-0.6) | 6.5 (2.7-13.5) | 0.1 (0-0.2) | -0.21 (-0.44-0.03) |
| Acute hepatitis A | Tokelau | 0 (0-0) | 0.2 (0.1-0.3) | 0 (0-0) | 0.1 (0-0.1) | -0.44 (-0.62--0.25) |
| Acute hepatitis A | Turkmenistan | 76.3 (47.6-109.4) | 1.6 (1-2.2) | 6.1 (3.4-10.8) | 0.1 (0.1-0.2) | -1.81 (-2.44--1.18) |
| Acute hepatitis A | Tuvalu | 0 (0-0.1) | 0.3 (0.2-0.6) | 0 (0-0) | 0.1 (0-0.1) | -0.71 (-0.93--0.48) |
| Acute hepatitis A | Ukraine | 4.8 (3.2-7.3) | 0 (0-0) | 0.9 (0.6-1.4) | 0 (0-0) | -0.22 (-0.37--0.08) |
| Acute hepatitis A | Union of the Comoros | 3.4 (1.7-6.2) | 1 (0.5-1.7) | 2.5 (0.9-5.2) | 0.4 (0.2-0.9) | -0.71 (-1.4--0.01) |
| Acute hepatitis A | United Arab Emirates | 3.1 (1.6-5.3) | 0.5 (0.2-0.9) | 6.2 (3.7-9.2) | 0.1 (0.1-0.2) | -0.43 (-0.95-0.1) |
| Acute hepatitis A | United Kingdom of Great Britain and Northern Ireland | 0.9 (0.8-1.2) | 0 (0-0) | 1.5 (1.4-1.7) | 0 (0-0) | 0.01 (0-0.02) |
| Acute hepatitis A | United Mexican States | 172.3 (152.1-194.8) | 0.2 (0.2-0.2) | 62.2 (50.7-75.7) | 0.1 (0-0.1) | -0.15 (-0.28--0.03) |
| Acute hepatitis A | United Republic of Tanzania | 361.1 (197.8-633.5) | 1.6 (0.8-3.1) | 157 (76.1-304.2) | 0.4 (0.2-0.7) | -1.7 (-2.49--0.91) |
| Acute hepatitis A | United States of America | 63.7 (57.1-71.7) | 0 (0-0) | 90.3 (78-100.8) | 0 (0-0) | -0.08 (-0.11--0.05) |
| Acute hepatitis A | United States Virgin Islands | 0 (0-0) | 0 (0-0) | 0 (0-0) | 0 (0-0) | -0.03 (-0.05--0.01) |
| Acute hepatitis B | American Samoa | 0 (0-0.1) | 0.1 (0.1-0.2) | 0 (0-0.1) | 0.1 (0.1-0.1) | -0.15 (-0.25--0.04) |
| Acute hepatitis B | Antigua and Barbuda | 0 (0-0) | 0 (0-0) | 0 (0-0) | 0 (0-0) | -0.07 (-0.09--0.04) |
| Acute hepatitis B | Arab Republic of Egypt | 2962 (1129.8-5086.4) | 9.9 (3.6-17.7) | 2106.2 (791.2-3576.9) | 3.2 (1.3-5.7) | -2.56 (-4.13--0.97) |
| Acute hepatitis B | Argentine Republic | 9.6 (5.7-19.3) | 0 (0-0.1) | 4.2 (3.2-5.2) | 0 (0-0) | -0.13 (-0.17--0.09) |
| Acute hepatitis B | Australia | 0.6 (0.5-0.8) | 0 (0-0) | 2.9 (2.4-3.5) | 0 (0-0) | -0.02 (-0.03--0.01) |
| Acute hepatitis B | Barbados | 0.2 (0.1-0.2) | 0.1 (0-0.1) | 0.3 (0.2-0.3) | 0.1 (0.1-0.1) | -0.11 (-0.19--0.02) |
| Acute hepatitis B | Belize | 0 (0-0.1) | 0 (0-0) | 0 (0-0.1) | 0 (0-0) | -0.11 (-0.15--0.07) |
| Acute hepatitis B | Bermuda | 0 (0-0) | 0 (0-0) | 0 (0-0) | 0 (0-0) | -0.02 (-0.03--0.01) |
| Acute hepatitis B | Bolivarian Republic of Venezuela | 1.4 (0.8-2.3) | 0 (0-0) | 6.6 (4.4-9.5) | 0 (0-0) | -0.02 (-0.07-0.03) |
| Acute hepatitis B | Bosnia and Herzegovina | 7.4 (4.1-12.5) | 0.2 (0.1-0.3) | 4.1 (2.4-6.4) | 0.1 (0-0.1) | -0.15 (-0.26--0.04) |
| Acute hepatitis B | Brunei Darussalam | 3.2 (2.3-4.4) | 1.9 (1.3-2.6) | 1.2 (0.9-1.5) | 0.3 (0.2-0.3) | -1.79 (-2.35--1.23) |
| Acute hepatitis B | Burkina Faso | 212.6 (124.9-314.3) | 2.9 (1.8-4.2) | 249.1 (154.5-387.5) | 1.6 (1.1-2.3) | -1.31 (-2.29--0.33) |
| Acute hepatitis B | Canada | 4.8 (4.1-5.7) | 0 (0-0) | 2.7 (2.2-3.3) | 0 (0-0) | -0.05 (-0.07--0.03) |
| Acute hepatitis B | Central African Republic | 93.6 (29.6-224.3) | 4.4 (1.5-8.3) | 60.2 (28.3-115.5) | 1.6 (0.8-2.9) | -1.7 (-2.69--0.69) |
| Acute hepatitis B | Commonwealth of Dominica | 0 (0-0) | 0 (0-0.1) | 0 (0-0) | 0 (0-0) | -0.07 (-0.11--0.03) |
| Acute hepatitis B | Commonwealth of the Bahamas | 0.1 (0-0.1) | 0 (0-0) | 0.1 (0-0.1) | 0 (0-0) | -0.07 (-0.11--0.04) |
| Acute hepatitis B | Cook Islands | 0 (0-0) | 0 (0-0) | 0 (0-0) | 0 (0-0) | -0.04 (-0.05--0.02) |
| Acute hepatitis B | Czech Republic | 1.9 (1.5-2.4) | 0 (0-0) | 1.8 (1.4-2.3) | 0 (0-0) | -0.06 (-0.1--0.03) |
| Acute hepatitis B | Democratic People's Republic of Korea | 180.8 (82.6-316.6) | 1 (0.5-1.7) | 44.3 (27.6-66) | 0.1 (0.1-0.2) | -1.11 (-1.46--0.76) |
| Acute hepatitis B | Democratic Republic of Sao Tome and Principe | 1 (0.6-1.4) | 1.1 (0.7-1.5) | 0.4 (0.3-0.6) | 0.3 (0.2-0.4) | -1.31 (-1.78--0.84) |
| Acute hepatitis B | Democratic Republic of the Congo | 70.5 (28.6-150.3) | 0.3 (0.1-0.6) | 96.8 (35.4-185.5) | 0.2 (0.1-0.3) | -0.35 (-0.6--0.1) |
| Acute hepatitis B | Democratic Republic of Timor-Leste | 0.8 (0.1-1.8) | 0.2 (0-0.4) | 0.8 (0.2-2.1) | 0.1 (0-0.2) | -0.61 (-0.81--0.42) |
| Acute hepatitis B | Democratic Socialist Republic of Sri Lanka | 32.9 (18.5-53.6) | 0.3 (0.1-0.4) | 16.4 (7.9-25.4) | 0.1 (0-0.1) | -0.61 (-0.77--0.45) |
| Acute hepatitis B | Dominican Republic | 1.6 (1-2.4) | 0 (0-0) | 3.6 (2.2-5.3) | 0 (0-0) | -0.08 (-0.14--0.02) |
| Acute hepatitis B | Eastern Republic of Uruguay | 0.2 (0.1-0.2) | 0 (0-0) | 0.1 (0.1-0.1) | 0 (0-0) | -0.05 (-0.06--0.03) |
| Acute hepatitis B | Federal Democratic Republic of Ethiopia | 1153.6 (320.7-2073.2) | 4.5 (1.2-8) | 1590.8 (580.9-2443.6) | 2.6 (0.9-4) | -1.93 (-2.95--0.89) |
| Acute hepatitis B | Federal Democratic Republic of Nepal | 70 (10.4-217) | 0.5 (0.1-1.4) | 155.4 (46.3-342.3) | 0.6 (0.2-1.2) | -1.48 (-2.49--0.46) |
| Acute hepatitis B | Federal Republic of Germany | 48.5 (38.5-58.1) | 0 (0-0.1) | 8.6 (6.6-11) | 0 (0-0) | -0.07 (-0.09--0.04) |
| Acute hepatitis B | Federal Republic of Nigeria | 7380.5 (3123.6-13374) | 9.4 (3.8-17.8) | 2450.9 (1236.6-4042.4) | 1.4 (0.7-2.2) | -4.49 (-5.68--3.29) |
| Acute hepatitis B | Federal Republic of Somalia | 548.8 (215.7-949.6) | 13.8 (5.4-24) | 1337.6 (518.4-2297.7) | 12.8 (5-21.1) | -0.59 (-2.19-1.04) |
| Acute hepatitis B | Federated States of Micronesia | 0.4 (0.2-0.6) | 0.4 (0.2-0.7) | 0.1 (0-0.2) | 0.1 (0-0.2) | -0.65 (-0.85--0.45) |
| Acute hepatitis B | Federative Republic of Brazil | 55.6 (46.9-75.4) | 0 (0-0.1) | 91.5 (83.4-104.7) | 0 (0-0) | -0.18 (-0.26--0.1) |
| Acute hepatitis B | French Republic | 10.9 (9.3-12.5) | 0 (0-0) | 7.4 (5.8-9) | 0 (0-0) | -0.04 (-0.06--0.02) |
| Acute hepatitis B | Gabonese Republic | 10.5 (5.4-22) | 1.5 (0.7-3.1) | 2.2 (1-4.7) | 0.2 (0.1-0.4) | -1.26 (-1.67--0.85) |
| Acute hepatitis B | Georgia | 6.7 (4.2-9.4) | 0.1 (0.1-0.2) | 1.3 (0.9-1.7) | 0 (0-0) | -0.41 (-0.52--0.29) |
| Acute hepatitis B | Grand Duchy of Luxembourg | 0 (0-0) | 0 (0-0) | 0 (0-0) | 0 (0-0) | -0.02 (-0.03--0.01) |
| Acute hepatitis B | Greenland | 0 (0-0) | 0.1 (0-0.1) | 0 (0-0) | 0 (0-0) | -0.15 (-0.19--0.11) |
| Acute hepatitis B | Grenada | 0.1 (0.1-0.2) | 0.1 (0.1-0.2) | 0.1 (0-0.1) | 0 (0-0.1) | -0.16 (-0.24--0.09) |
| Acute hepatitis B | Guam | 0.1 (0-0.1) | 0.1 (0-0.1) | 0.1 (0.1-0.1) | 0.1 (0-0.1) | -0.02 (-0.08-0.04) |
| Acute hepatitis B | Hashemite Kingdom of Jordan | 15.4 (8.8-24) | 0.8 (0.5-1.3) | 10.6 (6.8-15.9) | 0.1 (0.1-0.2) | -1.09 (-1.4--0.78) |
| Acute hepatitis B | Hellenic Republic | 9.4 (8.2-10.7) | 0.1 (0.1-0.1) | 8.5 (7.2-10.1) | 0 (0-0.1) | -0.09 (-0.18--0.01) |
| Acute hepatitis B | Hungary | 3.3 (2.6-4.2) | 0 (0-0) | 2 (1.4-2.9) | 0 (0-0) | -0.04 (-0.07--0.01) |
| Acute hepatitis B | Independent State of Papua New Guinea | 38.3 (13.1-80) | 1.3 (0.4-2.6) | 20.9 (5.5-47.1) | 0.3 (0.1-0.6) | -1.69 (-2.17--1.2) |
| Acute hepatitis B | Independent State of Samoa | 0.2 (0.1-0.3) | 0.2 (0.1-0.2) | 0.1 (0.1-0.2) | 0.1 (0.1-0.1) | -0.19 (-0.29--0.08) |
| Acute hepatitis B | Ireland | 0 (0-0.1) | 0 (0-0) | 0 (0-0.1) | 0 (0-0) | -0.02 (-0.03--0.01) |
| Acute hepatitis B | Islamic Republic of Afghanistan | 1393.4 (517.7-2300.1) | 18.4 (7.3-30.2) | 911.5 (482-1448.5) | 7.2 (3.9-11.4) | -3 (-4.94--1.03) |
| Acute hepatitis B | Islamic Republic of Iran | 492.6 (331.3-713.1) | 1.5 (1-2.2) | 410.8 (345.6-486.2) | 0.5 (0.4-0.6) | -1.18 (-1.71--0.64) |
| Acute hepatitis B | Islamic Republic of Mauritania | 129.1 (40.8-265.2) | 9.1 (2.8-18.8) | 37.1 (19.8-62.8) | 1.3 (0.7-2.2) | -3.17 (-4.32--2.01) |
| Acute hepatitis B | Islamic Republic of Pakistan | 1097.3 (365.1-2565.4) | 1.4 (0.4-3.4) | 2474 (1230.1-4292.9) | 1.6 (0.8-2.7) | -1.06 (-2.17-0.07) |
| Acute hepatitis B | Jamaica | 0.5 (0.3-0.7) | 0 (0-0) | 1 (0.8-1.4) | 0 (0-0) | -0.05 (-0.09--0.01) |
| Acute hepatitis B | Japan | 26.9 (24.3-30) | 0 (0-0) | 55.7 (48.2-62) | 0 (0-0) | -0.21 (-0.32--0.1) |
| Acute hepatitis B | Kingdom of Bahrain | 5 (3.3-6.9) | 2.6 (1.7-3.6) | 7.5 (4.5-11.6) | 0.8 (0.5-1.2) | -1.58 (-2.34--0.81) |
| Acute hepatitis B | Kingdom of Belgium | 4.3 (3.7-5) | 0 (0-0) | 2.4 (2-2.9) | 0 (0-0) | -0.06 (-0.09--0.03) |
| Acute hepatitis B | Kingdom of Bhutan | 3.5 (1.2-7.7) | 0.8 (0.3-1.7) | 3.6 (1.6-6.6) | 0.5 (0.2-0.9) | -1.92 (-2.71--1.13) |
| Acute hepatitis B | Kingdom of Cambodia | 206.8 (86.3-366.8) | 3.7 (1.5-6.6) | 153 (60.6-264.9) | 1.1 (0.5-1.9) | -3.96 (-5.07--2.84) |
| Acute hepatitis B | Kingdom of Denmark | 1.7 (1.5-2) | 0 (0-0) | 1 (0.8-1.2) | 0 (0-0) | -0.05 (-0.08--0.02) |
| Acute hepatitis B | Kingdom of Eswatini | 10.2 (4.4-17.6) | 2.9 (1.3-4.9) | 7.2 (4.2-11.7) | 1 (0.6-1.6) | -1.35 (-2.23--0.47) |
| Acute hepatitis B | Kingdom of Lesotho | 28.7 (13-48) | 3.2 (1.5-5.2) | 22.2 (13.8-34.1) | 1.8 (1.2-2.7) | -0.63 (-1.66-0.42) |
| Acute hepatitis B | Kingdom of Morocco | 174.3 (83.8-301) | 1.1 (0.5-2) | 227.8 (149.1-350.5) | 0.6 (0.4-1) | -0.94 (-1.48--0.39) |
| Acute hepatitis B | Kingdom of Norway | 0.1 (0.1-0.1) | 0 (0-0) | 0.5 (0.4-0.6) | 0 (0-0) | 0 (-0.01-0.01) |
| Acute hepatitis B | Kingdom of Saudi Arabia | 335.7 (156.8-654.8) | 4.5 (2-9.2) | 106.5 (76-155.5) | 0.4 (0.3-0.6) | -2.76 (-3.4--2.12) |
| Acute hepatitis B | Kingdom of Spain | 2.4 (1.9-2.8) | 0 (0-0) | 1.9 (1.5-2.4) | 0 (0-0) | -0.02 (-0.03--0.01) |
| Acute hepatitis B | Kingdom of Sweden | 0.1 (0-0.1) | 0 (0-0) | 0.7 (0.5-0.8) | 0 (0-0) | 0 (0-0.01) |
| Acute hepatitis B | Kingdom of Thailand | 286.5 (208.3-379.2) | 0.7 (0.5-0.9) | 163.5 (122.1-212.8) | 0.2 (0.1-0.2) | -0.42 (-0.73--0.12) |
| Acute hepatitis B | Kingdom of the Netherlands | 1.1 (1-1.3) | 0 (0-0) | 0.5 (0.4-0.6) | 0 (0-0) | -0.02 (-0.03--0.01) |
| Acute hepatitis B | Kingdom of Tonga | 5.2 (3-7.2) | 7.3 (4.4-10.1) | 1.5 (0.9-2.4) | 1.7 (1-2.7) | -2.7 (-3.75--1.65) |
| Acute hepatitis B | Kyrgyz Republic | 55.4 (42-73.4) | 1.2 (0.9-1.5) | 6.9 (4.4-10.4) | 0.1 (0.1-0.2) | -1.66 (-2.1--1.21) |
| Acute hepatitis B | Lao People's Democratic Republic | 52.2 (19.7-100.4) | 1.8 (0.8-3.4) | 24.7 (12.5-44) | 0.4 (0.2-0.7) | -2.97 (-3.67--2.26) |
| Acute hepatitis B | Lebanese Republic | 5.9 (2.8-10.3) | 0.3 (0.1-0.4) | 7.4 (5.1-9.9) | 0.1 (0.1-0.2) | -0.6 (-0.83--0.37) |
| Acute hepatitis B | Malaysia | 157.1 (119.7-204.4) | 1.4 (1-1.8) | 203.7 (160.2-259.5) | 0.7 (0.5-0.9) | -0.54 (-1.13-0.05) |
| Acute hepatitis B | Mongolia | 27.1 (13.5-50.7) | 1.2 (0.6-2.1) | 4.3 (2.7-6.5) | 0.1 (0.1-0.2) | -1.28 (-1.69--0.87) |
| Acute hepatitis B | Montenegro | 0 (0-0.1) | 0 (0-0) | 0 (0-0.1) | 0 (0-0) | -0.01 (-0.01-0) |
| Acute hepatitis B | New Zealand | 0.5 (0.4-0.5) | 0 (0-0) | 0 (0-0) | 0 (0-0) | -0.03 (-0.04--0.02) |
| Acute hepatitis B | North Macedonia | 0.8 (0.4-1.3) | 0 (0-0.1) | 0.8 (0.4-1.5) | 0 (0-0.1) | -0.07 (-0.11--0.02) |
| Acute hepatitis B | Northern Mariana Islands | 0.1 (0.1-0.1) | 0.2 (0.2-0.3) | 0.1 (0-0.1) | 0.1 (0.1-0.2) | -0.22 (-0.36--0.08) |
| Acute hepatitis B | Palestine | 8.3 (4.5-13.7) | 0.8 (0.4-1.4) | 5.1 (3.8-6.6) | 0.2 (0.1-0.2) | -0.96 (-1.27--0.65) |
| Acute hepatitis B | People's Democratic Republic of Algeria | 201.4 (117.8-289.2) | 1.3 (0.8-1.9) | 211.6 (126-311.1) | 0.6 (0.4-0.9) | -0.82 (-1.34--0.28) |
| Acute hepatitis B | People's Republic of Bangladesh | 867.8 (193.1-1901.7) | 1.4 (0.3-3.1) | 1079.9 (413.7-1870.6) | 0.7 (0.3-1.3) | -2.05 (-3.06--1.02) |
| Acute hepatitis B | People's Republic of China | 16094.5 (11438.9-21647.8) | 1.7 (1.2-2.2) | 2424 (1835.1-3239.6) | 0.1 (0.1-0.2) | -2.13 (-2.58--1.67) |
| Acute hepatitis B | Plurinational State of Bolivia | 1.7 (0.8-3.7) | 0 (0-0.1) | 2.9 (1.8-4.4) | 0 (0-0) | -0.19 (-0.31--0.08) |
| Acute hepatitis B | Portuguese Republic | 8.9 (7.8-10) | 0.1 (0.1-0.1) | 6.2 (5.1-7.5) | 0 (0-0) | -0.14 (-0.22--0.07) |
| Acute hepatitis B | Principality of Andorra | 0.1 (0.1-0.2) | 0.3 (0.1-0.4) | 0.3 (0.2-0.5) | 0.2 (0.1-0.3) | -0.36 (-0.62--0.1) |
| Acute hepatitis B | Principality of Monaco | 0 (0-0) | 0 (0-0) | 0 (0-0) | 0 (0-0) | 0 (-0.01-0.01) |
| Acute hepatitis B | Puerto Rico | 0.1 (0.1-0.2) | 0 (0-0) | 0.8 (0.6-0.9) | 0 (0-0) | -0.12 (-0.17--0.07) |
| Acute hepatitis B | Republic of Albania | 1.6 (0.9-2.8) | 0 (0-0.1) | 0.9 (0.4-1.7) | 0 (0-0.1) | -0.15 (-0.21--0.09) |
| Acute hepatitis B | Republic of Angola | 676.7 (150.5-1567.2) | 8.9 (2.1-16.1) | 255.3 (140.2-427.6) | 1.2 (0.7-1.8) | -3.85 (-4.97--2.71) |
| Acute hepatitis B | Republic of Armenia | 10.6 (7.6-14.6) | 0.3 (0.2-0.4) | 0.8 (0.5-1.1) | 0 (0-0) | -0.38 (-0.5--0.27) |
| Acute hepatitis B | Republic of Austria | 1 (0.8-1.1) | 0 (0-0) | 0.5 (0.4-0.6) | 0 (0-0) | -0.02 (-0.04--0.01) |
| Acute hepatitis B | Republic of Azerbaijan | 52.1 (35.8-72.7) | 0.7 (0.5-0.9) | 12.1 (6.6-19.9) | 0.1 (0.1-0.2) | -0.84 (-1.1--0.58) |
| Acute hepatitis B | Republic of Belarus | 21.2 (17.8-25.5) | 0.2 (0.2-0.3) | 9.1 (6.6-11.6) | 0.1 (0.1-0.1) | -0.24 (-0.4--0.09) |
| Acute hepatitis B | Republic of Benin | 68.4 (48.8-97.8) | 1.9 (1.3-2.7) | 47.5 (29-71.7) | 0.6 (0.3-0.8) | -1.65 (-2.33--0.97) |
| Acute hepatitis B | Republic of Botswana | 10.6 (5.1-18.5) | 1.6 (0.8-2.8) | 6.8 (3.7-10.9) | 0.4 (0.2-0.6) | -1.32 (-1.84--0.8) |
| Acute hepatitis B | Republic of Bulgaria | 2.9 (2.1-4) | 0 (0-0) | 4.5 (3.1-6.5) | 0 (0-0.1) | -0.04 (-0.11-0.02) |
| Acute hepatitis B | Republic of Burundi | 49.6 (5.4-111.8) | 1.5 (0.2-3.5) | 81 (17.9-173.7) | 1.1 (0.2-2.5) | -0.65 (-1.33-0.03) |
| Acute hepatitis B | Republic of Cabo Verde | 4 (3.2-5) | 1.5 (1.2-2) | 2.1 (1.5-2.9) | 0.4 (0.3-0.6) | -1.25 (-1.88--0.62) |
| Acute hepatitis B | Republic of Cameroon | 23.2 (9.3-55.6) | 0.3 (0.1-0.8) | 31.7 (14.9-61.2) | 0.2 (0.1-0.3) | -0.47 (-0.71--0.23) |
| Acute hepatitis B | Republic of Chad | 171.6 (107.6-263.3) | 3.8 (2.3-6.2) | 304.2 (207.1-452.4) | 3 (1.9-4.4) | -0.82 (-1.97-0.35) |
| Acute hepatitis B | Republic of Chile | 0.4 (0.3-0.5) | 0 (0-0) | 0.8 (0.7-1) | 0 (0-0) | -0.09 (-0.11--0.06) |
| Acute hepatitis B | Republic of Colombia | 2.8 (2.3-3.5) | 0 (0-0) | 6.8 (5.3-9) | 0 (0-0) | -0.07 (-0.11--0.03) |
| Acute hepatitis B | Republic of Costa Rica | 0.2 (0.1-0.3) | 0 (0-0) | 0.4 (0.4-0.5) | 0 (0-0) | -0.04 (-0.07--0.02) |
| Acute hepatitis B | Republic of Côte d'Ivoire | 151.2 (94.8-227.6) | 1.9 (1.2-2.8) | 67.6 (41.5-101.8) | 0.4 (0.2-0.6) | -1.5 (-2.08--0.91) |
| Acute hepatitis B | Republic of Croatia | 0.3 (0.3-0.4) | 0 (0-0) | 0.2 (0.2-0.3) | 0 (0-0) | -0.01 (-0.01-0) |
| Acute hepatitis B | Republic of Cuba | 2.5 (1.8-3.4) | 0 (0-0) | 1.7 (1.4-2) | 0 (0-0) | -0.11 (-0.15--0.07) |
| Acute hepatitis B | Republic of Cyprus | 0.3 (0.2-0.4) | 0 (0-0) | 0.2 (0.2-0.3) | 0 (0-0) | -0.06 (-0.08--0.03) |
| Acute hepatitis B | Republic of Djibouti | 3.6 (1.6-6.3) | 1.6 (0.7-2.7) | 10.8 (6.1-17.6) | 1.2 (0.7-2) | -0.53 (-1.29-0.23) |
| Acute hepatitis B | Republic of Ecuador | 1.1 (0.7-1.6) | 0 (0-0) | 2 (1.5-2.5) | 0 (0-0) | -0.1 (-0.16--0.04) |
| Acute hepatitis B | Republic of El Salvador | 2.2 (1.3-3.3) | 0 (0-0.1) | 1.7 (1.1-2.4) | 0 (0-0) | -0.08 (-0.12--0.04) |
| Acute hepatitis B | Republic of Equatorial Guinea | 0.2 (0-0.5) | 0.1 (0-0.2) | 0.3 (0.1-0.7) | 0 (0-0.1) | -0.26 (-0.33--0.18) |
| Acute hepatitis B | Republic of Estonia | 1.8 (1.6-2.1) | 0.1 (0.1-0.2) | 0.2 (0.1-0.2) | 0 (0-0) | -0.27 (-0.35--0.19) |
| Acute hepatitis B | Republic of Fiji | 2 (1.2-2.8) | 0.4 (0.2-0.5) | 0.6 (0.4-0.9) | 0.1 (0-0.1) | -0.6 (-0.79--0.42) |
| Acute hepatitis B | Republic of Finland | 0.7 (0.6-0.9) | 0 (0-0) | 0.3 (0.2-0.4) | 0 (0-0) | -0.02 (-0.03--0.01) |
| Acute hepatitis B | Republic of Ghana | 300.6 (169.2-457.3) | 2.8 (1.6-4.2) | 53.4 (30.3-90.9) | 0.2 (0.1-0.4) | -2.87 (-3.53--2.21) |
| Acute hepatitis B | Republic of Guatemala | 1.6 (0.8-3) | 0 (0-0) | 2.2 (1.5-3.1) | 0 (0-0) | -0.13 (-0.22--0.04) |
| Acute hepatitis B | Republic of Guinea | 236.4 (132-378.9) | 4.6 (2.6-7.7) | 160.9 (101.3-251.6) | 1.9 (1.2-2.9) | -1.66 (-2.74--0.56) |
| Acute hepatitis B | Republic of Guinea-Bissau | 32.1 (17.8-50.7) | 4.6 (2.5-7.2) | 19.7 (13.4-27.8) | 1.6 (1.1-2.3) | -1.85 (-2.92--0.77) |
| Acute hepatitis B | Republic of Guyana | 0.1 (0-0.1) | 0 (0-0) | 0.5 (0.3-0.6) | 0.1 (0-0.1) | -0.22 (-0.33--0.1) |
| Acute hepatitis B | Republic of Haiti | 5.4 (1.8-15.3) | 0.1 (0-0.2) | 11.7 (6-21.3) | 0.1 (0.1-0.2) | -0.43 (-0.73--0.14) |
| Acute hepatitis B | Republic of Honduras | 3.8 (1.6-7.4) | 0.1 (0-0.1) | 5.4 (3-8.8) | 0.1 (0-0.1) | -0.34 (-0.52--0.15) |
| Acute hepatitis B | Republic of Iceland | 0 (0-0) | 0 (0-0) | 0 (0-0) | 0 (0-0) | -0.02 (-0.03--0.01) |
| Acute hepatitis B | Republic of India | 5533.7 (2775-10044.9) | 0.7 (0.4-1.3) | 10871.6 (5998.3-14817.8) | 0.8 (0.5-1.2) | -1.15 (-2.29-0.01) |
| Acute hepatitis B | Republic of Indonesia | 1447.2 (542.1-2696.8) | 1.2 (0.4-2.3) | 2170.1 (1048.4-3596.5) | 0.8 (0.4-1.4) | -1.75 (-2.55--0.94) |
| Acute hepatitis B | Republic of Iraq | 93.6 (59.8-135.8) | 0.9 (0.5-1.3) | 119.3 (78-165.7) | 0.4 (0.3-0.6) | -1.09 (-1.63--0.54) |
| Acute hepatitis B | Republic of Italy | 4.6 (3.4-6.3) | 0 (0-0) | 26.9 (22.6-30.7) | 0 (0-0) | 0.02 (-0.02-0.07) |
| Acute hepatitis B | Republic of Kazakhstan | 140.4 (109.7-175.2) | 0.8 (0.7-1.1) | 15.1 (10.3-21.7) | 0.1 (0.1-0.1) | -1.08 (-1.38--0.77) |
| Acute hepatitis B | Republic of Kenya | 200 (64.9-303.8) | 1.6 (0.5-2.6) | 342.8 (153.8-523.9) | 1.1 (0.5-1.8) | -0.62 (-1.4-0.17) |
| Acute hepatitis B | Republic of Kiribati | 1.8 (1.3-2.5) | 3.1 (2.2-4.2) | 1.2 (0.8-1.9) | 1.2 (0.7-1.8) | -1.47 (-2.54--0.38) |
| Acute hepatitis B | Republic of Korea | 83.9 (61.9-108.4) | 0.2 (0.2-0.3) | 9.5 (6.4-12.9) | 0 (0-0) | -0.39 (-0.49--0.28) |
| Acute hepatitis B | Republic of Latvia | 15 (13.1-17.1) | 0.6 (0.5-0.7) | 1.1 (0.8-1.3) | 0.1 (0-0.1) | -0.75 (-1.01--0.49) |
| Acute hepatitis B | Republic of Liberia | 36.5 (25.1-50) | 1.8 (1.3-2.6) | 32.7 (20.2-50.3) | 0.9 (0.6-1.4) | -1.43 (-2.24--0.61) |
| Acute hepatitis B | Republic of Lithuania | 5.6 (4.7-6.6) | 0.2 (0.1-0.2) | 0.7 (0.5-0.8) | 0 (0-0) | -0.24 (-0.33--0.15) |
| Acute hepatitis B | Republic of Madagascar | 126 (65.4-172.4) | 1.8 (0.9-2.4) | 124.3 (57.4-199.6) | 0.7 (0.3-1.1) | -1.15 (-1.81--0.48) |
| Acute hepatitis B | Republic of Malawi | 221.2 (127.7-332.6) | 3.6 (2.1-5.3) | 271 (185.3-387.8) | 2.4 (1.7-3.4) | -1.4 (-2.58--0.2) |
| Acute hepatitis B | Republic of Maldives | 0.7 (0.4-1.3) | 0.6 (0.3-0.9) | 0.3 (0.2-0.4) | 0.1 (0.1-0.1) | -1.03 (-1.28--0.78) |
| Acute hepatitis B | Republic of Mali | 14 (4-35.4) | 0.2 (0.1-0.5) | 33.1 (13.2-61.4) | 0.2 (0.1-0.5) | -0.14 (-0.39-0.12) |
| Acute hepatitis B | Republic of Malta | 0.1 (0-0.1) | 0 (0-0) | 0 (0-0.1) | 0 (0-0) | -0.03 (-0.05--0.01) |
| Acute hepatitis B | Republic of Mauritius | 0.1 (0.1-0.3) | 0 (0-0) | 0 (0-0.1) | 0 (0-0) | -0.36 (-0.44--0.27) |
| Acute hepatitis B | Republic of Moldova | 25.8 (21-31.4) | 0.6 (0.5-0.8) | 2.1 (1.6-2.7) | 0.1 (0.1-0.1) | -0.55 (-0.77--0.33) |
| Acute hepatitis B | Republic of Mozambique | 432.2 (196.7-723.7) | 5.1 (2.6-8.5) | 260 (163.5-398.2) | 1.5 (0.9-2.3) | -2.32 (-3.41--1.21) |
| Acute hepatitis B | Republic of Namibia | 9.4 (4.4-15.4) | 1.3 (0.6-2.1) | 8.8 (4.9-13.9) | 0.5 (0.3-0.8) | -0.94 (-1.51--0.37) |
| Acute hepatitis B | Republic of Nauru | 0 (0-0) | 0.4 (0.2-0.6) | 0 (0-0) | 0.1 (0.1-0.2) | -0.63 (-0.88--0.37) |
| Acute hepatitis B | Republic of Nicaragua | 1.9 (1-3.7) | 0 (0-0.1) | 1.3 (0.7-1.9) | 0 (0-0) | -0.24 (-0.33--0.15) |
| Acute hepatitis B | Republic of Niue | 0 (0-0) | 0.2 (0.1-0.2) | 0 (0-0) | 0.1 (0-0.1) | -0.27 (-0.37--0.17) |
| Acute hepatitis B | Republic of Palau | 0.1 (0.1-0.2) | 0.9 (0.6-1.4) | 0.1 (0.1-0.1) | 0.3 (0.2-0.5) | -0.78 (-1.23--0.33) |
| Acute hepatitis B | Republic of Panama | 0.4 (0.3-0.5) | 0 (0-0) | 0.7 (0.5-0.9) | 0 (0-0) | -0.06 (-0.1--0.02) |
| Acute hepatitis B | Republic of Paraguay | 3.1 (1.6-5.3) | 0.1 (0-0.2) | 3.5 (2.4-4.9) | 0.1 (0-0.1) | -0.1 (-0.18--0.03) |
| Acute hepatitis B | Republic of Peru | 7.2 (4.6-11.4) | 0 (0-0.1) | 5.2 (3.2-7.4) | 0 (0-0) | -0.17 (-0.24--0.11) |
| Acute hepatitis B | Republic of Poland | 0.9 (0.7-1.1) | 0 (0-0) | 2.9 (2.4-3.4) | 0 (0-0) | -0.01 (-0.03-0.01) |
| Acute hepatitis B | Republic of Rwanda | 127.6 (57.9-229.1) | 3.1 (1.3-5.5) | 97.8 (47.3-141.7) | 1.1 (0.6-1.6) | -2.55 (-3.5--1.6) |
| Acute hepatitis B | Republic of San Marino | 0.1 (0.1-0.1) | 0.3 (0.2-0.4) | 0.1 (0-0.1) | 0.1 (0.1-0.2) | -0.54 (-0.8--0.28) |
| Acute hepatitis B | Republic of Senegal | 32.5 (20.9-49.4) | 0.6 (0.4-0.9) | 28.4 (16-46.6) | 0.3 (0.2-0.4) | -0.66 (-1--0.31) |
| Acute hepatitis B | Republic of Serbia | 8.1 (5.2-11.7) | 0.1 (0.1-0.1) | 4.1 (2.6-5.7) | 0 (0-0) | -0.1 (-0.15--0.04) |
| Acute hepatitis B | Republic of Seychelles | 0.5 (0.2-0.9) | 0.8 (0.4-1.6) | 0.4 (0.2-0.7) | 0.3 (0.2-0.5) | -1.17 (-1.61--0.73) |
| Acute hepatitis B | Republic of Sierra Leone | 134.8 (68.7-224.9) | 3.8 (2-6.1) | 48.6 (30.7-69.6) | 0.8 (0.5-1.2) | -2.14 (-3.05--1.21) |
| Acute hepatitis B | Republic of Singapore | 0.2 (0.1-0.2) | 0 (0-0) | 0.3 (0.2-0.3) | 0 (0-0) | -0.07 (-0.11--0.04) |
| Acute hepatitis B | Republic of Slovenia | 0.6 (0.6-0.8) | 0 (0-0) | 0.4 (0.3-0.5) | 0 (0-0) | -0.04 (-0.06--0.02) |
| Acute hepatitis B | Republic of South Africa | 137.4 (89.7-184.5) | 0.5 (0.4-0.7) | 142.4 (108.6-184.9) | 0.3 (0.2-0.4) | -0.35 (-0.66--0.04) |
| Acute hepatitis B | Republic of South Sudan | 87.2 (39.8-147.2) | 2.6 (1.1-4.2) | 149 (79.5-239.5) | 2.8 (1.5-4.5) | 0.18 (-0.79-1.17) |
| Acute hepatitis B | Republic of Sudan | 1161.2 (440.6-2297.7) | 10.3 (4-20.5) | 582.8 (247.7-1051.8) | 2.5 (1.1-4.6) | -3.04 (-4.51--1.54) |
| Acute hepatitis B | Republic of Suriname | 0.3 (0.2-0.5) | 0.1 (0.1-0.1) | 0.3 (0.2-0.4) | 0.1 (0-0.1) | -0.13 (-0.2--0.06) |
| Acute hepatitis B | Republic of Tajikistan | 177.2 (130.2-231.3) | 2.7 (2.1-3.3) | 83 (43.9-162.3) | 0.8 (0.4-1.5) | -1.38 (-2.09--0.66) |
| Acute hepatitis B | Republic of the Congo | 78.5 (20.2-186.7) | 4.3 (1.2-8.7) | 27.7 (9.4-56) | 0.7 (0.2-1.4) | -2.35 (-3.24--1.46) |
| Acute hepatitis B | Republic of the Gambia | 1.8 (0.9-3.3) | 0.3 (0.1-0.5) | 1.7 (0.7-3.6) | 0.1 (0-0.2) | -0.43 (-0.61--0.26) |
| Acute hepatitis B | Republic of the Marshall Islands | 0.1 (0-0.1) | 0.2 (0.1-0.3) | 0 (0-0.1) | 0.1 (0-0.1) | -0.5 (-0.71--0.3) |
| Acute hepatitis B | Republic of the Niger | 232.4 (132.6-349) | 3.7 (2.4-5.6) | 178.8 (106.1-291.9) | 1.3 (0.7-2.2) | -1.64 (-2.55--0.71) |
| Acute hepatitis B | Republic of the Philippines | 540.1 (347.4-771.2) | 1.1 (0.7-1.5) | 100.8 (79.3-124.3) | 0.1 (0.1-0.1) | -1.72 (-2.09--1.34) |
| Acute hepatitis B | Republic of the Union of Myanmar | 62.7 (9.4-137.7) | 0.2 (0-0.5) | 77.5 (23.9-163.6) | 0.1 (0-0.3) | -1.04 (-1.44--0.63) |
| Acute hepatitis B | Republic of Trinidad and Tobago | 0.6 (0.4-0.8) | 0.1 (0-0.1) | 0.9 (0.6-1.2) | 0.1 (0-0.1) | -0.12 (-0.19--0.05) |
| Acute hepatitis B | Republic of Tunisia | 76.9 (42.4-117.9) | 1.3 (0.7-2.1) | 63.8 (39.9-97.6) | 0.5 (0.3-0.7) | -0.8 (-1.27--0.33) |
| Acute hepatitis B | Republic of Turkey | 338.7 (190.5-543) | 0.7 (0.4-1) | 149.5 (112.1-193.8) | 0.2 (0.1-0.2) | -1.09 (-1.4--0.77) |
| Acute hepatitis B | Republic of Uganda | 440.3 (216.8-715.6) | 4.7 (2.2-7.6) | 385.4 (219-608.2) | 1.7 (0.9-2.6) | -2.18 (-3.25--1.1) |
| Acute hepatitis B | Republic of Uzbekistan | 554.2 (413-708.6) | 2.2 (1.7-2.8) | 63.5 (49.3-80.6) | 0.2 (0.2-0.2) | -2.21 (-2.82--1.6) |
| Acute hepatitis B | Republic of Vanuatu | 0.7 (0.3-1.4) | 0.7 (0.3-1.3) | 0.4 (0.1-0.7) | 0.1 (0.1-0.3) | -1.01 (-1.34--0.67) |
| Acute hepatitis B | Republic of Yemen | 840.5 (258.7-1847.5) | 12.5 (4.4-25) | 432.4 (199.5-737.2) | 2.6 (1.2-4.3) | -3.72 (-5.19--2.23) |
| Acute hepatitis B | Republic of Zambia | 229.5 (123.1-362.5) | 5 (2.8-7.5) | 172.6 (109.6-249.1) | 1.5 (1-2.2) | -2.33 (-3.46--1.2) |
| Acute hepatitis B | Republic of Zimbabwe | 99.4 (21.3-195.7) | 2.1 (0.5-4.1) | 81.3 (31.3-187) | 0.9 (0.4-1.9) | -0.29 (-1.02-0.44) |
| Acute hepatitis B | Romania | 11.3 (7.7-16.8) | 0.1 (0-0.1) | 4.1 (3.1-5.3) | 0 (0-0) | -0.08 (-0.11--0.05) |
| Acute hepatitis B | Russian Federation | 125.3 (110.3-136.9) | 0.1 (0.1-0.1) | 24.1 (21-26.5) | 0 (0-0) | -0.16 (-0.23--0.09) |
| Acute hepatitis B | Saint Kitts and Nevis | 0 (0-0.1) | 0.1 (0.1-0.2) | 0 (0-0) | 0 (0-0) | -0.23 (-0.31--0.15) |
| Acute hepatitis B | Saint Lucia | 0.1 (0.1-0.1) | 0.1 (0.1-0.1) | 0.1 (0-0.1) | 0 (0-0) | -0.18 (-0.24--0.12) |
| Acute hepatitis B | Saint Vincent and the Grenadines | 0 (0-0) | 0 (0-0) | 0 (0-0) | 0 (0-0) | -0.02 (-0.06-0.02) |
| Acute hepatitis B | Slovak Republic | 1.2 (0.7-1.7) | 0 (0-0) | 1.3 (0.7-2.2) | 0 (0-0) | -0.02 (-0.04-0.01) |
| Acute hepatitis B | Socialist Republic of Viet Nam | 596.4 (330.7-1069.2) | 1.3 (0.7-2.4) | 190.8 (117.9-284.5) | 0.2 (0.1-0.3) | -2.11 (-2.61--1.62) |
| Acute hepatitis B | Solomon Islands | 4.9 (1.6-10) | 2.1 (0.6-4.4) | 1.8 (0.7-3.7) | 0.3 (0.1-0.7) | -1.81 (-2.37--1.25) |
| Acute hepatitis B | State of Eritrea | 51.6 (21.4-84.1) | 2.8 (1.1-4.4) | 69.1 (35.2-109.9) | 1.6 (0.8-2.6) | -1.01 (-1.93--0.08) |
| Acute hepatitis B | State of Israel | 3.5 (2.9-4.2) | 0.1 (0.1-0.1) | 1.7 (1.4-2) | 0 (0-0) | -0.09 (-0.14--0.05) |
| Acute hepatitis B | State of Kuwait | 0.4 (0.3-0.4) | 0 (0-0.1) | 0.4 (0.3-0.6) | 0 (0-0) | -0.14 (-0.18--0.09) |
| Acute hepatitis B | State of Libya | 35.2 (13.5-69.7) | 1.6 (0.6-3.3) | 41.9 (28.8-62.6) | 0.7 (0.5-1.1) | -0.91 (-1.45--0.36) |
| Acute hepatitis B | State of Qatar | 1.6 (1.1-2.2) | 1.1 (0.8-1.6) | 3.5 (2.1-5.6) | 0.3 (0.2-0.5) | -1.3 (-1.75--0.86) |
| Acute hepatitis B | Sultanate of Oman | 2.8 (1-6.3) | 0.3 (0.1-0.8) | 3.4 (1.4-5.9) | 0.1 (0.1-0.2) | -1.65 (-2.66--0.63) |
| Acute hepatitis B | Swiss Confederation | 3 (2.5-3.7) | 0 (0-0) | 1.4 (1.1-1.7) | 0 (0-0) | -0.11 (-0.16--0.06) |
| Acute hepatitis B | Syrian Arab Republic | 25.4 (12.1-46.7) | 0.4 (0.2-0.7) | 10.6 (6-17) | 0.1 (0-0.1) | -1.14 (-1.5--0.79) |
| Acute hepatitis B | Taiwan (Province of China) | 1.9 (1.4-3.3) | 0 (0-0) | 1 (0.8-1.3) | 0 (0-0) | -0.06 (-0.08--0.04) |
| Acute hepatitis B | Togolese Republic | 5.2 (2.3-10.2) | 0.2 (0.1-0.4) | 9.9 (4.5-19.4) | 0.2 (0.1-0.3) | -0.21 (-0.44-0.03) |
| Acute hepatitis B | Tokelau | 0 (0-0) | 0.3 (0.2-0.5) | 0 (0-0) | 0.2 (0.1-0.3) | -0.44 (-0.62--0.25) |
| Acute hepatitis B | Turkmenistan | 116.6 (91.3-147.4) | 2.7 (2.2-3.3) | 31.2 (20.9-45.8) | 0.6 (0.4-0.9) | -1.81 (-2.44--1.18) |
| Acute hepatitis B | Tuvalu | 0 (0-0.1) | 0.4 (0.2-0.6) | 0 (0-0) | 0.1 (0.1-0.2) | -0.71 (-0.93--0.48) |
| Acute hepatitis B | Ukraine | 105.7 (91-122.2) | 0.2 (0.2-0.3) | 25.7 (17.1-34.8) | 0.1 (0-0.1) | -0.22 (-0.37--0.08) |
| Acute hepatitis B | Union of the Comoros | 3.9 (1.7-6.1) | 1.4 (0.6-2.1) | 6.3 (3.3-10.1) | 1.1 (0.6-1.7) | -0.71 (-1.4--0.01) |
| Acute hepatitis B | United Arab Emirates | 6.6 (4-10.9) | 1 (0.7-1.7) | 27 (17.6-39.9) | 0.6 (0.4-0.8) | -0.43 (-0.95-0.1) |
| Acute hepatitis B | United Kingdom of Great Britain and Northern Ireland | 0.4 (0.3-0.5) | 0 (0-0) | 4.6 (4.2-5.1) | 0 (0-0) | 0.01 (0-0.02) |
| Acute hepatitis B | United Mexican States | 24.4 (21.3-27.9) | 0 (0-0) | 62.2 (52.6-73.5) | 0.1 (0-0.1) | -0.15 (-0.28--0.03) |
| Acute hepatitis B | United Republic of Tanzania | 414.5 (238.1-579.8) | 2.6 (1.3-3.7) | 313.6 (173.4-460.1) | 0.8 (0.5-1.2) | -1.7 (-2.49--0.91) |
| Acute hepatitis B | United States of America | 57.7 (50.1-63.2) | 0 (0-0) | 32.4 (27.7-36.8) | 0 (0-0) | -0.08 (-0.11--0.05) |
| Acute hepatitis B | United States Virgin Islands | 0 (0-0) | 0 (0-0) | 0 (0-0) | 0 (0-0) | -0.03 (-0.05--0.01) |
| Acute hepatitis C | American Samoa | 0 (0-0) | 0 (0-0) | 0 (0-0) | 0 (0-0) | -0.15 (-0.25--0.04) |
| Acute hepatitis C | Antigua and Barbuda | 0 (0-0) | 0 (0-0) | 0 (0-0) | 0 (0-0) | -0.07 (-0.09--0.04) |
| Acute hepatitis C | Arab Republic of Egypt | 149.4 (17-986.7) | 0.4 (0-2.9) | 18.9 (1.2-118.9) | 0 (0-0.2) | -2.56 (-4.13--0.97) |
| Acute hepatitis C | Argentine Republic | 6.2 (3-11.7) | 0 (0-0) | 3.1 (2.5-3.7) | 0 (0-0) | -0.13 (-0.17--0.09) |
| Acute hepatitis C | Australia | 1.7 (1.5-2) | 0 (0-0) | 0.4 (0.3-0.5) | 0 (0-0) | -0.02 (-0.03--0.01) |
| Acute hepatitis C | Barbados | 0 (0-0) | 0 (0-0) | 0 (0-0) | 0 (0-0) | -0.11 (-0.19--0.02) |
| Acute hepatitis C | Belize | 0 (0-0) | 0 (0-0) | 0 (0-0) | 0 (0-0) | -0.11 (-0.15--0.07) |
| Acute hepatitis C | Bermuda | 0 (0-0) | 0 (0-0) | 0 (0-0) | 0 (0-0) | -0.02 (-0.03--0.01) |
| Acute hepatitis C | Bolivarian Republic of Venezuela | 1.9 (1-3.1) | 0 (0-0) | 2.5 (1.7-3.5) | 0 (0-0) | -0.02 (-0.07-0.03) |
| Acute hepatitis C | Bosnia and Herzegovina | 0.1 (0-0.3) | 0 (0-0) | 0 (0-0.1) | 0 (0-0) | -0.15 (-0.26--0.04) |
| Acute hepatitis C | Brunei Darussalam | 0.7 (0.2-1.2) | 0.5 (0.2-0.9) | 0.1 (0.1-0.2) | 0 (0-0.1) | -1.79 (-2.35--1.23) |
| Acute hepatitis C | Burkina Faso | 31.6 (7.2-73.2) | 0.5 (0.1-1.2) | 18.6 (5.9-46.5) | 0.1 (0-0.3) | -1.31 (-2.29--0.33) |
| Acute hepatitis C | Canada | 3.3 (2.6-4) | 0 (0-0) | 0.3 (0.3-0.4) | 0 (0-0) | -0.05 (-0.07--0.03) |
| Acute hepatitis C | Central African Republic | 16.1 (3.5-55) | 0.9 (0.2-3.3) | 9.9 (2.4-27.1) | 0.3 (0.1-0.8) | -1.7 (-2.69--0.69) |
| Acute hepatitis C | Commonwealth of Dominica | 0 (0-0) | 0 (0-0) | 0 (0-0) | 0 (0-0) | -0.07 (-0.11--0.03) |
| Acute hepatitis C | Commonwealth of the Bahamas | 0 (0-0) | 0 (0-0) | 0 (0-0) | 0 (0-0) | -0.07 (-0.11--0.04) |
| Acute hepatitis C | Cook Islands | 0 (0-0) | 0 (0-0) | 0 (0-0) | 0 (0-0) | -0.04 (-0.05--0.02) |
| Acute hepatitis C | Czech Republic | 0.2 (0.1-0.2) | 0 (0-0) | 0.2 (0.1-0.2) | 0 (0-0) | -0.06 (-0.1--0.03) |
| Acute hepatitis C | Democratic People's Republic of Korea | 26.8 (9.8-67.9) | 0.1 (0.1-0.4) | 4.5 (1.6-8.9) | 0 (0-0) | -1.11 (-1.46--0.76) |
| Acute hepatitis C | Democratic Republic of Sao Tome and Principe | 0.1 (0.1-0.2) | 0.1 (0.1-0.2) | 0 (0-0) | 0 (0-0) | -1.31 (-1.78--0.84) |
| Acute hepatitis C | Democratic Republic of the Congo | 21.8 (5.7-46.3) | 0.1 (0-0.2) | 19.2 (3-37.1) | 0 (0-0.1) | -0.35 (-0.6--0.1) |
| Acute hepatitis C | Democratic Republic of Timor-Leste | 0.7 (0.1-2.5) | 0.1 (0-0.5) | 0.3 (0.1-0.7) | 0 (0-0.1) | -0.61 (-0.81--0.42) |
| Acute hepatitis C | Democratic Socialist Republic of Sri Lanka | 8.3 (3.9-15.4) | 0.1 (0-0.1) | 1.8 (0.9-3.3) | 0 (0-0) | -0.61 (-0.77--0.45) |
| Acute hepatitis C | Dominican Republic | 1.2 (0.8-1.7) | 0 (0-0) | 1.7 (1.2-2.7) | 0 (0-0) | -0.08 (-0.14--0.02) |
| Acute hepatitis C | Eastern Republic of Uruguay | 0.2 (0.1-0.3) | 0 (0-0) | 0.1 (0.1-0.1) | 0 (0-0) | -0.05 (-0.06--0.03) |
| Acute hepatitis C | Federal Democratic Republic of Ethiopia | 412.3 (25.7-1196.8) | 1.6 (0.1-4.9) | 270 (45.1-651.6) | 0.5 (0.1-1.1) | -1.93 (-2.95--0.89) |
| Acute hepatitis C | Federal Democratic Republic of Nepal | 62.1 (0.8-145.1) | 0.4 (0-1.1) | 84.2 (8.1-173.6) | 0.3 (0-0.6) | -1.48 (-2.49--0.46) |
| Acute hepatitis C | Federal Republic of Germany | 10.8 (7-15.3) | 0 (0-0) | 1.6 (1.1-2.1) | 0 (0-0) | -0.07 (-0.09--0.04) |
| Acute hepatitis C | Federal Republic of Nigeria | 1120.9 (282.4-3399.3) | 1.6 (0.4-5) | 143.3 (50.3-329.9) | 0.1 (0-0.2) | -4.49 (-5.68--3.29) |
| Acute hepatitis C | Federal Republic of Somalia | 115.6 (3.9-559.2) | 2.9 (0.1-14.2) | 221.3 (9.7-1042.4) | 2.2 (0.1-9.5) | -0.59 (-2.19-1.04) |
| Acute hepatitis C | Federated States of Micronesia | 0.2 (0.1-0.3) | 0.2 (0.1-0.4) | 0 (0-0.1) | 0 (0-0.1) | -0.65 (-0.85--0.45) |
| Acute hepatitis C | Federative Republic of Brazil | 21.7 (17.6-27.7) | 0 (0-0) | 13.5 (11.9-15.5) | 0 (0-0) | -0.18 (-0.26--0.1) |
| Acute hepatitis C | French Republic | 4.5 (3.5-5.5) | 0 (0-0) | 0.8 (0.6-1) | 0 (0-0) | -0.04 (-0.06--0.02) |
| Acute hepatitis C | Gabonese Republic | 1 (0.2-4.9) | 0.1 (0-0.7) | 0.2 (0-0.4) | 0 (0-0) | -1.26 (-1.67--0.85) |
| Acute hepatitis C | Georgia | 15.6 (11.6-20.1) | 0.3 (0.2-0.4) | 0.2 (0.1-0.3) | 0 (0-0) | -0.41 (-0.52--0.29) |
| Acute hepatitis C | Grand Duchy of Luxembourg | 0 (0-0) | 0 (0-0) | 0 (0-0) | 0 (0-0) | -0.02 (-0.03--0.01) |
| Acute hepatitis C | Greenland | 0 (0-0) | 0 (0-0) | 0 (0-0) | 0 (0-0) | -0.15 (-0.19--0.11) |
| Acute hepatitis C | Grenada | 0 (0-0) | 0 (0-0) | 0 (0-0) | 0 (0-0) | -0.16 (-0.24--0.09) |
| Acute hepatitis C | Guam | 0 (0-0) | 0 (0-0) | 0 (0-0) | 0 (0-0) | -0.02 (-0.08-0.04) |
| Acute hepatitis C | Hashemite Kingdom of Jordan | 2.6 (1.1-6.1) | 0.2 (0.1-0.4) | 1.8 (1.1-3) | 0 (0-0) | -1.09 (-1.4--0.78) |
| Acute hepatitis C | Hellenic Republic | 0.9 (0.6-1.2) | 0 (0-0) | 0.5 (0.4-0.6) | 0 (0-0) | -0.09 (-0.18--0.01) |
| Acute hepatitis C | Hungary | 0.2 (0.1-0.2) | 0 (0-0) | 0 (0-0.1) | 0 (0-0) | -0.04 (-0.07--0.01) |
| Acute hepatitis C | Independent State of Papua New Guinea | 16.2 (1.6-59.3) | 0.5 (0-1.9) | 6.3 (0.7-20.4) | 0.1 (0-0.2) | -1.69 (-2.17--1.2) |
| Acute hepatitis C | Independent State of Samoa | 0 (0-0.1) | 0 (0-0.1) | 0 (0-0) | 0 (0-0) | -0.19 (-0.29--0.08) |
| Acute hepatitis C | Ireland | 0.3 (0.2-0.4) | 0 (0-0) | 0.1 (0-0.1) | 0 (0-0) | -0.02 (-0.03--0.01) |
| Acute hepatitis C | Islamic Republic of Afghanistan | 101.4 (5.9-321.5) | 1.3 (0.1-4.4) | 26.1 (2.6-77.5) | 0.2 (0-0.6) | -3 (-4.94--1.03) |
| Acute hepatitis C | Islamic Republic of Iran | 28.5 (10-51) | 0.1 (0-0.2) | 1.6 (1-3) | 0 (0-0) | -1.18 (-1.71--0.64) |
| Acute hepatitis C | Islamic Republic of Mauritania | 10 (2.1-35.5) | 0.7 (0.2-2.6) | 1.3 (0.6-2.8) | 0 (0-0.1) | -3.17 (-4.32--2.01) |
| Acute hepatitis C | Islamic Republic of Pakistan | 679.8 (29.3-1467.9) | 1 (0-2.2) | 1128.9 (184-2145.7) | 0.8 (0.1-1.4) | -1.06 (-2.17-0.07) |
| Acute hepatitis C | Jamaica | 0.2 (0.1-0.3) | 0 (0-0) | 0.1 (0.1-0.1) | 0 (0-0) | -0.05 (-0.09--0.01) |
| Acute hepatitis C | Japan | 37.9 (33.7-41.4) | 0 (0-0) | 4.8 (4.1-5.4) | 0 (0-0) | -0.21 (-0.32--0.1) |
| Acute hepatitis C | Kingdom of Bahrain | 0.1 (0-0.3) | 0 (0-0.1) | 0 (0-0.1) | 0 (0-0) | -1.58 (-2.34--0.81) |
| Acute hepatitis C | Kingdom of Belgium | 1.3 (1-1.7) | 0 (0-0) | 0.2 (0.2-0.3) | 0 (0-0) | -0.06 (-0.09--0.03) |
| Acute hepatitis C | Kingdom of Bhutan | 1.3 (0-3.3) | 0.3 (0-0.8) | 1 (0.2-2.5) | 0.2 (0-0.4) | -1.92 (-2.71--1.13) |
| Acute hepatitis C | Kingdom of Cambodia | 201.7 (57.1-503.6) | 3.3 (0.9-8.3) | 55.3 (14.6-161.9) | 0.4 (0.1-1.1) | -3.96 (-5.07--2.84) |
| Acute hepatitis C | Kingdom of Denmark | 0.2 (0.1-0.2) | 0 (0-0) | 0 (0-0.1) | 0 (0-0) | -0.05 (-0.08--0.02) |
| Acute hepatitis C | Kingdom of Eswatini | 0.8 (0.2-1.8) | 0.2 (0.1-0.5) | 0.3 (0.2-0.5) | 0.1 (0-0.1) | -1.35 (-2.23--0.47) |
| Acute hepatitis C | Kingdom of Lesotho | 2.4 (0.4-6) | 0.3 (0.1-0.7) | 1.2 (0.5-2.3) | 0.1 (0-0.2) | -0.63 (-1.66-0.42) |
| Acute hepatitis C | Kingdom of Morocco | 16.3 (3-65.8) | 0.1 (0-0.4) | 5.8 (1.5-27.3) | 0 (0-0.1) | -0.94 (-1.48--0.39) |
| Acute hepatitis C | Kingdom of Norway | 0 (0-0.1) | 0 (0-0) | 0 (0-0) | 0 (0-0) | 0 (-0.01-0.01) |
| Acute hepatitis C | Kingdom of Saudi Arabia | 111.7 (35.8-261.4) | 1.6 (0.5-3.8) | 10.7 (5.6-21.6) | 0 (0-0.1) | -2.76 (-3.4--2.12) |
| Acute hepatitis C | Kingdom of Spain | 4 (3.3-4.8) | 0 (0-0) | 0.9 (0.7-1.2) | 0 (0-0) | -0.02 (-0.03--0.01) |
| Acute hepatitis C | Kingdom of Sweden | 0.3 (0.2-0.3) | 0 (0-0) | 0.1 (0.1-0.1) | 0 (0-0) | 0 (0-0.01) |
| Acute hepatitis C | Kingdom of Thailand | 19.1 (6.5-40.9) | 0 (0-0.1) | 8.8 (5.6-13.2) | 0 (0-0) | -0.42 (-0.73--0.12) |
| Acute hepatitis C | Kingdom of the Netherlands | 0.2 (0.2-0.3) | 0 (0-0) | 0 (0-0) | 0 (0-0) | -0.02 (-0.03--0.01) |
| Acute hepatitis C | Kingdom of Tonga | 1 (0.3-2) | 1.5 (0.5-3) | 0.3 (0.2-0.5) | 0.3 (0.2-0.6) | -2.7 (-3.75--1.65) |
| Acute hepatitis C | Kyrgyz Republic | 2 (1-3.9) | 0 (0-0.1) | 0.3 (0.1-0.6) | 0 (0-0) | -1.66 (-2.1--1.21) |
| Acute hepatitis C | Lao People's Democratic Republic | 38.5 (9.2-111.3) | 1.3 (0.3-3.8) | 5.3 (1.8-13.4) | 0.1 (0-0.2) | -2.97 (-3.67--2.26) |
| Acute hepatitis C | Lebanese Republic | 2.2 (0.8-3.9) | 0.1 (0-0.2) | 1.2 (0.8-1.8) | 0 (0-0) | -0.6 (-0.83--0.37) |
| Acute hepatitis C | Malaysia | 4.1 (2-6.8) | 0 (0-0.1) | 2.9 (1.8-4.4) | 0 (0-0) | -0.54 (-1.13-0.05) |
| Acute hepatitis C | Mongolia | 0.4 (0.2-0.8) | 0 (0-0) | 0.1 (0-0.1) | 0 (0-0) | -1.28 (-1.69--0.87) |
| Acute hepatitis C | Montenegro | 0 (0-0) | 0 (0-0) | 0 (0-0) | 0 (0-0) | -0.01 (-0.01-0) |
| Acute hepatitis C | New Zealand | 0 (0-0) | 0 (0-0) | 0 (0-0) | 0 (0-0) | -0.03 (-0.04--0.02) |
| Acute hepatitis C | North Macedonia | 0.1 (0-0.1) | 0 (0-0) | 0 (0-0.1) | 0 (0-0) | -0.07 (-0.11--0.02) |
| Acute hepatitis C | Northern Mariana Islands | 0 (0-0) | 0 (0-0) | 0 (0-0) | 0 (0-0) | -0.22 (-0.36--0.08) |
| Acute hepatitis C | Palestine | 1.6 (0.5-4) | 0.2 (0-0.4) | 0.4 (0.2-0.7) | 0 (0-0) | -0.96 (-1.27--0.65) |
| Acute hepatitis C | People's Democratic Republic of Algeria | 9.3 (3-33.8) | 0.1 (0-0.3) | 2.8 (0.6-13.8) | 0 (0-0) | -0.82 (-1.34--0.28) |
| Acute hepatitis C | People's Republic of Bangladesh | 358.7 (7.2-842.3) | 0.6 (0-1.4) | 242.3 (33.4-485.1) | 0.2 (0-0.3) | -2.05 (-3.06--1.02) |
| Acute hepatitis C | People's Republic of China | 2005 (737.4-3905.8) | 0.2 (0.1-0.4) | 73.2 (42.8-148.6) | 0 (0-0) | -2.13 (-2.58--1.67) |
| Acute hepatitis C | Plurinational State of Bolivia | 1.1 (0.3-1.9) | 0 (0-0) | 0.8 (0.5-1.2) | 0 (0-0) | -0.19 (-0.31--0.08) |
| Acute hepatitis C | Portuguese Republic | 1.5 (1-2.1) | 0 (0-0) | 0.2 (0.2-0.3) | 0 (0-0) | -0.14 (-0.22--0.07) |
| Acute hepatitis C | Principality of Andorra | 0.1 (0.1-0.2) | 0.2 (0.1-0.4) | 0 (0-0.1) | 0 (0-0.1) | -0.36 (-0.62--0.1) |
| Acute hepatitis C | Principality of Monaco | 0 (0-0) | 0 (0-0) | 0 (0-0) | 0 (0-0) | 0 (-0.01-0.01) |
| Acute hepatitis C | Puerto Rico | 1.4 (1-1.8) | 0 (0-0.1) | 0.2 (0.2-0.3) | 0 (0-0) | -0.12 (-0.17--0.07) |
| Acute hepatitis C | Republic of Albania | 0.1 (0-0.2) | 0 (0-0) | 0 (0-0.1) | 0 (0-0) | -0.15 (-0.21--0.09) |
| Acute hepatitis C | Republic of Angola | 131.5 (17.8-526.4) | 2.1 (0.3-8.7) | 19.2 (3.6-87.7) | 0.1 (0-0.5) | -3.85 (-4.97--2.71) |
| Acute hepatitis C | Republic of Armenia | 0.6 (0.3-1.1) | 0 (0-0) | 0.1 (0-0.1) | 0 (0-0) | -0.38 (-0.5--0.27) |
| Acute hepatitis C | Republic of Austria | 0.5 (0.3-0.6) | 0 (0-0) | 0 (0-0.1) | 0 (0-0) | -0.02 (-0.04--0.01) |
| Acute hepatitis C | Republic of Azerbaijan | 0.8 (0.4-1.3) | 0 (0-0) | 0.1 (0.1-0.3) | 0 (0-0) | -0.84 (-1.1--0.58) |
| Acute hepatitis C | Republic of Belarus | 1.2 (0.5-1.7) | 0 (0-0) | 0.8 (0.5-1) | 0 (0-0) | -0.24 (-0.4--0.09) |
| Acute hepatitis C | Republic of Benin | 10.4 (3.2-18.3) | 0.3 (0.1-0.6) | 4.3 (2-7.1) | 0.1 (0-0.1) | -1.65 (-2.33--0.97) |
| Acute hepatitis C | Republic of Botswana | 1 (0.3-2.1) | 0.2 (0-0.3) | 0.3 (0.2-0.5) | 0 (0-0) | -1.32 (-1.84--0.8) |
| Acute hepatitis C | Republic of Bulgaria | 1 (0.6-1.5) | 0 (0-0) | 0.2 (0.1-0.4) | 0 (0-0) | -0.04 (-0.11-0.02) |
| Acute hepatitis C | Republic of Burundi | 32.5 (6.6-89.8) | 1 (0.2-2.6) | 27.7 (5.6-73.3) | 0.4 (0.1-1) | -0.65 (-1.33-0.03) |
| Acute hepatitis C | Republic of Cabo Verde | 0.2 (0.1-0.3) | 0.1 (0-0.1) | 0.1 (0-0.1) | 0 (0-0) | -1.25 (-1.88--0.62) |
| Acute hepatitis C | Republic of Cameroon | 7.1 (3.4-12.7) | 0.1 (0-0.2) | 3.7 (1.2-6.8) | 0 (0-0) | -0.47 (-0.71--0.23) |
| Acute hepatitis C | Republic of Chad | 30.6 (7.6-73.1) | 0.7 (0.2-1.8) | 28.3 (8.4-60.6) | 0.3 (0.1-0.6) | -0.82 (-1.97-0.35) |
| Acute hepatitis C | Republic of Chile | 1.1 (0.8-1.4) | 0 (0-0) | 0.3 (0.3-0.4) | 0 (0-0) | -0.09 (-0.11--0.06) |
| Acute hepatitis C | Republic of Colombia | 3 (2.2-4.1) | 0 (0-0) | 1.1 (0.8-1.4) | 0 (0-0) | -0.07 (-0.11--0.03) |
| Acute hepatitis C | Republic of Costa Rica | 0.4 (0.3-0.5) | 0 (0-0) | 0.3 (0.3-0.4) | 0 (0-0) | -0.04 (-0.07--0.02) |
| Acute hepatitis C | Republic of Côte d'Ivoire | 17.2 (5.7-34.3) | 0.2 (0.1-0.5) | 5.8 (3-9.6) | 0 (0-0.1) | -1.5 (-2.08--0.91) |
| Acute hepatitis C | Republic of Croatia | 0 (0-0) | 0 (0-0) | 0 (0-0) | 0 (0-0) | -0.01 (-0.01-0) |
| Acute hepatitis C | Republic of Cuba | 0.5 (0.2-0.8) | 0 (0-0) | 0.1 (0.1-0.1) | 0 (0-0) | -0.11 (-0.15--0.07) |
| Acute hepatitis C | Republic of Cyprus | 0 (0-0) | 0 (0-0) | 0 (0-0) | 0 (0-0) | -0.06 (-0.08--0.03) |
| Acute hepatitis C | Republic of Djibouti | 0.6 (0.1-1.9) | 0.3 (0-0.8) | 1 (0.3-2.7) | 0.1 (0-0.3) | -0.53 (-1.29-0.23) |
| Acute hepatitis C | Republic of Ecuador | 2.1 (1.5-2.8) | 0 (0-0) | 0.3 (0.2-0.4) | 0 (0-0) | -0.1 (-0.16--0.04) |
| Acute hepatitis C | Republic of El Salvador | 0.9 (0.4-1.3) | 0 (0-0) | 0.2 (0.1-0.4) | 0 (0-0) | -0.08 (-0.12--0.04) |
| Acute hepatitis C | Republic of Equatorial Guinea | 0.1 (0-0.3) | 0 (0-0.1) | 0 (0-0.1) | 0 (0-0) | -0.26 (-0.33--0.18) |
| Acute hepatitis C | Republic of Estonia | 0.2 (0.1-0.2) | 0 (0-0) | 0 (0-0) | 0 (0-0) | -0.27 (-0.35--0.19) |
| Acute hepatitis C | Republic of Fiji | 0.4 (0.2-0.7) | 0.1 (0-0.1) | 0.1 (0.1-0.2) | 0 (0-0) | -0.6 (-0.79--0.42) |
| Acute hepatitis C | Republic of Finland | 0.2 (0.1-0.2) | 0 (0-0) | 0 (0-0.1) | 0 (0-0) | -0.02 (-0.03--0.01) |
| Acute hepatitis C | Republic of Ghana | 84.8 (42.6-140.7) | 0.8 (0.4-1.3) | 9.8 (4.2-18.8) | 0 (0-0.1) | -2.87 (-3.53--2.21) |
| Acute hepatitis C | Republic of Guatemala | 2.4 (1.3-4.5) | 0 (0-0.1) | 0.7 (0.6-0.9) | 0 (0-0) | -0.13 (-0.22--0.04) |
| Acute hepatitis C | Republic of Guinea | 30.6 (6-75.4) | 0.7 (0.1-1.8) | 11.9 (4.3-25.1) | 0.1 (0.1-0.3) | -1.66 (-2.74--0.56) |
| Acute hepatitis C | Republic of Guinea-Bissau | 4.3 (0.6-9.3) | 0.6 (0.1-1.4) | 1.6 (0.5-2.9) | 0.1 (0-0.2) | -1.85 (-2.92--0.77) |
| Acute hepatitis C | Republic of Guyana | 0.2 (0.1-0.2) | 0 (0-0) | 0.1 (0.1-0.1) | 0 (0-0) | -0.22 (-0.33--0.1) |
| Acute hepatitis C | Republic of Haiti | 3 (0.3-6.5) | 0.1 (0-0.2) | 2.8 (0.5-5.8) | 0 (0-0.1) | -0.43 (-0.73--0.14) |
| Acute hepatitis C | Republic of Honduras | 3.7 (1-8.5) | 0.1 (0-0.2) | 2.3 (1-4.3) | 0 (0-0.1) | -0.34 (-0.52--0.15) |
| Acute hepatitis C | Republic of Iceland | 0 (0-0) | 0 (0-0) | 0 (0-0) | 0 (0-0) | -0.02 (-0.03--0.01) |
| Acute hepatitis C | Republic of India | 1816.7 (129.1-3290.9) | 0.3 (0-0.5) | 1976.6 (487-3633) | 0.2 (0-0.3) | -1.15 (-2.29-0.01) |
| Acute hepatitis C | Republic of Indonesia | 958.3 (408.2-2005.4) | 0.7 (0.3-1.6) | 467.8 (232.8-809.6) | 0.2 (0.1-0.3) | -1.75 (-2.55--0.94) |
| Acute hepatitis C | Republic of Iraq | 2.2 (0.6-8.8) | 0 (0-0.1) | 1.3 (0.4-5.8) | 0 (0-0) | -1.09 (-1.63--0.54) |
| Acute hepatitis C | Republic of Italy | 9 (6.9-11) | 0 (0-0) | 2.5 (2-3) | 0 (0-0) | 0.02 (-0.02-0.07) |
| Acute hepatitis C | Republic of Kazakhstan | 15.5 (9.7-21.2) | 0.1 (0.1-0.1) | 0.9 (0.6-1.4) | 0 (0-0) | -1.08 (-1.38--0.77) |
| Acute hepatitis C | Republic of Kenya | 37 (5-73.4) | 0.3 (0-0.6) | 46.5 (18.3-83.5) | 0.2 (0.1-0.3) | -0.62 (-1.4-0.17) |
| Acute hepatitis C | Republic of Kiribati | 0.2 (0-0.4) | 0.3 (0.1-0.7) | 0.1 (0-0.3) | 0.1 (0-0.3) | -1.47 (-2.54--0.38) |
| Acute hepatitis C | Republic of Korea | 7.3 (2.2-13.4) | 0 (0-0) | 0.5 (0.2-1.3) | 0 (0-0) | -0.39 (-0.49--0.28) |
| Acute hepatitis C | Republic of Latvia | 0.4 (0.3-0.6) | 0 (0-0) | 0 (0-0.1) | 0 (0-0) | -0.75 (-1.01--0.49) |
| Acute hepatitis C | Republic of Liberia | 4.8 (1.4-8.1) | 0.3 (0.1-0.5) | 2.3 (0.9-4.5) | 0.1 (0-0.1) | -1.43 (-2.24--0.61) |
| Acute hepatitis C | Republic of Lithuania | 0.6 (0.4-0.7) | 0 (0-0) | 0 (0-0) | 0 (0-0) | -0.24 (-0.33--0.15) |
| Acute hepatitis C | Republic of Madagascar | 13 (1.2-30.8) | 0.2 (0-0.4) | 12.2 (2.2-25.1) | 0.1 (0-0.1) | -1.15 (-1.81--0.48) |
| Acute hepatitis C | Republic of Malawi | 31.5 (2.9-95.3) | 0.6 (0.1-1.7) | 23 (5.9-63.2) | 0.2 (0.1-0.6) | -1.4 (-2.58--0.2) |
| Acute hepatitis C | Republic of Maldives | 0.2 (0-0.4) | 0.1 (0-0.3) | 0.1 (0-0.1) | 0 (0-0) | -1.03 (-1.28--0.78) |
| Acute hepatitis C | Republic of Mali | 7.7 (2.5-16) | 0.1 (0-0.3) | 8.5 (2.4-18.4) | 0.1 (0-0.1) | -0.14 (-0.39-0.12) |
| Acute hepatitis C | Republic of Malta | 0 (0-0) | 0 (0-0) | 0 (0-0) | 0 (0-0) | -0.03 (-0.05--0.01) |
| Acute hepatitis C | Republic of Mauritius | 0.5 (0.3-1) | 0.1 (0-0.1) | 0.1 (0.1-0.1) | 0 (0-0) | -0.36 (-0.44--0.27) |
| Acute hepatitis C | Republic of Moldova | 1.9 (1.5-2.4) | 0 (0-0.1) | 0.2 (0.1-0.3) | 0 (0-0) | -0.55 (-0.77--0.33) |
| Acute hepatitis C | Republic of Mozambique | 52.5 (2.2-197.2) | 0.7 (0-2.6) | 27.8 (4.1-90.2) | 0.2 (0-0.5) | -2.32 (-3.41--1.21) |
| Acute hepatitis C | Republic of Namibia | 0.8 (0.3-1.4) | 0.1 (0-0.2) | 0.4 (0.2-0.6) | 0 (0-0) | -0.94 (-1.51--0.37) |
| Acute hepatitis C | Republic of Nauru | 0 (0-0) | 0.1 (0-0.2) | 0 (0-0) | 0 (0-0) | -0.63 (-0.88--0.37) |
| Acute hepatitis C | Republic of Nicaragua | 1 (0.5-1.6) | 0 (0-0) | 0.3 (0.2-0.5) | 0 (0-0) | -0.24 (-0.33--0.15) |
| Acute hepatitis C | Republic of Niue | 0 (0-0) | 0 (0-0.1) | 0 (0-0) | 0 (0-0) | -0.27 (-0.37--0.17) |
| Acute hepatitis C | Republic of Palau | 0 (0-0) | 0 (0-0.1) | 0 (0-0) | 0 (0-0) | -0.78 (-1.23--0.33) |
| Acute hepatitis C | Republic of Panama | 0.2 (0.1-0.2) | 0 (0-0) | 0.1 (0.1-0.1) | 0 (0-0) | -0.06 (-0.1--0.02) |
| Acute hepatitis C | Republic of Paraguay | 0.1 (0.1-0.2) | 0 (0-0) | 0.1 (0.1-0.2) | 0 (0-0) | -0.1 (-0.18--0.03) |
| Acute hepatitis C | Republic of Peru | 2.6 (1.3-3.7) | 0 (0-0) | 1.5 (1-2.3) | 0 (0-0) | -0.17 (-0.24--0.11) |
| Acute hepatitis C | Republic of Poland | 1.1 (0.8-1.3) | 0 (0-0) | 0.2 (0.1-0.2) | 0 (0-0) | -0.01 (-0.03-0.01) |
| Acute hepatitis C | Republic of Rwanda | 32.8 (5-72) | 0.8 (0.1-1.8) | 14.3 (4.5-29) | 0.2 (0.1-0.4) | -2.55 (-3.5--1.6) |
| Acute hepatitis C | Republic of San Marino | 0 (0-0) | 0.1 (0-0.1) | 0 (0-0) | 0 (0-0) | -0.54 (-0.8--0.28) |
| Acute hepatitis C | Republic of Senegal | 5.4 (0.9-9.5) | 0.1 (0-0.2) | 3.2 (1.2-5.5) | 0 (0-0.1) | -0.66 (-1--0.31) |
| Acute hepatitis C | Republic of Serbia | 0.1 (0.1-0.2) | 0 (0-0) | 0.1 (0-0.1) | 0 (0-0) | -0.1 (-0.15--0.04) |
| Acute hepatitis C | Republic of Seychelles | 0.2 (0.1-0.4) | 0.3 (0.2-0.7) | 0.1 (0-0.2) | 0.1 (0-0.2) | -1.17 (-1.61--0.73) |
| Acute hepatitis C | Republic of Sierra Leone | 13.6 (3.1-32.7) | 0.4 (0.1-1) | 4.2 (1.8-7.6) | 0.1 (0-0.1) | -2.14 (-3.05--1.21) |
| Acute hepatitis C | Republic of Singapore | 0 (0-0.1) | 0 (0-0) | 0 (0-0) | 0 (0-0) | -0.07 (-0.11--0.04) |
| Acute hepatitis C | Republic of Slovenia | 0 (0-0) | 0 (0-0) | 0 (0-0) | 0 (0-0) | -0.04 (-0.06--0.02) |
| Acute hepatitis C | Republic of South Africa | 7.9 (4.3-13.5) | 0 (0-0.1) | 7.2 (4.6-10.6) | 0 (0-0) | -0.35 (-0.66--0.04) |
| Acute hepatitis C | Republic of South Sudan | 14.5 (1.2-47.9) | 0.4 (0-1.5) | 21.1 (2.5-62.3) | 0.4 (0-1.1) | 0.18 (-0.79-1.17) |
| Acute hepatitis C | Republic of Sudan | 63.5 (10.1-188.6) | 0.5 (0.1-1.7) | 7.9 (1.8-24.8) | 0 (0-0.1) | -3.04 (-4.51--1.54) |
| Acute hepatitis C | Republic of Suriname | 0 (0-0.1) | 0 (0-0) | 0 (0-0) | 0 (0-0) | -0.13 (-0.2--0.06) |
| Acute hepatitis C | Republic of Tajikistan | 7.4 (3.2-13.4) | 0.1 (0.1-0.2) | 2.7 (1.1-5.9) | 0 (0-0.1) | -1.38 (-2.09--0.66) |
| Acute hepatitis C | Republic of the Congo | 5.2 (1.1-24.5) | 0.4 (0.1-1.8) | 1.4 (0.4-4.3) | 0 (0-0.1) | -2.35 (-3.24--1.46) |
| Acute hepatitis C | Republic of the Gambia | 0.5 (0.1-0.9) | 0.1 (0-0.1) | 0.3 (0.1-0.6) | 0 (0-0) | -0.43 (-0.61--0.26) |
| Acute hepatitis C | Republic of the Marshall Islands | 0 (0-0) | 0.1 (0-0.1) | 0 (0-0) | 0 (0-0) | -0.5 (-0.71--0.3) |
| Acute hepatitis C | Republic of the Niger | 35.3 (6-74.2) | 0.6 (0.1-1.5) | 21 (5.1-40) | 0.1 (0-0.3) | -1.64 (-2.55--0.71) |
| Acute hepatitis C | Republic of the Philippines | 71.9 (37-146.2) | 0.2 (0.1-0.3) | 9.1 (5.7-13) | 0 (0-0) | -1.72 (-2.09--1.34) |
| Acute hepatitis C | Republic of the Union of Myanmar | 104.3 (25.2-226.9) | 0.4 (0.1-0.8) | 38.3 (17.4-71.8) | 0.1 (0-0.1) | -1.04 (-1.44--0.63) |
| Acute hepatitis C | Republic of Trinidad and Tobago | 0.2 (0.1-0.2) | 0 (0-0) | 0.1 (0-0.1) | 0 (0-0) | -0.12 (-0.19--0.05) |
| Acute hepatitis C | Republic of Tunisia | 2.5 (0.6-9.6) | 0 (0-0.2) | 0.7 (0.2-3.3) | 0 (0-0) | -0.8 (-1.27--0.33) |
| Acute hepatitis C | Republic of Turkey | 40.6 (14-70.4) | 0.1 (0-0.2) | 12.8 (8.1-19.8) | 0 (0-0) | -1.09 (-1.4--0.77) |
| Acute hepatitis C | Republic of Uganda | 74.4 (7.9-281.2) | 0.8 (0.1-3.1) | 35.7 (11.7-80.9) | 0.2 (0.1-0.4) | -2.18 (-3.25--1.1) |
| Acute hepatitis C | Republic of Uzbekistan | 9.3 (4.7-17.1) | 0 (0-0.1) | 1.5 (1.1-2.1) | 0 (0-0) | -2.21 (-2.82--1.6) |
| Acute hepatitis C | Republic of Vanuatu | 0.2 (0-0.7) | 0.2 (0-0.7) | 0.1 (0-0.2) | 0 (0-0.1) | -1.01 (-1.34--0.67) |
| Acute hepatitis C | Republic of Yemen | 35.2 (2.4-126.9) | 0.5 (0-2) | 6.5 (1.5-20.4) | 0 (0-0.1) | -3.72 (-5.19--2.23) |
| Acute hepatitis C | Republic of Zambia | 23 (4.7-63.2) | 0.6 (0.1-1.8) | 10.6 (4.2-20.7) | 0.1 (0-0.2) | -2.33 (-3.46--1.2) |
| Acute hepatitis C | Republic of Zimbabwe | 41.4 (9.7-98.6) | 0.9 (0.2-2.2) | 48.8 (17.4-101) | 0.6 (0.2-1.2) | -0.29 (-1.02-0.44) |
| Acute hepatitis C | Romania | 0.8 (0.5-1.3) | 0 (0-0) | 0.2 (0.1-0.3) | 0 (0-0) | -0.08 (-0.11--0.05) |
| Acute hepatitis C | Russian Federation | 2.8 (2.1-3.5) | 0 (0-0) | 1.2 (1-1.4) | 0 (0-0) | -0.16 (-0.23--0.09) |
| Acute hepatitis C | Saint Kitts and Nevis | 0 (0-0) | 0 (0-0) | 0 (0-0) | 0 (0-0) | -0.23 (-0.31--0.15) |
| Acute hepatitis C | Saint Lucia | 0 (0-0) | 0 (0-0) | 0 (0-0) | 0 (0-0) | -0.18 (-0.24--0.12) |
| Acute hepatitis C | Saint Vincent and the Grenadines | 0 (0-0) | 0 (0-0) | 0 (0-0) | 0 (0-0) | -0.02 (-0.06-0.02) |
| Acute hepatitis C | Slovak Republic | 0.1 (0-0.1) | 0 (0-0) | 0 (0-0.1) | 0 (0-0) | -0.02 (-0.04-0.01) |
| Acute hepatitis C | Socialist Republic of Viet Nam | 198.8 (76.7-373.9) | 0.4 (0.2-0.8) | 31.4 (15.8-62.7) | 0 (0-0.1) | -2.11 (-2.61--1.62) |
| Acute hepatitis C | Solomon Islands | 1 (0.1-4.5) | 0.5 (0-2) | 0.4 (0.1-1.1) | 0.1 (0-0.2) | -1.81 (-2.37--1.25) |
| Acute hepatitis C | State of Eritrea | 11 (0.9-32) | 0.6 (0.1-1.7) | 9.9 (2.4-22) | 0.3 (0.1-0.5) | -1.01 (-1.93--0.08) |
| Acute hepatitis C | State of Israel | 0.4 (0.3-0.5) | 0 (0-0) | 0.1 (0.1-0.1) | 0 (0-0) | -0.09 (-0.14--0.05) |
| Acute hepatitis C | State of Kuwait | 0.4 (0.3-0.4) | 0 (0-0.1) | 0.3 (0.2-0.4) | 0 (0-0) | -0.14 (-0.18--0.09) |
| Acute hepatitis C | State of Libya | 1.4 (0.3-5.6) | 0.1 (0-0.2) | 0.5 (0.1-2.6) | 0 (0-0) | -0.91 (-1.45--0.36) |
| Acute hepatitis C | State of Qatar | 0.3 (0.1-0.5) | 0.3 (0.1-0.4) | 0.4 (0.2-0.7) | 0 (0-0.1) | -1.3 (-1.75--0.86) |
| Acute hepatitis C | Sultanate of Oman | 0.5 (0.1-1.4) | 0.1 (0-0.2) | 0.3 (0.1-0.6) | 0 (0-0) | -1.65 (-2.66--0.63) |
| Acute hepatitis C | Swiss Confederation | 2.9 (2.2-3.6) | 0 (0-0) | 1.8 (1.4-2.3) | 0 (0-0) | -0.11 (-0.16--0.06) |
| Acute hepatitis C | Syrian Arab Republic | 5.5 (1.6-11.9) | 0.1 (0-0.2) | 1.4 (0.8-2.5) | 0 (0-0) | -1.14 (-1.5--0.79) |
| Acute hepatitis C | Taiwan (Province of China) | 3.9 (2-4.7) | 0 (0-0) | 0.5 (0.4-0.6) | 0 (0-0) | -0.06 (-0.08--0.04) |
| Acute hepatitis C | Togolese Republic | 1.8 (0.6-3) | 0.1 (0-0.1) | 1.3 (0.5-2.5) | 0 (0-0) | -0.21 (-0.44-0.03) |
| Acute hepatitis C | Tokelau | 0 (0-0) | 0.1 (0-0.1) | 0 (0-0) | 0 (0-0) | -0.44 (-0.62--0.25) |
| Acute hepatitis C | Turkmenistan | 17.5 (10.9-27.7) | 0.4 (0.3-0.7) | 3 (1.2-5.9) | 0.1 (0-0.1) | -1.81 (-2.44--1.18) |
| Acute hepatitis C | Tuvalu | 0 (0-0) | 0.1 (0-0.3) | 0 (0-0) | 0 (0-0) | -0.71 (-0.93--0.48) |
| Acute hepatitis C | Ukraine | 29.3 (20.5-37.1) | 0 (0-0.1) | 3.9 (2.6-5.7) | 0 (0-0) | -0.22 (-0.37--0.08) |
| Acute hepatitis C | Union of the Comoros | 0.7 (0.1-1.9) | 0.3 (0-0.7) | 0.7 (0.2-1.4) | 0.1 (0-0.3) | -0.71 (-1.4--0.01) |
| Acute hepatitis C | United Arab Emirates | 1.2 (0.4-2.3) | 0.2 (0.1-0.4) | 2.2 (1.2-3.5) | 0.1 (0-0.1) | -0.43 (-0.95-0.1) |
| Acute hepatitis C | United Kingdom of Great Britain and Northern Ireland | 1.6 (1.3-1.8) | 0 (0-0) | 0.3 (0.3-0.4) | 0 (0-0) | 0.01 (0-0.02) |
| Acute hepatitis C | United Mexican States | 44.4 (33.5-54.9) | 0.1 (0-0.1) | 20.1 (17-23.2) | 0 (0-0) | -0.15 (-0.28--0.03) |
| Acute hepatitis C | United Republic of Tanzania | 63.7 (7.5-162.5) | 0.4 (0.1-1.1) | 38.2 (11.5-77.4) | 0.1 (0-0.2) | -1.7 (-2.49--0.91) |
| Acute hepatitis C | United States of America | 51.6 (44.4-57.8) | 0 (0-0) | 6.7 (5.7-7.8) | 0 (0-0) | -0.08 (-0.11--0.05) |
| Acute hepatitis C | United States Virgin Islands | 0 (0-0) | 0 (0-0) | 0 (0-0) | 0 (0-0) | -0.03 (-0.05--0.01) |
| Acute hepatitis E | American Samoa | 0 (0-0) | 0 (0-0) | 0 (0-0) | 0 (0-0) | -0.15 (-0.25--0.04) |
| Acute hepatitis E | Antigua and Barbuda | 0 (0-0) | 0 (0-0) | 0 (0-0) | 0 (0-0) | -0.07 (-0.09--0.04) |
| Acute hepatitis E | Arab Republic of Egypt | 68.5 (16.3-155) | 0.2 (0-0.4) | 32.5 (8-74) | 0 (0-0.1) | -2.56 (-4.13--0.97) |
| Acute hepatitis E | Argentine Republic | 0.7 (0.4-1.3) | 0 (0-0) | 0.4 (0.3-0.6) | 0 (0-0) | -0.13 (-0.17--0.09) |
| Acute hepatitis E | Australia | 0.1 (0-0.1) | 0 (0-0) | 0.2 (0.1-0.4) | 0 (0-0) | -0.02 (-0.03--0.01) |
| Acute hepatitis E | Barbados | 0 (0-0) | 0 (0-0) | 0 (0-0) | 0 (0-0) | -0.11 (-0.19--0.02) |
| Acute hepatitis E | Belize | 0 (0-0) | 0 (0-0) | 0 (0-0) | 0 (0-0) | -0.11 (-0.15--0.07) |
| Acute hepatitis E | Bermuda | 0 (0-0) | 0 (0-0) | 0 (0-0) | 0 (0-0) | -0.02 (-0.03--0.01) |
| Acute hepatitis E | Bolivarian Republic of Venezuela | 0.2 (0.1-0.4) | 0 (0-0) | 0.3 (0.1-0.5) | 0 (0-0) | -0.02 (-0.07-0.03) |
| Acute hepatitis E | Bosnia and Herzegovina | 0 (0-0) | 0 (0-0) | 0 (0-0) | 0 (0-0) | -0.15 (-0.26--0.04) |
| Acute hepatitis E | Brunei Darussalam | 0.1 (0-0.2) | 0 (0-0.1) | 0 (0-0.1) | 0 (0-0) | -1.79 (-2.35--1.23) |
| Acute hepatitis E | Burkina Faso | 13.1 (4.9-30) | 0.2 (0.1-0.5) | 10.3 (2.9-29.8) | 0.1 (0-0.2) | -1.31 (-2.29--0.33) |
| Acute hepatitis E | Canada | 0 (0-0) | 0 (0-0) | 0.1 (0.1-0.1) | 0 (0-0) | -0.05 (-0.07--0.03) |
| Acute hepatitis E | Central African Republic | 3.2 (0.6-8) | 0.1 (0-0.3) | 2.6 (0.6-7.1) | 0.1 (0-0.2) | -1.7 (-2.69--0.69) |
| Acute hepatitis E | Commonwealth of Dominica | 0 (0-0) | 0 (0-0) | 0 (0-0) | 0 (0-0) | -0.07 (-0.11--0.03) |
| Acute hepatitis E | Commonwealth of the Bahamas | 0 (0-0) | 0 (0-0) | 0 (0-0) | 0 (0-0) | -0.07 (-0.11--0.04) |
| Acute hepatitis E | Cook Islands | 0 (0-0) | 0 (0-0) | 0 (0-0) | 0 (0-0) | -0.04 (-0.05--0.02) |
| Acute hepatitis E | Czech Republic | 0.2 (0.1-0.2) | 0 (0-0) | 0.2 (0.1-0.2) | 0 (0-0) | -0.06 (-0.1--0.03) |
| Acute hepatitis E | Democratic People's Republic of Korea | 6.2 (1-16.8) | 0 (0-0.1) | 2.1 (0.6-4) | 0 (0-0) | -1.11 (-1.46--0.76) |
| Acute hepatitis E | Democratic Republic of Sao Tome and Principe | 0 (0-0.1) | 0 (0-0.1) | 0 (0-0) | 0 (0-0) | -1.31 (-1.78--0.84) |
| Acute hepatitis E | Democratic Republic of the Congo | 5.6 (1.1-14.2) | 0 (0-0) | 6.8 (1.5-14.9) | 0 (0-0) | -0.35 (-0.6--0.1) |
| Acute hepatitis E | Democratic Republic of Timor-Leste | 0.2 (0-0.4) | 0 (0-0.1) | 0.1 (0-0.4) | 0 (0-0) | -0.61 (-0.81--0.42) |
| Acute hepatitis E | Democratic Socialist Republic of Sri Lanka | 4.9 (1.8-9.9) | 0 (0-0.1) | 2.2 (0.9-4.6) | 0 (0-0) | -0.61 (-0.77--0.45) |
| Acute hepatitis E | Dominican Republic | 0.4 (0.2-0.7) | 0 (0-0) | 0.6 (0.3-1) | 0 (0-0) | -0.08 (-0.14--0.02) |
| Acute hepatitis E | Eastern Republic of Uruguay | 0 (0-0) | 0 (0-0) | 0 (0-0) | 0 (0-0) | -0.05 (-0.06--0.03) |
| Acute hepatitis E | Federal Democratic Republic of Ethiopia | 117.2 (28.6-309.3) | 0.4 (0.1-1.2) | 194.1 (57.5-506.4) | 0.3 (0.1-0.8) | -1.93 (-2.95--0.89) |
| Acute hepatitis E | Federal Democratic Republic of Nepal | 13.4 (3.4-47.7) | 0.1 (0-0.2) | 21.8 (8.4-50.5) | 0.1 (0-0.2) | -1.48 (-2.49--0.46) |
| Acute hepatitis E | Federal Republic of Germany | 0.5 (0.3-0.7) | 0 (0-0) | 14.1 (10.7-17.9) | 0 (0-0) | -0.07 (-0.09--0.04) |
| Acute hepatitis E | Federal Republic of Nigeria | 379.5 (69.7-975.8) | 0.5 (0.1-1.5) | 98.6 (29.7-229.4) | 0.1 (0-0.1) | -4.49 (-5.68--3.29) |
| Acute hepatitis E | Federal Republic of Somalia | 25.3 (3.3-81.8) | 0.6 (0.1-1.9) | 59.1 (9-207.3) | 0.5 (0.1-1.9) | -0.59 (-2.19-1.04) |
| Acute hepatitis E | Federated States of Micronesia | 0 (0-0.1) | 0.1 (0-0.1) | 0 (0-0) | 0 (0-0) | -0.65 (-0.85--0.45) |
| Acute hepatitis E | Federative Republic of Brazil | 8.4 (7.2-9.8) | 0 (0-0) | 5 (3.7-6.4) | 0 (0-0) | -0.18 (-0.26--0.1) |
| Acute hepatitis E | French Republic | 0.2 (0.2-0.3) | 0 (0-0) | 1.9 (1.2-2.7) | 0 (0-0) | -0.04 (-0.06--0.02) |
| Acute hepatitis E | Gabonese Republic | 0.3 (0.1-0.8) | 0 (0-0.1) | 0.1 (0-0.2) | 0 (0-0) | -1.26 (-1.67--0.85) |
| Acute hepatitis E | Georgia | 0.3 (0.1-0.5) | 0 (0-0) | 0.1 (0-0.1) | 0 (0-0) | -0.41 (-0.52--0.29) |
| Acute hepatitis E | Grand Duchy of Luxembourg | 0 (0-0) | 0 (0-0) | 0 (0-0) | 0 (0-0) | -0.02 (-0.03--0.01) |
| Acute hepatitis E | Greenland | 0 (0-0) | 0 (0-0) | 0 (0-0) | 0 (0-0) | -0.15 (-0.19--0.11) |
| Acute hepatitis E | Grenada | 0 (0-0) | 0 (0-0) | 0 (0-0) | 0 (0-0) | -0.16 (-0.24--0.09) |
| Acute hepatitis E | Guam | 0 (0-0) | 0 (0-0) | 0 (0-0) | 0 (0-0) | -0.02 (-0.08-0.04) |
| Acute hepatitis E | Hashemite Kingdom of Jordan | 0.9 (0.4-1.6) | 0 (0-0.1) | 1 (0.5-1.8) | 0 (0-0) | -1.09 (-1.4--0.78) |
| Acute hepatitis E | Hellenic Republic | 0.1 (0.1-0.1) | 0 (0-0) | 1.5 (1-2.1) | 0 (0-0) | -0.09 (-0.18--0.01) |
| Acute hepatitis E | Hungary | 0.4 (0.3-0.5) | 0 (0-0) | 0.3 (0.1-0.4) | 0 (0-0) | -0.04 (-0.07--0.01) |
| Acute hepatitis E | Independent State of Papua New Guinea | 5 (1.2-15.6) | 0.2 (0-0.6) | 3.9 (0.9-11.4) | 0 (0-0.1) | -1.69 (-2.17--1.2) |
| Acute hepatitis E | Independent State of Samoa | 0 (0-0) | 0 (0-0) | 0 (0-0) | 0 (0-0) | -0.19 (-0.29--0.08) |
| Acute hepatitis E | Ireland | 0 (0-0) | 0 (0-0) | 0.1 (0-0.1) | 0 (0-0) | -0.02 (-0.03--0.01) |
| Acute hepatitis E | Islamic Republic of Afghanistan | 38.6 (10.3-102.4) | 0.5 (0.1-1.4) | 27.9 (8.9-64.8) | 0.1 (0-0.4) | -3 (-4.94--1.03) |
| Acute hepatitis E | Islamic Republic of Iran | 19.9 (11-40.2) | 0 (0-0.1) | 4.2 (2.3-7.7) | 0 (0-0) | -1.18 (-1.71--0.64) |
| Acute hepatitis E | Islamic Republic of Mauritania | 3.5 (0.4-14.9) | 0.3 (0-1.1) | 0.8 (0.3-1.7) | 0 (0-0.1) | -3.17 (-4.32--2.01) |
| Acute hepatitis E | Islamic Republic of Pakistan | 115.2 (32.4-352.2) | 0.1 (0-0.3) | 270.5 (84.4-626) | 0.1 (0-0.3) | -1.06 (-2.17-0.07) |
| Acute hepatitis E | Jamaica | 0 (0-0) | 0 (0-0) | 0 (0-0.1) | 0 (0-0) | -0.05 (-0.09--0.01) |
| Acute hepatitis E | Japan | 2.9 (2.5-3.2) | 0 (0-0) | 2.8 (1.9-3.4) | 0 (0-0) | -0.21 (-0.32--0.1) |
| Acute hepatitis E | Kingdom of Bahrain | 0.3 (0.1-0.6) | 0.1 (0-0.2) | 0.2 (0.1-0.6) | 0 (0-0.1) | -1.58 (-2.34--0.81) |
| Acute hepatitis E | Kingdom of Belgium | 0 (0-0.1) | 0 (0-0) | 0.5 (0.4-0.7) | 0 (0-0) | -0.06 (-0.09--0.03) |
| Acute hepatitis E | Kingdom of Bhutan | 0.3 (0-1) | 0.1 (0-0.1) | 0.4 (0.1-0.9) | 0.1 (0-0.1) | -1.92 (-2.71--1.13) |
| Acute hepatitis E | Kingdom of Cambodia | 19.8 (6.7-38.3) | 0.3 (0.1-0.7) | 16.6 (7.1-32.5) | 0.1 (0.1-0.2) | -3.96 (-5.07--2.84) |
| Acute hepatitis E | Kingdom of Denmark | 0 (0-0) | 0 (0-0) | 0.3 (0.2-0.4) | 0 (0-0) | -0.05 (-0.08--0.02) |
| Acute hepatitis E | Kingdom of Eswatini | 0.4 (0.1-1) | 0.1 (0-0.3) | 0.3 (0.1-0.5) | 0 (0-0.1) | -1.35 (-2.23--0.47) |
| Acute hepatitis E | Kingdom of Lesotho | 1.1 (0.3-2.9) | 0.1 (0-0.3) | 0.8 (0.4-1.4) | 0.1 (0-0.1) | -0.63 (-1.66-0.42) |
| Acute hepatitis E | Kingdom of Morocco | 6.7 (3.3-15.7) | 0 (0-0.1) | 4.6 (2.1-12) | 0 (0-0) | -0.94 (-1.48--0.39) |
| Acute hepatitis E | Kingdom of Norway | 0 (0-0) | 0 (0-0) | 0.1 (0-0.1) | 0 (0-0) | 0 (-0.01-0.01) |
| Acute hepatitis E | Kingdom of Saudi Arabia | 6.2 (3-12.6) | 0.1 (0-0.1) | 1.9 (0.9-5.2) | 0 (0-0) | -2.76 (-3.4--2.12) |
| Acute hepatitis E | Kingdom of Spain | 0.2 (0.2-0.3) | 0 (0-0) | 2.6 (1.7-3.5) | 0 (0-0) | -0.02 (-0.03--0.01) |
| Acute hepatitis E | Kingdom of Sweden | 0 (0-0) | 0 (0-0) | 0 (0-0.1) | 0 (0-0) | 0 (0-0.01) |
| Acute hepatitis E | Kingdom of Thailand | 6.1 (2.8-11.8) | 0 (0-0) | 9.3 (5-17.4) | 0 (0-0) | -0.42 (-0.73--0.12) |
| Acute hepatitis E | Kingdom of the Netherlands | 0.2 (0.1-0.2) | 0 (0-0) | 1.1 (0.9-1.4) | 0 (0-0) | -0.02 (-0.03--0.01) |
| Acute hepatitis E | Kingdom of Tonga | 0.4 (0.1-0.8) | 0.5 (0.2-1.1) | 0.2 (0.1-0.5) | 0.2 (0.1-0.5) | -2.7 (-3.75--1.65) |
| Acute hepatitis E | Kyrgyz Republic | 1.3 (0.9-2.2) | 0 (0-0) | 0.2 (0.1-0.3) | 0 (0-0) | -1.66 (-2.1--1.21) |
| Acute hepatitis E | Lao People's Democratic Republic | 4.5 (1.2-9.5) | 0.1 (0-0.3) | 2 (0.8-3.6) | 0 (0-0.1) | -2.97 (-3.67--2.26) |
| Acute hepatitis E | Lebanese Republic | 0.6 (0.3-1) | 0 (0-0) | 0.9 (0.5-1.6) | 0 (0-0) | -0.6 (-0.83--0.37) |
| Acute hepatitis E | Malaysia | 1 (0.5-1.6) | 0 (0-0) | 1.2 (0.7-2.1) | 0 (0-0) | -0.54 (-1.13-0.05) |
| Acute hepatitis E | Mongolia | 0.1 (0-0.2) | 0 (0-0) | 0 (0-0.1) | 0 (0-0) | -1.28 (-1.69--0.87) |
| Acute hepatitis E | Montenegro | 0 (0-0) | 0 (0-0) | 0 (0-0) | 0 (0-0) | -0.01 (-0.01-0) |
| Acute hepatitis E | New Zealand | 0 (0-0) | 0 (0-0) | 0 (0-0) | 0 (0-0) | -0.03 (-0.04--0.02) |
| Acute hepatitis E | North Macedonia | 0.2 (0.1-0.5) | 0 (0-0) | 0 (0-0.1) | 0 (0-0) | -0.07 (-0.11--0.02) |
| Acute hepatitis E | Northern Mariana Islands | 0 (0-0) | 0 (0-0) | 0 (0-0) | 0 (0-0) | -0.22 (-0.36--0.08) |
| Acute hepatitis E | Palestine | 0.5 (0.2-0.9) | 0 (0-0.1) | 0.3 (0.2-0.5) | 0 (0-0) | -0.96 (-1.27--0.65) |
| Acute hepatitis E | People's Democratic Republic of Algeria | 7.5 (3.5-15.3) | 0 (0-0.1) | 4.3 (2-11) | 0 (0-0) | -0.82 (-1.34--0.28) |
| Acute hepatitis E | People's Republic of Bangladesh | 92.6 (33.7-218.8) | 0.1 (0.1-0.3) | 135 (50-344.9) | 0.1 (0-0.3) | -2.05 (-3.06--1.02) |
| Acute hepatitis E | People's Republic of China | 800.3 (248.2-1529.8) | 0.1 (0-0.2) | 144.3 (80.2-290.7) | 0 (0-0) | -2.13 (-2.58--1.67) |
| Acute hepatitis E | Plurinational State of Bolivia | 0.2 (0-0.3) | 0 (0-0) | 0.2 (0.1-0.3) | 0 (0-0) | -0.19 (-0.31--0.08) |
| Acute hepatitis E | Portuguese Republic | 0 (0-0) | 0 (0-0) | 0.5 (0.3-0.7) | 0 (0-0) | -0.14 (-0.22--0.07) |
| Acute hepatitis E | Principality of Andorra | 0 (0-0.1) | 0 (0-0.1) | 0 (0-0.1) | 0 (0-0.1) | -0.36 (-0.62--0.1) |
| Acute hepatitis E | Principality of Monaco | 0 (0-0) | 0 (0-0) | 0 (0-0) | 0 (0-0) | 0 (-0.01-0.01) |
| Acute hepatitis E | Puerto Rico | 0 (0-0.1) | 0 (0-0) | 0.1 (0-0.1) | 0 (0-0) | -0.12 (-0.17--0.07) |
| Acute hepatitis E | Republic of Albania | 1.2 (0.2-2.3) | 0 (0-0.1) | 0.2 (0.1-0.4) | 0 (0-0) | -0.15 (-0.21--0.09) |
| Acute hepatitis E | Republic of Angola | 16.3 (2.2-48.6) | 0.2 (0-0.7) | 7.2 (1.6-23.8) | 0 (0-0.1) | -3.85 (-4.97--2.71) |
| Acute hepatitis E | Republic of Armenia | 0.2 (0.1-0.3) | 0 (0-0) | 0 (0-0) | 0 (0-0) | -0.38 (-0.5--0.27) |
| Acute hepatitis E | Republic of Austria | 0 (0-0.1) | 0 (0-0) | 0.6 (0.4-0.7) | 0 (0-0) | -0.02 (-0.04--0.01) |
| Acute hepatitis E | Republic of Azerbaijan | 4.6 (2-9.1) | 0.1 (0-0.1) | 0.9 (0.3-1.9) | 0 (0-0) | -0.84 (-1.1--0.58) |
| Acute hepatitis E | Republic of Belarus | 0.9 (0.7-1.2) | 0 (0-0) | 0.9 (0.6-1.2) | 0 (0-0) | -0.24 (-0.4--0.09) |
| Acute hepatitis E | Republic of Benin | 4.3 (1.8-7.2) | 0.1 (0.1-0.2) | 2.5 (1-4.5) | 0 (0-0.1) | -1.65 (-2.33--0.97) |
| Acute hepatitis E | Republic of Botswana | 0.5 (0.2-1) | 0.1 (0-0.2) | 0.3 (0.2-0.6) | 0 (0-0) | -1.32 (-1.84--0.8) |
| Acute hepatitis E | Republic of Bulgaria | 0.4 (0.3-0.6) | 0 (0-0) | 0.4 (0.2-0.5) | 0 (0-0) | -0.04 (-0.11-0.02) |
| Acute hepatitis E | Republic of Burundi | 2.8 (0.7-8.3) | 0.1 (0-0.2) | 5.4 (1.1-14.7) | 0.1 (0-0.2) | -0.65 (-1.33-0.03) |
| Acute hepatitis E | Republic of Cabo Verde | 0.1 (0-0.1) | 0 (0-0) | 0 (0-0.1) | 0 (0-0) | -1.25 (-1.88--0.62) |
| Acute hepatitis E | Republic of Cameroon | 3.7 (1.5-8.7) | 0.1 (0-0.1) | 3.5 (1.2-8.3) | 0 (0-0) | -0.47 (-0.71--0.23) |
| Acute hepatitis E | Republic of Chad | 11.5 (3.6-26.9) | 0.3 (0.1-0.7) | 12.2 (4.1-29.1) | 0.1 (0-0.3) | -0.82 (-1.97-0.35) |
| Acute hepatitis E | Republic of Chile | 0.1 (0.1-0.2) | 0 (0-0) | 0.1 (0.1-0.1) | 0 (0-0) | -0.09 (-0.11--0.06) |
| Acute hepatitis E | Republic of Colombia | 0.9 (0.6-1.2) | 0 (0-0) | 0.3 (0.2-0.4) | 0 (0-0) | -0.07 (-0.11--0.03) |
| Acute hepatitis E | Republic of Costa Rica | 0.1 (0.1-0.1) | 0 (0-0) | 0.1 (0.1-0.1) | 0 (0-0) | -0.04 (-0.07--0.02) |
| Acute hepatitis E | Republic of Côte d'Ivoire | 9 (3.4-17.1) | 0.1 (0.1-0.3) | 4.4 (1.9-9.3) | 0 (0-0.1) | -1.5 (-2.08--0.91) |
| Acute hepatitis E | Republic of Croatia | 0 (0-0) | 0 (0-0) | 0 (0-0) | 0 (0-0) | -0.01 (-0.01-0) |
| Acute hepatitis E | Republic of Cuba | 0 (0-0.1) | 0 (0-0) | 0.1 (0-0.1) | 0 (0-0) | -0.11 (-0.15--0.07) |
| Acute hepatitis E | Republic of Cyprus | 0 (0-0) | 0 (0-0) | 0 (0-0) | 0 (0-0) | -0.06 (-0.08--0.03) |
| Acute hepatitis E | Republic of Djibouti | 0.3 (0.1-0.8) | 0.1 (0-0.3) | 0.9 (0.2-2.1) | 0.1 (0-0.3) | -0.53 (-1.29-0.23) |
| Acute hepatitis E | Republic of Ecuador | 0.2 (0.1-0.3) | 0 (0-0) | 0.1 (0.1-0.2) | 0 (0-0) | -0.1 (-0.16--0.04) |
| Acute hepatitis E | Republic of El Salvador | 0.4 (0.2-0.8) | 0 (0-0) | 0.1 (0.1-0.2) | 0 (0-0) | -0.08 (-0.12--0.04) |
| Acute hepatitis E | Republic of Equatorial Guinea | 0 (0-0.1) | 0 (0-0) | 0 (0-0.1) | 0 (0-0) | -0.26 (-0.33--0.18) |
| Acute hepatitis E | Republic of Estonia | 0 (0-0.1) | 0 (0-0) | 0 (0-0) | 0 (0-0) | -0.27 (-0.35--0.19) |
| Acute hepatitis E | Republic of Fiji | 0.2 (0.1-0.4) | 0 (0-0.1) | 0.1 (0.1-0.2) | 0 (0-0) | -0.6 (-0.79--0.42) |
| Acute hepatitis E | Republic of Finland | 0 (0-0) | 0 (0-0) | 0 (0-0.1) | 0 (0-0) | -0.02 (-0.03--0.01) |
| Acute hepatitis E | Republic of Ghana | 37.4 (19.9-70.6) | 0.4 (0.2-0.8) | 17.6 (8.5-32.1) | 0.1 (0-0.1) | -2.87 (-3.53--2.21) |
| Acute hepatitis E | Republic of Guatemala | 0.3 (0.1-0.6) | 0 (0-0) | 0.3 (0.2-0.6) | 0 (0-0) | -0.13 (-0.22--0.04) |
| Acute hepatitis E | Republic of Guinea | 14.3 (4-34.6) | 0.3 (0.1-0.8) | 6.5 (2.3-15.4) | 0.1 (0-0.2) | -1.66 (-2.74--0.56) |
| Acute hepatitis E | Republic of Guinea-Bissau | 1.7 (0.5-3.8) | 0.3 (0.1-0.7) | 0.8 (0.3-1.6) | 0.1 (0-0.1) | -1.85 (-2.92--0.77) |
| Acute hepatitis E | Republic of Guyana | 0.1 (0-0.1) | 0 (0-0) | 0.1 (0.1-0.1) | 0 (0-0) | -0.22 (-0.33--0.1) |
| Acute hepatitis E | Republic of Haiti | 0.5 (0.1-1.1) | 0 (0-0) | 0.8 (0.2-1.4) | 0 (0-0) | -0.43 (-0.73--0.14) |
| Acute hepatitis E | Republic of Honduras | 1 (0.1-2.5) | 0 (0-0) | 0.5 (0.2-1.1) | 0 (0-0) | -0.34 (-0.52--0.15) |
| Acute hepatitis E | Republic of Iceland | 0 (0-0) | 0 (0-0) | 0 (0-0) | 0 (0-0) | -0.02 (-0.03--0.01) |
| Acute hepatitis E | Republic of India | 721.4 (309-1554.4) | 0.1 (0-0.2) | 1486.3 (637.4-2963.1) | 0.1 (0.1-0.2) | -1.15 (-2.29-0.01) |
| Acute hepatitis E | Republic of Indonesia | 328.6 (130.1-651.8) | 0.3 (0.1-0.5) | 432.9 (203.7-797.1) | 0.2 (0.1-0.3) | -1.75 (-2.55--0.94) |
| Acute hepatitis E | Republic of Iraq | 5.7 (2.7-9.2) | 0 (0-0.1) | 7.1 (3.9-12.6) | 0 (0-0) | -1.09 (-1.63--0.54) |
| Acute hepatitis E | Republic of Italy | 0.3 (0.3-0.4) | 0 (0-0) | 0.4 (0.3-0.6) | 0 (0-0) | 0.02 (-0.02-0.07) |
| Acute hepatitis E | Republic of Kazakhstan | 3.4 (2.3-4.9) | 0 (0-0) | 0.6 (0.4-0.8) | 0 (0-0) | -1.08 (-1.38--0.77) |
| Acute hepatitis E | Republic of Kenya | 24.1 (7.3-53.4) | 0.2 (0-0.5) | 37.9 (10.9-88.2) | 0.1 (0-0.3) | -0.62 (-1.4-0.17) |
| Acute hepatitis E | Republic of Kiribati | 0.1 (0-0.1) | 0.1 (0-0.2) | 0.1 (0-0.2) | 0.1 (0-0.1) | -1.47 (-2.54--0.38) |
| Acute hepatitis E | Republic of Korea | 0.6 (0.2-1.2) | 0 (0-0) | 0.2 (0.1-0.4) | 0 (0-0) | -0.39 (-0.49--0.28) |
| Acute hepatitis E | Republic of Latvia | 0.3 (0.2-0.4) | 0 (0-0) | 0.2 (0.1-0.3) | 0 (0-0) | -0.75 (-1.01--0.49) |
| Acute hepatitis E | Republic of Liberia | 2.9 (1.1-5) | 0.2 (0.1-0.3) | 1.6 (0.6-3.5) | 0.1 (0-0.1) | -1.43 (-2.24--0.61) |
| Acute hepatitis E | Republic of Lithuania | 0.3 (0.2-0.4) | 0 (0-0) | 0.1 (0-0.1) | 0 (0-0) | -0.24 (-0.33--0.15) |
| Acute hepatitis E | Republic of Madagascar | 7.6 (2.6-16.2) | 0.1 (0-0.2) | 9.2 (3.2-21.3) | 0.1 (0-0.1) | -1.15 (-1.81--0.48) |
| Acute hepatitis E | Republic of Malawi | 5.4 (1.8-13.8) | 0.1 (0-0.2) | 7.2 (2.5-17.4) | 0.1 (0-0.1) | -1.4 (-2.58--0.2) |
| Acute hepatitis E | Republic of Maldives | 0.1 (0-0.1) | 0 (0-0.1) | 0 (0-0.1) | 0 (0-0) | -1.03 (-1.28--0.78) |
| Acute hepatitis E | Republic of Mali | 2.6 (1.1-7.7) | 0.1 (0-0.2) | 4.1 (1.4-10.5) | 0 (0-0.1) | -0.14 (-0.39-0.12) |
| Acute hepatitis E | Republic of Malta | 0 (0-0) | 0 (0-0) | 0 (0-0) | 0 (0-0) | -0.03 (-0.05--0.01) |
| Acute hepatitis E | Republic of Mauritius | 0.1 (0.1-0.2) | 0 (0-0) | 0 (0-0) | 0 (0-0) | -0.36 (-0.44--0.27) |
| Acute hepatitis E | Republic of Moldova | 0.2 (0.1-0.3) | 0 (0-0) | 0.1 (0-0.1) | 0 (0-0) | -0.55 (-0.77--0.33) |
| Acute hepatitis E | Republic of Mozambique | 13.2 (2.9-39.5) | 0.1 (0-0.5) | 9.4 (3.1-24) | 0 (0-0.1) | -2.32 (-3.41--1.21) |
| Acute hepatitis E | Republic of Namibia | 0.4 (0.2-0.9) | 0.1 (0-0.1) | 0.4 (0.2-0.7) | 0 (0-0.1) | -0.94 (-1.51--0.37) |
| Acute hepatitis E | Republic of Nauru | 0 (0-0) | 0 (0-0.1) | 0 (0-0) | 0 (0-0) | -0.63 (-0.88--0.37) |
| Acute hepatitis E | Republic of Nicaragua | 0.4 (0.1-0.9) | 0 (0-0) | 0.2 (0.1-0.3) | 0 (0-0) | -0.24 (-0.33--0.15) |
| Acute hepatitis E | Republic of Niue | 0 (0-0) | 0 (0-0) | 0 (0-0) | 0 (0-0) | -0.27 (-0.37--0.17) |
| Acute hepatitis E | Republic of Palau | 0 (0-0) | 0.1 (0-0.2) | 0 (0-0) | 0 (0-0.1) | -0.78 (-1.23--0.33) |
| Acute hepatitis E | Republic of Panama | 0 (0-0) | 0 (0-0) | 0 (0-0.1) | 0 (0-0) | -0.06 (-0.1--0.02) |
| Acute hepatitis E | Republic of Paraguay | 0 (0-0.1) | 0 (0-0) | 0 (0-0.1) | 0 (0-0) | -0.1 (-0.18--0.03) |
| Acute hepatitis E | Republic of Peru | 0.3 (0.1-0.5) | 0 (0-0) | 0.3 (0.2-0.7) | 0 (0-0) | -0.17 (-0.24--0.11) |
| Acute hepatitis E | Republic of Poland | 0.6 (0.5-0.7) | 0 (0-0) | 0 (0-0) | 0 (0-0) | -0.01 (-0.03-0.01) |
| Acute hepatitis E | Republic of Rwanda | 4.6 (1.7-8.1) | 0.1 (0-0.2) | 4.7 (1.4-10.3) | 0.1 (0-0.1) | -2.55 (-3.5--1.6) |
| Acute hepatitis E | Republic of San Marino | 0 (0-0) | 0.1 (0-0.1) | 0 (0-0) | 0 (0-0.1) | -0.54 (-0.8--0.28) |
| Acute hepatitis E | Republic of Senegal | 2.1 (0.9-3.8) | 0 (0-0.1) | 1.6 (0.6-3.3) | 0 (0-0) | -0.66 (-1--0.31) |
| Acute hepatitis E | Republic of Serbia | 0 (0-0.1) | 0 (0-0) | 0 (0-0) | 0 (0-0) | -0.1 (-0.15--0.04) |
| Acute hepatitis E | Republic of Seychelles | 0.1 (0-0.2) | 0.2 (0.1-0.3) | 0.1 (0-0.2) | 0.1 (0-0.1) | -1.17 (-1.61--0.73) |
| Acute hepatitis E | Republic of Sierra Leone | 8.1 (2.3-18.5) | 0.3 (0.1-0.7) | 2.8 (1.2-5.8) | 0.1 (0-0.1) | -2.14 (-3.05--1.21) |
| Acute hepatitis E | Republic of Singapore | 0 (0-0) | 0 (0-0) | 0 (0-0) | 0 (0-0) | -0.07 (-0.11--0.04) |
| Acute hepatitis E | Republic of Slovenia | 0 (0-0) | 0 (0-0) | 0 (0-0) | 0 (0-0) | -0.04 (-0.06--0.02) |
| Acute hepatitis E | Republic of South Africa | 3.8 (1.2-6.3) | 0 (0-0) | 5.9 (2.7-11.2) | 0 (0-0) | -0.35 (-0.66--0.04) |
| Acute hepatitis E | Republic of South Sudan | 7.2 (1.6-17.9) | 0.2 (0-0.5) | 11.3 (3.1-26.1) | 0.2 (0.1-0.5) | 0.18 (-0.79-1.17) |
| Acute hepatitis E | Republic of Sudan | 49.4 (13.2-152.4) | 0.3 (0.1-1.1) | 22.6 (7.4-70.4) | 0.1 (0-0.2) | -3.04 (-4.51--1.54) |
| Acute hepatitis E | Republic of Suriname | 0 (0-0) | 0 (0-0) | 0 (0-0) | 0 (0-0) | -0.13 (-0.2--0.06) |
| Acute hepatitis E | Republic of Tajikistan | 0.1 (0-0.2) | 0 (0-0) | 0 (0-0.1) | 0 (0-0) | -1.38 (-2.09--0.66) |
| Acute hepatitis E | Republic of the Congo | 1.4 (0.3-5.2) | 0.1 (0-0.2) | 0.7 (0.2-2) | 0 (0-0) | -2.35 (-3.24--1.46) |
| Acute hepatitis E | Republic of the Gambia | 0.3 (0.1-0.7) | 0.1 (0-0.1) | 0.3 (0.1-0.7) | 0 (0-0) | -0.43 (-0.61--0.26) |
| Acute hepatitis E | Republic of the Marshall Islands | 0 (0-0) | 0 (0-0) | 0 (0-0) | 0 (0-0) | -0.5 (-0.71--0.3) |
| Acute hepatitis E | Republic of the Niger | 13.8 (4-27.9) | 0.3 (0.1-0.6) | 9.1 (3.4-20.9) | 0.1 (0-0.2) | -1.64 (-2.55--0.71) |
| Acute hepatitis E | Republic of the Philippines | 37 (18.1-62.7) | 0.1 (0-0.1) | 8.1 (4.3-17.2) | 0 (0-0) | -1.72 (-2.09--1.34) |
| Acute hepatitis E | Republic of the Union of Myanmar | 16.6 (6.3-34.3) | 0.1 (0-0.1) | 16.1 (6.9-33.1) | 0 (0-0.1) | -1.04 (-1.44--0.63) |
| Acute hepatitis E | Republic of Trinidad and Tobago | 0 (0-0) | 0 (0-0) | 0 (0-0) | 0 (0-0) | -0.12 (-0.19--0.05) |
| Acute hepatitis E | Republic of Tunisia | 2.3 (1-5.3) | 0 (0-0.1) | 1.2 (0.5-3.4) | 0 (0-0) | -0.8 (-1.27--0.33) |
| Acute hepatitis E | Republic of Turkey | 18.9 (6.4-41) | 0 (0-0.1) | 6 (3.1-10.4) | 0 (0-0) | -1.09 (-1.4--0.77) |
| Acute hepatitis E | Republic of Uganda | 25.3 (4.9-69.3) | 0.2 (0.1-0.6) | 30.1 (9.5-72.6) | 0.1 (0-0.3) | -2.18 (-3.25--1.1) |
| Acute hepatitis E | Republic of Uzbekistan | 0.7 (0.5-1.1) | 0 (0-0) | 0.2 (0.1-0.3) | 0 (0-0) | -2.21 (-2.82--1.6) |
| Acute hepatitis E | Republic of Vanuatu | 0.1 (0-0.1) | 0.1 (0-0.1) | 0 (0-0.1) | 0 (0-0) | -1.01 (-1.34--0.67) |
| Acute hepatitis E | Republic of Yemen | 33 (6.1-102.6) | 0.3 (0.1-1.4) | 12.3 (3.6-34.9) | 0.1 (0-0.2) | -3.72 (-5.19--2.23) |
| Acute hepatitis E | Republic of Zambia | 5.6 (1.7-12.1) | 0.1 (0-0.3) | 5.2 (2.2-10.7) | 0 (0-0.1) | -2.33 (-3.46--1.2) |
| Acute hepatitis E | Republic of Zimbabwe | 48.4 (12.1-99.4) | 1.1 (0.3-2.2) | 64.9 (22.8-111) | 0.8 (0.3-1.4) | -0.29 (-1.02-0.44) |
| Acute hepatitis E | Romania | 0.1 (0-0.1) | 0 (0-0) | 0.1 (0-0.1) | 0 (0-0) | -0.08 (-0.11--0.05) |
| Acute hepatitis E | Russian Federation | 0.4 (0.2-0.5) | 0 (0-0) | 0.9 (0.6-1.1) | 0 (0-0) | -0.16 (-0.23--0.09) |
| Acute hepatitis E | Saint Kitts and Nevis | 0 (0-0) | 0 (0-0) | 0 (0-0) | 0 (0-0) | -0.23 (-0.31--0.15) |
| Acute hepatitis E | Saint Lucia | 0 (0-0) | 0 (0-0) | 0 (0-0) | 0 (0-0) | -0.18 (-0.24--0.12) |
| Acute hepatitis E | Saint Vincent and the Grenadines | 0 (0-0) | 0 (0-0) | 0 (0-0) | 0 (0-0) | -0.02 (-0.06-0.02) |
| Acute hepatitis E | Slovak Republic | 0 (0-0.1) | 0 (0-0) | 0 (0-0) | 0 (0-0) | -0.02 (-0.04-0.01) |
| Acute hepatitis E | Socialist Republic of Viet Nam | 43.9 (16.7-81.7) | 0.1 (0-0.2) | 22.5 (11.2-52.2) | 0 (0-0) | -2.11 (-2.61--1.62) |
| Acute hepatitis E | Solomon Islands | 0.3 (0-0.7) | 0.1 (0-0.3) | 0.1 (0-0.3) | 0 (0-0.1) | -1.81 (-2.37--1.25) |
| Acute hepatitis E | State of Eritrea | 3.3 (0.7-8) | 0.2 (0-0.4) | 5.7 (1.5-13) | 0.1 (0-0.3) | -1.01 (-1.93--0.08) |
| Acute hepatitis E | State of Israel | 0 (0-0) | 0 (0-0) | 0.4 (0.3-0.5) | 0 (0-0) | -0.09 (-0.14--0.05) |
| Acute hepatitis E | State of Kuwait | 0.1 (0-0.1) | 0 (0-0) | 0 (0-0) | 0 (0-0) | -0.14 (-0.18--0.09) |
| Acute hepatitis E | State of Libya | 1.4 (0.5-3.1) | 0 (0-0.1) | 0.9 (0.4-3.2) | 0 (0-0.1) | -0.91 (-1.45--0.36) |
| Acute hepatitis E | State of Qatar | 0.1 (0-0.2) | 0.1 (0-0.1) | 0.3 (0.1-0.6) | 0 (0-0) | -1.3 (-1.75--0.86) |
| Acute hepatitis E | Sultanate of Oman | 0.2 (0.1-0.6) | 0 (0-0) | 0.4 (0.2-0.8) | 0 (0-0) | -1.65 (-2.66--0.63) |
| Acute hepatitis E | Swiss Confederation | 0 (0-0.1) | 0 (0-0) | 0.8 (0.5-1.1) | 0 (0-0) | -0.11 (-0.16--0.06) |
| Acute hepatitis E | Syrian Arab Republic | 2.3 (0.9-4.3) | 0 (0-0.1) | 1.1 (0.5-2.2) | 0 (0-0) | -1.14 (-1.5--0.79) |
| Acute hepatitis E | Taiwan (Province of China) | 0.2 (0.1-0.3) | 0 (0-0) | 0.1 (0-0.1) | 0 (0-0) | -0.06 (-0.08--0.04) |
| Acute hepatitis E | Togolese Republic | 0.8 (0.3-1.7) | 0 (0-0.1) | 1 (0.3-2.3) | 0 (0-0) | -0.21 (-0.44-0.03) |
| Acute hepatitis E | Tokelau | 0 (0-0) | 0 (0-0.1) | 0 (0-0) | 0 (0-0) | -0.44 (-0.62--0.25) |
| Acute hepatitis E | Turkmenistan | 2.2 (1.3-4) | 0 (0-0.1) | 0.4 (0.3-0.7) | 0 (0-0) | -1.81 (-2.44--1.18) |
| Acute hepatitis E | Tuvalu | 0 (0-0) | 0 (0-0.1) | 0 (0-0) | 0 (0-0) | -0.71 (-0.93--0.48) |
| Acute hepatitis E | Ukraine | 3.3 (2.5-4.4) | 0 (0-0) | 0.8 (0.5-1.2) | 0 (0-0) | -0.22 (-0.37--0.08) |
| Acute hepatitis E | Union of the Comoros | 0.3 (0.1-0.8) | 0.1 (0-0.3) | 0.6 (0.2-1.4) | 0.1 (0-0.3) | -0.71 (-1.4--0.01) |
| Acute hepatitis E | United Arab Emirates | 0.5 (0.3-0.9) | 0.1 (0-0.1) | 1.8 (0.9-3.4) | 0 (0-0.1) | -0.43 (-0.95-0.1) |
| Acute hepatitis E | United Kingdom of Great Britain and Northern Ireland | 0.2 (0.2-0.2) | 0 (0-0) | 3.5 (3-3.9) | 0 (0-0) | 0.01 (0-0.02) |
| Acute hepatitis E | United Mexican States | 17.9 (15.3-21.1) | 0 (0-0) | 5.2 (3.6-7.1) | 0 (0-0) | -0.15 (-0.28--0.03) |
| Acute hepatitis E | United Republic of Tanzania | 30.6 (9.2-70.5) | 0.2 (0.1-0.4) | 29.5 (11-70.2) | 0.1 (0-0.2) | -1.7 (-2.49--0.91) |
| Acute hepatitis E | United States of America | 1.8 (1.5-2.1) | 0 (0-0) | 4.5 (3.3-5.5) | 0 (0-0) | -0.08 (-0.11--0.05) |
| Acute hepatitis E | United States Virgin Islands | 0 (0-0) | 0 (0-0) | 0 (0-0) | 0 (0-0) | -0.03 (-0.05--0.01) |

Table S3. The number of Acute hepatitis DALYs and age standardized DALYs rate of Acute hepatitis caused by different reasons from 1990 to 2021.

| cause | location | 1990 | | 2021 | | 1990-2021 |
| --- | --- | --- | --- | --- | --- | --- |
|  |  | Number of DALYs  No. ×103 (95% UI) | DALYs rate | Number of DALYs  No. ×103 (95% UI) | DALYs rate | EAPC  No. (95% CI) |
| Acute hepatitis | American Samoa | 7.8 (6.2-9.7) | 16.9 (13.7-21) | 5.9 (4.5-7.7) | 11.5 (8.9-15) | -1.11 (-2.46-0.26) |
| Acute hepatitis | Antigua and Barbuda | 4.5 (3.4-6) | 7.2 (5.5-9.6) | 4.2 (2.9-5.9) | 5 (3.4-7) | -0.78 (-1.93-0.39) |
| Acute hepatitis | Arab Republic of Egypt | 253692.2 (117674.4-380111.6) | 562.6 (243.8-824.7) | 91486.8 (37938.7-159140.3) | 110.9 (46-195.6) | -4.87 (-7.61--2.04) |
| Acute hepatitis | Argentine Republic | 3395.8 (2513.4-5389.9) | 10.1 (7.5-16) | 2213.2 (1567.9-3023.1) | 5 (3.5-6.9) | -1.42 (-2.73--0.08) |
| Acute hepatitis | Australia | 761.3 (534.4-1063.5) | 4.5 (3.2-6.2) | 1048.5 (708-1478.6) | 4 (2.7-5.7) | -0.4 (-1.37-0.58) |
| Acute hepatitis | Barbados | 32.6 (26.4-40.8) | 13.2 (10.7-16.7) | 25.1 (20.2-31.4) | 9 (7.1-11.4) | -0.66 (-2.13-0.83) |
| Acute hepatitis | Belize | 21.4 (16.5-27.3) | 9.8 (7.7-12.5) | 25.4 (17.7-35.3) | 5.6 (3.9-7.7) | -1.17 (-2.37-0.05) |
| Acute hepatitis | Bermuda | 2.9 (2-4.1) | 5.1 (3.5-7.3) | 2.2 (1.5-3.2) | 4.5 (3-6.6) | -0.29 (-1.41-0.85) |
| Acute hepatitis | Bolivarian Republic of Venezuela | 1946.6 (1508-2693.4) | 9 (7-12.4) | 2082.2 (1603-2692) | 8.4 (6.4-10.9) | -0.07 (-1.41-1.3) |
| Acute hepatitis | Bosnia and Herzegovina | 579.2 (397.6-834.4) | 13.2 (9-19.2) | 250 (180.7-336.1) | 7.6 (5.4-10.1) | -1.1 (-2.62-0.45) |
| Acute hepatitis | Brunei Darussalam | 244.9 (188.4-315.8) | 109.9 (85-138.4) | 79.9 (63.5-97.1) | 17.9 (14.1-22.2) | -5.38 (-7.2--3.54) |
| Acute hepatitis | Burkina Faso | 21063.8 (15398.5-29023.2) | 212.4 (163.9-276.4) | 20197.1 (12182.7-30645.6) | 93.5 (62.3-132.4) | -2.85 (-4.9--0.76) |
| Acute hepatitis | Canada | 1253.7 (936.5-1653.4) | 4.4 (3.3-5.9) | 1320.7 (921.3-1802) | 3.4 (2.3-4.7) | -0.73 (-1.63-0.18) |
| Acute hepatitis | Central African Republic | 13133.8 (6492-24886.4) | 380.8 (209.6-649.6) | 6760.5 (3706.7-10866.1) | 121.3 (67.8-194.2) | -3.15 (-5.29--0.97) |
| Acute hepatitis | Commonwealth of Dominica | 6.8 (5.1-9.1) | 9 (6.8-11.9) | 4.3 (3.1-5.7) | 6.9 (5.1-9.3) | -0.5 (-1.77-0.78) |
| Acute hepatitis | Commonwealth of the Bahamas | 21.8 (16.6-28.4) | 8 (6.1-10.2) | 21.3 (15-29.4) | 5.5 (3.9-7.6) | -0.79 (-2.02-0.46) |
| Acute hepatitis | Cook Islands | 1.4 (1-1.9) | 7.2 (5.1-9.9) | 0.9 (0.6-1.3) | 5.2 (3.6-7.4) | -0.64 (-1.78-0.53) |
| Acute hepatitis | Czech Republic | 853.9 (712.3-1042.4) | 8.7 (7.3-10.7) | 455 (338.5-616.3) | 4.6 (3.4-6.3) | -0.68 (-1.82-0.47) |
| Acute hepatitis | Democratic People's Republic of Korea | 14479.4 (8304.5-21873.2) | 68.5 (39.8-102.8) | 4109.6 (2949.8-5645.3) | 14.2 (10.4-19.3) | -4.22 (-5.95--2.45) |
| Acute hepatitis | Democratic Republic of Sao Tome and Principe | 108.3 (73.6-146) | 92.3 (64.1-126.9) | 39.9 (30-52.5) | 20.7 (15.9-26.9) | -4.3 (-6.24--2.33) |
| Acute hepatitis | Democratic Republic of the Congo | 21383.4 (13707.6-31989.4) | 43.2 (28.7-62.5) | 17645.6 (11684.2-25149.8) | 20.5 (13.4-30) | -1.76 (-3.44--0.04) |
| Acute hepatitis | Democratic Republic of Timor-Leste | 328.8 (158.7-714.3) | 38.6 (17.5-72) | 178.7 (104.8-270) | 13.3 (7.5-20.8) | -3.25 (-4.62--1.85) |
| Acute hepatitis | Democratic Socialist Republic of Sri Lanka | 4385.6 (3026.7-5926.1) | 27.5 (18.4-37.7) | 1832.5 (1329.9-2430.6) | 7.8 (5.7-10.4) | -3.58 (-4.86--2.28) |
| Acute hepatitis | Dominican Republic | 870.2 (672.4-1156.3) | 10.2 (7.9-13.4) | 924.2 (694.1-1214.3) | 8.4 (6.3-11) | -0.8 (-2.01-0.42) |
| Acute hepatitis | Eastern Republic of Uruguay | 186.4 (141.4-248.4) | 6.1 (4.6-8.2) | 136.1 (93.9-190.1) | 4.3 (2.9-6) | -0.65 (-1.9-0.61) |
| Acute hepatitis | Federal Democratic Republic of Ethiopia | 164577.5 (111076.2-233379.9) | 395.8 (276.6-558.6) | 145613.1 (104522.1-190759.9) | 165.6 (120.3-215.8) | -3.02 (-4.68--1.32) |
| Acute hepatitis | Federal Democratic Republic of Nepal | 114684 (60614.3-191005.3) | 440.7 (250.1-703.1) | 30204.1 (21461.7-40626.2) | 98.3 (70.8-131.6) | -2.12 (-4.38-0.2) |
| Acute hepatitis | Federal Republic of Germany | 4099.6 (3312-5149.3) | 4.7 (3.8-6) | 2837.6 (2073-3693.9) | 3 (2.2-4.1) | -0.79 (-1.71-0.14) |
| Acute hepatitis | Federal Republic of Nigeria | 730167.6 (329019.5-1146441.4) | 759.2 (332.8-1218) | 212870.1 (117715.2-325978.9) | 90.5 (50.3-137.7) | -7.86 (-9.86--5.81) |
| Acute hepatitis | Federal Republic of Somalia | 55299.3 (24743.7-94363.9) | 860.4 (391.3-1482.6) | 101442.2 (44692.6-174071.1) | 664.4 (307-1131.6) | -0.93 (-3.03-1.22) |
| Acute hepatitis | Federated States of Micronesia | 43.9 (28.2-68.3) | 44.3 (28-66.5) | 13.1 (9.5-18.3) | 12.8 (9.2-17.8) | -3.41 (-4.68--2.12) |
| Acute hepatitis | Federative Republic of Brazil | 22754.1 (19725.7-27338.9) | 15.2 (13.2-18.3) | 14874 (11650.7-18936.2) | 6.8 (5.3-8.8) | -1.62 (-2.97--0.24) |
| Acute hepatitis | French Republic | 2565.8 (1895.8-3444.2) | 4.4 (3.2-5.9) | 2328.5 (1648.2-3193.2) | 3.5 (2.4-4.8) | -0.65 (-1.69-0.41) |
| Acute hepatitis | Gabonese Republic | 860.8 (449.5-1379.7) | 88.8 (48.2-159.4) | 255.5 (163.6-410.1) | 14.7 (9.4-24) | -4.25 (-6.04--2.43) |
| Acute hepatitis | Georgia | 1707.5 (1431-2000.6) | 33.3 (27.8-39.2) | 218.4 (163.8-291.2) | 6.8 (5-9.1) | -3.6 (-4.92--2.27) |
| Acute hepatitis | Grand Duchy of Luxembourg | 13 (9-18.3) | 3.3 (2.3-4.7) | 18.7 (12.6-26.1) | 2.8 (1.8-3.9) | -0.4 (-1.3-0.5) |
| Acute hepatitis | Greenland | 4.4 (3.3-6.1) | 8 (6-11.1) | 2.3 (1.6-3.3) | 4.2 (2.9-5.9) | -1.43 (-2.58--0.26) |
| Acute hepatitis | Grenada | 12.5 (9.2-18.8) | 13.8 (10.2-20.8) | 6.9 (5.1-9.1) | 6.9 (5.1-9.1) | -1.48 (-2.82--0.12) |
| Acute hepatitis | Guam | 15.3 (11.6-19.8) | 11.3 (8.6-14.5) | 15.2 (11.7-19.6) | 9.4 (7.2-12.2) | -0.4 (-1.74-0.97) |
| Acute hepatitis | Hashemite Kingdom of Jordan | 1499.3 (989.3-2096.6) | 51.9 (33.3-71.7) | 1250.9 (966.7-1614.8) | 11.2 (8.5-14.3) | -4.04 (-5.55--2.51) |
| Acute hepatitis | Hellenic Republic | 766.1 (619.3-968.5) | 7.2 (5.7-9.1) | 636.9 (503.7-799.8) | 5.6 (4.3-7.2) | -0.6 (-1.95-0.76) |
| Acute hepatitis | Hungary | 701.8 (559.5-874.6) | 7.2 (5.7-8.9) | 410.9 (303.6-560.6) | 4.6 (3.3-6.3) | -0.54 (-1.61-0.54) |
| Acute hepatitis | Independent State of Papua New Guinea | 6088.2 (3128.6-9960.6) | 147.9 (68.3-249.9) | 4043.3 (2015.7-6787.1) | 38.3 (18-65) | -4.65 (-6.15--3.12) |
| Acute hepatitis | Independent State of Samoa | 30.5 (23-39.2) | 18.7 (14.5-23.8) | 23.9 (18.1-30.5) | 11.5 (8.7-14.5) | -1.31 (-2.7-0.11) |
| Acute hepatitis | Ireland | 135.6 (96.4-192.2) | 3.7 (2.7-5.3) | 157.8 (110.4-224.9) | 3.1 (2.2-4.6) | -0.45 (-1.45-0.56) |
| Acute hepatitis | Islamic Republic of Afghanistan | 120360 (83425.3-164043.8) | 1213 (840.6-1674.1) | 81459.4 (56899.7-119376.3) | 375.4 (251.6-543.7) | -4.7 (-7.44--1.88) |
| Acute hepatitis | Islamic Republic of Iran | 43627.1 (31283.8-55925.6) | 87.8 (63.2-114.9) | 18646.5 (15791.7-21807) | 21.7 (18.3-25.6) | -4.48 (-6.57--2.35) |
| Acute hepatitis | Islamic Republic of Mauritania | 9585.7 (3847.4-17314.3) | 554.8 (233.3-972.7) | 2470.1 (1533.1-3769.4) | 71.2 (43.8-111.8) | -6.22 (-8.59--3.79) |
| Acute hepatitis | Islamic Republic of Pakistan | 469519.1 (315953.1-641907.5) | 388.1 (269.4-521.2) | 367462.3 (262360.8-515604.6) | 169.5 (119.2-238.7) | -1.06 (-3.14-1.06) |
| Acute hepatitis | Jamaica | 221.4 (168.2-285.8) | 8.9 (6.8-11.3) | 190.4 (144.7-248.5) | 6.9 (5.2-9) | -0.46 (-1.74-0.82) |
| Acute hepatitis | Japan | 8553.6 (6587.6-10960.2) | 6.7 (5.2-8.5) | 6511.6 (4815.7-8713.3) | 4.9 (3.6-6.6) | -1.87 (-3.12--0.61) |
| Acute hepatitis | Kingdom of Bahrain | 282.8 (208.4-367.2) | 94.8 (68.5-127) | 363.9 (250-525.5) | 27.9 (19.2-39.7) | -3.91 (-6.19--1.58) |
| Acute hepatitis | Kingdom of Belgium | 517 (411.5-658.5) | 5 (4-6.4) | 428.2 (313.4-582.5) | 3.6 (2.5-5) | -0.83 (-1.84-0.2) |
| Acute hepatitis | Kingdom of Bhutan | 2224.4 (986.2-4044.8) | 295.6 (140.5-529.2) | 457.3 (259.2-721.7) | 64.2 (35.7-101.5) | -3.14 (-5.15--1.09) |
| Acute hepatitis | Kingdom of Cambodia | 52968.4 (40369.3-67341.3) | 642.6 (480.7-834.7) | 15765.5 (11460.9-22415.2) | 99.2 (72.6-140.3) | -5.97 (-7.83--4.07) |
| Acute hepatitis | Kingdom of Denmark | 203.1 (159.8-265.3) | 3.7 (2.9-4.8) | 184.2 (134.6-250) | 2.8 (2-3.9) | -0.64 (-1.57-0.3) |
| Acute hepatitis | Kingdom of Eswatini | 916.8 (504.9-1412.7) | 152.7 (80.8-246.4) | 492.2 (299.4-738) | 53.1 (32.8-79.9) | -2.9 (-5.1--0.65) |
| Acute hepatitis | Kingdom of Lesotho | 1915 (1065.1-2858.9) | 156.7 (86.1-241.2) | 1359.5 (865.7-1973.7) | 90.2 (57.8-130.1) | -1.2 (-3.49-1.14) |
| Acute hepatitis | Kingdom of Morocco | 18137.1 (12272.8-27069.7) | 81.5 (53.9-121.4) | 11020.3 (7653-16037.7) | 29.8 (20.8-42.9) | -2.98 (-5.08--0.84) |
| Acute hepatitis | Kingdom of Norway | 126.8 (84.4-180.4) | 3 (2-4.3) | 161.9 (112.3-226) | 2.9 (2-4.1) | -0.07 (-1-0.87) |
| Acute hepatitis | Kingdom of Saudi Arabia | 31469.7 (17791.6-50219.6) | 297.5 (152.6-504.4) | 7431.5 (5616.6-9739.7) | 21.5 (16.6-27.6) | -6.34 (-8.21--4.44) |
| Acute hepatitis | Kingdom of Spain | 1794.1 (1241.8-2510) | 4.5 (3.1-6.3) | 1736.6 (1158-2528.2) | 3.6 (2.4-5.2) | -0.43 (-1.4-0.55) |
| Acute hepatitis | Kingdom of Sweden | 196.8 (128-286) | 2.2 (1.5-3.3) | 236.3 (160.2-332.8) | 2.2 (1.4-3) | 0.01 (-0.78-0.81) |
| Acute hepatitis | Kingdom of Thailand | 17968.4 (13504.1-23006) | 34 (25.6-43.9) | 10435.6 (8405-12857.5) | 13.9 (11.4-17) | -1.49 (-3.31-0.36) |
| Acute hepatitis | Kingdom of the Netherlands | 463.6 (323.9-639.7) | 3 (2.1-4.2) | 478.4 (333.5-668.5) | 2.7 (1.8-3.8) | -0.3 (-1.21-0.61) |
| Acute hepatitis | Kingdom of Tonga | 468.4 (346.9-612.2) | 531.6 (384.6-691) | 131.3 (92.1-185.8) | 131.4 (93.3-185.6) | -4.2 (-5.89--2.48) |
| Acute hepatitis | Kyrgyz Republic | 9676.8 (7756.5-11636.1) | 181.6 (146.2-217.3) | 1079 (770.9-1496.4) | 15 (10.7-20.9) | -5.4 (-7.61--3.14) |
| Acute hepatitis | Lao People's Democratic Republic | 10610.5 (6459.1-16111.1) | 256.4 (162.9-369.4) | 2610 (1759.6-3954.5) | 37.2 (24.8-56.6) | -6.46 (-8.22--4.67) |
| Acute hepatitis | Lebanese Republic | 896.9 (610.2-1210.2) | 33.5 (22.5-45.7) | 685.1 (553.8-839.9) | 12.1 (9.7-14.8) | -2.48 (-3.92--1.01) |
| Acute hepatitis | Malaysia | 8366.7 (6700.8-10549.6) | 58.1 (45.6-73.4) | 9141.5 (7467.2-11147) | 28.7 (23.5-35) | -1.5 (-3.86-0.93) |
| Acute hepatitis | Mongolia | 3335.2 (1740.6-5989.9) | 120.4 (65.9-211.2) | 601.7 (444.8-802) | 17.6 (13.1-23.4) | -4.03 (-6.36--1.65) |
| Acute hepatitis | Montenegro | 29.1 (19.2-40.7) | 4.7 (3.1-6.6) | 22.9 (15.7-32.9) | 4.2 (2.8-6) | -0.27 (-1.33-0.81) |
| Acute hepatitis | New Zealand | 151.9 (109.2-209.4) | 4.4 (3.2-6) | 168.8 (108.3-244.7) | 3.2 (2.1-4.7) | -0.53 (-1.49-0.43) |
| Acute hepatitis | North Macedonia | 211.2 (146.7-299.7) | 11.5 (7.9-16.7) | 116.1 (83.3-158.7) | 5.9 (4.1-8.2) | -1.2 (-2.42-0.04) |
| Acute hepatitis | Northern Mariana Islands | 9.3 (7.1-11.9) | 21.5 (16.5-27) | 6.9 (5.5-8.6) | 13.1 (10.3-16.5) | -1.28 (-2.82-0.3) |
| Acute hepatitis | Palestine | 797.7 (513.8-1124.1) | 48.3 (28.9-71.3) | 518 (410.2-652.1) | 12.3 (9.9-15.2) | -3.55 (-5.1--1.98) |
| Acute hepatitis | People's Democratic Republic of Algeria | 17004.1 (12194.1-23733.2) | 74.6 (53-100.4) | 10630.9 (7754.4-14588.9) | 25.5 (18.5-35.1) | -2.98 (-5.09--0.83) |
| Acute hepatitis | People's Republic of Bangladesh | 416460.1 (335817.2-498499.1) | 419.5 (343.1-490.9) | 137099.5 (103359.5-182847.1) | 86.4 (65.1-114.5) | -3.42 (-5.51--1.28) |
| Acute hepatitis | People's Republic of China | 1656678.4 (1415695.9-1939344.5) | 152.8 (130.3-179) | 179097.5 (140781.3-224381.3) | 11.9 (9.3-15) | -7.21 (-8.96--5.43) |
| Acute hepatitis | Plurinational State of Bolivia | 1444.6 (987.2-2005.8) | 18.1 (12.9-24.6) | 1071.8 (788.2-1377.6) | 9.1 (6.7-11.6) | -1.34 (-2.89-0.24) |
| Acute hepatitis | Portuguese Republic | 776.8 (645.3-957.1) | 7.8 (6.5-9.6) | 498.9 (378.1-649.9) | 4.4 (3.2-5.9) | -1.48 (-2.75--0.19) |
| Acute hepatitis | Principality of Andorra | 16.7 (10.7-24.2) | 35.3 (21.7-53) | 14.5 (9.7-20.9) | 13.8 (9.3-19.4) | -2.52 (-3.86--1.16) |
| Acute hepatitis | Principality of Monaco | 1.1 (0.8-1.5) | 3.4 (2.4-4.7) | 1.3 (0.9-1.7) | 3.2 (2.2-4.3) | -0.14 (-1.06-0.78) |
| Acute hepatitis | Puerto Rico | 333.9 (266.8-420.1) | 9.3 (7.5-11.7) | 164.2 (123.6-221.2) | 5.7 (4.3-7.9) | -1.18 (-2.4-0.05) |
| Acute hepatitis | Republic of Albania | 664.7 (487.1-965.2) | 17.9 (13.1-25.8) | 162 (113.8-223.8) | 7.4 (5-10.8) | -1.88 (-3.19--0.56) |
| Acute hepatitis | Republic of Angola | 93156.5 (42887.4-176889.3) | 729.6 (369.9-1249.5) | 22515.5 (12148.4-38313.9) | 71.4 (41.2-109.8) | -7.15 (-9.33--4.91) |
| Acute hepatitis | Republic of Armenia | 1147.4 (852.3-1527) | 32 (23.9-42.6) | 178.5 (127.4-241.5) | 6.5 (4.6-8.8) | -2.86 (-4.35--1.35) |
| Acute hepatitis | Republic of Austria | 296 (210.3-407.4) | 3.8 (2.6-5.2) | 292.8 (200.4-408.7) | 3.2 (2.1-4.6) | -0.45 (-1.42-0.53) |
| Acute hepatitis | Republic of Azerbaijan | 6316.6 (4841.6-7999.9) | 76 (58.7-96.2) | 1416.1 (997-2051.5) | 14.8 (10.2-21.7) | -4.15 (-5.97--2.3) |
| Acute hepatitis | Republic of Belarus | 2527.8 (2176-2936.1) | 29 (24.9-34.2) | 982.1 (762.3-1229.4) | 12.8 (9.6-16.2) | -1.75 (-3.44--0.02) |
| Acute hepatitis | Republic of Benin | 7496.8 (5706.1-9982) | 158.4 (120.6-207.7) | 4598 (3032.8-6512.8) | 40 (26-57.2) | -4.25 (-6.18--2.29) |
| Acute hepatitis | Republic of Botswana | 902.8 (546.2-1339.3) | 92.1 (51.7-141.1) | 504.4 (338.9-712.9) | 23 (15.7-32.3) | -3.58 (-5.49--1.64) |
| Acute hepatitis | Republic of Bulgaria | 838.2 (647-1073.7) | 11.2 (8.7-14.4) | 459.6 (334.2-603) | 7.7 (5.5-10.1) | -0.77 (-2.1-0.58) |
| Acute hepatitis | Republic of Burundi | 7497.4 (3370-15297.3) | 151.7 (63.5-320.1) | 8112.1 (3973.6-13632.5) | 78.4 (38.9-136.7) | -1.39 (-3.2-0.45) |
| Acute hepatitis | Republic of Cabo Verde | 249.8 (197.8-314.4) | 83.8 (66.7-105.2) | 134.1 (104.9-174.6) | 24.2 (18.9-31.5) | -3.54 (-5.8--1.23) |
| Acute hepatitis | Republic of Cameroon | 4079.6 (2542.6-8107.2) | 42.9 (26.1-86.4) | 4876.4 (3252.3-7183) | 17.5 (11.5-26.2) | -2.22 (-3.74--0.67) |
| Acute hepatitis | Republic of Chad | 17078.4 (11998.2-23248.8) | 296.8 (204.4-404.7) | 24948.4 (17923-34818.8) | 178.9 (124.3-254) | -1.78 (-3.87-0.36) |
| Acute hepatitis | Republic of Chile | 1092.5 (890.9-1372.5) | 8 (6.6-10) | 826.2 (575.9-1155.8) | 4.6 (3.2-6.5) | -0.93 (-2.17-0.33) |
| Acute hepatitis | Republic of Colombia | 3543.2 (2813.1-4566) | 9.7 (7.8-12.5) | 2941.2 (2091.4-4016.2) | 6.3 (4.4-8.5) | -0.94 (-2.28-0.42) |
| Acute hepatitis | Republic of Costa Rica | 236 (178.2-312.5) | 7 (5.4-9.2) | 242.3 (174.9-329.1) | 5.4 (3.9-7.3) | -0.58 (-1.74-0.58) |
| Acute hepatitis | Republic of C么te d'Ivoire | 15296 (10288.4-20902.9) | 143.1 (98.9-193.2) | 6985.7 (5012-9348.1) | 29.1 (20.6-40) | -4.04 (-5.92--2.12) |
| Acute hepatitis | Republic of Croatia | 235.6 (158.7-333.4) | 5 (3.4-7.1) | 160.9 (107.6-228.3) | 4.3 (2.8-6.1) | -0.31 (-1.34-0.73) |
| Acute hepatitis | Republic of Cuba | 925 (715.8-1174.2) | 8.6 (6.7-10.9) | 474.8 (343.2-652.6) | 4.9 (3.5-6.9) | -1.07 (-2.33-0.21) |
| Acute hepatitis | Republic of Cyprus | 38.9 (29.5-51.1) | 5 (3.8-6.5) | 47.4 (33.6-65.7) | 3.2 (2.2-4.4) | -0.74 (-1.73-0.26) |
| Acute hepatitis | Republic of Djibouti | 400.3 (252.1-632.7) | 112.4 (73.5-169.7) | 795.2 (520-1168) | 72.1 (49.1-105.2) | -1.51 (-3.35-0.37) |
| Acute hepatitis | Republic of Ecuador | 1174.7 (946.9-1450.6) | 10.5 (8.5-12.9) | 1127.1 (835.9-1499.9) | 6.2 (4.6-8.2) | -1.19 (-2.53-0.18) |
| Acute hepatitis | Republic of El Salvador | 731.7 (542.6-973) | 10.9 (8.2-14.2) | 388.6 (282.7-525.5) | 6 (4.4-8.1) | -1.31 (-2.47--0.14) |
| Acute hepatitis | Republic of Equatorial Guinea | 97 (62.8-165.8) | 18.5 (12.4-31.9) | 122.9 (85-177.8) | 7.7 (5.4-11.1) | -2.15 (-3.4--0.88) |
| Acute hepatitis | Republic of Estonia | 275.9 (239.4-319.6) | 20.8 (17.9-24.1) | 55.4 (40.8-75.7) | 5.1 (3.7-7.1) | -2.85 (-4.32--1.35) |
| Acute hepatitis | Republic of Fiji | 273.5 (191.5-371.1) | 36.4 (25.5-49) | 100.2 (76.8-131) | 10.7 (8.2-14) | -3.25 (-4.66--1.81) |
| Acute hepatitis | Republic of Finland | 155.6 (113.7-214.6) | 2.9 (2.1-4) | 134.9 (91.4-182.8) | 2.2 (1.5-3.1) | -0.38 (-1.17-0.42) |
| Acute hepatitis | Republic of Ghana | 33632.1 (22173.3-47576.7) | 260.2 (172.7-370.2) | 8974.9 (6768.5-12154.7) | 30.3 (22.5-41.7) | -6.59 (-8.12--5.05) |
| Acute hepatitis | Republic of Guatemala | 1809 (1251-2709.4) | 15.2 (10.8-21.9) | 1255.7 (952.2-1640.9) | 7.9 (6.1-10.4) | -1.18 (-2.64-0.3) |
| Acute hepatitis | Republic of Guinea | 22195.3 (14013.1-32143.4) | 353.7 (218.4-499.4) | 12313.9 (8268.8-17884.8) | 112 (76.9-162.9) | -3.49 (-5.58--1.35) |
| Acute hepatitis | Republic of Guinea-Bissau | 3248.3 (2085.8-4807.9) | 368.4 (240.7-526.2) | 1629.4 (1197.9-2235.8) | 103.9 (75.5-142.8) | -3.81 (-5.96--1.61) |
| Acute hepatitis | Republic of Guyana | 214.8 (182.9-252.6) | 25.6 (22-29.7) | 82 (61.9-105.1) | 10.7 (8.1-13.7) | -0.97 (-2.37-0.46) |
| Acute hepatitis | Republic of Haiti | 5071.2 (2639.4-9332.6) | 58.6 (33.6-100.3) | 3688.7 (2336.3-5604.3) | 26.1 (17-38.9) | -1.59 (-3.61-0.48) |
| Acute hepatitis | Republic of Honduras | 2323.2 (1576.6-3348.7) | 35.3 (25.4-49.1) | 1238.4 (908.4-1731.8) | 12.7 (9.3-17.4) | -2.55 (-4.06--1.02) |
| Acute hepatitis | Republic of Iceland | 6.4 (4.7-8.8) | 2.5 (1.8-3.4) | 8.5 (5.8-11.9) | 2.3 (1.5-3.2) | -0.24 (-1.01-0.53) |
| Acute hepatitis | Republic of India | 3785867.3 (3147858.9-4580030.1) | 375.3 (317.6-443.4) | 1891678.2 (1345548.1-2791844) | 149.6 (104.4-225.4) | -0.94 (-3.37-1.55) |
| Acute hepatitis | Republic of Indonesia | 372354.7 (251759.1-528749.6) | 232.7 (153-337.1) | 209672.6 (169520.5-269886.7) | 73.2 (59.4-93.2) | -3.4 (-5.01--1.76) |
| Acute hepatitis | Republic of Iraq | 15258.9 (11485.1-20918.2) | 91.7 (69.8-120.3) | 9470.7 (7168.1-12334.9) | 27.2 (20.1-35.5) | -2.73 (-5--0.4) |
| Acute hepatitis | Republic of Italy | 3020.5 (2129.6-4176.2) | 5.2 (3.7-7.2) | 2951.4 (2199-3860.3) | 4.7 (3.4-6.3) | -0.39 (-1.54-0.78) |
| Acute hepatitis | Republic of Kazakhstan | 13463.5 (10808.8-16694.6) | 77.2 (61.7-95.5) | 1793.7 (1370-2281.4) | 9.5 (7.2-12) | -5.4 (-7.07--3.69) |
| Acute hepatitis | Republic of Kenya | 27716.6 (23257.2-34022) | 138.8 (114-173) | 31217.4 (25399.7-37818.6) | 77.1 (60.2-98.6) | -1.42 (-3.2-0.39) |
| Acute hepatitis | Republic of Kiribati | 281 (212.3-371.6) | 368.9 (275.2-481) | 168.5 (115.7-231.8) | 137.8 (95.1-185.5) | -2.5 (-5-0.07) |
| Acute hepatitis | Republic of Korea | 8029.7 (6417.8-9950.8) | 19.7 (15.9-24.6) | 3256.2 (2273.3-4491.9) | 5.6 (3.9-7.6) | -2.61 (-4.08--1.12) |
| Acute hepatitis | Republic of Latvia | 1325.9 (1174.5-1473) | 59.6 (52.4-66.8) | 137.6 (108.9-170.3) | 9 (7.1-11.2) | -3.76 (-5.79--1.69) |
| Acute hepatitis | Republic of Liberia | 3995.4 (3007.5-5312.5) | 158.7 (117.1-210.7) | 2828.8 (1904.4-4235.2) | 62 (41.8-93.6) | -3.66 (-5.67--1.6) |
| Acute hepatitis | Republic of Lithuania | 634.2 (539.5-747.8) | 19.5 (16.5-23.1) | 119.9 (90.2-162.3) | 5.5 (4.1-7.5) | -2.52 (-4.01--1) |
| Acute hepatitis | Republic of Madagascar | 14750.7 (12259.4-17541.3) | 133 (112.1-156.2) | 12122 (7541.7-17929.8) | 49.7 (31.7-71.8) | -2.64 (-4.58--0.66) |
| Acute hepatitis | Republic of Malawi | 25647.7 (19311.3-32069.6) | 266.3 (203.6-331.1) | 19846.7 (14318.8-27360.9) | 128.8 (95.8-171.4) | -2.82 (-5.17--0.41) |
| Acute hepatitis | Republic of Maldives | 123.6 (82-178.7) | 57.4 (38.4-80.5) | 46 (35.5-58.9) | 9 (7-11.5) | -4.76 (-6.13--3.36) |
| Acute hepatitis | Republic of Mali | 3089.5 (1867.3-6751.3) | 37 (21.9-73.8) | 4973.8 (3022.3-8275.2) | 24.9 (14.6-42.5) | -0.87 (-2.31-0.59) |
| Acute hepatitis | Republic of Malta | 15 (10.8-20.3) | 4 (2.9-5.5) | 14.9 (10.4-19.9) | 3.4 (2.3-4.6) | -0.47 (-1.53-0.61) |
| Acute hepatitis | Republic of Mauritius | 164.8 (135.8-200.7) | 15.9 (13.3-19.3) | 65.6 (46.1-91.7) | 5.3 (3.7-7.5) | -2.61 (-3.76--1.44) |
| Acute hepatitis | Republic of Moldova | 2395.9 (1971.7-2898) | 57.5 (47-69.9) | 293.9 (222.6-384.8) | 10.2 (7.8-13.4) | -2.97 (-4.83--1.07) |
| Acute hepatitis | Republic of Mozambique | 44788.6 (29686.3-67509.9) | 331.4 (218.6-479.7) | 21504.8 (14173.6-31021.6) | 86.3 (58.7-124.4) | -4.32 (-6.48--2.11) |
| Acute hepatitis | Republic of Namibia | 775.4 (479.2-1092.4) | 73.6 (43.7-106.2) | 611.6 (403.5-909.6) | 29.6 (19.5-43.6) | -2.61 (-4.62--0.56) |
| Acute hepatitis | Republic of Nauru | 4 (3-5.3) | 40.9 (30.6-54.4) | 1.7 (1.2-2.3) | 15.4 (10.8-20.8) | -2.81 (-4.39--1.2) |
| Acute hepatitis | Republic of Nicaragua | 1282.4 (893.3-1833.6) | 22.2 (16.1-30.5) | 446.7 (333.7-590.4) | 6.6 (5-8.7) | -2.27 (-3.67--0.84) |
| Acute hepatitis | Republic of Niue | 0.5 (0.4-0.6) | 20.6 (15.7-26.8) | 0.2 (0.1-0.3) | 11.9 (9.1-16.2) | -1.96 (-3.31--0.6) |
| Acute hepatitis | Republic of Palau | 12.5 (8.6-16.9) | 85.9 (58.6-117.6) | 7 (5.4-9.2) | 34.9 (27.3-44.4) | -2.16 (-4.22--0.06) |
| Acute hepatitis | Republic of Panama | 221.2 (173.6-283.3) | 8.4 (6.6-10.6) | 244.6 (179.4-319.6) | 5.9 (4.3-7.6) | -0.6 (-1.91-0.72) |
| Acute hepatitis | Republic of Paraguay | 518.4 (366.6-740.4) | 11.7 (8.2-17.1) | 511.4 (376.1-673.6) | 7.1 (5.3-9.3) | -0.9 (-2.37-0.59) |
| Acute hepatitis | Republic of Peru | 3425.4 (2579.7-4398.2) | 13.8 (10.5-17.4) | 2404.3 (1769.1-3212.6) | 6.7 (4.9-8.9) | -1.64 (-3--0.26) |
| Acute hepatitis | Republic of Poland | 1977.7 (1370.1-2770.2) | 5.4 (3.8-7.5) | 1410 (988.3-1962.3) | 4.3 (2.9-6) | -0.65 (-1.72-0.43) |
| Acute hepatitis | Republic of Rwanda | 18174.7 (13775.9-23840.9) | 284.4 (215-370.8) | 8609.4 (6044.1-11864.9) | 75.8 (54.8-104.5) | -4.62 (-6.71--2.49) |
| Acute hepatitis | Republic of San Marino | 8.4 (6.8-10.2) | 36 (28.5-45.3) | 4.8 (3.2-6.9) | 12 (8.2-17) | -2.72 (-4.15--1.27) |
| Acute hepatitis | Republic of Senegal | 4088.1 (2651.5-5842.6) | 57.2 (37.1-82.2) | 3099.2 (2054.3-4334.9) | 22.9 (15.1-32.8) | -2.58 (-4.34--0.79) |
| Acute hepatitis | Republic of Serbia | 856.1 (620.9-1125.4) | 9.9 (7-13.2) | 456.4 (334.1-611) | 5.6 (4.1-7.7) | -1.11 (-2.42-0.22) |
| Acute hepatitis | Republic of Seychelles | 54.1 (37.7-77.9) | 86.7 (59.8-127.3) | 32.2 (24.6-42.9) | 26.9 (20.7-35.3) | -3.46 (-4.82--2.07) |
| Acute hepatitis | Republic of Sierra Leone | 13376.9 (8042.8-20091) | 293.8 (175.9-427.3) | 4402.6 (3027.6-6205.8) | 57.5 (39.1-81.6) | -4.78 (-6.81--2.7) |
| Acute hepatitis | Republic of Singapore | 187.5 (126.3-266) | 5.9 (4.1-8.3) | 286.7 (191.7-420.6) | 4.4 (3-6.6) | -0.89 (-2.03-0.25) |
| Acute hepatitis | Republic of Slovenia | 131.8 (101.4-171.9) | 7.1 (5.5-9.2) | 84.3 (58.8-117.3) | 4.6 (3.2-6.5) | -0.6 (-1.74-0.55) |
| Acute hepatitis | Republic of South Africa | 11798.2 (9137.2-14849.6) | 35.2 (26.7-44.5) | 9147.1 (7483.5-11218.2) | 16.6 (13.6-20.3) | -1.51 (-3.33-0.33) |
| Acute hepatitis | Republic of South Sudan | 9390.6 (6849-13058.9) | 176.1 (125.9-256.4) | 13346 (8852.6-19169.4) | 167.2 (108.7-245.6) | 0.18 (-1.7-2.1) |
| Acute hepatitis | Republic of Sudan | 144274.5 (92673.3-211086.5) | 740.6 (414.4-1113.1) | 43627.2 (20785.6-73186.9) | 133 (60.7-227.6) | -5.56 (-8.19--2.87) |
| Acute hepatitis | Republic of Suriname | 56.4 (42.2-71.3) | 13.8 (10.4-17.3) | 46.1 (35.3-59.4) | 8.3 (6.4-10.7) | -1.26 (-2.59-0.09) |
| Acute hepatitis | Republic of Tajikistan | 18720 (14128.2-24713.9) | 241 (190.2-304.9) | 7313.4 (3869.6-14146.9) | 62.2 (33.9-116.9) | -3.52 (-6.25--0.72) |
| Acute hepatitis | Republic of the Congo | 8141.1 (2650.7-16495.8) | 304.8 (114.5-565.9) | 2248.2 (1093.8-3839.7) | 44.6 (21.7-76.3) | -5.4 (-7.71--3.03) |
| Acute hepatitis | Republic of the Gambia | 312.1 (189-504.5) | 36.3 (22-59.4) | 305.3 (198.6-489) | 14.8 (9.2-24.8) | -2.28 (-3.69--0.85) |
| Acute hepatitis | Republic of the Marshall Islands | 14.3 (9.1-20.9) | 33.2 (20-48.9) | 8 (6-10.9) | 14.1 (10.5-19.2) | -2.28 (-3.86--0.66) |
| Acute hepatitis | Republic of the Niger | 26353.8 (18517.5-37345.2) | 306.3 (220.3-418.9) | 16178.8 (10148.4-24804) | 83.7 (51-130.7) | -3.76 (-5.72--1.76) |
| Acute hepatitis | Republic of the Philippines | 76051.8 (63681.4-91922.3) | 115.5 (96.8-137.4) | 14957.6 (12034.4-18677.3) | 13.4 (10.7-16.6) | -6.29 (-7.94--4.6) |
| Acute hepatitis | Republic of the Union of Myanmar | 26213.6 (17931.7-41170.8) | 69.9 (49.8-104.8) | 13116.4 (9457.1-17418.4) | 23.4 (16.9-31.3) | -3.25 (-4.82--1.65) |
| Acute hepatitis | Republic of Trinidad and Tobago | 149.5 (118.6-187.2) | 12.3 (9.8-15.2) | 108.2 (83-139.3) | 8.2 (6.3-10.6) | -1.02 (-2.41-0.39) |
| Acute hepatitis | Republic of Tunisia | 5751.6 (3729.4-8941.7) | 74.5 (48.9-111.8) | 2748.3 (1820.2-3898.1) | 21.4 (14.3-29.9) | -2.9 (-4.97--0.79) |
| Acute hepatitis | Republic of Turkey | 52013.7 (36577-75972.5) | 85.5 (60.5-120.7) | 9899.6 (7965.4-12225.2) | 11.5 (9.3-14.2) | -5.64 (-7.36--3.89) |
| Acute hepatitis | Republic of Uganda | 43961.2 (24254.7-73643.5) | 286.1 (164.3-472.8) | 33331.6 (21366.6-49246.7) | 98.3 (67.7-137.2) | -3.92 (-5.86--1.95) |
| Acute hepatitis | Republic of Uzbekistan | 87002.4 (73907.8-102901.3) | 306.2 (262.7-356.3) | 7139.1 (5619.2-9004.7) | 20.3 (16-25.5) | -5.74 (-8.38--3.02) |
| Acute hepatitis | Republic of Vanuatu | 102 (49.9-162.6) | 72.4 (33.6-117.1) | 67 (39.1-98) | 22 (12.5-32.6) | -3.57 (-5.15--1.96) |
| Acute hepatitis | Republic of Yemen | 105405.9 (54999.7-182234.9) | 787.3 (367.1-1331.5) | 29715.9 (16340.3-48863.4) | 121.9 (62.3-204) | -6.83 (-9.44--4.15) |
| Acute hepatitis | Republic of Zambia | 24256.7 (16652.6-33801.4) | 325.9 (227.8-439.4) | 13437.4 (9265.7-18410.3) | 85.3 (59.3-114.8) | -4.32 (-6.62--1.97) |
| Acute hepatitis | Republic of Zimbabwe | 12438.3 (4149-21906.7) | 175.9 (54.5-316.1) | 13932.3 (6757.7-23832.9) | 117.5 (57.3-199.9) | -0.1 (-1.26-1.08) |
| Acute hepatitis | Romania | 2201 (1659.5-2881.6) | 10.4 (7.8-13.5) | 1001.9 (711.8-1396.8) | 5.7 (4-8.1) | -1.23 (-2.51-0.08) |
| Acute hepatitis | Russian Federation | 16131.3 (13837.4-19154.8) | 12.8 (11.1-15) | 6699.5 (4998.1-9009.2) | 5.8 (4.4-7.8) | -1.65 (-3.16--0.12) |
| Acute hepatitis | Saint Kitts and Nevis | 6.4 (4.9-8.4) | 15.4 (12-20.2) | 3.8 (2.9-5) | 7 (5.3-9.1) | -1.94 (-3.25--0.61) |
| Acute hepatitis | Saint Lucia | 17.7 (13.9-22.9) | 12.5 (9.9-16) | 10 (7.4-13.5) | 6.1 (4.4-8.2) | -1.62 (-2.88--0.34) |
| Acute hepatitis | Saint Vincent and the Grenadines | 6.5 (4.4-9.2) | 5.2 (3.6-7.3) | 6.2 (4.5-8.5) | 5.8 (4.1-7.9) | -0.18 (-1.42-1.08) |
| Acute hepatitis | Slovak Republic | 319.7 (225-435.3) | 6.2 (4.4-8.5) | 252.7 (183.8-352.2) | 5.2 (3.8-7.4) | -0.29 (-1.44-0.87) |
| Acute hepatitis | Socialist Republic of Viet Nam | 68933.6 (48245.8-97758.5) | 129.3 (88.8-189.2) | 18936 (14694.4-24394.3) | 17.9 (14-23) | -5.62 (-7.25--3.97) |
| Acute hepatitis | Solomon Islands | 550.8 (221-1012.2) | 176.2 (65.9-325.8) | 233.9 (118.1-380.7) | 35.3 (17.7-57.7) | -4.63 (-6.36--2.87) |
| Acute hepatitis | State of Eritrea | 6299.8 (4722.8-8471.2) | 224 (169.4-305.5) | 6109.1 (4129.2-9105.6) | 110.9 (76.6-162.2) | -1.86 (-3.76-0.07) |
| Acute hepatitis | State of Israel | 305.4 (242.8-377.8) | 6.2 (5-7.7) | 327.9 (235.1-449.4) | 3.4 (2.4-4.7) | -1.16 (-2.24--0.08) |
| Acute hepatitis | State of Kuwait | 120.2 (91.9-157.1) | 8 (6.3-10) | 215.6 (148.2-303.8) | 4.9 (3.4-6.9) | -1.34 (-2.38--0.29) |
| Acute hepatitis | State of Libya | 2559.7 (1314.5-4294.5) | 81.6 (39.1-145.1) | 2034.7 (1490.6-2940.2) | 31 (22.5-44.8) | -2.73 (-4.88--0.54) |
| Acute hepatitis | State of Qatar | 146.6 (114.1-186.2) | 59.2 (47.2-73.2) | 341.8 (250-456.9) | 16 (11.9-22) | -3.95 (-5.51--2.35) |
| Acute hepatitis | Sultanate of Oman | 3183.2 (2106.3-4342.2) | 250 (148.4-361.3) | 1232.2 (936.4-1604.9) | 37.6 (28.3-50.2) | -2.83 (-5.59-0.01) |
| Acute hepatitis | Swiss Confederation | 416.2 (352.1-493.5) | 5.7 (4.8-6.9) | 359.7 (279-468.5) | 3.6 (2.7-4.7) | -1.38 (-2.32--0.43) |
| Acute hepatitis | Syrian Arab Republic | 7200.3 (5329.3-9348.3) | 66.3 (47.3-92.2) | 1803.7 (1321.7-2378.8) | 12.6 (9.3-16.6) | -3.7 (-5.47--1.9) |
| Acute hepatitis | Taiwan (Province of China) | 2296.9 (1563.7-3244.8) | 10.8 (7.4-15.1) | 1514.5 (976.8-2137.1) | 6 (4-8.5) | -1.25 (-2.51-0.02) |
| Acute hepatitis | Togolese Republic | 1088.8 (711.4-1834.3) | 33.2 (21.6-54.8) | 1469.6 (953-2200.8) | 19.7 (12.6-30.4) | -1.25 (-2.83-0.35) |
| Acute hepatitis | Tokelau | 0.5 (0.3-0.7) | 32.9 (20.9-46.3) | 0.3 (0.2-0.5) | 24.2 (17.5-34) | -2.21 (-3.76--0.64) |
| Acute hepatitis | Turkmenistan | 16589.9 (13796-19862.1) | 331.5 (280.6-392.3) | 2647.1 (1796.3-3802.6) | 49.9 (34-71.7) | -5.13 (-7.34--2.87) |
| Acute hepatitis | Tuvalu | 4.8 (3.3-6.7) | 50.2 (36-69.7) | 1.8 (1.4-2.4) | 14.9 (11.3-19.4) | -3.29 (-4.83--1.73) |
| Acute hepatitis | Ukraine | 10261.2 (9043.3-11633.9) | 23.1 (19.9-26.6) | 2886.7 (2184-3787.2) | 8 (6.2-10.4) | -2 (-3.51--0.47) |
| Acute hepatitis | Union of the Comoros | 474 (294-679.5) | 114.5 (75.8-159.8) | 468.4 (307-686.1) | 68.9 (45.3-100) | -1.83 (-3.64-0.01) |
| Acute hepatitis | United Arab Emirates | 579.7 (410.3-807.4) | 56.7 (39.5-79.4) | 1803.4 (1308.5-2439.8) | 24.5 (18.8-32.2) | -1.8 (-3.51--0.05) |
| Acute hepatitis | United Kingdom of Great Britain and Northern Ireland | 1819.7 (1206.8-2578.5) | 3.2 (2.1-4.6) | 2250 (1566.1-3103.9) | 3.3 (2.3-4.7) | 0.15 (-0.8-1.11) |
| Acute hepatitis | United Mexican States | 22564 (19924.4-25758.1) | 21.5 (19.1-24.5) | 11679.8 (9587.9-14393.6) | 9.8 (8-12.1) | -1.53 (-2.95--0.08) |
| Acute hepatitis | United Republic of Tanzania | 52104.9 (38973.5-68844.3) | 201.9 (156.3-251.9) | 31349.5 (20990.4-43754.4) | 60 (41.9-81) | -3.52 (-5.36--1.64) |
| Acute hepatitis | United States of America | 13662.8 (10999.6-16982.6) | 5.3 (4.2-6.6) | 13521.2 (10344.3-17545.8) | 4.1 (3-5.3) | -1.11 (-2.15--0.07) |
| Acute hepatitis | United States Virgin Islands | 7.1 (5.3-9.6) | 6.5 (4.9-8.8) | 3.6 (2.6-5.1) | 5.2 (3.6-7.3) | -0.5 (-1.57-0.57) |
| Acute hepatitis A | American Samoa | 3.2 (2.4-4.3) | 6.3 (4.6-8.2) | 2.3 (1.7-3.2) | 4.5 (3.3-6.3) | -1.11 (-2.46-0.26) |
| Acute hepatitis A | Antigua and Barbuda | 3 (2.1-4.1) | 4.7 (3.4-6.4) | 2.9 (1.9-4.2) | 3.6 (2.4-5.3) | -0.78 (-1.93-0.39) |
| Acute hepatitis A | Arab Republic of Egypt | 123907.5 (48491.5-220791.7) | 243.5 (91-432.9) | 14841.1 (6767.6-33849) | 15.8 (6.5-38.5) | -4.87 (-7.61--2.04) |
| Acute hepatitis A | Argentine Republic | 2510.2 (1817.6-3861) | 7.4 (5.4-11.4) | 1704.2 (1131.4-2418.1) | 3.9 (2.6-5.6) | -1.42 (-2.73--0.08) |
| Acute hepatitis A | Australia | 423.3 (271.5-607.3) | 2.5 (1.6-3.6) | 596.3 (374.8-870.6) | 2.5 (1.6-3.7) | -0.4 (-1.37-0.58) |
| Acute hepatitis A | Barbados | 22 (17.1-27.5) | 8.9 (6.9-11.2) | 11.6 (8.4-15.7) | 4.6 (3.3-6.3) | -0.66 (-2.13-0.83) |
| Acute hepatitis A | Belize | 15.3 (11.7-19.7) | 6.6 (5.1-8.5) | 18.1 (12.2-25.8) | 3.8 (2.6-5.5) | -1.17 (-2.37-0.05) |
| Acute hepatitis A | Bermuda | 2.1 (1.4-3) | 3.8 (2.5-5.5) | 1.6 (1-2.3) | 3.5 (2.3-5.2) | -0.29 (-1.41-0.85) |
| Acute hepatitis A | Bolivarian Republic of Venezuela | 1577.3 (1206.5-2165.7) | 7.1 (5.5-9.8) | 1418.4 (1069.8-1907.6) | 5.8 (4.3-7.8) | -0.07 (-1.41-1.3) |
| Acute hepatitis A | Bosnia and Herzegovina | 192.5 (129.9-271.7) | 4.4 (2.9-6.2) | 83.4 (56.5-122.3) | 3.2 (2.1-4.7) | -1.1 (-2.62-0.45) |
| Acute hepatitis A | Brunei Darussalam | 34.3 (20-56.7) | 11.9 (7.1-19.6) | 16 (11-22.2) | 3.7 (2.5-5.2) | -5.38 (-7.2--3.54) |
| Acute hepatitis A | Burkina Faso | 5585.9 (2744.1-10267) | 59.3 (32.7-104.4) | 3709.1 (1938.8-7092.2) | 17.6 (8.5-32.6) | -2.85 (-4.9--0.76) |
| Acute hepatitis A | Canada | 644.3 (436.2-909.4) | 2.3 (1.6-3.3) | 824.2 (567.2-1158.7) | 2.2 (1.5-3.2) | -0.73 (-1.63-0.18) |
| Acute hepatitis A | Central African Republic | 6067.6 (1965.6-13686.4) | 140.8 (46.9-322) | 2474.9 (763-5392.5) | 36.2 (10.9-77.9) | -3.15 (-5.29--0.97) |
| Acute hepatitis A | Commonwealth of Dominica | 4.5 (3.4-6.2) | 5.8 (4.3-7.8) | 2.6 (1.8-3.6) | 4.2 (2.9-5.9) | -0.5 (-1.77-0.78) |
| Acute hepatitis A | Commonwealth of the Bahamas | 15.5 (11.4-20.6) | 5.5 (4.1-7.2) | 14.5 (9.6-21.1) | 3.8 (2.6-5.6) | -0.79 (-2.02-0.46) |
| Acute hepatitis A | Cook Islands | 0.8 (0.5-1.1) | 3.6 (2.4-5.1) | 0.5 (0.3-0.8) | 3.3 (2.2-4.9) | -0.64 (-1.78-0.53) |
| Acute hepatitis A | Czech Republic | 646.5 (537-785.5) | 6.7 (5.6-8.2) | 289.2 (205.4-400.5) | 3.2 (2.2-4.6) | -0.68 (-1.82-0.47) |
| Acute hepatitis A | Democratic People's Republic of Korea | 3244.9 (1478.7-7414.8) | 15.2 (7-33.9) | 1023.3 (660.9-1498.9) | 4 (2.6-5.9) | -4.22 (-5.95--2.45) |
| Acute hepatitis A | Democratic Republic of Sao Tome and Principe | 38.3 (21.2-58) | 32 (19.3-49.7) | 12.1 (8.3-16.6) | 5.4 (3.8-7.5) | -4.3 (-6.24--2.33) |
| Acute hepatitis A | Democratic Republic of the Congo | 13557.6 (7322.1-22220.5) | 22.4 (13.4-35.6) | 7016.7 (3899.4-11132.3) | 6.6 (3.5-11.1) | -1.76 (-3.44--0.04) |
| Acute hepatitis A | Democratic Republic of Timor-Leste | 217.5 (83.2-548.3) | 22.6 (8.1-50.5) | 98.2 (54.9-151.9) | 6.7 (3.4-10.8) | -3.25 (-4.62--1.85) |
| Acute hepatitis A | Democratic Socialist Republic of Sri Lanka | 2126 (1378.7-3182.5) | 12.4 (7.9-19) | 735.1 (514.2-1038.8) | 3.4 (2.4-4.8) | -3.58 (-4.86--2.28) |
| Acute hepatitis A | Dominican Republic | 553.1 (410.7-772.7) | 6.1 (4.5-8.6) | 470.8 (324-672.5) | 4.2 (2.9-6) | -0.8 (-2.01-0.42) |
| Acute hepatitis A | Eastern Republic of Uruguay | 155.3 (114.5-209.6) | 5.2 (3.8-7) | 112.8 (76.4-159.7) | 3.7 (2.5-5.3) | -0.65 (-1.9-0.61) |
| Acute hepatitis A | Federal Democratic Republic of Ethiopia | 87069.3 (44570.6-138444.1) | 173.1 (81.4-304.3) | 48064.9 (19464.4-114673.8) | 47.6 (17.3-122.6) | -3.02 (-4.68--1.32) |
| Acute hepatitis A | Federal Democratic Republic of Nepal | 105948.8 (54827-177123.6) | 396.5 (215.4-637.7) | 16870.9 (10244.9-27163.1) | 54.2 (32.7-88.1) | -2.12 (-4.38-0.2) |
| Acute hepatitis A | Federal Republic of Germany | 1896.1 (1309.2-2626.1) | 2.3 (1.6-3.3) | 1828.4 (1199.4-2503.8) | 2.2 (1.4-3.1) | -0.79 (-1.71-0.14) |
| Acute hepatitis A | Federal Republic of Nigeria | 180830.2 (66613.9-359857.3) | 203.9 (84.1-378.9) | 35647.3 (20879-57584.5) | 15.6 (9.2-25.9) | -7.86 (-9.86--5.81) |
| Acute hepatitis A | Federal Republic of Somalia | 19954.5 (8174.8-41472.4) | 223.7 (88.1-558.5) | 18777.6 (5099.3-53912.4) | 97.6 (24.4-340.4) | -0.93 (-3.03-1.22) |
| Acute hepatitis A | Federated States of Micronesia | 10.4 (4.3-22.6) | 8.3 (3.5-18.1) | 4.1 (2.5-6.2) | 3.7 (2.3-5.7) | -3.41 (-4.68--2.12) |
| Acute hepatitis A | Federative Republic of Brazil | 15868.6 (13573.2-19092) | 10.2 (8.8-12.2) | 8469.6 (6080.2-11708.5) | 4.1 (2.9-5.7) | -1.62 (-2.97--0.24) |
| Acute hepatitis A | French Republic | 1685.9 (1143.7-2371.3) | 3 (2-4.3) | 1652 (1132.1-2327.6) | 2.6 (1.7-3.8) | -0.65 (-1.69-0.41) |
| Acute hepatitis A | Gabonese Republic | 253.7 (90.4-464.8) | 21 (7.7-37) | 90.9 (62.7-130.8) | 4.5 (3.1-6.5) | -4.25 (-6.04--2.43) |
| Acute hepatitis A | Georgia | 313.9 (216.8-445.9) | 6.2 (4.3-8.9) | 103.8 (68.8-148.1) | 3.6 (2.4-5.2) | -3.6 (-4.92--2.27) |
| Acute hepatitis A | Grand Duchy of Luxembourg | 8.5 (5.5-12.6) | 2.2 (1.4-3.3) | 13.3 (8.6-19.1) | 2.1 (1.3-3) | -0.4 (-1.3-0.5) |
| Acute hepatitis A | Greenland | 2.6 (1.9-3.7) | 4.8 (3.5-6.7) | 1.6 (1.1-2.3) | 3 (2-4.3) | -1.43 (-2.58--0.26) |
| Acute hepatitis A | Grenada | 6.4 (4.4-8.9) | 6.5 (4.5-9.1) | 3.6 (2.3-5.2) | 3.7 (2.4-5.3) | -1.48 (-2.82--0.12) |
| Acute hepatitis A | Guam | 6.2 (4.3-8.6) | 4.3 (3-5.9) | 5.8 (4.1-8) | 3.9 (2.7-5.4) | -0.4 (-1.74-0.97) |
| Acute hepatitis A | Hashemite Kingdom of Jordan | 537.6 (353.5-814.1) | 15.9 (9.9-25.2) | 589.1 (409.3-836) | 4.7 (3.3-6.6) | -4.04 (-5.55--2.51) |
| Acute hepatitis A | Hellenic Republic | 295.6 (197.9-424.5) | 2.9 (2-4.2) | 250.4 (165.3-354) | 2.8 (1.8-4) | -0.6 (-1.95-0.76) |
| Acute hepatitis A | Hungary | 400.2 (304.7-517.1) | 4.2 (3.2-5.4) | 216.1 (144.7-308.7) | 2.8 (1.8-4) | -0.54 (-1.61-0.54) |
| Acute hepatitis A | Independent State of Papua New Guinea | 2677.7 (1137.9-5514.2) | 55.8 (22.1-128.9) | 2065.2 (942.7-3644.1) | 18.1 (8-33.2) | -4.65 (-6.15--3.12) |
| Acute hepatitis A | Independent State of Samoa | 12.1 (7.8-17.7) | 6.2 (4.1-9.1) | 10 (6.7-14.1) | 4.3 (2.9-6.1) | -1.31 (-2.7-0.11) |
| Acute hepatitis A | Ireland | 103.9 (71-151) | 2.9 (2-4.2) | 125.5 (86.6-181.3) | 2.6 (1.7-3.8) | -0.45 (-1.45-0.56) |
| Acute hepatitis A | Islamic Republic of Afghanistan | 63770.1 (29671.7-98845.9) | 564.2 (243.2-912.3) | 38199.7 (18920.8-63819.4) | 138.1 (58.5-253.2) | -4.7 (-7.44--1.88) |
| Acute hepatitis A | Islamic Republic of Iran | 17980.7 (11953.1-23632.7) | 29.2 (19.4-40.5) | 3889.7 (2797.1-5439.1) | 4.8 (3.4-6.6) | -4.48 (-6.57--2.35) |
| Acute hepatitis A | Islamic Republic of Mauritania | 2357.6 (956.1-5704) | 154 (62.7-356.5) | 525.8 (327.9-808.3) | 14.8 (8.5-24.2) | -6.22 (-8.59--3.79) |
| Acute hepatitis A | Islamic Republic of Pakistan | 374617.8 (231208.2-542224.2) | 291.5 (173.7-425.1) | 179639.7 (88871.6-345631.8) | 76.8 (35.9-153.8) | -1.06 (-3.14-1.06) |
| Acute hepatitis A | Jamaica | 164.7 (122.9-213.7) | 6.3 (4.8-8.2) | 117.1 (81-162.5) | 4.3 (3-5.9) | -0.46 (-1.74-0.82) |
| Acute hepatitis A | Japan | 3221 (2288.1-4356.3) | 2.8 (2.1-3.7) | 2516.7 (1651.2-3575.9) | 2.4 (1.6-3.5) | -1.87 (-3.12--0.61) |
| Acute hepatitis A | Kingdom of Bahrain | 60.4 (41.7-86.5) | 17.9 (11.2-28.5) | 67.5 (46.5-96.8) | 4.9 (3.4-7.1) | -3.91 (-6.19--1.58) |
| Acute hepatitis A | Kingdom of Belgium | 275.3 (190.9-383.2) | 2.8 (2-3.9) | 276.6 (183.7-400) | 2.5 (1.6-3.7) | -0.83 (-1.84-0.2) |
| Acute hepatitis A | Kingdom of Bhutan | 1913.2 (722.5-3684) | 243 (100.8-474.1) | 181.7 (88.9-423.2) | 26 (12.7-59.6) | -3.14 (-5.15--1.09) |
| Acute hepatitis A | Kingdom of Cambodia | 33338.1 (21682.9-46872.5) | 378.9 (238.7-561.3) | 6325.3 (3624.6-10548.5) | 39.6 (22.5-65.9) | -5.97 (-7.83--4.07) |
| Acute hepatitis A | Kingdom of Denmark | 107.5 (75.5-152.3) | 2 (1.4-2.9) | 112.5 (75.8-159) | 1.8 (1.2-2.7) | -0.64 (-1.57-0.3) |
| Acute hepatitis A | Kingdom of Eswatini | 417.9 (246.1-720.5) | 47 (25.4-85.3) | 140.8 (83-213) | 13 (7.3-20.3) | -2.9 (-5.1--0.65) |
| Acute hepatitis A | Kingdom of Lesotho | 714.8 (376.3-1309.5) | 44.4 (19.8-92.4) | 369 (196.9-680.4) | 22.1 (11.2-42) | -1.2 (-3.49-1.14) |
| Acute hepatitis A | Kingdom of Morocco | 10122.5 (6340.8-15762.4) | 40.3 (23.2-65.6) | 2822.9 (1854.5-4565.9) | 7.7 (5.1-12.4) | -2.98 (-5.08--0.84) |
| Acute hepatitis A | Kingdom of Norway | 95.5 (60.9-138.7) | 2.3 (1.4-3.4) | 119.1 (78.4-170.3) | 2.3 (1.5-3.3) | -0.07 (-1-0.87) |
| Acute hepatitis A | Kingdom of Saudi Arabia | 12286.7 (5948.8-22260.4) | 102.9 (41.5-208.7) | 2137.2 (1532.8-2822.7) | 5.9 (4.2-7.8) | -6.34 (-8.21--4.44) |
| Acute hepatitis A | Kingdom of Spain | 986.4 (641.2-1410.1) | 2.5 (1.7-3.6) | 1009.2 (656.2-1479.2) | 2.4 (1.5-3.6) | -0.43 (-1.4-0.55) |
| Acute hepatitis A | Kingdom of Sweden | 143.1 (89.2-210.2) | 1.7 (1-2.5) | 162.4 (106.3-231.3) | 1.6 (1-2.2) | 0.01 (-0.78-0.81) |
| Acute hepatitis A | Kingdom of Thailand | 3123.6 (2003.1-4437.2) | 5.1 (3.3-7.3) | 2553.1 (1930.3-3396.2) | 4.4 (3.3-5.9) | -1.49 (-3.31-0.36) |
| Acute hepatitis A | Kingdom of the Netherlands | 346.5 (227.4-497) | 2.3 (1.5-3.3) | 355.7 (236.2-507.9) | 2.1 (1.4-3.1) | -0.3 (-1.21-0.61) |
| Acute hepatitis A | Kingdom of Tonga | 151.3 (87.2-242.7) | 154.5 (86.4-253.9) | 40.1 (23.5-62.8) | 37.5 (22.1-58.5) | -4.2 (-5.89--2.48) |
| Acute hepatitis A | Kyrgyz Republic | 5502.2 (4079-7027.2) | 101.7 (75.6-129.3) | 524.9 (379.1-726) | 7.1 (5.1-9.8) | -5.4 (-7.61--3.14) |
| Acute hepatitis A | Lao People's Democratic Republic | 5720.1 (2610-9507.5) | 122.3 (53.8-203.1) | 976.7 (547.9-1525.7) | 13.4 (7.3-21.5) | -6.46 (-8.22--4.67) |
| Acute hepatitis A | Lebanese Republic | 538.9 (331.3-741.3) | 19.8 (11.9-27.4) | 317.7 (245.5-413.5) | 5.8 (4.4-7.7) | -2.48 (-3.92--1.01) |
| Acute hepatitis A | Malaysia | 1157.3 (839.8-1541.7) | 6.1 (4.4-8.1) | 1211.8 (853.7-1671.8) | 3.7 (2.6-5.2) | -1.5 (-3.86-0.93) |
| Acute hepatitis A | Mongolia | 1235.3 (513.3-2359.3) | 40.7 (17.8-76.6) | 185.1 (128.8-259.2) | 5.3 (3.7-7.5) | -4.03 (-6.36--1.65) |
| Acute hepatitis A | Montenegro | 20.8 (13.3-29.9) | 3.3 (2.1-4.8) | 16.5 (10.8-24.3) | 3.2 (2.1-4.7) | -0.27 (-1.33-0.81) |
| Acute hepatitis A | New Zealand | 82.4 (54.9-118.9) | 2.4 (1.6-3.5) | 112.5 (71.6-164.3) | 2.3 (1.4-3.4) | -0.53 (-1.49-0.43) |
| Acute hepatitis A | North Macedonia | 116.3 (80.3-165.3) | 6.2 (4.2-9) | 58.6 (37.6-85) | 3.3 (2.1-4.7) | -1.2 (-2.42-0.04) |
| Acute hepatitis A | Northern Mariana Islands | 2.5 (1.8-3.4) | 5.5 (4-7.3) | 1.9 (1.4-2.6) | 4.2 (2.9-5.7) | -1.28 (-2.82-0.3) |
| Acute hepatitis A | Palestine | 335.8 (198.5-525.5) | 16.3 (8.9-28.1) | 232.6 (165.1-326) | 4.4 (3.3-6) | -3.55 (-5.1--1.98) |
| Acute hepatitis A | People's Democratic Republic of Algeria | 6186.6 (3662.2-10966.6) | 22.4 (13.2-40.1) | 2317.4 (1511.8-3667.6) | 5.3 (3.4-8.5) | -2.98 (-5.09--0.83) |
| Acute hepatitis A | People's Republic of Bangladesh | 353551.1 (269815.3-457233.1) | 339.8 (259.8-434) | 75406.7 (44316.7-116986.8) | 47.6 (28.1-73.6) | -3.42 (-5.51--1.28) |
| Acute hepatitis A | People's Republic of China | 723349.2 (515992.8-982603.3) | 65.7 (46.7-90) | 50928.6 (37153.2-68437.5) | 4.1 (2.9-5.6) | -7.21 (-8.96--5.43) |
| Acute hepatitis A | Plurinational State of Bolivia | 1191.9 (826.7-1644.2) | 14.4 (10.2-19.7) | 756.8 (553.6-981.1) | 6.3 (4.6-8.1) | -1.34 (-2.89-0.24) |
| Acute hepatitis A | Portuguese Republic | 310.7 (204.3-441.4) | 3.2 (2.1-4.5) | 249.8 (163.7-367.2) | 2.6 (1.7-3.9) | -1.48 (-2.75--0.19) |
| Acute hepatitis A | Principality of Andorra | 6.4 (3.9-10.3) | 14.3 (8.3-23.8) | 4.4 (2.8-6.2) | 4.6 (3.1-6.3) | -2.52 (-3.86--1.16) |
| Acute hepatitis A | Principality of Monaco | 0.6 (0.4-0.9) | 2.2 (1.4-3.2) | 0.7 (0.5-1.1) | 2.1 (1.4-3.1) | -0.14 (-1.06-0.78) |
| Acute hepatitis A | Puerto Rico | 233.3 (180.2-298.4) | 6.5 (5-8.3) | 103.3 (73.1-146.9) | 4 (2.8-5.8) | -1.18 (-2.4-0.05) |
| Acute hepatitis A | Republic of Albania | 394.9 (284.6-551.3) | 10.5 (7.6-14.5) | 79.2 (53.4-113.2) | 3.8 (2.5-5.3) | -1.88 (-3.19--0.56) |
| Acute hepatitis A | Republic of Angola | 42241.5 (10195.4-102759.1) | 249.2 (63.7-589.8) | 5050.7 (1956.5-11286.9) | 11.7 (4.6-25.6) | -7.15 (-9.33--4.91) |
| Acute hepatitis A | Republic of Armenia | 327.7 (241.3-446.2) | 9.1 (6.7-12.3) | 88.3 (57.5-126) | 3.5 (2.3-5) | -2.86 (-4.35--1.35) |
| Acute hepatitis A | Republic of Austria | 193.7 (127.3-276.1) | 2.5 (1.7-3.7) | 206.4 (135.9-291.2) | 2.4 (1.5-3.4) | -0.45 (-1.42-0.53) |
| Acute hepatitis A | Republic of Azerbaijan | 1976.3 (1230.5-2874.1) | 23.3 (14.7-33.6) | 414.9 (286.7-594.8) | 4.3 (3-6.3) | -4.15 (-5.97--2.3) |
| Acute hepatitis A | Republic of Belarus | 892.4 (637.1-1103.8) | 9.8 (6.7-12.1) | 379.4 (284.1-503.1) | 4.8 (3.5-6.5) | -1.75 (-3.44--0.02) |
| Acute hepatitis A | Republic of Benin | 2401.7 (1409.7-3968.5) | 55.6 (36-93.2) | 1325.2 (864.1-2055.8) | 11.2 (7.1-17.9) | -4.25 (-6.18--2.29) |
| Acute hepatitis A | Republic of Botswana | 373.4 (234.3-594.6) | 28.5 (15.9-50) | 159 (116.5-222.3) | 6.9 (5-9.6) | -3.58 (-5.49--1.64) |
| Acute hepatitis A | Republic of Bulgaria | 461.4 (339.2-604.6) | 6.5 (4.9-8.6) | 189.3 (130.5-264.4) | 4 (2.7-5.6) | -0.77 (-2.1-0.58) |
| Acute hepatitis A | Republic of Burundi | 3226.1 (1266.6-6892.7) | 52.4 (17.6-120.1) | 2464 (908.6-6628.6) | 19.6 (6.1-59.6) | -1.39 (-3.2-0.45) |
| Acute hepatitis A | Republic of Cabo Verde | 34.4 (20-56.7) | 10.5 (6-17.3) | 25.3 (17.8-34.9) | 4.5 (3.1-6.1) | -3.54 (-5.8--1.23) |
| Acute hepatitis A | Republic of Cameroon | 1849.5 (1000.5-3498.8) | 19.3 (10.5-37.9) | 2015.2 (1296.9-3126.8) | 6.5 (4-10.5) | -2.22 (-3.74--0.67) |
| Acute hepatitis A | Republic of Chad | 4946.8 (2524.1-9154.5) | 92.3 (51.3-174) | 5452.6 (3094.1-9419.1) | 41.5 (22.6-72.4) | -1.78 (-3.87-0.36) |
| Acute hepatitis A | Republic of Chile | 930 (751.1-1176.1) | 6.7 (5.5-8.5) | 640.4 (429.9-930.2) | 3.7 (2.5-5.5) | -0.93 (-2.17-0.33) |
| Acute hepatitis A | Republic of Colombia | 2473.6 (1974.7-3211.2) | 6.5 (5.2-8.4) | 1931.8 (1334.5-2658.8) | 4.2 (2.9-5.7) | -0.94 (-2.28-0.42) |
| Acute hepatitis A | Republic of Costa Rica | 173 (124.6-233.6) | 5 (3.6-6.6) | 175.6 (121.8-247.2) | 4 (2.7-5.6) | -0.58 (-1.74-0.58) |
| Acute hepatitis A | Republic of C么te d'Ivoire | 4441.5 (2615.4-6897.3) | 45 (27.4-71.6) | 2205.3 (1521.6-3100.3) | 8.5 (5.8-12.3) | -4.04 (-5.92--2.12) |
| Acute hepatitis A | Republic of Croatia | 144.1 (94.5-202.5) | 3.2 (2.1-4.6) | 93.7 (58.7-137.5) | 2.9 (1.8-4.3) | -0.31 (-1.34-0.73) |
| Acute hepatitis A | Republic of Cuba | 688.1 (517.7-887.1) | 6.4 (4.9-8.2) | 329.9 (224.7-470) | 3.7 (2.5-5.4) | -1.07 (-2.33-0.21) |
| Acute hepatitis A | Republic of Cyprus | 21.5 (14.8-30) | 2.8 (1.9-3.9) | 29.3 (19-42.7) | 2.1 (1.4-3.2) | -0.74 (-1.73-0.26) |
| Acute hepatitis A | Republic of Djibouti | 160.8 (76-303.1) | 37.2 (16.9-74) | 201.9 (96.8-399.3) | 17.4 (8.1-35.9) | -1.51 (-3.35-0.37) |
| Acute hepatitis A | Republic of Ecuador | 879.2 (689.8-1110) | 7.5 (5.9-9.4) | 842.1 (618-1138.9) | 4.6 (3.4-6.2) | -1.19 (-2.53-0.18) |
| Acute hepatitis A | Republic of El Salvador | 430.2 (300.2-572.6) | 6.2 (4.4-8.2) | 246.7 (166.8-358.3) | 3.7 (2.5-5.4) | -1.31 (-2.47--0.14) |
| Acute hepatitis A | Republic of Equatorial Guinea | 67.8 (39.5-130.5) | 11.1 (6.6-20.3) | 71.8 (46.2-106.7) | 3.8 (2.5-5.7) | -2.15 (-3.4--0.88) |
| Acute hepatitis A | Republic of Estonia | 120.6 (99.5-148.7) | 8.6 (7.1-10.7) | 36.1 (24.7-51.5) | 3.5 (2.3-5.1) | -2.85 (-4.32--1.35) |
| Acute hepatitis A | Republic of Fiji | 119.3 (78-181.9) | 14.5 (9.3-22.4) | 47 (33.4-63.3) | 5 (3.5-6.7) | -3.25 (-4.66--1.81) |
| Acute hepatitis A | Republic of Finland | 90.6 (62.2-128.4) | 1.7 (1.2-2.5) | 86.5 (55.8-118.4) | 1.5 (0.9-2.1) | -0.38 (-1.17-0.42) |
| Acute hepatitis A | Republic of Ghana | 9423.4 (6263.8-13927.7) | 85 (53.9-127.8) | 3862.9 (2678.9-5330.6) | 13.2 (8.8-19.2) | -6.59 (-8.12--5.05) |
| Acute hepatitis A | Republic of Guatemala | 1370.1 (930.8-2061.7) | 10.9 (7.5-16) | 873.9 (657.2-1173.2) | 5.3 (4-7.1) | -1.18 (-2.64-0.3) |
| Acute hepatitis A | Republic of Guinea | 5957.1 (3016.7-10844) | 103.3 (53.6-183.6) | 2652.2 (1606.4-4416.1) | 25 (14.6-43.1) | -3.49 (-5.58--1.35) |
| Acute hepatitis A | Republic of Guinea-Bissau | 1009.6 (560.8-1812.4) | 129.6 (76.3-221.6) | 432.7 (262.8-680.8) | 29.3 (17.1-45.9) | -3.81 (-5.96--1.61) |
| Acute hepatitis A | Republic of Guyana | 186.3 (158.1-219.7) | 21.8 (18.6-25.3) | 39.3 (28.2-52.9) | 5 (3.6-6.7) | -0.97 (-2.37-0.46) |
| Acute hepatitis A | Republic of Haiti | 4454.7 (2225.4-8069.1) | 49.2 (25.5-84.9) | 2634.7 (1596.6-4013.8) | 18 (11-27) | -1.59 (-3.61-0.48) |
| Acute hepatitis A | Republic of Honduras | 1647.3 (946-2501.5) | 24.3 (14.4-36.1) | 750.3 (530.1-1010.8) | 7.4 (5.2-9.9) | -2.55 (-4.06--1.02) |
| Acute hepatitis A | Republic of Iceland | 3.9 (2.6-5.7) | 1.5 (1-2.2) | 5.6 (3.6-8.1) | 1.5 (1-2.2) | -0.24 (-1.01-0.53) |
| Acute hepatitis A | Republic of India | 3265356.6 (2645523.2-3994811.1) | 318 (259.4-386.8) | 976391.2 (596791.2-1768595.9) | 78.1 (46.4-145.7) | -0.94 (-3.37-1.55) |
| Acute hepatitis A | Republic of Indonesia | 247312.8 (151666.4-357490.1) | 150.1 (88.5-220.9) | 83822.9 (50661.2-140178.1) | 29.8 (18.1-49.2) | -3.4 (-5.01--1.76) |
| Acute hepatitis A | Republic of Iraq | 10236.3 (7334.1-14228.8) | 56.9 (39.2-77.6) | 3914.5 (2996.4-5037.3) | 10.6 (8-13.6) | -2.73 (-5--0.4) |
| Acute hepatitis A | Republic of Italy | 1505.9 (984-2131.7) | 2.8 (1.8-3.9) | 1578.7 (1082-2214.3) | 3.1 (2-4.4) | -0.39 (-1.54-0.78) |
| Acute hepatitis A | Republic of Kazakhstan | 2690.8 (1829.8-3587.4) | 15.2 (10.5-20.2) | 669.5 (459.4-986.5) | 3.6 (2.5-5.4) | -5.4 (-7.07--3.69) |
| Acute hepatitis A | Republic of Kenya | 14096 (9621.7-21867.2) | 58.7 (37.1-95.6) | 10845.6 (6871.5-16606.6) | 25.1 (15.2-38.7) | -1.42 (-3.2-0.39) |
| Acute hepatitis A | Republic of Kiribati | 160.7 (115.1-226) | 201.9 (147.8-281) | 89.6 (58.2-126.3) | 71 (46.9-99.2) | -2.5 (-5-0.07) |
| Acute hepatitis A | Republic of Korea | 2829.6 (2070.5-3736) | 6.7 (4.9-9) | 1354.6 (919.8-1891.1) | 3 (2-4.2) | -2.61 (-4.08--1.12) |
| Acute hepatitis A | Republic of Latvia | 301.6 (245.1-376.2) | 12.9 (10.5-16) | 70.9 (53.5-93.6) | 4.7 (3.5-6.3) | -3.76 (-5.79--1.69) |
| Acute hepatitis A | Republic of Liberia | 1283.8 (741.6-2016.6) | 54.2 (33.3-88.7) | 724.1 (419.8-1273.3) | 15.7 (8.8-28.1) | -3.66 (-5.67--1.6) |
| Acute hepatitis A | Republic of Lithuania | 184.4 (138.6-243.1) | 5.5 (4.2-7.3) | 67.9 (45.6-99) | 3.4 (2.3-4.9) | -2.52 (-4.01--1) |
| Acute hepatitis A | Republic of Madagascar | 7283.1 (4267.7-11373.8) | 53.4 (31.6-92.5) | 4258.1 (2023.8-7998.9) | 15 (7-29.4) | -2.64 (-4.58--0.66) |
| Acute hepatitis A | Republic of Malawi | 11916 (6817.1-20133.5) | 95.8 (49.8-183.9) | 4894.8 (2314.2-9570.8) | 26.6 (11.6-55.2) | -2.82 (-5.17--0.41) |
| Acute hepatitis A | Republic of Maldives | 65.9 (37.8-97.9) | 26.8 (14.6-41) | 20.6 (14.6-29) | 4 (2.9-5.7) | -4.76 (-6.13--3.36) |
| Acute hepatitis A | Republic of Mali | 1469.2 (786-3299.2) | 17 (8.6-37.3) | 2010.9 (1202.9-3340.3) | 9.1 (4.9-16.8) | -0.87 (-2.31-0.59) |
| Acute hepatitis A | Republic of Malta | 11 (7.5-15.6) | 3 (2-4.3) | 11.3 (7.7-15.6) | 2.7 (1.8-3.8) | -0.47 (-1.53-0.61) |
| Acute hepatitis A | Republic of Mauritius | 109.8 (88.2-134.4) | 10.5 (8.5-12.9) | 36.6 (24.7-52.1) | 3.3 (2.2-4.8) | -2.61 (-3.76--1.44) |
| Acute hepatitis A | Republic of Moldova | 225.3 (154-321.1) | 5.2 (3.5-7.4) | 99.8 (67.8-145.2) | 3.6 (2.4-5.2) | -2.97 (-4.83--1.07) |
| Acute hepatitis A | Republic of Mozambique | 19303.3 (9064.4-36557.2) | 108.9 (52.9-230) | 5729.2 (2676.6-11513) | 18.3 (7.4-39) | -4.32 (-6.48--2.11) |
| Acute hepatitis A | Republic of Namibia | 315.8 (203.9-492.4) | 23.3 (13.8-40.1) | 180.7 (122.7-254.1) | 7.5 (5.1-10.7) | -2.61 (-4.62--0.56) |
| Acute hepatitis A | Republic of Nauru | 1.6 (1.1-2.3) | 14.7 (9.8-21.7) | 0.8 (0.6-1.1) | 6.7 (4.7-9.4) | -2.81 (-4.39--1.2) |
| Acute hepatitis A | Republic of Nicaragua | 996.9 (686.1-1420.1) | 16.8 (12-23.3) | 307.6 (221.2-424.9) | 4.4 (3.2-6) | -2.27 (-3.67--0.84) |
| Acute hepatitis A | Republic of Niue | 0.2 (0.1-0.3) | 7.9 (5.6-11.4) | 0.1 (0.1-0.1) | 5.9 (4.2-8.3) | -1.96 (-3.31--0.6) |
| Acute hepatitis A | Republic of Palau | 5.8 (3.8-8.5) | 39.1 (25.6-57.9) | 3 (2.1-4.2) | 15.5 (11.2-20.9) | -2.16 (-4.22--0.06) |
| Acute hepatitis A | Republic of Panama | 166.8 (128.7-216.6) | 6.2 (4.8-7.9) | 178.4 (125.7-243.9) | 4.2 (3-5.8) | -0.6 (-1.91-0.72) |
| Acute hepatitis A | Republic of Paraguay | 291.7 (210.9-420.5) | 6 (4.3-8.7) | 283.6 (190.8-405.3) | 3.8 (2.5-5.4) | -0.9 (-2.37-0.59) |
| Acute hepatitis A | Republic of Peru | 2462.1 (1862.8-3166.8) | 9.5 (7.2-12) | 1585.7 (1141.1-2175.2) | 4.4 (3.2-6.1) | -1.64 (-3--0.26) |
| Acute hepatitis A | Republic of Poland | 1275.7 (848.1-1806.2) | 3.6 (2.4-5) | 997.2 (674-1419.3) | 3.3 (2.2-4.7) | -0.65 (-1.72-0.43) |
| Acute hepatitis A | Republic of Rwanda | 9949.1 (6179.9-14880.4) | 132.1 (78-210.1) | 3003.4 (1585.7-5338.6) | 24.2 (11.7-44.4) | -4.62 (-6.71--2.49) |
| Acute hepatitis A | Republic of San Marino | 4.6 (3.5-5.9) | 20.3 (14.9-26.9) | 2.3 (1.6-3.3) | 5.9 (4.2-8.4) | -2.72 (-4.15--1.27) |
| Acute hepatitis A | Republic of Senegal | 1441.7 (819.9-2613.5) | 21.5 (12.4-40) | 1036.5 (688-1456.5) | 7 (4.4-10.2) | -2.58 (-4.34--0.79) |
| Acute hepatitis A | Republic of Serbia | 339.6 (226-492.1) | 3.9 (2.6-5.7) | 242.4 (159.2-348.9) | 3.5 (2.3-5.1) | -1.11 (-2.42-0.22) |
| Acute hepatitis A | Republic of Seychelles | 21.4 (12.9-33.1) | 33.5 (19.2-53.1) | 9.6 (6-16) | 8.5 (5.5-13.6) | -3.46 (-4.82--2.07) |
| Acute hepatitis A | Republic of Sierra Leone | 3773 (1801.2-6674.3) | 90.1 (45.9-152.1) | 1212.8 (781.3-1926.7) | 16.4 (10.2-27.2) | -4.78 (-6.81--2.7) |
| Acute hepatitis A | Republic of Singapore | 90 (61.9-126.9) | 2.8 (2-4) | 132.4 (86.6-191.6) | 2.4 (1.5-3.6) | -0.89 (-2.03-0.25) |
| Acute hepatitis A | Republic of Slovenia | 75.5 (53.8-102.3) | 4.2 (3-5.6) | 48.4 (31.8-69.5) | 3.2 (2.1-4.6) | -0.6 (-1.74-0.55) |
| Acute hepatitis A | Republic of South Africa | 4827.6 (3813.5-6329) | 11.8 (9.4-15.6) | 2691.8 (2062.3-3493.3) | 4.9 (3.8-6.4) | -1.51 (-3.33-0.33) |
| Acute hepatitis A | Republic of South Sudan | 4178.7 (2280.9-6898) | 59.6 (30-107.6) | 4640.2 (2023-10204) | 46.1 (17.9-116.6) | 0.18 (-1.7-2.1) |
| Acute hepatitis A | Republic of Sudan | 88853.7 (49702.1-133979) | 372.6 (188.8-672.2) | 17958.3 (7164.1-35160.3) | 47.5 (16.9-98.3) | -5.56 (-8.19--2.87) |
| Acute hepatitis A | Republic of Suriname | 26.5 (19.4-35.2) | 6.1 (4.5-8.1) | 21.5 (14.8-30.2) | 3.9 (2.7-5.4) | -1.26 (-2.59-0.09) |
| Acute hepatitis A | Republic of Tajikistan | 4470.5 (1956.6-7778.4) | 53.5 (24.9-89.8) | 1098.4 (629.1-2070.8) | 9.3 (5.4-16.8) | -3.52 (-6.25--0.72) |
| Acute hepatitis A | Republic of the Congo | 2904.1 (544.9-8987.4) | 92.7 (17.7-300.8) | 570.6 (252-1255.5) | 10.3 (4.2-23.5) | -5.4 (-7.71--3.03) |
| Acute hepatitis A | Republic of the Gambia | 130.9 (72.4-235.1) | 15.2 (7.9-28.7) | 140.3 (86.5-221.2) | 5.9 (3.5-10) | -2.28 (-3.69--0.85) |
| Acute hepatitis A | Republic of the Marshall Islands | 8.7 (4.7-13.8) | 18.6 (9.6-29.8) | 4 (2.8-5.6) | 6.8 (4.8-9.6) | -2.28 (-3.86--0.66) |
| Acute hepatitis A | Republic of the Niger | 7681.5 (3385.1-15135.4) | 95.9 (50.5-181.5) | 4049.8 (2226.7-7686.3) | 21.1 (10.5-39.8) | -3.76 (-5.72--1.76) |
| Acute hepatitis A | Republic of the Philippines | 35129.6 (25805.1-45060.2) | 49.8 (35.9-63.7) | 5964 (4535-7787) | 5.1 (3.9-6.6) | -6.29 (-7.94--4.6) |
| Acute hepatitis A | Republic of the Union of Myanmar | 17383.2 (8247.9-31430) | 44 (20-76.3) | 6876.3 (4023.6-10371.2) | 12.3 (7.2-18.7) | -3.25 (-4.82--1.65) |
| Acute hepatitis A | Republic of Trinidad and Tobago | 97 (72.9-122.2) | 7.6 (5.8-9.6) | 55 (39.2-74.6) | 4.5 (3.2-6) | -1.02 (-2.41-0.39) |
| Acute hepatitis A | Republic of Tunisia | 1964.5 (1117.7-3586.1) | 22.4 (12.7-41.3) | 569.8 (368-924.1) | 5 (3.3-7.9) | -2.9 (-4.97--0.79) |
| Acute hepatitis A | Republic of Turkey | 26689.2 (16878.6-40442.2) | 41.9 (26.7-63.1) | 3423.4 (2456-4785.4) | 4.3 (3-6) | -5.64 (-7.36--3.89) |
| Acute hepatitis A | Republic of Uganda | 16779.1 (7573-31437.5) | 76.7 (36.8-158.6) | 9964.7 (4889-22327.3) | 23.7 (10.8-54.3) | -3.92 (-5.86--1.95) |
| Acute hepatitis A | Republic of Uzbekistan | 43893.8 (34352.9-56603.4) | 150 (118.7-193.6) | 2504.8 (1926.2-3187.2) | 7.2 (5.5-9.1) | -5.74 (-8.38--3.02) |
| Acute hepatitis A | Republic of Vanuatu | 41.5 (16.6-86.3) | 25.4 (9.3-58.6) | 32.8 (17.6-54.6) | 9.9 (5.1-17.1) | -3.57 (-5.15--1.96) |
| Acute hepatitis A | Republic of Yemen | 61208.5 (28935.7-111083.4) | 346.7 (137-744.1) | 10661.6 (5382.9-19824.2) | 36.3 (16.1-70.9) | -6.83 (-9.44--4.15) |
| Acute hepatitis A | Republic of Zambia | 10359.6 (5556.8-17930.4) | 109.7 (54.1-200.3) | 3683.5 (2096.4-6076.6) | 19.7 (10.4-33.2) | -4.32 (-6.62--1.97) |
| Acute hepatitis A | Republic of Zimbabwe | 4339.6 (1588.2-8793.3) | 45.2 (15.5-86.2) | 5097.6 (2469.3-8989.2) | 37.1 (17.4-67.2) | -0.1 (-1.26-1.08) |
| Acute hepatitis A | Romania | 990.5 (700.7-1349.3) | 4.8 (3.4-6.4) | 496.2 (333-714.3) | 3.3 (2.2-4.8) | -1.23 (-2.51-0.08) |
| Acute hepatitis A | Russian Federation | 5338.8 (3743.7-7537.3) | 3.9 (2.7-5.5) | 3955.2 (2636.1-5757.9) | 3.5 (2.3-5.1) | -1.65 (-3.16--0.12) |
| Acute hepatitis A | Saint Kitts and Nevis | 3.5 (2.6-4.6) | 8 (6-10.4) | 1.9 (1.3-2.8) | 3.8 (2.5-5.4) | -1.94 (-3.25--0.61) |
| Acute hepatitis A | Saint Lucia | 10.2 (7.8-13.4) | 6.7 (5-8.7) | 5.6 (3.7-8) | 3.7 (2.4-5.2) | -1.62 (-2.88--0.34) |
| Acute hepatitis A | Saint Vincent and the Grenadines | 4.8 (3.1-7) | 3.6 (2.3-5.2) | 3.9 (2.5-5.5) | 3.7 (2.4-5.3) | -0.18 (-1.42-1.08) |
| Acute hepatitis A | Slovak Republic | 188.2 (126.7-266.7) | 3.7 (2.5-5.2) | 137 (91.8-196.7) | 3.2 (2.1-4.6) | -0.29 (-1.44-0.87) |
| Acute hepatitis A | Socialist Republic of Viet Nam | 34261.8 (21188.8-49766.9) | 61.8 (37-92.3) | 6405.7 (4438.4-8867.3) | 6.4 (4.5-8.8) | -5.62 (-7.25--3.97) |
| Acute hepatitis A | Solomon Islands | 198 (57.3-517.4) | 53.1 (14-147.6) | 95.9 (42.4-184.1) | 13.2 (5.6-26.1) | -4.63 (-6.36--2.87) |
| Acute hepatitis A | State of Eritrea | 2905.8 (1410.2-4645.4) | 85.9 (39.8-151.3) | 1907.5 (830-4049.3) | 32.2 (13.2-68) | -1.86 (-3.76-0.07) |
| Acute hepatitis A | State of Israel | 133.2 (86-193.8) | 2.7 (1.7-3.9) | 223.7 (143.7-331.5) | 2.4 (1.5-3.5) | -1.16 (-2.24--0.08) |
| Acute hepatitis A | State of Kuwait | 68.5 (45.5-98) | 3.8 (2.6-5.3) | 145.7 (95.5-209.5) | 3.4 (2.3-5) | -1.34 (-2.38--0.29) |
| Acute hepatitis A | State of Libya | 986.8 (468-1666.2) | 25.9 (11.3-43.9) | 391.8 (244.6-638.1) | 6 (3.8-9.9) | -2.73 (-4.88--0.54) |
| Acute hepatitis A | State of Qatar | 46 (33.9-60.7) | 17 (12.3-23.1) | 116.2 (78.8-161.6) | 4.8 (3.5-6.7) | -3.95 (-5.51--2.35) |
| Acute hepatitis A | Sultanate of Oman | 2998.9 (1971.2-4141.2) | 234.7 (138.4-339.2) | 992.4 (769.5-1279.2) | 31.1 (24.1-41.1) | -2.83 (-5.59-0.01) |
| Acute hepatitis A | Swiss Confederation | 173 (126.5-232) | 2.6 (1.9-3.4) | 199.7 (140.1-273.1) | 2.2 (1.5-3) | -1.38 (-2.32--0.43) |
| Acute hepatitis A | Syrian Arab Republic | 5529.7 (4096.4-7335.5) | 47.5 (33.9-65.1) | 1084.6 (795.2-1449.1) | 7.6 (5.6-10.1) | -3.7 (-5.47--1.9) |
| Acute hepatitis A | Taiwan (Province of China) | 724.9 (472.3-1053.5) | 3.3 (2.2-4.8) | 579.2 (378.5-841.5) | 3.1 (2-4.5) | -1.25 (-2.51-0.02) |
| Acute hepatitis A | Togolese Republic | 490.6 (277.6-846.2) | 14.8 (8.2-27.1) | 589.1 (367-931.2) | 7.3 (4.3-12.1) | -1.25 (-2.83-0.35) |
| Acute hepatitis A | Tokelau | 0.2 (0.1-0.3) | 12.1 (7-19.5) | 0.1 (0.1-0.2) | 7.9 (5.3-11.6) | -2.21 (-3.76--0.64) |
| Acute hepatitis A | Turkmenistan | 6256.1 (3876.1-8978.7) | 119.5 (76.7-170) | 561.5 (369.3-872.1) | 10.5 (6.9-16.4) | -5.13 (-7.34--2.87) |
| Acute hepatitis A | Tuvalu | 2.2 (1.1-3.6) | 21.8 (11.2-35.7) | 0.7 (0.5-1) | 5.9 (4.2-8.1) | -3.29 (-4.83--1.73) |
| Acute hepatitis A | Ukraine | 1813.8 (1247.3-2591.7) | 4 (2.7-5.7) | 1113.5 (736.5-1662.1) | 3.4 (2.2-4.9) | -2 (-3.51--0.47) |
| Acute hepatitis A | Union of the Comoros | 222 (107.5-381.4) | 45 (23.4-79.6) | 136.9 (65.3-244.5) | 19.2 (8.9-36) | -1.83 (-3.64-0.01) |
| Acute hepatitis A | United Arab Emirates | 197.4 (127.5-310.7) | 17.4 (10-29.1) | 484.5 (357.7-654) | 6.8 (5.1-9) | -1.8 (-3.51--0.05) |
| Acute hepatitis A | United Kingdom of Great Britain and Northern Ireland | 1369 (892.6-1962.3) | 2.5 (1.6-3.6) | 1509.2 (984.7-2174.8) | 2.4 (1.5-3.4) | 0.15 (-0.8-1.11) |
| Acute hepatitis A | United Mexican States | 15442.8 (13359.5-18012.2) | 14.5 (12.6-16.8) | 6827.7 (5387-8821.4) | 5.6 (4.5-7.3) | -1.53 (-2.95--0.08) |
| Acute hepatitis A | United Republic of Tanzania | 25424.8 (13922.7-42689) | 78.5 (45.7-139) | 11172.6 (6320.5-19762.5) | 18.4 (10.3-33.1) | -3.52 (-5.36--1.64) |
| Acute hepatitis A | United States of America | 8200.7 (5977.9-10891.3) | 3.2 (2.3-4.3) | 10448.2 (7728.4-13891) | 3.2 (2.3-4.4) | -1.11 (-2.15--0.07) |
| Acute hepatitis A | United States Virgin Islands | 4.6 (3.2-6.6) | 4.2 (3-6) | 2.3 (1.4-3.3) | 3.6 (2.3-5.3) | -0.5 (-1.57-0.57) |
| Acute hepatitis B | American Samoa | 3.3 (2.5-4.4) | 7.9 (6-10.3) | 2.8 (2.1-3.8) | 5.5 (4.2-7.4) | -1.11 (-2.46-0.26) |
| Acute hepatitis B | Antigua and Barbuda | 1 (0.7-1.4) | 1.8 (1.3-2.3) | 0.8 (0.6-1.1) | 0.8 (0.6-1.1) | -0.78 (-1.93-0.39) |
| Acute hepatitis B | Arab Republic of Egypt | 118446.4 (46923.7-199527) | 296.3 (114.7-508.4) | 73562.5 (28855.7-124011.1) | 91.8 (35.5-155.4) | -4.87 (-7.61--2.04) |
| Acute hepatitis B | Argentine Republic | 578 (388.4-1021.8) | 1.8 (1.2-3.1) | 322.9 (244.7-430.8) | 0.7 (0.5-0.9) | -1.42 (-2.73--0.08) |
| Acute hepatitis B | Australia | 229.8 (142.6-351.3) | 1.3 (0.8-2) | 355.4 (247.1-534.8) | 1.2 (0.8-1.8) | -0.4 (-1.37-0.58) |
| Acute hepatitis B | Barbados | 8.1 (5.7-11) | 3.3 (2.4-4.6) | 10.8 (8.2-14.1) | 3.6 (2.6-4.7) | -0.66 (-2.13-0.83) |
| Acute hepatitis B | Belize | 4.3 (3-6.1) | 2.2 (1.6-3.1) | 4.3 (3.2-5.8) | 1 (0.8-1.4) | -1.17 (-2.37-0.05) |
| Acute hepatitis B | Bermuda | 0.5 (0.3-0.7) | 0.8 (0.5-1.1) | 0.4 (0.2-0.5) | 0.5 (0.3-0.7) | -0.29 (-1.41-0.85) |
| Acute hepatitis B | Bolivarian Republic of Venezuela | 184 (123.3-264) | 0.9 (0.6-1.3) | 458.7 (315.3-684.3) | 1.8 (1.2-2.8) | -0.07 (-1.41-1.3) |
| Acute hepatitis B | Bosnia and Herzegovina | 361.6 (208.8-593.9) | 8.2 (4.8-13.7) | 153.3 (98-224.2) | 4 (2.5-5.8) | -1.1 (-2.62-0.45) |
| Acute hepatitis B | Brunei Darussalam | 174 (125.3-235.4) | 79.1 (56.8-106) | 54.7 (41.4-69.7) | 12 (9-15.5) | -5.38 (-7.2--3.54) |
| Acute hepatitis B | Burkina Faso | 13007.8 (7249.1-19979.6) | 125.5 (75.7-183.4) | 14746.9 (8818.7-24155.9) | 67.9 (43.6-102.3) | -2.85 (-4.9--0.76) |
| Acute hepatitis B | Canada | 440.2 (323.5-598) | 1.5 (1.1-2.1) | 396.5 (268.4-592.9) | 0.9 (0.6-1.4) | -0.73 (-1.63-0.18) |
| Acute hepatitis B | Central African Republic | 5957.4 (1709.7-16312) | 197.2 (67.5-436) | 3557.5 (1660.9-6759.7) | 70.3 (34.5-129.7) | -3.15 (-5.29--0.97) |
| Acute hepatitis B | Commonwealth of Dominica | 1.7 (1.1-2.5) | 2.4 (1.7-3.6) | 1.2 (0.9-1.7) | 2 (1.4-2.8) | -0.5 (-1.77-0.78) |
| Acute hepatitis B | Commonwealth of the Bahamas | 4.3 (3.2-5.6) | 1.7 (1.3-2.2) | 4.6 (3.3-6.3) | 1.1 (0.8-1.5) | -0.79 (-2.02-0.46) |
| Acute hepatitis B | Cook Islands | 0.5 (0.3-0.7) | 2.9 (1.9-4.3) | 0.3 (0.2-0.5) | 1.4 (0.9-2.2) | -0.64 (-1.78-0.53) |
| Acute hepatitis B | Czech Republic | 148.1 (112.4-196.7) | 1.4 (1.1-1.8) | 114.1 (86.4-154.9) | 0.9 (0.7-1.2) | -0.68 (-1.82-0.47) |
| Acute hepatitis B | Democratic People's Republic of Korea | 9690.3 (4804.9-16030.1) | 45.8 (22.7-76.3) | 2726.7 (1883.7-3806) | 8.9 (6.3-12.3) | -4.22 (-5.95--2.45) |
| Acute hepatitis B | Democratic Republic of Sao Tome and Principe | 61 (39.8-84.6) | 52.4 (33.9-74) | 24.8 (17.7-33.7) | 13.7 (10-18.4) | -4.3 (-6.24--2.33) |
| Acute hepatitis B | Democratic Republic of the Congo | 5872 (3228.2-10863.9) | 15.7 (8.6-28.1) | 8535.5 (4774.1-13285.6) | 11.3 (6.2-17.9) | -1.76 (-3.44--0.04) |
| Acute hepatitis B | Democratic Republic of Timor-Leste | 56.2 (19-117.8) | 8.3 (2.9-17.6) | 54.6 (24.5-111.2) | 4.6 (2-9.6) | -3.25 (-4.62--1.85) |
| Acute hepatitis B | Democratic Socialist Republic of Sri Lanka | 1565.1 (990.5-2381.3) | 10.7 (6.7-16.7) | 858.7 (586.8-1227.1) | 3.4 (2.3-4.8) | -3.58 (-4.86--2.28) |
| Acute hepatitis B | Dominican Republic | 176.5 (121.7-254.1) | 2.2 (1.6-3.1) | 289.3 (197.6-409.6) | 2.7 (1.8-3.8) | -0.8 (-2.01-0.42) |
| Acute hepatitis B | Eastern Republic of Uruguay | 17.7 (13-23.5) | 0.6 (0.4-0.7) | 12.9 (8.8-18.6) | 0.3 (0.2-0.5) | -0.65 (-1.9-0.61) |
| Acute hepatitis B | Federal Democratic Republic of Ethiopia | 53336.9 (15567.4-98967) | 153.2 (44.9-273.2) | 75462.9 (29131.5-114940.7) | 91.4 (36.2-139.5) | -3.02 (-4.68--1.32) |
| Acute hepatitis B | Federal Democratic Republic of Nepal | 4245.5 (797.1-13362.2) | 21.5 (4.1-63.3) | 8144.5 (2655.8-17353.4) | 26.7 (8.6-56.6) | -2.12 (-4.38-0.2) |
| Acute hepatitis B | Federal Republic of Germany | 1732.9 (1425.3-2056.6) | 1.9 (1.6-2.2) | 448.2 (333.3-580.5) | 0.4 (0.3-0.5) | -0.79 (-1.71-0.14) |
| Acute hepatitis B | Federal Republic of Nigeria | 463151.3 (189116.3-819473.4) | 459.6 (194.1-839.7) | 161295.7 (85908.9-256623.7) | 68 (36.5-109.5) | -7.86 (-9.86--5.81) |
| Acute hepatitis B | Federal Republic of Somalia | 28129.7 (11129.3-49545.5) | 507.8 (201.2-868) | 68525.8 (26798.5-119566.6) | 468.1 (184.9-785.7) | -0.93 (-3.03-1.22) |
| Acute hepatitis B | Federated States of Micronesia | 22.6 (12.2-36.4) | 23.7 (13.4-37.4) | 6.4 (4-10) | 6.5 (4-10.1) | -3.41 (-4.68--2.12) |
| Acute hepatitis B | Federative Republic of Brazil | 4775.6 (3924-6045.8) | 3.5 (2.8-4.4) | 4973.8 (4226.8-6057.4) | 2.1 (1.8-2.5) | -1.62 (-2.97--0.24) |
| Acute hepatitis B | French Republic | 635.9 (519.6-808.5) | 1 (0.8-1.3) | 470.5 (356-629.4) | 0.6 (0.4-0.8) | -0.65 (-1.69-0.41) |
| Acute hepatitis B | Gabonese Republic | 535.6 (271.1-1056) | 60.1 (31.8-126.2) | 138.8 (76-246.3) | 8.7 (4.8-15.6) | -4.25 (-6.04--2.43) |
| Acute hepatitis B | Georgia | 504.9 (334-694) | 10.1 (6.5-13.9) | 84.6 (65.2-108.1) | 2.3 (1.8-2.9) | -3.6 (-4.92--2.27) |
| Acute hepatitis B | Grand Duchy of Luxembourg | 3.1 (2.2-4.5) | 0.7 (0.5-1) | 3.4 (2.1-5.3) | 0.4 (0.3-0.7) | -0.4 (-1.3-0.5) |
| Acute hepatitis B | Greenland | 1.4 (1-2.1) | 2.6 (1.8-3.8) | 0.5 (0.3-0.8) | 0.9 (0.6-1.4) | -1.43 (-2.58--0.26) |
| Acute hepatitis B | Grenada | 5 (3.5-8.8) | 5.8 (4.1-10.4) | 2.7 (2.2-3.4) | 2.6 (2.1-3.3) | -1.48 (-2.82--0.12) |
| Acute hepatitis B | Guam | 7 (5.1-9.4) | 5.4 (4-7.2) | 8 (6.2-10.3) | 4.6 (3.5-5.9) | -0.4 (-1.74-0.97) |
| Acute hepatitis B | Hashemite Kingdom of Jordan | 789.4 (455.5-1211) | 29.5 (17.2-44.6) | 509.7 (353.6-734.4) | 5.1 (3.5-7.2) | -4.04 (-5.55--2.51) |
| Acute hepatitis B | Hellenic Republic | 423.1 (359.8-505.3) | 3.8 (3.2-4.5) | 318 (261.5-391.6) | 2.3 (1.9-2.8) | -0.6 (-1.95-0.76) |
| Acute hepatitis B | Hungary | 226 (177.6-275.7) | 2.1 (1.7-2.5) | 147 (106.7-199.9) | 1.3 (0.9-1.8) | -0.54 (-1.61-0.54) |
| Acute hepatitis B | Independent State of Papua New Guinea | 2230.1 (973.2-4453) | 59.9 (22.9-121.6) | 1381.9 (592.8-2706.1) | 14.3 (6-28.4) | -4.65 (-6.15--3.12) |
| Acute hepatitis B | Independent State of Samoa | 14.4 (10-19.6) | 9.8 (6.8-13.3) | 11.2 (7.6-15.2) | 5.8 (4-7.9) | -1.31 (-2.7-0.11) |
| Acute hepatitis B | Ireland | 14.9 (9-23.8) | 0.4 (0.3-0.7) | 18.4 (11.1-29.2) | 0.3 (0.2-0.5) | -0.45 (-1.45-0.56) |
| Acute hepatitis B | Islamic Republic of Afghanistan | 50560.5 (15837.3-87387.7) | 586 (198.1-955.6) | 40054.5 (20998.6-65306.6) | 225.1 (121.9-354) | -4.7 (-7.44--1.88) |
| Acute hepatitis B | Islamic Republic of Iran | 22649.4 (15427.1-31668.8) | 53 (36.6-75.4) | 14224.2 (12042.2-16801.9) | 16.3 (13.8-19.4) | -4.48 (-6.57--2.35) |
| Acute hepatitis B | Islamic Republic of Mauritania | 6562.1 (2169.3-13286.4) | 362.7 (115.6-741.9) | 1828.6 (1041.4-2883.1) | 53.2 (30.6-87.2) | -6.22 (-8.59--3.79) |
| Acute hepatitis B | Islamic Republic of Pakistan | 57463 (20774.1-127814.1) | 55.7 (19.8-126.4) | 122173.4 (60731.9-211406.3) | 59.5 (29.8-102.7) | -1.06 (-3.14-1.06) |
| Acute hepatitis B | Jamaica | 36.3 (26.4-49.8) | 1.6 (1.2-2.3) | 54.1 (40.5-70.6) | 1.9 (1.4-2.6) | -0.46 (-1.74-0.82) |
| Acute hepatitis B | Japan | 3448.5 (2564-4661.5) | 2.5 (1.8-3.3) | 3216.3 (2493.5-4241.2) | 1.9 (1.5-2.6) | -1.87 (-3.12--0.61) |
| Acute hepatitis B | Kingdom of Bahrain | 199.3 (136.9-272.1) | 71.2 (48.2-98.2) | 279.6 (175.6-426.3) | 21.8 (13.9-32) | -3.91 (-6.19--1.58) |
| Acute hepatitis B | Kingdom of Belgium | 176.7 (153.1-208.6) | 1.6 (1.4-1.9) | 99.6 (80.9-125.6) | 0.7 (0.6-0.9) | -0.83 (-1.84-0.2) |
| Acute hepatitis B | Kingdom of Bhutan | 213.3 (75.4-445.2) | 36.1 (13.7-78.3) | 198 (92.9-354.4) | 27.2 (12.6-47.2) | -3.14 (-5.15--1.09) |
| Acute hepatitis B | Kingdom of Cambodia | 9211.6 (4134.2-15891.8) | 127.6 (54.4-225.4) | 6342.7 (2711-10916) | 40.3 (17.1-68.7) | -5.97 (-7.83--4.07) |
| Acute hepatitis B | Kingdom of Denmark | 80.8 (67.3-96.8) | 1.4 (1.2-1.7) | 51.9 (38.4-69.5) | 0.7 (0.5-1) | -0.64 (-1.57-0.3) |
| Acute hepatitis B | Kingdom of Eswatini | 444.3 (192.5-769.4) | 95.4 (42.1-160.6) | 320.7 (186.6-515.2) | 36.7 (22.1-57.7) | -2.9 (-5.1--0.65) |
| Acute hepatitis B | Kingdom of Lesotho | 1064.1 (472.9-1820.2) | 100.6 (45.2-171.1) | 902.8 (561.4-1389.1) | 62.2 (39.7-94.9) | -1.2 (-3.49-1.14) |
| Acute hepatitis B | Kingdom of Morocco | 6832.7 (3587.3-11116.4) | 36 (17.9-59.5) | 7665.3 (4998.8-12019.6) | 20.7 (13.4-31.9) | -2.98 (-5.08--0.84) |
| Acute hepatitis B | Kingdom of Norway | 20.4 (13.7-30.4) | 0.5 (0.3-0.7) | 30.1 (23.6-40.2) | 0.5 (0.4-0.6) | -0.07 (-1-0.87) |
| Acute hepatitis B | Kingdom of Saudi Arabia | 14295.5 (6905-26673.3) | 143.9 (65.7-282.4) | 4599.3 (3338.3-6502.4) | 13.5 (9.8-18.9) | -6.34 (-8.21--4.44) |
| Acute hepatitis B | Kingdom of Spain | 594.8 (370.3-872.7) | 1.5 (0.9-2.1) | 538.9 (320.6-855.9) | 0.8 (0.5-1.3) | -0.43 (-1.4-0.55) |
| Acute hepatitis B | Kingdom of Sweden | 26.2 (16.4-40.8) | 0.3 (0.2-0.4) | 46.6 (33.8-64.7) | 0.4 (0.3-0.5) | 0.01 (-0.78-0.81) |
| Acute hepatitis B | Kingdom of Thailand | 13343.6 (9771-17430.2) | 26.2 (19.5-34.1) | 6950.6 (5424.1-8834.6) | 8.2 (6.6-10.4) | -1.49 (-3.31-0.36) |
| Acute hepatitis B | Kingdom of the Netherlands | 81 (63.3-105.4) | 0.5 (0.4-0.6) | 63 (43.7-89.9) | 0.3 (0.2-0.4) | -0.3 (-1.21-0.61) |
| Acute hepatitis B | Kingdom of Tonga | 249.7 (151.2-350.9) | 294.1 (176.2-412.2) | 69.4 (39.5-111.8) | 71.3 (41.5-114.3) | -4.2 (-5.89--2.48) |
| Acute hepatitis B | Kyrgyz Republic | 3915.4 (2935.5-5206.7) | 74.8 (57-99) | 474.8 (325.2-684.6) | 6.8 (4.6-9.8) | -5.4 (-7.61--3.14) |
| Acute hepatitis B | Lao People's Democratic Republic | 2660.3 (957.8-5649.6) | 73.7 (29.5-140) | 1252.4 (734.6-2123.7) | 18.4 (10.7-31.3) | -6.46 (-8.22--4.67) |
| Acute hepatitis B | Lebanese Republic | 248.2 (134.5-395.4) | 9.6 (5.2-15.1) | 289 (220-362.3) | 4.9 (3.8-6.2) | -2.48 (-3.92--1.01) |
| Acute hepatitis B | Malaysia | 6854.6 (5289.2-8797.8) | 49.9 (38.7-63.6) | 7621.9 (6164.4-9404.3) | 24 (19.4-30) | -1.5 (-3.86-0.93) |
| Acute hepatitis B | Mongolia | 2040.2 (988.2-3859.7) | 77.4 (40.7-140) | 374.2 (263.1-510.1) | 11.1 (7.8-14.9) | -4.03 (-6.36--1.65) |
| Acute hepatitis B | Montenegro | 4.9 (3.2-7.3) | 0.8 (0.5-1.2) | 3.7 (2.4-5.3) | 0.5 (0.4-0.8) | -0.27 (-1.33-0.81) |
| Acute hepatitis B | New Zealand | 59.2 (43.1-84) | 1.7 (1.2-2.4) | 41.6 (23.7-67.1) | 0.7 (0.4-1.1) | -0.53 (-1.49-0.43) |
| Acute hepatitis B | North Macedonia | 63.6 (37.9-101.7) | 3.5 (2-5.7) | 46.3 (29-73.1) | 2 (1.1-3.4) | -1.2 (-2.42-0.04) |
| Acute hepatitis B | Northern Mariana Islands | 5.8 (4.2-7.7) | 13.7 (10.1-17.8) | 4.3 (3.4-5.5) | 7.8 (6.1-9.8) | -1.28 (-2.82-0.3) |
| Acute hepatitis B | Palestine | 367.3 (213-583.1) | 25.7 (14.3-41.8) | 236.3 (181-302.8) | 6.7 (5.2-8.5) | -3.55 (-5.1--1.98) |
| Acute hepatitis B | People's Democratic Republic of Algeria | 9747.5 (6013-14626.7) | 47.9 (28.8-67.6) | 7854.7 (4904.5-11370.4) | 19.1 (11.9-27.6) | -2.98 (-5.09--0.83) |
| Acute hepatitis B | People's Republic of Bangladesh | 41034.2 (10981-87988.5) | 52.4 (13.4-111.6) | 45592.2 (19189.5-76733.6) | 28.6 (12-48.6) | -3.42 (-5.51--1.28) |
| Acute hepatitis B | People's Republic of China | 802752.4 (555930.3-1058589.2) | 74.9 (52-98.7) | 114254 (87683.9-146707) | 6.8 (5.3-8.6) | -7.21 (-8.96--5.43) |
| Acute hepatitis B | Plurinational State of Bolivia | 148.5 (75-303.6) | 2.1 (1.2-3.8) | 214.3 (142.6-309) | 1.9 (1.3-2.7) | -1.34 (-2.89-0.24) |
| Acute hepatitis B | Portuguese Republic | 395.5 (350.3-449.7) | 4 (3.5-4.5) | 204.8 (168.2-248.8) | 1.4 (1.2-1.7) | -1.48 (-2.75--0.19) |
| Acute hepatitis B | Principality of Andorra | 5.1 (2.8-8.4) | 11.1 (6.1-18.1) | 8 (4.5-12.6) | 7.2 (4.2-11.5) | -2.52 (-3.86--1.16) |
| Acute hepatitis B | Principality of Monaco | 0.4 (0.2-0.5) | 1 (0.7-1.3) | 0.4 (0.3-0.5) | 0.8 (0.5-1.1) | -0.14 (-1.06-0.78) |
| Acute hepatitis B | Puerto Rico | 26 (17.3-37.6) | 0.7 (0.5-1) | 37.3 (29.3-47.2) | 1.1 (0.9-1.3) | -1.18 (-2.4-0.05) |
| Acute hepatitis B | Republic of Albania | 145.3 (90.2-234.1) | 4.1 (2.6-6.5) | 58.7 (35.1-98.6) | 2.4 (1.3-4.7) | -1.88 (-3.19--0.56) |
| Acute hepatitis B | Republic of Angola | 42573.3 (9198.7-111541.1) | 390.4 (92.1-829.2) | 15683.7 (8360.9-29354.1) | 53.8 (31.6-83.3) | -7.15 (-9.33--4.91) |
| Acute hepatitis B | Republic of Armenia | 748.1 (538.6-1026.6) | 20.9 (15.1-28.6) | 69.8 (50.4-96) | 2.2 (1.6-3.1) | -2.86 (-4.35--1.35) |
| Acute hepatitis B | Republic of Austria | 72.4 (55.2-99.8) | 0.9 (0.7-1.2) | 51.8 (33.6-79) | 0.5 (0.3-0.7) | -0.45 (-1.42-0.53) |
| Acute hepatitis B | Republic of Azerbaijan | 3860.8 (2655-5391.1) | 47 (32.4-65.7) | 875.9 (551.6-1358.4) | 9 (5.5-14.4) | -4.15 (-5.97--2.3) |
| Acute hepatitis B | Republic of Belarus | 1478.2 (1228.2-1818) | 17.5 (14.2-21.9) | 499.3 (359.1-641.7) | 6.9 (4.6-9.2) | -1.75 (-3.44--0.02) |
| Acute hepatitis B | Republic of Benin | 4237.6 (2926.4-6172.8) | 84.1 (60.5-117) | 2832.1 (1834.1-4198.8) | 25.2 (16.1-37) | -4.25 (-6.18--2.29) |
| Acute hepatitis B | Republic of Botswana | 462.3 (217.2-802) | 56.3 (27.7-98.2) | 309.6 (170.6-475.6) | 14.4 (8.3-21.8) | -3.58 (-5.49--1.64) |
| Acute hepatitis B | Republic of Bulgaria | 274.3 (201.6-372.3) | 3.3 (2.4-4.4) | 220.6 (159.3-296) | 2.9 (2.1-3.8) | -0.77 (-2.1-0.58) |
| Acute hepatitis B | Republic of Burundi | 2463.7 (357.8-5737.6) | 58.4 (7.8-133.4) | 3961 (979-8540.9) | 41.6 (10.2-88.3) | -1.39 (-3.2-0.45) |
| Acute hepatitis B | Republic of Cabo Verde | 203.3 (160.8-255.1) | 69.4 (54.8-86.3) | 101.4 (76.6-135.2) | 18.4 (13.9-24.9) | -3.54 (-5.8--1.23) |
| Acute hepatitis B | Republic of Cameroon | 1566.1 (787.3-3413.8) | 16.7 (8.3-36.1) | 2293 (1444.4-3719.7) | 9 (5.7-14.5) | -2.22 (-3.74--0.67) |
| Acute hepatitis B | Republic of Chad | 9894.3 (6034.6-14950.6) | 163.7 (103.8-256.8) | 17162.2 (12236.9-24881.4) | 121.1 (81.2-182.3) | -1.78 (-3.87-0.36) |
| Acute hepatitis B | Republic of Chile | 83.9 (58.2-119.5) | 0.7 (0.5-0.9) | 126.7 (88.5-181.7) | 0.6 (0.4-0.8) | -0.93 (-2.17-0.33) |
| Acute hepatitis B | Republic of Colombia | 708.2 (491.9-1081.8) | 2.1 (1.5-3.3) | 798 (533.2-1218.8) | 1.6 (1.1-2.5) | -0.94 (-2.28-0.42) |
| Acute hepatitis B | Republic of Costa Rica | 27.3 (19.2-39) | 0.9 (0.6-1.3) | 35.6 (25.9-49.2) | 0.7 (0.5-1) | -0.58 (-1.74-0.58) |
| Acute hepatitis B | Republic of C么te d'Ivoire | 9282.7 (5879.7-14024.6) | 83.3 (53.8-121.6) | 4094.6 (2706.9-5753.7) | 17.8 (11.8-25.3) | -4.04 (-5.92--2.12) |
| Acute hepatitis B | Republic of Croatia | 70.1 (47.7-106.8) | 1.3 (0.9-2) | 50.2 (32.5-74.5) | 0.9 (0.6-1.4) | -0.31 (-1.34-0.73) |
| Acute hepatitis B | Republic of Cuba | 160.2 (120.8-214.1) | 1.5 (1.1-2) | 88.1 (68.3-110.7) | 0.7 (0.5-0.8) | -1.07 (-2.33-0.21) |
| Acute hepatitis B | Republic of Cyprus | 15.3 (11.7-19.7) | 1.9 (1.5-2.5) | 15.1 (10.5-21.4) | 0.8 (0.6-1.2) | -0.74 (-1.73-0.26) |
| Acute hepatitis B | Republic of Djibouti | 190.9 (90-337.8) | 60.4 (27.4-103.6) | 502.3 (280.8-814.8) | 46.1 (26-74.4) | -1.51 (-3.35-0.37) |
| Acute hepatitis B | Republic of Ecuador | 120.8 (89.3-159.8) | 1.3 (0.9-1.7) | 190.2 (147.3-246.6) | 1.1 (0.8-1.4) | -1.19 (-2.53-0.18) |
| Acute hepatitis B | Republic of El Salvador | 190.6 (114.5-292.8) | 2.9 (1.9-4.2) | 101.1 (65.7-142.4) | 1.6 (1.1-2.3) | -1.31 (-2.47--0.14) |
| Acute hepatitis B | Republic of Equatorial Guinea | 16.6 (7.8-37.1) | 4.3 (2.2-9.4) | 36.1 (22.7-57.7) | 2.9 (1.8-4.6) | -2.15 (-3.4--0.88) |
| Acute hepatitis B | Republic of Estonia | 137.3 (116.2-160.4) | 10.9 (9.2-12.8) | 14 (10.8-18.3) | 1.2 (0.9-1.5) | -2.85 (-4.32--1.35) |
| Acute hepatitis B | Republic of Fiji | 119.4 (75-168.1) | 17 (10.6-23.5) | 39.1 (27.5-53.9) | 4.2 (3-5.8) | -3.25 (-4.66--1.81) |
| Acute hepatitis B | Republic of Finland | 50.5 (37.4-69.6) | 0.9 (0.7-1.2) | 33.7 (22.4-51.2) | 0.6 (0.4-0.9) | -0.38 (-1.17-0.42) |
| Acute hepatitis B | Republic of Ghana | 17509.9 (9873.9-26938.5) | 124.3 (71.6-185.6) | 3584 (2312-5561.9) | 12.1 (7.8-18.7) | -6.59 (-8.12--5.05) |
| Acute hepatitis B | Republic of Guatemala | 202.1 (121.2-315.5) | 2 (1.2-3) | 257.7 (181.3-364.8) | 1.8 (1.3-2.5) | -1.18 (-2.64-0.3) |
| Acute hepatitis B | Republic of Guinea | 13843 (7735.3-22728.5) | 209.4 (120.4-338.7) | 8652.1 (5572.2-13436.7) | 78.1 (51.5-119.4) | -3.49 (-5.58--1.35) |
| Acute hepatitis B | Republic of Guinea-Bissau | 1896.4 (1075.9-3099.9) | 200.1 (111-314.6) | 1065.2 (736.6-1476.2) | 66.7 (46.7-93.2) | -3.81 (-5.96--1.61) |
| Acute hepatitis B | Republic of Guyana | 11.7 (8.1-16.5) | 1.5 (1-2.1) | 30 (21.6-40.9) | 4 (2.9-5.4) | -0.97 (-2.37-0.46) |
| Acute hepatitis B | Republic of Haiti | 408.6 (148.1-1257.2) | 5.7 (2.5-13.7) | 807.1 (429.6-1539.6) | 6 (3.4-10.8) | -1.59 (-3.61-0.48) |
| Acute hepatitis B | Republic of Honduras | 319.4 (146.9-613.7) | 5.1 (2.6-9.2) | 310.2 (175.8-521.8) | 3.4 (2-5.6) | -2.55 (-4.06--1.02) |
| Acute hepatitis B | Republic of Iceland | 2 (1.5-2.6) | 0.8 (0.6-1) | 1.6 (1.1-2.3) | 0.4 (0.3-0.6) | -0.24 (-1.01-0.53) |
| Acute hepatitis B | Republic of India | 359372.2 (182710.3-683294.4) | 39.4 (20.5-71.8) | 694032.3 (387602.1-960674.1) | 54.1 (30.3-74.8) | -0.94 (-3.37-1.55) |
| Acute hepatitis B | Republic of Indonesia | 64807 (26944.7-113087.6) | 44.4 (18.5-79.8) | 88683.4 (45565.2-143874.6) | 30.4 (15.5-49.2) | -3.4 (-5.01--1.76) |
| Acute hepatitis B | Republic of Iraq | 4477.3 (2841.8-6654.6) | 31.8 (21-46.1) | 4969.8 (3303.6-6894) | 15 (10-20.8) | -2.73 (-5--0.4) |
| Acute hepatitis B | Republic of Italy | 1044.6 (686.2-1549.8) | 1.7 (1.2-2.5) | 1112.3 (882.1-1457.9) | 1.3 (1-1.7) | -0.39 (-1.54-0.78) |
| Acute hepatitis B | Republic of Kazakhstan | 9502.2 (7460.6-11916.8) | 54.5 (43-67.8) | 945.1 (681.2-1282.4) | 4.9 (3.5-6.6) | -5.4 (-7.07--3.69) |
| Acute hepatitis B | Republic of Kenya | 10445.9 (4101.9-15990.7) | 61.7 (21.2-93.3) | 16261.3 (7933.7-22940.4) | 41.6 (19.9-61.1) | -1.42 (-3.2-0.39) |
| Acute hepatitis B | Republic of Kiribati | 106.5 (74.3-148.8) | 146.5 (101.5-202.6) | 67.8 (41.5-105.1) | 57.3 (35.2-86.3) | -2.5 (-5-0.07) |
| Acute hepatitis B | Republic of Korea | 4776.4 (3681-6102.1) | 11.9 (9.2-15.1) | 1719 (1147.9-2433.3) | 2.2 (1.5-3.1) | -2.61 (-4.08--1.12) |
| Acute hepatitis B | Republic of Latvia | 976.7 (840.6-1118.7) | 44.8 (38.2-51.8) | 53.2 (42.3-65.7) | 3.6 (2.8-4.6) | -3.76 (-5.79--1.69) |
| Acute hepatitis B | Republic of Liberia | 2265.7 (1435.7-3160.7) | 86.4 (61.2-118) | 1872.7 (1207-2843) | 41.3 (26.8-61.6) | -3.66 (-5.67--1.6) |
| Acute hepatitis B | Republic of Lithuania | 391.9 (327.6-464) | 12.3 (10.2-14.8) | 39.1 (31.5-48.5) | 1.6 (1.3-2) | -2.52 (-4.01--1) |
| Acute hepatitis B | Republic of Madagascar | 6370 (3636.9-8959.9) | 68.5 (37.1-92.7) | 6613.4 (3298.7-10226.3) | 29.4 (15-45.9) | -2.64 (-4.58--0.66) |
| Acute hepatitis B | Republic of Malawi | 11798.9 (6261.6-18502.9) | 145.5 (87.2-212.7) | 13383 (8959.3-19858.4) | 91.5 (62.4-129.4) | -2.82 (-5.17--0.41) |
| Acute hepatitis B | Republic of Maldives | 39.3 (19.3-73.9) | 21.9 (11.6-37.8) | 18.9 (13.1-24.8) | 3.6 (2.6-4.7) | -4.76 (-6.13--3.36) |
| Acute hepatitis B | Republic of Mali | 995 (426.2-2321.4) | 12.3 (5.5-25.9) | 2135.6 (1073.6-3721.4) | 11.8 (5.7-20.3) | -0.87 (-2.31-0.59) |
| Acute hepatitis B | Republic of Malta | 3.1 (2.4-4) | 0.8 (0.6-1) | 2.4 (1.8-3.3) | 0.4 (0.3-0.6) | -0.47 (-1.53-0.61) |
| Acute hepatitis B | Republic of Mauritius | 23.3 (14.4-35.6) | 2.2 (1.3-3.3) | 20 (11.4-31.9) | 1.3 (0.8-2.1) | -2.61 (-3.76--1.44) |
| Acute hepatitis B | Republic of Moldova | 2019.9 (1645.9-2484.9) | 48.9 (39.7-60.4) | 165.7 (123.8-219.6) | 5.7 (4.2-7.4) | -2.97 (-4.83--1.07) |
| Acute hepatitis B | Republic of Mozambique | 22279.4 (9456.5-40789) | 192.9 (94.7-316.9) | 13693.4 (8848.7-20858.5) | 59.5 (38.8-90.3) | -4.32 (-6.48--2.11) |
| Acute hepatitis B | Republic of Namibia | 404.8 (193.8-669.8) | 44.8 (21.8-73.6) | 390.4 (225.7-629.6) | 20.1 (12-31.2) | -2.61 (-4.62--0.56) |
| Acute hepatitis B | Republic of Nauru | 1.9 (1.2-2.8) | 20.5 (13.5-30.7) | 0.7 (0.4-1) | 6.8 (4.4-10.1) | -2.81 (-4.39--1.2) |
| Acute hepatitis B | Republic of Nicaragua | 164.5 (84.1-327.4) | 3 (1.7-5.4) | 88.2 (53.5-133.3) | 1.4 (0.9-2.1) | -2.27 (-3.67--0.84) |
| Acute hepatitis B | Republic of Niue | 0.2 (0.2-0.3) | 10 (7-13.6) | 0.1 (0.1-0.1) | 4.5 (3-7) | -1.96 (-3.31--0.6) |
| Acute hepatitis B | Republic of Palau | 6 (4-8.7) | 41.7 (27.6-60.5) | 3.5 (2.4-4.7) | 16.6 (11.8-22.2) | -2.16 (-4.22--0.06) |
| Acute hepatitis B | Republic of Panama | 34.9 (24.9-48.8) | 1.4 (1-1.9) | 45.3 (34.2-59.2) | 1.2 (0.9-1.5) | -0.6 (-1.91-0.72) |
| Acute hepatitis B | Republic of Paraguay | 204.6 (120-331.7) | 5.1 (3-8.2) | 201 (144.5-267.5) | 2.9 (2.1-3.9) | -0.9 (-2.37-0.59) |
| Acute hepatitis B | Republic of Peru | 686.7 (457-1026.6) | 3 (2.1-4.3) | 589.7 (415.5-844.3) | 1.6 (1.1-2.3) | -1.64 (-3--0.26) |
| Acute hepatitis B | Republic of Poland | 431.3 (290.9-604.3) | 1.1 (0.7-1.5) | 242.9 (180.3-340.1) | 0.5 (0.4-0.7) | -0.65 (-1.72-0.43) |
| Acute hepatitis B | Republic of Rwanda | 6364.3 (3136.1-11382) | 117.4 (52.3-208.3) | 4653.8 (2396.9-6810) | 42.9 (21.1-61.7) | -4.62 (-6.71--2.49) |
| Acute hepatitis B | Republic of San Marino | 2.6 (1.8-3.6) | 10.5 (7.5-14.4) | 1.8 (1-2.9) | 4.3 (2.3-6.7) | -2.72 (-4.15--1.27) |
| Acute hepatitis B | Republic of Senegal | 2179.1 (1445.8-3283.4) | 29.1 (19.9-41.7) | 1758.2 (1122.9-2639.1) | 13.7 (8.8-20.5) | -2.58 (-4.34--0.79) |
| Acute hepatitis B | Republic of Serbia | 468.7 (317-664.6) | 5.4 (3.5-7.9) | 176.2 (128.3-234.9) | 1.7 (1.2-2.3) | -1.11 (-2.42-0.22) |
| Acute hepatitis B | Republic of Seychelles | 20.3 (11-36.3) | 33.3 (17.6-60.5) | 17 (10-26.7) | 13.6 (8.2-21.3) | -3.46 (-4.82--2.07) |
| Acute hepatitis B | Republic of Sierra Leone | 8381.5 (4163-14507.1) | 175.2 (90-290.4) | 2769.9 (1794.8-3911.7) | 35.8 (23-50.9) | -4.78 (-6.81--2.7) |
| Acute hepatitis B | Republic of Singapore | 82.1 (51.2-129.5) | 2.5 (1.6-3.9) | 130.2 (82.9-196) | 1.5 (1-2.3) | -0.89 (-2.03-0.25) |
| Acute hepatitis B | Republic of Slovenia | 46.8 (37.6-58.8) | 2.4 (1.9-3) | 27.6 (20.1-38.2) | 1.1 (0.8-1.5) | -0.6 (-1.74-0.55) |
| Acute hepatitis B | Republic of South Africa | 6295.5 (4353.6-8253.8) | 21.3 (14.5-28.1) | 5726.8 (4458.9-7228.8) | 10.3 (8-13) | -1.51 (-3.33-0.33) |
| Acute hepatitis B | Republic of South Sudan | 4136.2 (1909.6-7008.4) | 93.5 (45.3-153.3) | 7103.4 (3997.9-11314.8) | 99.6 (54.1-158.8) | 0.18 (-1.7-2.1) |
| Acute hepatitis B | Republic of Sudan | 49250.4 (17015.2-93575.5) | 335.6 (128.9-662) | 23835.6 (10888.2-42022.3) | 80.8 (35.6-143.2) | -5.56 (-8.19--2.87) |
| Acute hepatitis B | Republic of Suriname | 24.4 (17.2-34.6) | 6.2 (4.5-8.7) | 19.4 (14.2-26.4) | 3.5 (2.5-4.9) | -1.26 (-2.59-0.09) |
| Acute hepatitis B | Republic of Tajikistan | 13631.8 (9728-18346.3) | 179.3 (134.4-229.3) | 5949.7 (3008.5-12025.2) | 50.6 (26.2-99.4) | -3.52 (-6.25--0.72) |
| Acute hepatitis B | Republic of the Congo | 4856.9 (1183.9-12729.9) | 194.5 (53.8-446.1) | 1535.7 (560-3013.6) | 31.5 (11.5-60.8) | -5.4 (-7.71--3.03) |
| Acute hepatitis B | Republic of the Gambia | 131.1 (76.5-221.2) | 15.4 (9.1-25.5) | 123.2 (68.9-224.5) | 6.9 (3.9-12.3) | -2.28 (-3.69--0.85) |
| Acute hepatitis B | Republic of the Marshall Islands | 4.1 (2.5-5.8) | 10.8 (6.8-15) | 3.1 (1.8-4.5) | 5.6 (3.4-8.2) | -2.28 (-3.86--0.66) |
| Acute hepatitis B | Republic of the Niger | 15628 (7712.4-24844.7) | 171.5 (108.7-257.6) | 10322.1 (6351.1-16531.5) | 53.7 (31.9-86.9) | -3.76 (-5.72--1.76) |
| Acute hepatitis B | Republic of the Philippines | 34408.6 (22029.7-49810.9) | 55 (36.2-77.5) | 7668.8 (5964.7-9846.2) | 7.1 (5.5-9) | -6.29 (-7.94--4.6) |
| Acute hepatitis B | Republic of the Union of Myanmar | 3101.9 (756.5-6213.2) | 9.3 (2.3-19) | 3689.4 (1414.7-7122.2) | 6.6 (2.5-12.8) | -3.25 (-4.82--1.65) |
| Acute hepatitis B | Republic of Trinidad and Tobago | 38.7 (29.1-51.3) | 3.4 (2.5-4.5) | 43.7 (32.6-58) | 3.1 (2.3-4) | -1.02 (-2.41-0.39) |
| Acute hepatitis B | Republic of Tunisia | 3499.3 (1942.7-5713.2) | 48.6 (27.5-76.7) | 2072 (1308.5-3096.6) | 15.5 (9.8-23.2) | -2.9 (-4.97--0.79) |
| Acute hepatitis B | Republic of Turkey | 21450.3 (11653.2-36912.8) | 37 (21-60.8) | 5638.7 (4318.5-7132.4) | 6.2 (4.8-7.9) | -5.64 (-7.36--3.89) |
| Acute hepatitis B | Republic of Uganda | 22103.7 (10778.2-38259.9) | 170.9 (83.5-275.2) | 19711.8 (11468.6-31315) | 63.7 (37.2-99) | -3.92 (-5.86--1.95) |
| Acute hepatitis B | Republic of Uzbekistan | 42200.5 (31137.8-54640.8) | 152.8 (114.4-193.5) | 4295.4 (3362.7-5407.8) | 12.1 (9.5-15.3) | -5.74 (-8.38--3.02) |
| Acute hepatitis B | Republic of Vanuatu | 44.8 (21.4-79.8) | 34.5 (16.2-60.8) | 25.5 (13.7-44) | 9.2 (4.9-15.8) | -3.57 (-5.15--1.96) |
| Acute hepatitis B | Republic of Yemen | 40125.4 (9188-98771.7) | 409.7 (127.6-869) | 17915.6 (8730.1-30034.6) | 81.7 (39.6-137) | -6.83 (-9.44--4.15) |
| Acute hepatitis B | Republic of Zambia | 12435 (6147.7-20064.9) | 191.2 (105.7-296.7) | 8839.9 (5613.5-12638.1) | 59.7 (38.8-85.1) | -4.32 (-6.62--1.97) |
| Acute hepatitis B | Republic of Zimbabwe | 4504.4 (1294.6-8471) | 74.3 (19.9-143.8) | 4141.6 (1936-8896.1) | 36.8 (17.7-79.6) | -0.1 (-1.26-1.08) |
| Acute hepatitis B | Romania | 1059.2 (783.7-1431.9) | 4.9 (3.6-6.7) | 417 (286.7-625.5) | 1.9 (1.3-2.8) | -1.23 (-2.51-0.08) |
| Acute hepatitis B | Russian Federation | 10175.2 (9020.9-11208.9) | 8.5 (7.6-9.3) | 2198.4 (1842.4-2703.1) | 2 (1.7-2.3) | -1.65 (-3.16--0.12) |
| Acute hepatitis B | Saint Kitts and Nevis | 2.3 (1.6-3.3) | 5.8 (4.1-8.3) | 1.4 (1.1-1.7) | 2.3 (1.9-2.9) | -1.94 (-3.25--0.61) |
| Acute hepatitis B | Saint Lucia | 5.8 (4.5-8) | 4.6 (3.5-6.2) | 3.2 (2.5-4.2) | 1.7 (1.4-2.2) | -1.62 (-2.88--0.34) |
| Acute hepatitis B | Saint Vincent and the Grenadines | 0.9 (0.6-1.3) | 0.9 (0.6-1.3) | 1.6 (1.3-2.1) | 1.4 (1.1-1.8) | -0.18 (-1.42-1.08) |
| Acute hepatitis B | Slovak Republic | 103.2 (72.5-141.7) | 2 (1.4-2.7) | 92.6 (61.2-135.7) | 1.6 (1-2.4) | -0.29 (-1.44-0.87) |
| Acute hepatitis B | Socialist Republic of Viet Nam | 24780.2 (14829.6-42919.7) | 48.5 (28.4-85.1) | 10229.9 (7144.9-14134.8) | 9.3 (6.5-12.8) | -5.62 (-7.25--3.97) |
| Acute hepatitis B | Solomon Islands | 279.6 (96.1-583.1) | 96.6 (32.3-194) | 107.3 (50-204.4) | 17.3 (8-32.2) | -4.63 (-6.36--2.87) |
| Acute hepatitis B | State of Eritrea | 2646.9 (1127.2-4549.3) | 108.3 (46.4-173.8) | 3414.5 (1792.8-5424.5) | 64 (33.5-99.9) | -1.86 (-3.76-0.07) |
| Acute hepatitis B | State of Israel | 151.7 (125.7-182.1) | 3.1 (2.6-3.7) | 72.9 (60.6-89.1) | 0.7 (0.6-0.9) | -1.16 (-2.24--0.08) |
| Acute hepatitis B | State of Kuwait | 28.3 (22.1-36.9) | 2.2 (1.8-2.8) | 44 (30.8-61.9) | 0.9 (0.6-1.2) | -1.34 (-2.38--0.29) |
| Acute hepatitis B | State of Libya | 1416.6 (613.6-2671.1) | 51.4 (20.7-101) | 1560.9 (1086.6-2306.9) | 23.7 (16.6-35.3) | -2.73 (-4.88--0.54) |
| Acute hepatitis B | State of Qatar | 78.4 (58.2-105.7) | 33 (24.5-43.6) | 188.6 (125.6-270.9) | 9.4 (6.3-13.8) | -3.95 (-5.51--2.35) |
| Acute hepatitis B | Sultanate of Oman | 146.6 (69.2-285) | 12.3 (5.5-25.3) | 192.6 (108.1-292.7) | 5.2 (2.8-8.2) | -2.83 (-5.59-0.01) |
| Acute hepatitis B | Swiss Confederation | 140.6 (113.5-173) | 1.9 (1.6-2.4) | 80.4 (59.1-110) | 0.7 (0.5-1) | -1.38 (-2.32--0.43) |
| Acute hepatitis B | Syrian Arab Republic | 1269 (701.4-2161.1) | 14.7 (8-25.2) | 584.2 (392.9-805.9) | 4 (2.8-5.5) | -3.7 (-5.47--1.9) |
| Acute hepatitis B | Taiwan (Province of China) | 1320.9 (819.5-1979.1) | 6.2 (3.9-9.1) | 828.3 (489.8-1278.1) | 2.4 (1.5-3.7) | -1.25 (-2.51-0.02) |
| Acute hepatitis B | Togolese Republic | 424.5 (249.7-759) | 13.2 (7.7-21.8) | 719.4 (414.9-1163.5) | 10.3 (6.1-16.6) | -1.25 (-2.83-0.35) |
| Acute hepatitis B | Tokelau | 0.3 (0.1-0.4) | 16.4 (9.5-25.5) | 0.2 (0.1-0.3) | 13.9 (8.3-21.4) | -2.21 (-3.76--0.64) |
| Acute hepatitis B | Turkmenistan | 8836.3 (6717.8-11559.3) | 180.7 (142.1-227) | 1854 (1242.3-2725.2) | 35 (23.5-51.2) | -5.13 (-7.34--2.87) |
| Acute hepatitis B | Tuvalu | 1.9 (1.2-2.8) | 21 (13.6-30.8) | 0.9 (0.6-1.2) | 7.2 (5.1-9.9) | -3.29 (-4.83--1.73) |
| Acute hepatitis B | Ukraine | 6841.8 (5861.9-7989.4) | 16.1 (13.6-19.2) | 1405.6 (1009.4-1855.9) | 3.8 (2.8-4.9) | -2 (-3.51--0.47) |
| Acute hepatitis B | Union of the Comoros | 196.3 (96.2-310.8) | 54.6 (25.8-83.3) | 275.4 (148.5-436.8) | 41.2 (22.3-65.3) | -1.83 (-3.64-0.01) |
| Acute hepatitis B | United Arab Emirates | 300.1 (189.5-485.2) | 31.1 (19.4-50.3) | 1127.2 (750.7-1610.9) | 15.1 (10.6-21) | -1.8 (-3.51--0.05) |
| Acute hepatitis B | United Kingdom of Great Britain and Northern Ireland | 260 (160.1-412.3) | 0.4 (0.3-0.7) | 469.5 (345.4-652.7) | 0.6 (0.5-0.9) | 0.15 (-0.8-1.11) |
| Acute hepatitis B | United Mexican States | 2245.5 (1956.4-2590.5) | 2.2 (1.9-2.5) | 3230.1 (2654.9-4011.7) | 2.8 (2.3-3.6) | -1.53 (-2.95--0.08) |
| Acute hepatitis B | United Republic of Tanzania | 21669.3 (12638.6-31148.6) | 100.6 (57.7-138.9) | 16393 (9302-24163.5) | 34 (19.3-49) | -3.52 (-5.36--1.64) |
| Acute hepatitis B | United States of America | 3018.9 (2650.5-3464.3) | 1.2 (1-1.3) | 1789.5 (1460.6-2221.5) | 0.5 (0.4-0.6) | -1.11 (-2.15--0.07) |
| Acute hepatitis B | United States Virgin Islands | 1 (0.7-1.5) | 1 (0.7-1.4) | 0.7 (0.5-1.1) | 0.8 (0.6-1.2) | -0.5 (-1.57-0.57) |
| Acute hepatitis C | American Samoa | 0.5 (0.3-0.7) | 1.1 (0.6-1.7) | 0.2 (0.1-0.3) | 0.4 (0.3-0.6) | -1.11 (-2.46-0.26) |
| Acute hepatitis C | Antigua and Barbuda | 0.2 (0.1-0.2) | 0.3 (0.2-0.4) | 0.1 (0.1-0.2) | 0.1 (0.1-0.2) | -0.78 (-1.93-0.39) |
| Acute hepatitis C | Arab Republic of Egypt | 7272.5 (1530.5-43133.4) | 15.7 (2.5-99.6) | 1239.3 (367.7-5706) | 1.4 (0.4-6.1) | -4.87 (-7.61--2.04) |
| Acute hepatitis C | Argentine Republic | 253.5 (129.7-464.9) | 0.8 (0.4-1.4) | 135.1 (111.1-169.7) | 0.3 (0.2-0.3) | -1.42 (-2.73--0.08) |
| Acute hepatitis C | Australia | 80.2 (64.5-102.8) | 0.5 (0.4-0.6) | 48.9 (28.2-82) | 0.2 (0.1-0.3) | -0.4 (-1.37-0.58) |
| Acute hepatitis C | Barbados | 1.1 (0.8-1.5) | 0.4 (0.3-0.6) | 0.9 (0.6-1.2) | 0.2 (0.2-0.3) | -0.66 (-2.13-0.83) |
| Acute hepatitis C | Belize | 0.8 (0.5-1.2) | 0.5 (0.3-0.7) | 0.8 (0.5-1.2) | 0.2 (0.1-0.3) | -1.17 (-2.37-0.05) |
| Acute hepatitis C | Bermuda | 0.1 (0-0.1) | 0.1 (0.1-0.2) | 0.1 (0-0.1) | 0.1 (0-0.2) | -0.29 (-1.41-0.85) |
| Acute hepatitis C | Bolivarian Republic of Venezuela | 133.5 (83.4-201.3) | 0.7 (0.5-1.1) | 135.2 (96.3-183.8) | 0.5 (0.4-0.7) | -0.07 (-1.41-1.3) |
| Acute hepatitis C | Bosnia and Herzegovina | 8.4 (4-14.1) | 0.2 (0.1-0.3) | 2.9 (1.8-4.8) | 0.1 (0-0.1) | -1.1 (-2.62-0.45) |
| Acute hepatitis C | Brunei Darussalam | 28.5 (8.4-52.9) | 16.4 (5.5-29.4) | 6 (3-9.4) | 1.4 (0.7-2.1) | -5.38 (-7.2--3.54) |
| Acute hepatitis C | Burkina Faso | 1746 (425.1-3971.9) | 20.1 (4.9-47.7) | 1102.7 (375.2-2837.8) | 5.2 (1.8-12.4) | -2.85 (-4.9--0.76) |
| Acute hepatitis C | Canada | 128 (103.4-155) | 0.4 (0.3-0.5) | 38.5 (23.6-62.2) | 0.1 (0.1-0.1) | -0.73 (-1.63-0.18) |
| Acute hepatitis C | Central African Republic | 869.9 (182.1-2838.6) | 36.2 (8.1-123.6) | 526.5 (135.3-1412.5) | 11.4 (3-31.1) | -3.15 (-5.29--0.97) |
| Acute hepatitis C | Commonwealth of Dominica | 0.2 (0.1-0.3) | 0.2 (0.1-0.4) | 0.1 (0-0.1) | 0.1 (0.1-0.2) | -0.5 (-1.77-0.78) |
| Acute hepatitis C | Commonwealth of the Bahamas | 0.8 (0.6-1.1) | 0.4 (0.3-0.5) | 0.5 (0.3-0.8) | 0.1 (0.1-0.2) | -0.79 (-2.02-0.46) |
| Acute hepatitis C | Cook Islands | 0 (0-0.1) | 0.3 (0.1-0.4) | 0 (0-0) | 0.1 (0.1-0.2) | -0.64 (-1.78-0.53) |
| Acute hepatitis C | Czech Republic | 14.4 (9.4-21.7) | 0.1 (0.1-0.2) | 13.3 (8.6-20.8) | 0.1 (0.1-0.2) | -0.68 (-1.82-0.47) |
| Acute hepatitis C | Democratic People's Republic of Korea | 1163.1 (448.7-2849.1) | 5.7 (2.2-13.9) | 188.3 (84.6-341.1) | 0.6 (0.3-1.2) | -4.22 (-5.95--2.45) |
| Acute hepatitis C | Democratic Republic of Sao Tome and Principe | 6.5 (3.2-10.6) | 5.8 (2.9-9.8) | 1.7 (1-2.7) | 0.9 (0.6-1.4) | -4.3 (-6.24--2.33) |
| Acute hepatitis C | Democratic Republic of the Congo | 1366.8 (480.5-2823.4) | 3.9 (1.3-7.8) | 1229.2 (397.9-2238.1) | 1.7 (0.5-2.9) | -1.76 (-3.44--0.04) |
| Acute hepatitis C | Democratic Republic of Timor-Leste | 44 (8.5-131.6) | 6.1 (1.1-20.3) | 15 (5.9-34.8) | 1.2 (0.4-2.8) | -3.25 (-4.62--1.85) |
| Acute hepatitis C | Democratic Socialist Republic of Sri Lanka | 412.4 (210.6-718.5) | 2.6 (1.3-4.7) | 94.1 (58.3-152.3) | 0.4 (0.3-0.6) | -3.58 (-4.86--2.28) |
| Acute hepatitis C | Dominican Republic | 79.9 (53.1-109.8) | 1.2 (0.8-1.6) | 86.3 (61.8-124.8) | 0.8 (0.6-1.1) | -0.8 (-2.01-0.42) |
| Acute hepatitis C | Eastern Republic of Uruguay | 9.1 (5.9-12.7) | 0.3 (0.2-0.4) | 5.7 (4.2-8.1) | 0.1 (0.1-0.2) | -0.65 (-1.9-0.61) |
| Acute hepatitis C | Federal Democratic Republic of Ethiopia | 18053.5 (1181.1-52062.9) | 54.7 (3.8-159) | 12151.6 (2167.3-29682.3) | 15.5 (2.9-37.3) | -3.02 (-4.68--1.32) |
| Acute hepatitis C | Federal Democratic Republic of Nepal | 3434.5 (64-8064.8) | 18.5 (0.3-43.4) | 3738.7 (381.5-7954.1) | 12.8 (1.3-26.7) | -2.12 (-4.38-0.2) |
| Acute hepatitis C | Federal Republic of Germany | 354.1 (233.2-494.6) | 0.3 (0.2-0.5) | 112.2 (71.4-178.6) | 0.1 (0.1-0.2) | -0.79 (-1.71-0.14) |
| Acute hepatitis C | Federal Republic of Nigeria | 63421.4 (16202.2-186121.6) | 73.3 (18.8-224.5) | 9046.9 (3590.1-19977.3) | 4 (1.6-8.9) | -7.86 (-9.86--5.81) |
| Acute hepatitis C | Federal Republic of Somalia | 5707.9 (215.6-28402) | 107.1 (4-516.4) | 10714 (499-53212.4) | 79.1 (3.7-354.2) | -0.93 (-3.03-1.22) |
| Acute hepatitis C | Federated States of Micronesia | 8.4 (3.4-15.8) | 9.6 (3.8-18.1) | 1.6 (0.8-2.7) | 1.6 (0.8-2.7) | -3.41 (-4.68--2.12) |
| Acute hepatitis C | Federative Republic of Brazil | 1286 (1037.1-1618) | 1 (0.8-1.2) | 840.8 (647.3-1145.9) | 0.4 (0.3-0.5) | -1.62 (-2.97--0.24) |
| Acute hepatitis C | French Republic | 172 (133.4-218.2) | 0.3 (0.2-0.3) | 74.5 (45.6-122.8) | 0.1 (0-0.1) | -0.65 (-1.69-0.41) |
| Acute hepatitis C | Gabonese Republic | 49.2 (11.4-207.8) | 5.6 (1.2-25.3) | 12.9 (6.3-26.4) | 0.8 (0.4-1.7) | -4.25 (-6.04--2.43) |
| Acute hepatitis C | Georgia | 849.4 (626.6-1132.1) | 16.1 (11.7-21.9) | 16.2 (10.9-24.8) | 0.5 (0.3-0.8) | -3.6 (-4.92--2.27) |
| Acute hepatitis C | Grand Duchy of Luxembourg | 0.8 (0.5-1.1) | 0.2 (0.1-0.2) | 0.6 (0.3-1) | 0.1 (0-0.1) | -0.4 (-1.3-0.5) |
| Acute hepatitis C | Greenland | 0.1 (0-0.1) | 0.1 (0.1-0.2) | 0 (0-0.1) | 0.1 (0-0.1) | -1.43 (-2.58--0.26) |
| Acute hepatitis C | Grenada | 0.6 (0.4-0.9) | 0.8 (0.5-1.2) | 0.1 (0.1-0.2) | 0.1 (0.1-0.2) | -1.48 (-2.82--0.12) |
| Acute hepatitis C | Guam | 1.1 (0.6-2) | 0.9 (0.4-1.5) | 0.6 (0.4-0.9) | 0.4 (0.2-0.5) | -0.4 (-1.74-0.97) |
| Acute hepatitis C | Hashemite Kingdom of Jordan | 109.3 (49.4-223.7) | 4.9 (2.1-11.6) | 77 (51.2-118.1) | 0.8 (0.5-1.2) | -4.04 (-5.55--2.51) |
| Acute hepatitis C | Hellenic Republic | 31.6 (20.3-39.9) | 0.2 (0.2-0.3) | 18.7 (14.5-25.7) | 0.1 (0.1-0.2) | -0.6 (-1.95-0.76) |
| Acute hepatitis C | Hungary | 15.9 (9.8-24.6) | 0.2 (0.1-0.2) | 8.5 (4.7-14.7) | 0.1 (0-0.1) | -0.54 (-1.61-0.54) |
| Acute hepatitis C | Independent State of Papua New Guinea | 894.4 (96.5-3193.5) | 24.4 (2.5-89.3) | 351.5 (60.4-1075) | 3.5 (0.5-11) | -4.65 (-6.15--3.12) |
| Acute hepatitis C | Independent State of Samoa | 2.5 (1.1-4.3) | 1.7 (0.7-3.1) | 1.4 (0.8-2.2) | 0.7 (0.4-1.1) | -1.31 (-2.7-0.11) |
| Acute hepatitis C | Ireland | 11.9 (8.4-14.9) | 0.3 (0.2-0.4) | 5.5 (3.5-8.6) | 0.1 (0.1-0.1) | -0.45 (-1.45-0.56) |
| Acute hepatitis C | Islamic Republic of Afghanistan | 4013.8 (235.5-11445.2) | 44.8 (2.7-135.9) | 1368.3 (200.5-3728) | 6.3 (0.8-19.2) | -4.7 (-7.44--1.88) |
| Acute hepatitis C | Islamic Republic of Iran | 1491 (490.3-2487.8) | 3.1 (1.1-5.3) | 132.8 (85.5-215.6) | 0.2 (0.1-0.3) | -4.48 (-6.57--2.35) |
| Acute hepatitis C | Islamic Republic of Mauritania | 498.1 (105-1754.4) | 28.8 (6.4-100) | 69 (34.3-136.9) | 2 (0.9-3.9) | -6.22 (-8.59--3.79) |
| Acute hepatitis C | Islamic Republic of Pakistan | 28997.1 (1754.2-62569.7) | 34.9 (1.8-72.6) | 47580.6 (8567.2-92859.5) | 25.8 (4.5-49) | -1.06 (-3.14-1.06) |
| Acute hepatitis C | Jamaica | 9.2 (6.1-13) | 0.5 (0.3-0.6) | 6.3 (4.5-8.9) | 0.2 (0.2-0.3) | -0.46 (-1.74-0.82) |
| Acute hepatitis C | Japan | 1377.9 (1170.1-1608) | 0.9 (0.8-1) | 364.1 (222.9-589.2) | 0.2 (0.1-0.3) | -1.87 (-3.12--0.61) |
| Acute hepatitis C | Kingdom of Bahrain | 3.1 (1.2-10.6) | 0.9 (0.3-3.6) | 3 (1.4-7.1) | 0.2 (0.1-0.5) | -3.91 (-6.19--1.58) |
| Acute hepatitis C | Kingdom of Belgium | 43.6 (32.1-54.9) | 0.4 (0.3-0.4) | 15.2 (9.8-24.2) | 0.1 (0.1-0.2) | -0.83 (-1.84-0.2) |
| Acute hepatitis C | Kingdom of Bhutan | 75.4 (2.7-184.6) | 13.4 (0.4-33.3) | 51.8 (9.4-120.1) | 7.2 (1.3-16.8) | -3.14 (-5.15--1.09) |
| Acute hepatitis C | Kingdom of Cambodia | 9463.4 (2873.6-23150.9) | 124 (35.3-311.1) | 2358.7 (647.8-6980.2) | 14.7 (4-43) | -5.97 (-7.83--4.07) |
| Acute hepatitis C | Kingdom of Denmark | 7.5 (5.3-10.7) | 0.1 (0.1-0.2) | 5.5 (3-9.2) | 0.1 (0-0.1) | -0.64 (-1.57-0.3) |
| Acute hepatitis C | Kingdom of Eswatini | 36.1 (10.3-81.4) | 6.8 (1.8-15.8) | 15.4 (7.5-23.8) | 1.7 (0.9-2.6) | -2.9 (-5.1--0.65) |
| Acute hepatitis C | Kingdom of Lesotho | 92.6 (18.7-232.3) | 8 (1.6-20) | 51.3 (22.4-94.2) | 3.4 (1.5-6.1) | -1.2 (-3.49-1.14) |
| Acute hepatitis C | Kingdom of Morocco | 742.3 (178.4-2508) | 3.6 (0.8-13.1) | 253 (100.4-884.9) | 0.7 (0.3-2.4) | -2.98 (-5.08--0.84) |
| Acute hepatitis C | Kingdom of Norway | 4.2 (2.7-6.3) | 0.1 (0.1-0.1) | 3.4 (1.9-5.8) | 0 (0-0.1) | -0.07 (-1-0.87) |
| Acute hepatitis C | Kingdom of Saudi Arabia | 4414.7 (1541-10549.5) | 47.9 (15.4-112.9) | 492.1 (287.7-913.5) | 1.6 (0.9-2.8) | -6.34 (-8.21--4.44) |
| Acute hepatitis C | Kingdom of Spain | 158.2 (130.1-199.7) | 0.4 (0.3-0.5) | 70.8 (45.2-113.7) | 0.1 (0.1-0.2) | -0.43 (-1.4-0.55) |
| Acute hepatitis C | Kingdom of Sweden | 13.9 (11.1-17.8) | 0.1 (0.1-0.2) | 9.4 (6-14.6) | 0.1 (0-0.1) | 0.01 (-0.78-0.81) |
| Acute hepatitis C | Kingdom of Thailand | 1028.6 (400.1-2003) | 1.9 (0.7-3.8) | 406.1 (269.1-560.5) | 0.6 (0.4-0.8) | -1.49 (-3.31-0.36) |
| Acute hepatitis C | Kingdom of the Netherlands | 14.5 (10.3-21.1) | 0.1 (0.1-0.1) | 11.5 (5.9-20.8) | 0 (0-0.1) | -0.3 (-1.21-0.61) |
| Acute hepatitis C | Kingdom of Tonga | 48.8 (16.4-96.8) | 61.1 (21.1-122.7) | 12.6 (6.7-22.3) | 13.2 (7-23.7) | -4.2 (-5.89--2.48) |
| Acute hepatitis C | Kyrgyz Republic | 144.6 (80.4-265) | 2.9 (1.6-5.4) | 43.6 (25-70.8) | 0.6 (0.3-1) | -5.4 (-7.61--3.14) |
| Acute hepatitis C | Lao People's Democratic Republic | 1980.5 (491.9-5401.9) | 54 (13.4-155.6) | 262.1 (99.7-633.9) | 3.8 (1.4-9.3) | -6.46 (-8.22--4.67) |
| Acute hepatitis C | Lebanese Republic | 74.8 (27.6-125.6) | 3 (1.1-5) | 36.7 (24.9-52.2) | 0.6 (0.4-0.9) | -2.48 (-3.92--1.01) |
| Acute hepatitis C | Malaysia | 251.7 (143.2-401.3) | 1.5 (0.8-2.3) | 174.5 (117.4-249.7) | 0.6 (0.4-0.8) | -1.5 (-3.86-0.93) |
| Acute hepatitis C | Mongolia | 45.4 (24.1-77.5) | 1.7 (1-2.8) | 28.4 (15.5-52.3) | 0.8 (0.4-1.4) | -4.03 (-6.36--1.65) |
| Acute hepatitis C | Montenegro | 0.8 (0.5-1.2) | 0.1 (0.1-0.2) | 0.6 (0.3-1) | 0.1 (0.1-0.2) | -0.27 (-1.33-0.81) |
| Acute hepatitis C | New Zealand | 3.7 (2.1-6.1) | 0.1 (0.1-0.2) | 5.5 (2.6-10.2) | 0.1 (0-0.2) | -0.53 (-1.49-0.43) |
| Acute hepatitis C | North Macedonia | 4.4 (2.6-6.7) | 0.2 (0.1-0.4) | 2.5 (1.5-4.1) | 0.1 (0.1-0.2) | -1.2 (-2.42-0.04) |
| Acute hepatitis C | Northern Mariana Islands | 0.4 (0.2-0.7) | 1 (0.6-1.5) | 0.2 (0.2-0.3) | 0.4 (0.3-0.6) | -1.28 (-2.82-0.3) |
| Acute hepatitis C | Palestine | 60.1 (22.5-133.5) | 4.7 (1.6-11.6) | 22.3 (14.9-31.4) | 0.6 (0.4-0.9) | -3.55 (-5.1--1.98) |
| Acute hepatitis C | People's Democratic Republic of Algeria | 506.8 (199-1516.7) | 2.4 (0.9-7.9) | 164.8 (70.8-500.5) | 0.4 (0.2-1.2) | -2.98 (-5.09--0.83) |
| Acute hepatitis C | People's Republic of Bangladesh | 16455 (450.2-37300.2) | 21.4 (0.5-49.7) | 9701.4 (1434.6-19149.1) | 6.1 (0.9-12.1) | -3.42 (-5.51--1.28) |
| Acute hepatitis C | People's Republic of China | 89581.5 (35216.1-167126) | 8.4 (3.3-15.8) | 3408.2 (2180.5-5828.8) | 0.2 (0.1-0.4) | -7.21 (-8.96--5.43) |
| Acute hepatitis C | Plurinational State of Bolivia | 69.7 (25.1-123.4) | 1.1 (0.4-1.8) | 48.3 (31.3-69) | 0.4 (0.3-0.6) | -1.34 (-2.89-0.24) |
| Acute hepatitis C | Portuguese Republic | 56.5 (37.1-76.4) | 0.5 (0.3-0.7) | 17.9 (11-29.3) | 0.1 (0.1-0.2) | -1.48 (-2.75--0.19) |
| Acute hepatitis C | Principality of Andorra | 4.3 (1.7-7.5) | 7.4 (3-12.8) | 1.3 (0.6-2.7) | 1 (0.5-2.1) | -2.52 (-3.86--1.16) |
| Acute hepatitis C | Principality of Monaco | 0 (0-0.1) | 0.1 (0.1-0.2) | 0.1 (0-0.1) | 0.1 (0.1-0.2) | -0.14 (-1.06-0.78) |
| Acute hepatitis C | Puerto Rico | 58.5 (43.1-73.6) | 1.6 (1.2-2.1) | 10.4 (7.6-14.1) | 0.3 (0.2-0.4) | -1.18 (-2.4-0.05) |
| Acute hepatitis C | Republic of Albania | 6.8 (3.2-11.1) | 0.2 (0.1-0.4) | 3 (1.7-5) | 0.1 (0.1-0.2) | -1.88 (-3.19--0.56) |
| Acute hepatitis C | Republic of Angola | 7198.8 (1091.4-27239.4) | 80.9 (11-330.4) | 1141.6 (300.5-4521.4) | 4.1 (1-17.9) | -7.15 (-9.33--4.91) |
| Acute hepatitis C | Republic of Armenia | 46.9 (26.1-74.7) | 1.3 (0.7-2.1) | 10.7 (6.7-17.6) | 0.4 (0.2-0.7) | -2.86 (-4.35--1.35) |
| Acute hepatitis C | Republic of Austria | 18.6 (12.5-25.2) | 0.2 (0.1-0.3) | 8.7 (4.8-15.6) | 0.1 (0-0.1) | -0.45 (-1.42-0.53) |
| Acute hepatitis C | Republic of Azerbaijan | 87.3 (52-130.4) | 1.1 (0.6-1.6) | 38 (21.3-66) | 0.4 (0.2-0.8) | -4.15 (-5.97--2.3) |
| Acute hepatitis C | Republic of Belarus | 79.7 (40.5-118.7) | 0.8 (0.4-1.3) | 47 (33.3-62.5) | 0.5 (0.4-0.7) | -1.75 (-3.44--0.02) |
| Acute hepatitis C | Republic of Benin | 599.9 (190.5-1033.8) | 13.5 (4.2-24.3) | 263.7 (138.5-425.8) | 2.2 (1.2-3.6) | -4.25 (-6.18--2.29) |
| Acute hepatitis C | Republic of Botswana | 43.7 (14.2-86.8) | 4.8 (1.5-9.9) | 15.9 (9-24.3) | 0.8 (0.5-1.2) | -3.58 (-5.49--1.64) |
| Acute hepatitis C | Republic of Bulgaria | 45.4 (30.2-62.9) | 0.5 (0.3-0.7) | 12.7 (8.4-18.3) | 0.2 (0.1-0.2) | -0.77 (-2.1-0.58) |
| Acute hepatitis C | Republic of Burundi | 1630.4 (336.4-4810.4) | 37.5 (8.1-101.8) | 1350 (297.7-3628.1) | 14.1 (3.1-36.6) | -1.39 (-3.2-0.45) |
| Acute hepatitis C | Republic of Cabo Verde | 8.5 (3.7-13.9) | 2.9 (1.2-4.8) | 4.1 (2.6-6.1) | 0.8 (0.5-1.1) | -3.54 (-5.8--1.23) |
| Acute hepatitis C | Republic of Cameroon | 434.8 (225-770.7) | 4.6 (2.3-7.6) | 280.2 (135.1-478.5) | 1 (0.5-1.7) | -2.22 (-3.74--0.67) |
| Acute hepatitis C | Republic of Chad | 1635.5 (396.3-3760) | 30.5 (7.7-73.3) | 1635.3 (510.4-3463.1) | 11.3 (3.5-24.1) | -1.78 (-3.87-0.36) |
| Acute hepatitis C | Republic of Chile | 55.3 (40.9-71.1) | 0.5 (0.3-0.6) | 30.7 (20.5-48.1) | 0.1 (0.1-0.2) | -0.93 (-2.17-0.33) |
| Acute hepatitis C | Republic of Colombia | 219.7 (153.9-294.1) | 0.7 (0.5-0.9) | 91.1 (62.7-135.8) | 0.2 (0.1-0.3) | -0.94 (-2.28-0.42) |
| Acute hepatitis C | Republic of Costa Rica | 23.1 (17-31.2) | 0.8 (0.6-1.1) | 16.8 (13.3-22.1) | 0.4 (0.3-0.5) | -0.58 (-1.74-0.58) |
| Acute hepatitis C | Republic of C么te d'Ivoire | 1012.8 (361.4-1990.5) | 9.7 (3.4-18.9) | 370.7 (206.9-585.7) | 1.5 (0.8-2.3) | -4.04 (-5.92--2.12) |
| Acute hepatitis C | Republic of Croatia | 4.5 (2.5-7.7) | 0.1 (0.1-0.2) | 3.6 (1.9-6.4) | 0.1 (0-0.1) | -0.31 (-1.34-0.73) |
| Acute hepatitis C | Republic of Cuba | 29.3 (18.6-43.2) | 0.3 (0.2-0.4) | 15.3 (9-25.8) | 0.1 (0.1-0.2) | -1.07 (-2.33-0.21) |
| Acute hepatitis C | Republic of Cyprus | 0.9 (0.5-1.4) | 0.1 (0.1-0.2) | 1 (0.5-1.8) | 0.1 (0-0.1) | -0.74 (-1.73-0.26) |
| Acute hepatitis C | Republic of Djibouti | 28.6 (3.9-99) | 9.4 (1.4-31.7) | 45.4 (15.1-117.8) | 4.4 (1.5-11.4) | -1.51 (-3.35-0.37) |
| Acute hepatitis C | Republic of Ecuador | 125.4 (88.5-167.7) | 1.3 (0.9-1.7) | 27.5 (19.4-40.6) | 0.2 (0.1-0.2) | -1.19 (-2.53-0.18) |
| Acute hepatitis C | Republic of El Salvador | 63.7 (34.2-99.1) | 1.1 (0.6-1.7) | 19.1 (12.7-29) | 0.3 (0.2-0.5) | -1.31 (-2.47--0.14) |
| Acute hepatitis C | Republic of Equatorial Guinea | 9 (3-19.5) | 2.3 (0.7-4.7) | 5.5 (2.9-10.3) | 0.5 (0.2-0.9) | -2.15 (-3.4--0.88) |
| Acute hepatitis C | Republic of Estonia | 11.9 (8.8-15.7) | 0.8 (0.6-1.1) | 2.1 (1.2-3.7) | 0.2 (0.1-0.3) | -2.85 (-4.32--1.35) |
| Acute hepatitis C | Republic of Fiji | 20 (9.5-38.4) | 2.9 (1.4-5.6) | 5.4 (3.3-8.8) | 0.6 (0.4-0.9) | -3.25 (-4.66--1.81) |
| Acute hepatitis C | Republic of Finland | 8.3 (5.6-11.9) | 0.1 (0.1-0.2) | 6.1 (3.4-10.3) | 0.1 (0-0.1) | -0.38 (-1.17-0.42) |
| Acute hepatitis C | Republic of Ghana | 4645.3 (2296.9-7614.7) | 35.9 (18-59.2) | 591.6 (275.4-1072.1) | 1.9 (0.9-3.4) | -6.59 (-8.12--5.05) |
| Acute hepatitis C | Republic of Guatemala | 191.4 (105.6-353.3) | 1.9 (1.1-3.2) | 64.5 (46.8-89.8) | 0.4 (0.3-0.6) | -1.18 (-2.64-0.3) |
| Acute hepatitis C | Republic of Guinea | 1634.7 (321.8-3784.3) | 29.1 (5.8-71.8) | 649.7 (251.7-1376.1) | 5.8 (2.2-12) | -3.49 (-5.58--1.35) |
| Acute hepatitis C | Republic of Guinea-Bissau | 246.8 (35.9-536.6) | 28.3 (4.2-60.4) | 85.7 (27.4-156.6) | 5.1 (1.8-9.2) | -3.81 (-5.96--1.61) |
| Acute hepatitis C | Republic of Guyana | 9.6 (7.6-11.6) | 1.5 (1.2-1.8) | 5.4 (3.8-7.4) | 0.7 (0.5-1) | -0.97 (-2.37-0.46) |
| Acute hepatitis C | Republic of Haiti | 152.8 (30.4-308.2) | 2.9 (0.5-6.1) | 146.2 (43.7-280.3) | 1.3 (0.4-2.5) | -1.59 (-3.61-0.48) |
| Acute hepatitis C | Republic of Honduras | 260.8 (78.8-575) | 4.6 (1.4-10.2) | 120.9 (55.6-221) | 1.4 (0.6-2.4) | -2.55 (-4.06--1.02) |
| Acute hepatitis C | Republic of Iceland | 0.2 (0.1-0.3) | 0.1 (0-0.1) | 0.2 (0.1-0.4) | 0.1 (0-0.1) | -0.24 (-1.01-0.53) |
| Acute hepatitis C | Republic of India | 102688.2 (8796.7-177433.6) | 12.2 (1-21.8) | 106008.1 (27603.9-192727.4) | 7.9 (2.1-14.3) | -0.94 (-3.37-1.55) |
| Acute hepatitis C | Republic of Indonesia | 44401.6 (20320.3-88731.3) | 28.2 (12.3-58.5) | 18849.1 (9568.1-32828) | 6.6 (3.5-11.4) | -3.4 (-5.01--1.76) |
| Acute hepatitis C | Republic of Iraq | 150.6 (68.5-422.1) | 0.9 (0.4-3.1) | 126.6 (63.7-294.1) | 0.4 (0.2-0.9) | -2.73 (-5--0.4) |
[truncated: 52,867 more chars]
